# The Risk Factors and Mortality Among Patients With Different Combination Patterns of Opioids and Benzodiazepines: A Retrospective Study

**DOI:** 10.1002/prp2.70215

**Published:** 2026-01-15

**Authors:** Fang‐Yu Su, Ming‐Che Tsai, Yee‐Yung Ng, Shiao‐Chi Wu

**Affiliations:** ^1^ Institute of Health and Welfare Policy, School of Medicine National Yang Ming Chiao Tung University Taipei Taiwan; ^2^ Department of Pharmacy Linkou Chang Gung Memorial Hospital Taoyuan Taiwan; ^3^ Department of Medicine, School of Medicine Fu Jen Catholic University New Taipei City Taiwan; ^4^ Department of Long‐Term Care, College of Nursing Asia University Taichung Taiwan

**Keywords:** adverse outcome, benzodiazepine, co‐prescription, mortality, opioid users

## Abstract

The concurrent use of opioids and benzodiazepines (BZDs) has raised safety concerns due to increased risks of respiratory depression and death. To evaluate mortality risks linked to different patterns of co‐prescription, we conducted a retrospective cohort study using data from a private hospital system between 2008 and 2018. A total of 418 549 opioid users were classified into four groups based on BZD use: opioids only, past use, continuous use, and new initiation. Cox proportional hazards regression model was used to estimate adjusted hazard ratios (aHRs) for 1‐year all‐cause mortality following 1‐year of concomitant use, adjusting for past medical history and Charlson Comorbidity Index (CCI). Compared to the opioids‐only group, mortality risks were significantly higher in the continuous (aHR = 1.78), new (aHR = 1.56), and past BZD use groups (aHR = 1.29). Higher mortality was also associated with older age, cancer, and greater comorbidity burden. These findings emphasize the need for cautious prescribing of opioids and BZDs, especially in older adults and those with complex medical conditions.

## 1 Introduction

Patients with chronic pain who have a history of gastrointestinal ulcers, renal insufficiency, or concomitant use of warfarin are at elevated risk for complications from nonsteroidal anti‐inflammatory drugs (NSAIDs), the most commonly used analgesics. For these patients, opioids are often considered as an alternative treatment option. However, individuals with chronic pain frequently experience comorbid conditions such as anxiety, depression, insomnia, and substance use disorders [1, 2]. As such, the therapeutic goals extend beyond pain relief to include improvements in emotional well‐being, sleep quality, and overall quality of life.

To address the complex needs of patients with chronic pain, opioids are frequently co‐prescribed with psychotropic medications, including antidepressants, antiepileptics, antipsychotics, and sedative‐hypnotics such as benzodiazepines (BZDs) [3]. Among antiepileptics, pregabalin and gabapentin have demonstrated efficacy and are recommended as first‐line adjuvant agents for neuropathic pain management [3]. Valproic acid, another widely used antiepileptic, has also been reported to influence sleep architecture and, in rare cases, to exacerbate obstructive sleep apnea and induce central sleep apnea. This mechanism is thought to involve disruption of central respiratory drive or instability in ventilatory control, underscoring the importance of recognizing this potential adverse effect [4]. In addition to psychotropic agents, alcohol is another potent central nervous system depressant, and its concomitant use with opioids or BZDs may further amplify the risk of respiratory depression.

Opioids and benzodiazepines are both strong central nervous system depressants, and their concurrent use has been recognized as particularly hazardous. When taken together, their respiratory depressant effects may act synergistically rather than additively, especially within brainstem regions critical for breathing control. Experimental and clinical studies have demonstrated that simultaneous activation of μ‐opioid and GABA‐A receptors markedly reduces central respiratory drive, thereby predisposing patients to severe and potentially fatal respiratory depression [5, 6].

Despite this practice, both the Australian clinical guidelines [7] and the U.S. Centers for Disease Control and Prevention (CDC) [8, 9] strongly advise against concurrent use of opioids and BZDs due to the increased risk of respiratory depression and death. Similarly, the 2023 American Geriatrics Society (AGS) Beers Criteria [10] recommend avoiding this combination in older adults, given their heightened vulnerability to adverse drug events, including severe respiratory compromise. Several U.S. studies have examined the outcomes of opioid‐BZD co‐prescription in specific groups, such as military veterans [11], people living with HIV [12], residents of North Carolina [13, 14], commercially insured individuals [15], retired veterans with post‐traumatic stress disorder (PTSD) [16], and patients with severe chronic obstructive pulmonary disease (COPD) [17].

In Taiwan, a nationwide study by the National Health Research Institute (NHRI) documented rising trends in the use of opioid analgesics—including morphine, fentanyl, pethidine, codeine, and buprenorphine—from 2002 to 2014, with the standardized defined daily dose per million inhabitants per day (S‐DDD/m/d) increasing by 41% (631.2–889.5) [18]. Moreover, total annual opioid consumption nearly doubled between 2008 and 2018 [19].

Despite these increases, evidence on the concurrent use of opioids and BZDs in Taiwan remains limited. In particular, little is known about its association with patient mortality. This gap is clinically important because Western studies have consistently demonstrated elevated risks. However, differences in healthcare systems, prescribing practices, and patient characteristics raise uncertainty about whether these findings can be generalized to Asian populations. To address this, the present study evaluated the mortality risk associated with different patterns of BZD use among patients receiving opioids for chronic pain within a hospital‐based cohort in Taiwan.

## 2 Methods

### 2.1 Data Source

This retrospective cohort study utilized data from the Chang Gung Research Database (CGRD), which comprises electronic medical records from the Chang Gung Memorial Hospital (CGMH) system—the largest healthcare system in Taiwan. The CGMH system includes three medical centers (Taipei, Linkou, and Kaohsiung branches), two regional hospitals (Keelung and Chiayi), and three district hospitals (Taoyuan, Fengshan, and Yunlin).

A previous study [20] using the CGRD reported that from 1997 to 2010, inpatient and outpatient cases from the CGMH system accounted for 12.4% and 21.2%, respectively, of the National Health Insurance Research Database (NHIRD), confirming CGMH as the largest healthcare provider in Taiwan. The NHIRD covers more than 99.9% of Taiwan's population [21]. The CGRD contains a range of data, including demographics, inpatient and outpatient data, diagnostic codes, details of prescriptions, and reports of image and functional examinations. The diagnostic codes in the CGRD have been demonstrated to be both accurate and valid [22]. All data are stored at Chang Gung Memorial Hospital, and only de‐identified results are available for analysis.

### 2.2 Study Design

The study cohort included patients aged ≥ 20 years who received opioid prescriptions (Anatomical Therapeutic Chemical [ATC] codes: N02AA, N02AB, N02AE, N02AX) within the CGMH system. Patterns of co‐prescription involving opioids and benzodiazepines (BZDs; ATC codes: N03AE, N05BA, N05CD, N05CF) were examined using electronic medical records between January 1, 2008, and December 31, 2018. Both inpatient and outpatient prescriptions were included. Patients were excluded if they received only one time of opioid prescription during the study period or if they were treated with methadone or buprenorphine for opioid dependence (ATC codes: N07BC). A full list of ATC codes and drug names is provided in Data S1.

Although Taiwan's healthcare system allows patients to seek medical care freely across institutions, opioid prescribing is strictly regulated by the National Health Insurance (NHI). All opioid prescriptions are centrally recorded in the NHI database. Consequently, once a patient has received an opioid prescription from one hospital, it is difficult to obtain additional opioids from other institutions. A warning message appears on the hospital computer screen to alert physicians when an opioid and its prescribed amount have already been issued, reminding them not to prescribe the opioid again. The cohort showed high treatment continuity, with an average of 8.1 prescription fills per patient per year, indicating that patients were consistently followed within the Chang Gung system.

**FIGURE 1:**
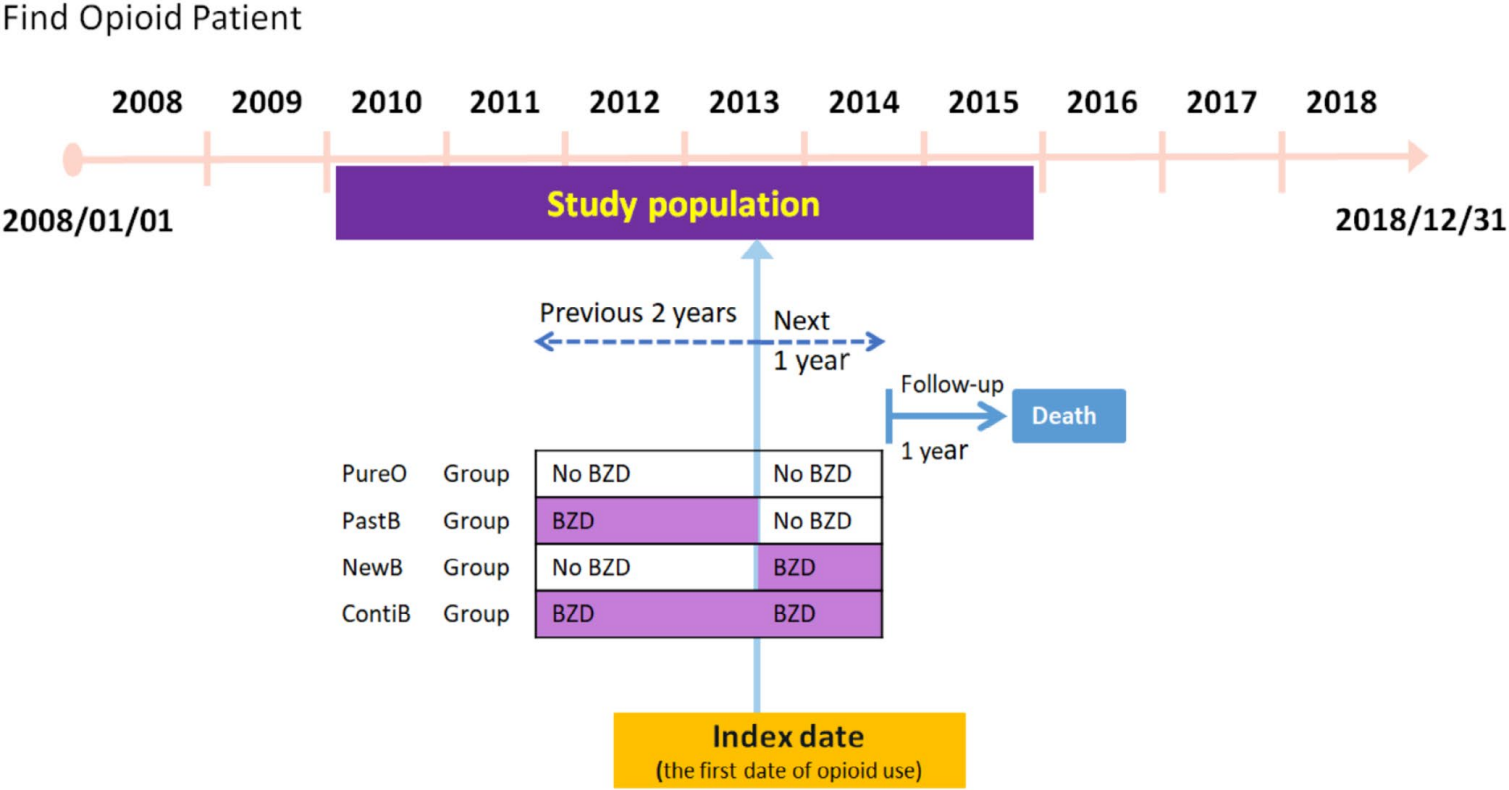
Scheme of the retrospective cohort study design.

The index date was defined as the date of each patient's first opioid prescription. BZD exposure was assessed across two timeframes: (1) the 2 years prior to the index date [23] and (2) the one‐year following the index date [24]. Based on BZD use relative to this date, patients were categorized into four mutually exclusive groups: (1) opioid only (PureO); (2) new BZD users who initiated BZDs after the index date (NewB); (3) past BZD users with a history of BZD use before but not on the index date (PastB); and (4) continuing BZD users with overlapping BZD use on the index date (ContiB). It should be noted that NewB indicates an absence of BZD prescriptions for at least 2 years before the index opioid prescription (Figure 1).

The study protocol was approved by the Institutional Review Board of Chang Gung Medical Foundation (IRB No. 201801306B0).

### 2.3 Dependent Variable

We defined the index date as the first day of opioid use. Patients were required to have continuous co‐use for 1 year from the index date [24], after which a 1‐year follow‐up period was conducted to assess adverse outcomes. Only patients who survived the entire 1‐year co‐use period were included in the final analysis. The primary objective was to evaluate all‐cause mortality and identify associated risk factors during the subsequent 1‐year follow‐up. Deaths were classified according to the hospital's disease statistics file, coded as “4” (in‐hospital death) or “A” (against advice discharge under critical condition, critical AAD) [25] “critical AAD” refers to patients who leave the hospital in critical condition and are at risk of dying at home, outside the hospital. This coding has been validated as a proxy for out‐of‐hospital mortality [25]. In Taiwan, some terminally ill patients choose AAD in line with the cultural belief of “fallen leaves return to their roots,” [26, 27] which emphasizes dying at home to reunite spiritually with ancestors and to avoid becoming a “wandering soul.” This practice is regarded as bringing peace to the deceased and blessings to their descendants, and AAD often reflects the wish to return home for the final moments of life.

### 2.4 Independent Variables

The main exposure of interest was the pattern of BZD use in patients who received opioid prescriptions. To evaluate potential overlap between opioid and BZD treatment, we examined prescription dates together with days of supply from both inpatient and outpatient pharmacy records. Based on these data, patients were categorized into four mutually exclusive groups: ContiB, PastB, NewB, and PureO.

### 2.5 Control Variables

Control variables included both demographic and clinical characteristics. Demographic data comprised sex and age, which were categorized into five groups: 20–44, 45–64, 65–74, 75–84, and ≥ 85 years. Past medical history included cancer (ICD‐9‐CM 140–239), anxiety (ICD‐9‐CM 300.0), depression (ICD‐9‐CM 292.2, 292.3, 300.4, 311), stroke or transient ischemic attack (ICD‐9‐CM 430–437, 435.9), chronic obstructive pulmonary disease (COPD; ICD‐9‐CM 490–496), and chronic kidney disease (CKD; ICD‐9‐CM 580–587) [28]. These conditions were identified using both outpatient and inpatient claims within 1 year prior to the index date, requiring at least two outpatient diagnoses or one inpatient diagnosis with relevant primary or secondary ICD codes.

Comorbidity burden was quantified using the D‐M Charlson Comorbidity Index (CCI) [29], and categorized into four levels: CCI = 0 (no comorbidities), CCI = 1 (mild), CCI = 2 (moderate), and CCI ≥ 3 (severe). This classification allowed for a systematic assessment of patients' baseline health status and its impact on clinical outcomes.

### 2.6 Data Analysis

All statistical analyses were conducted using the SAS System for Windows (version 9.4, SAS Institute, Cary, NC). Descriptive statistics were presented as frequency and percentage to describe the demographic characteristics across combination groups. Cox proportional hazards regression models were applied to examine the association between adverse outcomes and different opioid‐BZD combination groups. The PureO group (patients receiving opioids only) was used as the reference category for hazard ratio calculations. Adjusted hazard ratios (aHRs) and 95% confidence intervals (CIs) were calculated. A two‐tailed *p*‐value < 0.05 was considered statistically significant. Variance inflation factors (VIFs) were used to assess multicollinearity among covariates, with a threshold of < 5 indicating low collinearity.

## 3 Results

### 3.1 The Demographics and Comorbidities of Opioid Users With and Without Benzodiazepine Use

A total of 418 549 patients prescribed opioids between 2008 and 2018 were included in the analysis. Among them, 264 789 (63.26%) were classified into the PureO group, 68 845 (16.45%) into the NewB group, 53 016 (12.67%) into the PastB group, and 31 899 (7.62%) into the ContiB group. The proportion of females was slightly higher than males in both the PureO (63.51% vs. 62.99%) and ContiB (7.78% vs. 7.44%) groups. Notably, younger patients aged 20–44 were more likely to belong to the PureO group (80.21%) compared to older age groups (45–64: 59.64%, 65–74: 52.09%, 75–84: 48.75%, ≥ 85: 50.45%). In terms of past medical history, cancer was most prevalent in the PureO group (57.11%), whereas lower proportions were noted for stroke or transient ischemic attack (33.09%), COPD (39.17%), CKD (36.21%), anxiety (16.36%), and depression (11.93%). Additionally, patients with higher Charlson Comorbidity Index (CCI) scores were more commonly observed in the ContiB group, suggesting a greater comorbidity burden in this subgroup (Table 1).

**TABLE 1:** Demographic and comorbidity characteristics of opioid users with and without benzodiazepine use.

Characteristic	All	PureO	NewB	PastB	ContiB	*p*
	*N*	%	%	%	%	
All	418 549	63.26	16.45	12.67	7.62	
Gender						< 0.001
Male	197 256	62.99	18.23	11.34	7.44	< 0.001
Female	221 293	63.51	14.86	13.85	7.78	
Age group						< 0.001
20–44	131 481	80.21	10.57	5.86	3.36	
45–64	155 651	59.64	18.84	12.89	8.63	
65–74	64 064	52.09	19.79	17.66	10.45	
75–84	49 809	48.75	19.70	20.45	11.10	
≧ 85	17 544	50.45	17.90	21.31	10.34	
Past medical history						
Cancer	117 086	57.11	20.42	13.28	9.19	< 0.001
Past history of stroke or TIA	21 816	33.09	17.64	33.87	15.41	< 0.001
COPD	23 882	39.17	18.50	28.10	14.24	< 0.001
CKD	24 883	36.21	20.84	28.85	14.11	< 0.001
Anxiety	17 057	16.36	10.63	52.03	20.99	< 0.001
Depression	8402	11.93	10.37	58.11	19.60	< 0.001
CCI score						< 0.001
CCI = 0	250 012	75.20	12.52	7.66	4.62	
CCI = 1	53 389	51.64	18.08	19.74	10.54	
CCI = 2	50 555	48.84	22.69	17.50	10.97	
CCI ≥ 3	64 593	37.97	25.42	22.41	14.20	

*Note:* Distribution among groups was analyzed by *x* ^*2*^‐square test.

Abbreviations: CKD, chronic kidney disease; COPD, chronic obstructive pulmonary disease; TIA, transient ischemia attack.

A detailed breakdown of the primary diagnoses is presented in Data S2.

### 3.2 One‐Year Mortality Rate After a Year of Concurrent Opioid and BZD Use

After completing 1‐year of continuous opioid‐BZD co‐use, patients were followed for an additional year. During this follow‐up period, male patients exhibited significantly higher all‐cause mortality rates than female patients across all groups (0.96% vs. 0.46% in the PureO group, 2.24% vs. 1.63% in the NewB group, 2.11% vs. 1.38% in the PastB group, and 2.77% vs. 2.11% in the ContiB group; *p* < 0.001). Age was also a significant predictor of mortality, with death rates increasing progressively across older age groups (*p* < 0.001). Furthermore, mortality was significantly associated with comorbidity burden, as demonstrated by higher all‐cause mortality rates in patients with higher Charlson Comorbidity Index (CCI) scores across all groups (*p* < 0.001; Table 2).

**TABLE 2:** All‐cause mortality during one‐year follow‐up by opioid‐BZD use patterns.

Variable	All		*p*	PureO		*p*	NewB		*p*	PastB		*p*	ContiB		*p*
	%	95% CI		%	95% CI		%	95% CI		%	95% CI		%	95% CI	
**Gender**			< 0.001			< 0.001			< 0.001			< 0.001			0.001
Male	1.46	(1.40–1.51)		0.96	(0.90–1.01)		2.24	(2.09–2.40)		2.11	(1.92–2.30)		2.77	(2.51–3.04)	
Female	0.89	(0.85–0.93)		0.46	(0.42–0.49)		1.63	(1.50–1.77)		1.38	(1.25–1.51)		2.11	(1.90–2.33)	
**Age group**			< 0.001			< 0.001			< 0.001			< 0.001			< 0.001
20–44	0.27	(0.24–0.30)		0.11	(0.09–0.13)		0.98	(0.82–1.14)		0.51	(0.35–0.67)		1.29	(0.96–1.62)	
45–64	1.14	(1.09–1.19)		0.68	(0.63–0.74)		2.03	(1.87–2.19)		1.24	(1.08–1.39)		2.20	(1.96–2.45)	
65–74	1.62	(1.53–1.72)		1.24	(1.12–1.36)		2.01	(1.77–2.26)		1.82	(1.57–2.07)		2.48	(2.11–2.85)	
75–84	2.26	(2.13–2.39)		1.84	(1.67–2.01)		2.46	(2.15–2.76)		2.59	(2.28–2.90)		3.13	(2.67–3.59)	
≧85	3.12	(2.87–3.38)		2.41	(2.09–2.73)		3.72	(3.06–4.39)		3.72	(3.11–4.33)		4.36	(3.42–5.30)	
**Past medical history**															
Cancer	2.53	(2.44–2.62)	< 0.001	1.64	(1.54–1.74)	< 0.001	3.92	(3.68–4.17)	< 0.001	2.97	(2.70–3.23)	< 0.001	4.37	(3.98–4.76)	< 0.001
Past history of stroke or TIA	2.70	(2.49–2.92)	< 0.001	2.09	(1.76–2.42)	< 0.001	2.47	(1.98–2.96)	0.017	3.02	(2.63–3.41)	< 0.001	3.57	(2.94–4.20)	< 0.001
COPD	2.51	(2.31–2.71)	< 0.001	1.85	(1.58–2.12)	< 0.001	3.33	(2.80–3.86)	< 0.001	2.52	(2.14–2.89)	< 0.001	3.26	(2.67–3.86)	0.001
CKD	3.03	(2.81–3.24)	< 0.001	2.00	(1.71–2.29)	< 0.001	3.37	(2.88–3.87)	< 0.001	3.64	(3.20–4.07)	< 0.001	3.90	(3.26–4.54)	< 0.001
Anxiety	1.30	(1.13–1.47)	0.082	0.75	(0.43–1.07)	0.689	1.16	(0.67–1.65)	0.013	1.25	(1.02–1.48)	0.001	1.90	(1.45–2.35)	0.032
Depression	1.64	(1.37–1.91)	0.001	1.00	(0.38–1.62)	0.239	2.07	(1.12–3.01)	0.806	1.72	(1.36–2.09)	0.862	1.58	(0.98–2.18)	0.023
**CCI score**			< 0.001			< 0.001			< 0.001			< 0.001			< 0.001
CCI = 0	0.26	(0.24–0.28)		0.20	(0.18–0.22)		0.44	(0.37–0.51)		0.33	(0.25–0.41)		0.63	(0.49–0.78)	
CCI = 1	0.74	(0.66–0.81)		0.54	(0.46–0.63)		0.93	(0.74–1.12)		0.79	(0.62–0.96)		1.24	(0.96–1.53)	
CCI = 2	2.03	(1.90–2.15)		1.57	(1.42–1.73)		2.89	(2.59–3.20)		1.78	(1.50–2.05)		2.65	(2.23–3.07)	
CCI ≥ 3	4.29	(4.14–4.45)		3.72	(3.00–4.00)		4.78	(4.45–5.11)		4.10	(3.77–4.42)		5.25	(4.79–5.70)	

*Note:* Distribution among groups was analyzed by χ^2^‐square test.

Abbreviations: CKD, chronic kidney disease; COPD, chronic obstructive pulmonary disease; TIA, transient ischemic attack.

### 3.3 Adjusted Hazards Ratios for One‐Year Mortality Following Opioid‐BZD Co‐Use

Cox regression showed that one‐year all‐cause mortality was significantly higher in all BZD co‐use groups compared with the PureO reference group. The ContiB group had the highest adjusted hazards ratio (aHR = 1.78), followed by NewB (aHR = 1.56) and PastB (aHR = 1.29) (all *p* < 0.001). Male patients had a significantly higher mortality risk compared with females (aHR = 1.28; *p* < 0.001). Advancing age was also strongly associated with increased mortality: aHRs were 1.85 for ages 45–64, 2.14 for 65–74, 2.81 for 75–84, and 4.06 for ≥ 85 years (all *p* < 0.001). Regarding past medical conditions, cancer was significantly associated with increased mortality risk (aHR = 1.49). Conversely, both COPD (aHR = 0.85) and anxiety (aHR = 0.80) were significantly associated with decreased mortality risk. No statistically significant associations were identified for prior stroke or TIA (aHR = 0.92), CKD (aHR = 0.97), or depression (aHR = 1.03).

## 4 Discussion

Among opioid‐prescribed patients, most were classified into the PureO group, followed by NewB, PastB, and ContiB groups. This distribution indicates that BZD co‐prescription was often discontinued as patients' clinical status improved, with only a small proportion maintaining continuous opioid‐BZD therapy (Table 1). Females had a slightly higher proportion than males in the PureO group (63.51% vs. 62.99%), the PastB group (13.85% vs. 11.34%), and the ContiB group (7.78% vs. 7.44%) (Table 1). This pattern may reflect females' greater pain sensitivity, lower opioid tolerance, and higher anxiety disorder prevalence [30, 31].

Although previous studies have shown that anxiety tends to decrease with age even in the absence of treatment, most anxiety disorders do not persist into old age. Nevertheless, patients with chronic diseases exhibit high prevalence rates of stress (68.7%), anxiety (51.1%), and depression (58.8%) [32]. As individuals age, they become increasingly susceptible to various chronic conditions [33, 34]. Moreover, anxiety disorders commonly co‐occur with each other and with other mental health conditions [35]. These findings support our observation that the ContiB group had a significantly higher proportion of patients aged ≥ 45 years and with CCI ≥ 1, compared to those aged < 45 years and with CCI = 0 (*p* < 0.001).

The PastB, NewB, and ContiB groups each had significantly higher 1‐year mortality risks compared with the PureO group, consistent with findings among Medicare beneficiaries [28] and retired veterans [36]. The 95% confidence intervals for the NewB and ContiB groups overlapped, indicating similar risk magnitudes. These results collectively reinforce that opioid‐BZD co‐prescription is associated with an elevated mortality risk [7, 8, 9], particularly among older patients and those with higher CCI scores (Tables 2 and 3). The clinical reality such as illness severity and psychiatric distress may confound the association between BZD co‐use and mortality, as some prescribing decisions at high doses are influenced by palliative care considerations and the prioritization of quality of life over long‐term survival. Therefore, patients in BZD (NewB or ContiB) groups may differ from opioid‐only users in illness severity, psychiatric distress, or palliative care needs. After controlling for the CCI, the risk of mortality in BZD groups is still higher than for the opioid‐only users. It might indicate that some unknown residual confounders were not found in this study.

**TABLE 3:** Adjusted hazard ratios for one‐year mortality by opioid‐BZD use patterns.

Predictive variable	Crude			Adjusted		
	cHR	95% CI	*p*	aHR	95% CI	*p*
Group (ref:PureO)						
NewB	2.85	(2.65–3.06)	< 0.0001	1.56	(1.45–1.68)	< 0.0001
PastB	2.46	(2.27–2.66)	< 0.0001	1.29	(1.18–1.40)	< 0.0001
ContiB	3.53	(3.24–3.84)	< 0.0001	1.78	(1.63–1.94)	< 0.0001
Gender (ref:Female)						
Male	1.64	(1.55–1.74)	< 0.0001	1.28	(1.28–1.36)	< 0.0001
Age group (ref: 20–44)						
45–64	4.27	(3.81–4.79)	< 0.0001	1.85	(1.64–2.08)	< 0.0001
65–74	6.11	(5.41–6.89)	< 0.0001	2.14	(1.89–2.43)	< 0.0001
75–84	8.52	(7.56–9.60)	< 0.0001	2.81	(2.48–3.19)	< 0.0001
≧ 85	11.79	(10.31–13.48)	< 0.0001	4.06	(3.52–4.67)	< 0.0001
Past medical history						
Cancer	4.12	(3.89–4.37)	< 0.0001	1.49	(1.39–1.61)	< 0.0001
Past history of stroke or TIA	2.53	(2.33–2.76)	< 0.0001	0.92	(0.84–1.01)	0.079
COPD	2.35	(2.16–2.56)	< 0.0001	0.85	(0.78–0.93)	0.000
CKD	2.95	(2.73–3.19)	< 0.0001	0.97	(0.89–1.06)	0.463
Anxiety	1.13	(0.99–1.29)	0.079	0.80	(0.69–0.92)	0.002
Depression	1.44	(1.22–1.70)	< 0.0001	1.03	(0.87–1.23)	0.714
CCI score (ref:CCI = 0)						
CCI = 1	2.84	(2.50–3.21)	< 0.0001	2.13	(1.87–2.42)	< 0.0001
CCI = 2	7.86	(7.13–8.68)	< 0.0001	4.67	(4.18–5.21)	< 0.0001
CCI ≥ 3	16.84	(15.46–18.34)	< 0.0001	8.52	(7.63–9.51)	< 0.0001

*Note:* Cox proportional hazards regression.

Abbreviations: CKD, chronic kidney disease; COPD, chronic obstructive pulmonary disease; TIA, transient ischemic attack.

Our findings indicate that among past medical conditions, cancer history was associated with the greatest increase in mortality risk. In contrast, COPD and anxiety were significantly associated with lower mortality, while depression, prior stroke/TIA, and CKD did not show significant associations (Table 3). Moreover, pain management in cancer often requires higher opioid doses [37], reflecting cancer's status as a leading cause of death globally and in Taiwan and Japan [38].

In this study, only 17 057 of 418 549 patients (4.07%) were identified as having anxiety. The proportions of patients with anxiety varied across exposure groups, with 16.36%, 10.63%, 52.03%, and 20.99% in the PureO, NewB, PastB, and ContiB groups, respectively (Table 1). Although patients prescribed opioids with long‐term concurrent BZD use, many had chronic pain comorbid with anxiety disorders, and anxiety disorders have been repeatedly shown to be associated with increased mortality across various physical health conditions [39]. In this study, a history of anxiety was associated with a non‐significant increase in mortality in the unadjusted Cox model (HR = 1.13; 95% CI: 0.99–1.29), but after multivariable adjustment, it was significantly associated with reduced mortality (aHR = 0.80; 95% CI: 0.69–0.92). The depression showed a significant association with increased mortality in the unadjusted analysis (HR = 1.44; 95% CI: 1.22–1.70), but this association became non‐significant after adjustment (aHR = 1.03; 95% CI: 0.87–1.23). These findings support that the elevated mortality risk may not stem directly from the depressive disorders themselves, but rather from their comorbidity with physical illnesses [40].

Furthermore, pharmacologic management of anxiety often involves antidepressants such as SSRIs (selective serotonin reuptake inhibitors), SNRIs (serotonin norepinephrine reuptake inhibitors), or TCAs (tricyclic antidepressants), many of which are also used as adjuvant analgesics for chronic pain. Consequently, patients with anxiety may have been preferentially treated with antidepressants rather than BZDs, potentially reducing the likelihood of concurrent opioid‐BZD exposure and attenuating mortality risk.

COPD was associated with higher mortality in the unadjusted model (HR = 2.35; 95% CI: 2.16–2.56), but with reduced mortality after adjustment (aHR = 0.85; 95% CI: 0.78–0.93). Similar paradoxical findings have been reported in older adults with COPD, where short‐ to intermediate‐term co‐use of opioids and sedatives increased respiratory complications and mortality, whereas longer‐term past co‐use was linked to lower risk, possibly reflecting healthy user effects or pharmacologic tolerance [17]. These results emphasize the need for cautious prescribing and close monitoring of opioid‐sedative co‐use in patients with COPD.

These findings underscore the importance of individualized risk–benefit assessments when managing BZD therapy, particularly among older adults. While concurrent opioid‐BZD use should generally be avoided, the therapeutic benefits of BZDs in selected patients must also be considered. Deprescribing decisions should be patient‐centered and cautiously implemented to avoid potential harm.

The 2023 update of the American Geriatrics Society (AGS) Beers Criteria identifies the concurrent use of opioids and benzodiazepines (BZDs) as potentially inappropriate for adults aged 65 years and older, due to heightened risks of adverse outcomes [10]. Our findings support this recommendation, demonstrating a significantly elevated risk of all‐cause mortality among older adults receiving concomitant opioid‐BZD therapy.

However, emerging evidence has raised concerns regarding the consequences of abrupt or non‐individualized deprescribing. Maust et al. [41] reported that discontinuation of BZDs in patients undergoing stable, long‐term treatment was associated with increased risks of mortality and other adverse events. In response, the American Society of Addiction Medicine (ASAM) issued updated guidelines in 2025, advising against automatic tapering or discontinuation without thorough clinical evaluation [42].

Overall, while BZDs are associated with potential harms, their therapeutic value should not be underestimated—particularly in select patient populations. Deprescribing decisions should be individualized, based on a careful balance between benefits and risks, especially in older adults or those receiving chronic treatment. Abrupt withdrawal may lead to unintended consequences; therefore, clinical judgment and shared decision‐making are essential in guiding safe and appropriate BZD management. In addition, a potential dose–response relationship should be considered, as higher doses of opioids, BZDs, or both may further amplify the risk of respiratory depression and adverse outcomes. This clinical reality may also confound the observed association between BZD co‐use and mortality, since prescribing decisions at higher doses are often influenced by palliative care needs and the prioritization of quality of life over long‐term survival.

## 5 Limitations and Strength

This study has several limitations. First, mortality outcomes were based on in‐hospital deaths and critical AAD status, which may not capture all out‐of‐hospital deaths; however, validation against the Taiwan Death Registry demonstrated over 98% accuracy [25]. Therefore, the risk of outcome misclassification was minimized in this study. Future linkage to the National Death Registry would allow capture of out‐of‐hospital and non‐natural deaths, further enhancing outcome validity. Second, actual medication adherence could not be confirmed [28], and data on certain potential respiratory depressants (e.g., alcohol) were unavailable. Third, residual confounding by indication remains possible, as patients initiated on BZDs (NewB or ContiB) may differ in illness severity, psychiatric distress, or palliative care needs compared with opioid‐only users. Requiring one‐year continuous opioid‐BZD co‐use before follow‐up may introduce survivor bias and limit generalizability. As with other observational studies, design cannot fully eliminate bias. This limitation should be considered in future research.

Despite these limitations, the study leveraged a large, multi‐institutional cohort of 418 549 patients with high treatment continuity, providing robust local evidence on opioid‐BZD co‐use in an Asian population. Importantly, this study addresses a critical gap in the literature, as data on opioid‐BZD co‐prescribing and its impact on mortality in Asian populations are scarce. Findings align with international guidelines, including CDC recommendations and the 2023 AGS Beers Criteria, offering clinically relevant insights to guide safer prescribing.

## 6 Conclusion

Patients receiving both opioids and BZDs had a higher risk of mortality than those using opioids alone, regardless of whether BZD use was past, new, or continuous. This risk was especially pronounced in older adults, individuals with cancer, and those with higher CCI scores. Concurrent use should be avoided when possible, and alternative treatments considered. While initiating BZDs requires caution, deprescribing in long‐term users should also be undertaken carefully, as abrupt discontinuation may result in harm.

## Nomenclature

Belelli et al. [43].

## Author Contributions

F.‐Y.S. and S.‐C.W.: conceptualization, methodology. F.‐Y.S.: data curation, writing – original draft preparation and review and editing. M.‐C.T.: formal analysis, discussion and revision of the manuscript. Y.‐Y.N. and S.‐C.W.: supervision and project administration.

## Funding

The authors have nothing to report.

## Ethics Statement

This study was conducted in accordance with the Declaration of Helsinki. Ethics approval was obtained from the Institutional Review Board (IRB) of Chang Gung Medical Foundation (Approval Number: 201801306B0).

## Consent

Patient consent was waived due to the retrospective and de‐identified nature of the study, as approved by the Institutional Review Board.

## Conflicts of Interest

The authors declare no conflicts of interest.

## Supporting information


**Data S1:** prp270215‐sup‐0001‐SupplementaryMaterial1.docx.

**Data S2:** prp270215‐sup‐0002‐SupplementaryMaterial2.pdf.

## Data Availability

Due to institutional regulations, the datasets generated and analyzed during the current study are not publicly available but may be obtained from the first author upon reasonable request.
