# Supplementary material for: The Risk Factors and Mortality Among Patients With Different Combination Patterns of Opioids and Benzodiazepines: A Retrospective Study
Source: Pharmacol Res Perspect. 2026 Jan 15;14(1):e70215. doi: 10.1002/prp2.70215 (PMC12808813; doi:10.1002/prp2.70215)
Supplement: Supplementary file 1 — Data S1: prp270215‐sup‐0001‐SupplementaryMaterial1.docx. [file PRP2-14-e70215-s002.docx]

**Supplementary material 1 Anatomical Therapeutic Chemical [ATC] code and Drug name**

| **Drug** | **ATC code** | **Drug Name** |
| --- | --- | --- |
| **Opioid Drugs Included in the Study Cohort:** | | |
|  | N02AA | Morphine HCl 10mg/tab |
|  | N02AA | MST(Morphine sulfate continus)30mg/f.c tab |
|  | N02AA | Hydromorphone PR 8mg/tab |
|  | N02AA | MXL(Morphine sulfate continus) 60mg/cap |
|  | N02AA | Morphine sulfate 15mg/tab |
|  | N02AA | OxyCodone Immediate Release 5mg/cap |
|  | N02AA | OxyCodone Controlled-Release 10mg/tab |
|  | N02AA | OxyCodone Controlled-Release 20mg/tab |
|  | N02AB | Meperidine(Pethidine) HCl 50mg/tab |
|  | N02AB | Fentanyl tts 25mcg/hr/patch |
|  | N02AB | Fentanyl tts 50mcg/hr/patch |
|  | N02AB | Fentanyl tts 12mcg/hr/patch |
|  | N02AB | Painkyl (Fentanyl buccal film) 200mcg/film |
|  | N02AB | Painkyl (Fentanyl buccal film) 600mcg/film |
|  | N02AB | Fentanyl tts 75mcg/hr/patch |
|  | N02AB | Fentora (Fentanyl buccal tablets) 100mcg/tab |
|  | N02AB | Fentora (Fentanyl buccal tablets) 200mcg/tab |
|  | N02AE | Buprenorphine 35 mcg/h,transdermal patch |
|  | N02AE | Buprenorphine 52.5 mcg/h,transdermal patch |
|  | N02AE | Buprenorphine 0.2mg/sublingual tab |
|  | N02AF | Butorphanol tartrate nasal spray 10mg/ml,2.5ml/bot |
|  | N02AX | Tramadol HCl 50mg/cap |
|  | N02AX | Ultracet tab(Tramadol 37.5mg+Acetaminophen 325mg) |
| **Opioid Drugs Excluded from the Study Cohort** | | |
|  | N07BC | Buprenorphine 8mg/Naloxone 2mg sublingual tab |
|  | N07BC | Buprenorphine 2mg/Naloxone 0.5mg sublingual tab |
|  | N07BC | Methadone HCl 10mg/mL,1L/bot(oral) |
|  | N07BC | Methadone HCl 5mg/mL,1L/bot(oral) |
| **Benzodiazepine (BZD) Drugs Included from the Study Cohort** | | |
|  | N03AE | Clonazepam 0.5mg/tab |
|  | N03AE | Clonazepam 2mg/tab |
|  | N05BA | Clobazam 10mg/tab |
|  | N05BA | Alprazolam 0.5mg/tab |
|  | N05BA | Alprazolam S.R 0.5mg/tab |
|  | N05BA | Chlordiazepoxide 5mg/tab |
|  | N05BA | Fludiazepam 0.25mg/tab |
|  | N05BA | Diazepam 2mg/tab |
|  | N05BA | Diazepam 5mg/tab |
|  | N05BA | Alprazolam 0.5mg/tab |
|  | N05BA | LORazepam 1mg/tab |
|  | N05CD | Estazolam 2mg/tab |
|  | N05CD | Flurazepam hcl 30mg/cap |
|  | N05CD | Triazolam 0.25mg/tab |
|  | N05CF | Zolpidem 10mg/f.c tab |
|  | N05CF | Zolpidem CR 6.25mg/f.c tab |
|  | N05CF | Zolpidem 10mg/f.c tab |
