# Supplementary material for: The Risk Factors and Mortality Among Patients With Different Combination Patterns of Opioids and Benzodiazepines: A Retrospective Study
Source: Pharmacol Res Perspect. 2026 Jan 15;14(1):e70215. doi: 10.1002/prp2.70215 (PMC12808813; doi:10.1002/prp2.70215)
Supplement: Supplementary file 2 — Data S2: prp270215‐sup‐0002‐SupplementaryMaterial2.pdf. [file PRP2-14-e70215-s001.pdf]

Supplementary Material 2 Primary Diagnoses

		Group			
ICD-9-CM	ICD-9-CM	PureO	ContiB	NewB	PastB
218.9	Leiomyoma of uterus, unspecified	3.0822%	1.1148%	1.2251%	1.8567%
715.36	Osteoarthrosis, localized, not specified whether primary or	1.8086%	2.8257%	2.3676%	3.0605%
155.0	Malignant neoplasm of liver, primary	1.6264%	2.5882%	2.5081%	2.3248%
738.4	Acquired spondylolisthesis	1.0354%	3.2716%	2.1527%	1.9459%
550.90	Inguinal hernia, without mention of obstruction or gangrene	1.5908%	0.7658%	0.5371%	1.6624%
617.1	Endometriosis of ovary	1.5455%	0.2375%	0.3326%	0.5223%
722.10	Displacement of lumbar intervertebral disc whthout myelop	1.0294%	1.8030%	1.6652%	0.9618%
470	Deviated nasal septum	1.4567%	0.2860%	0.2335%	0.8089%
540.9	Acute appendictis, without mention of peritonitis	1.4229%	0.2278%	0.3058%	0.7102%
592.1	Calculus of ureter	1.1483%	0.6786%	0.5020%	1.3376%
617.0	Endometriosis of uterus	1.2070%	0.4847%	0.4876%	0.8535%
724.02	Spinal stenosis, lumbar region	0.8569%	1.6915%	1.3057%	1.1847%
220	Benign neoplasm of ovary	1.2389%	0.3490%	0.3078%	0.5892%
218.1	Intramural leiomyoma of uterus	1.0923%	0.2860%	0.3450%	0.5350%
654.21	Previous cesarean delivery NOS, delivered,with or without	1.1704%	0.0388%	0.0558%	0.3280%
154.1	Malignant neoplasm of rectum	0.7547%	0.8482%	1.3305%	0.6083%
813.42	Other fractures of distal end of radius (alone), closed	0.8786%	0.4604%	0.5805%	0.8471%
600.0	Hypertrophy (benign) of prostate	0.6645%	0.7416%	0.9049%	1.1561%
153.3	Malignant neoplasm of sigmoid colon	0.6747%	0.6252%	0.9359%	0.6083%
473.9	Unspecified sinusitis (chronic)	0.8407%	0.2423%	0.2066%	0.6720%
574.10	Calculus of gallbladder with other cholecystitis, without me	0.7603%	0.3296%	0.2355%	0.7516%
756.12	Spondylolisthesis	0.4712%	1.1681%	0.9069%	0.6242%
652.21	Breech presentation without mention of version, delivered,	0.8273%	0.0339%	0.0393%	0.2325%
820.8	Fracture of unspecified part of neck of femur, closed	0.4550%	0.7949%	0.8181%	1.0637%
820.21	Fracture of intertrochanteric section of femur, closed	0.4578%	0.7706%	0.7995%	0.9682%
486	Pneumonia, organism unspecified	0.3464%	1.4056%	0.7169%	1.4076%
V58.1	Encounter for chemotherapy	0.2890%	2.3313%	0.7603%	1.0255%
455.2	Internal hemorrhoids with other complication	0.6890%	0.2472%	0.2190%	0.4618%
174.9	Malignant neoplasm of female breast, unspecified	0.3861%	0.9500%	0.9565%	0.6752%
574.00	Calculus of gallbladder with acute cholecystitis, without me	0.5864%	0.3393%	0.2913%	0.6210%
780.57	Other and unspecified sleep apnea	0.6405%	0.1648%	0.1797%	0.3503%
735.0	Hallux valgus (acquired)	0.6724%	0.1163%	0.0950%	0.2707%
574.20	Calculus of gallbladder without mention of cholecystitis an	0.5688%	0.2714%	0.2004%	0.6752%
996.4	Mechanical complication of internal orthopedic device, imp	0.4805%	0.5574%	0.5826%	0.4968%
182.0	Malignant neoplasm of corpus uteri, except isthmus	0.3727%	0.6446%	1.1094%	0.3599%
844.2	Sprains and strains of cruciate ligament of knee	0.6063%	0.1163%	0.3698%	0.1242%
565.1	Anal fistula	0.5596%	0.2472%	0.2830%	0.4172%

ICD-9-CM	ICD-9-CM	PureO	ContiB	NewB	PastB
V54.0	Aftercare involving removal of fracture plate or other inter	0.5799%	0.1357%	0.1797%	0.4299%
733.42	Aseptic necrosis of head and neck of femur	0.4624%	0.4508%	0.5062%	0.4873%
414.01	Coronary atherosclerosis of native coronary artery	0.3718%	0.7852%	0.5537%	0.7771%
162.3	Malignant neoplasm of upper lobe, bronchus or lung	0.2937%	1.0275%	0.7830%	0.5637%
633.1	Tubal pregnancy	0.5841%	0.0048%	0.0702%	0.1688%
540.0	Acute appendicitis, with generalized peritonitis	0.4957%	0.1454%	0.1673%	0.2834%
455.8	Unspecified hemorrhoids with other complication	0.4097%	0.3781%	0.3326%	0.4331%
577.0	Acute pancreatitis	0.3380%	0.4362%	0.5929%	0.4522%
193	Malignant neoplasm of thyroid gland	0.4328%	0.2811%	0.2665%	0.3854%
737.30	Scoliosis [and kyphoscoliosis], idiopathic	0.3108%	0.5913%	0.6673%	0.3694%
592.0	Calculus of kidney	0.4231%	0.2520%	0.2706%	0.3376%
382.9	Unspecified otitis media	0.4134%	0.2326%	0.2479%	0.3949%
722.0	Displacement of cervical intervertebral disc without myelo	0.2946%	0.6592%	0.4710%	0.5510%
802.4	Fracture of malar and maxillary bones, closed	0.4356%	0.1793%	0.3078%	0.1210%
185	Malignant neoplasm of prostate	0.3232%	0.3829%	0.4896%	0.4204%
715.35	Osteoarthrosis, localized, not specified whether primary or	0.3343%	0.5186%	0.3595%	0.4108%
821.01	Fracture of shaft of femur, closed	0.3885%	0.1357%	0.3967%	0.1720%
810.02	Fracture in shaft of clavicle, closed	0.3972%	0.1406%	0.3285%	0.1720%
512.8	Other spontaneous pneumothorax	0.4268%	0.1648%	0.1797%	0.1656%
038.9	Unspecified septicemia	0.2349%	0.6010%	0.3698%	0.7994%
188.9	Malignant neoplasm of bladder, part unspecified	0.2890%	0.3732%	0.4855%	0.3949%
145.0	Malignant neoplasm of cheek mucosa	0.1642%	0.4992%	1.1073%	0.1401%
183.0	Malignant neoplasm of ovary	0.0925%	0.7852%	1.2148%	0.2006%
733.13	Pathologic fracture of vertebrae	0.2349%	0.5138%	0.4752%	0.5287%
645.11	Prolonged pregnancy, delivered, with or without mention o	0.4347%	0.0145%	0.0393%	0.1051%
141.9	Malignant neoplasm of tongue, unspecified	0.1868%	0.4217%	0.9751%	0.0987%
153.6	Malignant neoplasm of ascending colon	0.2900%	0.2908%	0.3925%	0.3439%
189.0	Malignant neoplasm of kidney, except pelvis	0.2909%	0.3199%	0.3719%	0.3376%
218.0	Submucous leiomyoma of uterus	0.3801%	0.0969%	0.1074%	0.2229%
661.11	Secondary uterine inertia, delivered, with or without menti	0.4217%	0.0048%	0.0289%	0.1051%
210.2	Benign neoplasm of major salivary glands	0.3866%	0.1115%	0.0661%	0.1847%
599.0	Urinary tract infection, site not specified	0.1762%	0.7367%	0.3161%	0.8025%
V59.6	Donors of liver	0.3820%	0.0339%	0.1777%	0.0414%
733.82	Nonunion of fracture	0.3163%	0.2714%	0.1818%	0.3089%
151.9	Malignant neoplasm of stomach, unspecified	0.2377%	0.3974%	0.5062%	0.2389%
600.9	Hyperplasia of prostate, unspecified	0.2502%	0.3296%	0.3058%	0.4968%
162.9	Malignant neoplasm of bronchus and lung, unspecified	0.1632%	0.7125%	0.6157%	0.3471%
650	Normal delivery	0.3834%	0.0097%	0.0248%	0.1369%
823.00	Fracture of upper end of tibia alone, closed	0.2849%	0.1599%	0.3677%	0.1815%

ICD-9-CM	ICD-9-CM	PureO	ContiB	NewB	PastB
162.5	Malignant neoplasm of lower lobe, bronchus or lung	0.1827%	0.6155%	0.5392%	0.3057%
518.81	Acute respiratory failure	0.1646%	0.5816%	0.4566%	0.5669%
635.92	Legally induced abortion, without mention of complication	0.3760%	0.0048%	0.0289%	0.1178%
241.1	Nontoxic multinodular goiter	0.3186%	0.1212%	0.1178%	0.2962%
625.6	Stress incontinence, female	0.2696%	0.1987%	0.1756%	0.4490%
618.3	Uterovaginal prolapse, complete	0.2900%	0.1745%	0.1777%	0.2834%
540.1	Acute appendictis, with peritoneal abscess	0.3237%	0.0872%	0.1198%	0.1497%
651.01	Twin pregnancy, delivered, with or without mention of ant	0.3579%	0.0145%	0.0083%	0.1274%
174.4	Malignant neoplasm of female breast, upper-outer quadrant	0.1970%	0.3393%	0.4690%	0.3057%
669.71	Ceasrean delivery, without mention of indication, delivered	0.3464%	0.0242%	0.0186%	0.1497%
822.0	Fracture of patella, closed	0.2497%	0.1987%	0.2314%	0.2166%
805.4	Fracture of lumbar vertebra, closed	0.1706%	0.3587%	0.4421%	0.3057%
682.6	Other cellulitis and abscess, leg, except foot	0.1910%	0.3732%	0.2789%	0.3567%
618.4	Uterovaginal prolapse, unspecified	0.2465%	0.1260%	0.1611%	0.3185%
410.71	Acute subendocardial infarction, initial episode of care	0.1554%	0.4314%	0.3781%	0.4013%
656.81	Other specified fetal and placental problems affecting man	0.3191%	0.0194%	0.0103%	0.0828%
174.8	Malignant neoplasm of other specified sites of female breas	0.1734%	0.2666%	0.4173%	0.2675%
575.0	Acute cholecystitis	0.2321%	0.1745%	0.1611%	0.3057%
180.9	Malignant neoplasm of cervix uteri, unspecified	0.1586%	0.3829%	0.4277%	0.1688%
217	Benign neoplasm of breast	0.2724%	0.0582%	0.0413%	0.1656%
717.83	Old disruption of anterior cruciate ligament	0.2673%	0.0436%	0.1136%	0.0924%
474.00	Chronic tonsillitis	0.2691%	0.0582%	0.0393%	0.1783%
661.01	Primary uterine inertia, delivered, with or without mention	0.2960%	0.0000%	0.0083%	0.0605%
403.91	Unspecified hypertensive renal disease with renal failure	0.1119%	0.6495%	0.2727%	0.4427%
886.0	Traumatic amputation of other fingers (complete) (partial)	0.1970%	0.1599%	0.3615%	0.0255%
813.44	Fracture of radius with ulna, lower end, closed	0.2150%	0.1066%	0.1302%	0.1815%
218.2	Subserous leiomyoma of uterus	0.2372%	0.0533%	0.0661%	0.1210%
566	Abscess of anal and rectal regions	0.1989%	0.1115%	0.1673%	0.1656%
560.81	Intestinal or peritoneal adhesions with obstruction(postope	0.1804%	0.1939%	0.1715%	0.2038%
641.01	Placenta previa without hemorrhage, delivered, with or wit	0.2539%	0.0097%	0.0145%	0.0510%
434.91	Unspecified cerebral artery occlusion with cerebral infarcti	0.1096%	0.4508%	0.2438%	0.3854%
155.1	Malignant neoplasm of intrahepatic bile ducts	0.1327%	0.3054%	0.3595%	0.1433%
571.5	Cirrhosis of liver without mention of alcohol	0.1286%	0.2908%	0.2851%	0.2866%
153.2	Malignant neoplasm of descending colon	0.1669%	0.1842%	0.2335%	0.1561%
444.22	Arterial embolism and thrombosis of lower extremity	0.1383%	0.2811%	0.2458%	0.2643%
361.00	Retinal detachment with retinal defect, unspecified	0.2266%	0.0533%	0.0620%	0.0860%
721.3	Lumbosacral spondylosis without myelopathy	0.1281%	0.2423%	0.3533%	0.1879%
V50.1	Elective surgery for other plastic surgery for unacceptable	0.2201%	0.0533%	0.0826%	0.0892%
620.2	Other and unspecified ovarian cyst	0.2178%	0.0775%	0.0806%	0.0828%

ICD-9-CM	ICD-9-CM	PureO	ContiB	NewB	PastB
233.0	Carcinoma in situ of breast	0.1794%	0.1357%	0.1302%	0.1783%
614.2	Salpingitis and oophoritis, not specified as acute, subacute	0.1924%	0.1066%	0.1240%	0.1019%
198.5	Secondary malignant neoplasm of bone and bone marrow	0.0953%	0.3344%	0.4318%	0.1369%
214.1	Lipoma of other skin and subcutaneous tissue	0.1993%	0.0485%	0.0764%	0.1338%
576.1	Cholangitis	0.1415%	0.2617%	0.1508%	0.2452%
239.0	Neoplasm of unspecified nature of digestive system	0.1619%	0.1454%	0.1364%	0.1847%
824.4	Fracture of bimalleolar of ankle, closed	0.1674%	0.1163%	0.1446%	0.1497%
241.0	Nontoxic uninodular goiter	0.1891%	0.0533%	0.0640%	0.1561%
510.9	Empyema, without mention of fistula	0.1369%	0.2133%	0.2107%	0.1401%
428.0	Congestive heart failure	0.0888%	0.4314%	0.2273%	0.2898%
824.2	Fracture of lateral malleolus of ankle, closed	0.1827%	0.0533%	0.0888%	0.1019%
524.4	Malocclusion, unspecified	0.2044%	0.0145%	0.0578%	0.0255%
473.8	Other chronic sinusitis	0.1651%	0.0969%	0.0475%	0.2580%
153.1	Malignant neoplasm of transverse colon	0.1378%	0.1454%	0.2107%	0.1274%
653.41	Fetopelvic disproportion, delivered, with or without mentio	0.2044%	0.0048%	0.0021%	0.0605%
641.11	Hemorrhage from placenta previa, delivered, with or witho	0.2063%	0.0048%	0.0103%	0.0318%
927.3	Crushing injury of finger(s)	0.1734%	0.0291%	0.1343%	0.0478%
723.0	Spinal stenosis in cervical region	0.1119%	0.3005%	0.2169%	0.1242%
658.11	Premature rupture of membranes, delivered, with or withou	0.1924%	0.0000%	0.0103%	0.0732%
728.86	Necrotizing fasciitis	0.1004%	0.1793%	0.3016%	0.1210%
812.41	Supracondylar fracture of humerus, closed	0.1776%	0.0145%	0.0434%	0.0510%
189.1	Malignant neoplasm of renal pelvis	0.1110%	0.1842%	0.1818%	0.1752%
805.2	Fracture of dorsal (thoracic) vertebra, closed	0.1013%	0.1793%	0.2355%	0.1561%
189.2	Malignant neoplasm of ureter	0.1017%	0.2181%	0.2045%	0.1752%
823.22	Fracture of shaft of tibia and fibula, closed	0.1494%	0.0485%	0.1426%	0.0446%
727.61	Complete rupture of rotator cuff	0.0846%	0.2811%	0.2190%	0.2197%
233.1	Carcinoma in situ of cervix uteri	0.1410%	0.1163%	0.0847%	0.1465%
722.71	Cervical intervertebral disc disorder with myelopathy	0.1068%	0.2036%	0.1901%	0.1465%
618.0	Prolapse of vaginal walls, without mention of uterine prola	0.1239%	0.1115%	0.1157%	0.1975%
562.11	Diverticulitis of colon (without mention of hemorrhage)	0.1221%	0.1309%	0.1384%	0.1624%
550.92	Inguinal hernia, without mention of obstruction or gangren	0.1452%	0.0485%	0.0930%	0.1210%
361.9	Unspecified retinal detachment	0.1609%	0.0679%	0.0413%	0.0796%
454.9	Varicose veins of lower extremities without mention of ulc	0.1466%	0.0824%	0.0351%	0.1720%
174.2	Malignant neoplasm of female breast, upper-inner quadrant	0.0948%	0.1793%	0.2520%	0.1178%
531.40	Gastric ulcer, chronic or unspecified with hemorrhage, with	0.0939%	0.2375%	0.1467%	0.2357%
455.0	Internal hemorrhoids without mention of complication	0.1225%	0.1066%	0.1529%	0.1115%
594.1	Other calculus in bladder	0.1364%	0.0969%	0.0723%	0.1433%
682.0	Other cellulitis and abscess, face	0.1313%	0.1066%	0.1095%	0.0987%
250.80	Diabetes with other specified manifestations, Type II [non-	0.0897%	0.2278%	0.1901%	0.1815%

ICD-9-CM	ICD-9-CM	PureO	ContiB	NewB	PastB
812.21	Fracture of shaft of humerus, closed	0.1258%	0.0679%	0.1508%	0.0828%
143.1	Malignant neoplasm of lower gum	0.0402%	0.2763%	0.4524%	0.0605%
996.66	Infection and inflammatory reaction due to internal joint pr	0.0916%	0.0824%	0.2892%	0.0796%
154.0	Malignant neoplasm of rectosigmoid junction	0.1124%	0.1018%	0.1839%	0.0732%
472.0	Chronic rhinitis	0.1461%	0.0582%	0.0289%	0.1019%
455.6	Unspecified hemorrhoids without mention of complication	0.0962%	0.2036%	0.1570%	0.1497%
620.1	Corpus luteum cyst or hematoma	0.1494%	0.0242%	0.0413%	0.0732%
823.20	Fracture of shaft of tibia alone, closed	0.1313%	0.0533%	0.1178%	0.0510%
610.1	Diffuse cystic mastopathy	0.1281%	0.0969%	0.0372%	0.1656%
197.7	Secondary malignant neoplasm of liver	0.0856%	0.1939%	0.1901%	0.1561%
628.2	Infertility, female, of tubal origin	0.1526%	0.0291%	0.0124%	0.0732%
733.81	Malunion of fracture	0.1350%	0.0485%	0.0744%	0.0796%
590.10	Acute pyelonephritis without lesion of renal medullary nec	0.1004%	0.1648%	0.0930%	0.2102%
157.0	Malignant neoplasm of head of pancreas	0.0735%	0.1551%	0.2727%	0.1178%
840.4	Sprains and strains of rotator cuff (capsule)	0.1031%	0.1648%	0.1384%	0.1115%
588.8	Other specified disorders resulting from impaired renal fun	0.1022%	0.0872%	0.0516%	0.2994%
148.9	Malignant neoplasm of hypopharynx, unspecified	0.0620%	0.1987%	0.3306%	0.0637%
532.50	Duodenal ulcer, chronic or unspecified with perforation, wi	0.1147%	0.0485%	0.1467%	0.0796%
642.51	Severe pre-eclampsia, delivered, with or without mention o	0.1521%	0.0145%	0.0289%	0.0159%
812.00	Fracture in unspecified part of upper end of humerus, close	0.0967%	0.1115%	0.1591%	0.1306%
550.10	Inguinal hernia, with obstruction, without mention of gangr	0.1258%	0.0679%	0.0661%	0.1019%
441.01	Dissection of aorta, thoracic	0.0846%	0.0824%	0.2665%	0.0669%
652.61	Multiple gestation with malpresentation of one fetus or mo	0.1521%	0.0000%	0.0062%	0.0510%
721.1	Cervical spondylosis with myelopathy	0.0805%	0.2326%	0.1735%	0.1242%
574.50	Calculus of bile duct without mention of cholecystitis with	0.1059%	0.0969%	0.0971%	0.1561%
860.0	Traumatic pneumothorax without mention of open wound i	0.1147%	0.0533%	0.1405%	0.0478%
211.3	Benign neoplasm of colon	0.1128%	0.0727%	0.0826%	0.1369%
531.50	Gastric ulcer, chronic or unspecified with perforation, with	0.1161%	0.0775%	0.1033%	0.0764%
721.0	Cervical spondylosis without myelopathy	0.0740%	0.2423%	0.1426%	0.1879%
927.20	Crushing injury of hand(s)	0.1013%	0.0679%	0.1921%	0.0255%
571.2	Alcoholic cirrhosis of liver	0.0610%	0.2860%	0.1549%	0.2166%
151.2	Malignant neoplasm of pyloric antrum of stomach	0.0999%	0.0872%	0.1405%	0.0987%
197.0	Secondary malignant neoplasm of lung	0.0735%	0.2133%	0.1529%	0.1752%
682.7	Other cellulitis and abscess, foot, except toes	0.0846%	0.1745%	0.1240%	0.1656%
424.0	Mitral valve disorders	0.0846%	0.1793%	0.1116%	0.1815%
823.32	Fracture of shaft of tibia and fibula, open	0.0976%	0.0824%	0.1839%	0.0414%
998.59	Other postoperative infection	0.0962%	0.1503%	0.1240%	0.0955%
197.2	Secondary malignant neoplasm of pleura	0.0467%	0.2811%	0.1921%	0.2389%
813.01	Fracture of olecranon process of ulna, closed	0.1198%	0.0533%	0.0702%	0.0701%

ICD-9-CM	ICD-9-CM	PureO	ContiB	NewB	PastB
709.2	Scar conditions and fibrosis of skin	0.1188%	0.0339%	0.0992%	0.0350%
831.04	Closed dislocation of acromioclavicular (joint)	0.1212%	0.0436%	0.0744%	0.0446%
578.9	Hemorrhage of gastrointestinal tract, unspecified	0.0777%	0.1793%	0.1074%	0.2038%
145.9	Malignant neoplasm of mouth, unspecified	0.0481%	0.1406%	0.3512%	0.0573%
202.80	Other lymphomas, unspecified, extranodal solid organ sites	0.0717%	0.2036%	0.1735%	0.1242%
662.21	Prolonged second stage, delivered, with or without mentior	0.1420%	0.0000%	0.0062%	0.0287%
572.0	Abscess of liver	0.0906%	0.1212%	0.1364%	0.1019%
860.4	Pneumohemothorax without mention of open wound into th	0.0994%	0.0582%	0.1529%	0.0541%
825.0	Fracture of calcaneus, closed	0.1082%	0.0388%	0.1198%	0.0446%
V53.31	Fitting and adjustment of cardiac pacemaker	0.0860%	0.0872%	0.0310%	0.2962%
722.52	Degeneration of lumbar or lumbosacral intervertebral disc	0.0814%	0.1163%	0.1384%	0.1433%
715.25	Osteoarthrosis, localized, secondary, pelvic region and thig	0.1045%	0.0872%	0.0909%	0.0764%
808.0	Fracture of acetabulum, closed	0.0943%	0.0436%	0.1880%	0.0223%
227.0	Benign neoplasm of suprarenal gland	0.0976%	0.0824%	0.0806%	0.1401%
614.1	Chronic salpingitis and oophoritis	0.1225%	0.0242%	0.0207%	0.0955%
620.8	Other noninflammatory disorders of ovary, fallopian tube, a	0.1272%	0.0145%	0.0227%	0.0573%
584.9	Acute renal failure, unspecified	0.0615%	0.2084%	0.1364%	0.2006%
410.91	Acute myocardial infarction of unspecified site, initial episo	0.0777%	0.1115%	0.1632%	0.1115%
V57.89	Other specified rehabilitation procedures	0.0240%	0.6398%	0.1343%	0.1752%
278.01	Morbid obesity	0.1184%	0.0242%	0.0413%	0.0732%
618.1	Uterine prolapse without mention of vaginal wall prolapse	0.0943%	0.0775%	0.0578%	0.1688%
824.8	Unspecified fracture of ankle, closed	0.1022%	0.0582%	0.1074%	0.0478%
205.00	Acute myeloid leukemia, without mention of remission	0.0536%	0.3054%	0.1942%	0.0796%
860.2	Traumatic hemothorax without mention of open wound into	0.0902%	0.0679%	0.1405%	0.0637%
431	Intracerebral hemorrhage	0.0758%	0.1212%	0.1653%	0.0860%
550.91	Inguinal hernia, without mention of obstruction or gangrene	0.1022%	0.0533%	0.0496%	0.1242%
518.89	Other diseases of lung, not elsewhere classified	0.0805%	0.1599%	0.1033%	0.1115%
196.0	Secondary and unspecified malignant neoplasm of lymph n	0.0731%	0.1454%	0.1508%	0.0796%
749.21	Complete cleft palate with cleft lip, unilateral	0.1309%	0.0000%	0.0041%	0.0000%
236.2	Neoplasm of uncertain behavior of ovary	0.1008%	0.0339%	0.0744%	0.0732%
153.4	Malignant neoplasm of cecum	0.0856%	0.1115%	0.1095%	0.0732%
810.03	Fracture in acromial end of clavicle, closed	0.0976%	0.0630%	0.0868%	0.0541%
491.21	Obstructive chronic bronchitis with acute exacerbation	0.0379%	0.2811%	0.1116%	0.2834%
656.31	Fetal distress affecting management of mother, delivered, v	0.1207%	0.0145%	0.0103%	0.0318%
720.9	Unspecified inflammatory spondylopathy	0.0518%	0.1793%	0.2273%	0.0605%
198.3	Secondary malignant neoplasm of brain and spinal cord	0.0416%	0.3587%	0.1446%	0.1369%
441.03	Dissection of aorta, thoracoabdominal	0.0647%	0.1066%	0.1839%	0.0796%
241.9	Unspecified nontoxic nodular goiter	0.0990%	0.0775%	0.0289%	0.1019%
813.41	Colles' fracture, closed	0.0763%	0.1018%	0.1054%	0.1210%

ICD-9-CM	ICD-9-CM	PureO	ContiB	NewB	PastB
157.9	Malignant neoplasm of pancreas, part unspecified	0.0606%	0.1163%	0.2004%	0.0732%
812.01	Fracture of surgical neck of humerus, closed	0.0828%	0.1163%	0.1054%	0.0637%
574.90	Calculus of gallbladder and bile duct without cholecystitis	0.0879%	0.0339%	0.0661%	0.1369%
430	Subarachnoid hemorrhage	0.0749%	0.1115%	0.1405%	0.0573%
507.0	Pneumonitis due to inhalation of food or vomitus	0.0458%	0.1696%	0.1012%	0.2803%
141.2	Malignant neoplasm of tip and lateral border of tongue	0.0536%	0.1745%	0.1818%	0.0573%
928.20	Crushing injury of foot	0.0758%	0.0291%	0.1653%	0.0223%
424.1	Aortic valve disorders	0.0657%	0.1551%	0.0826%	0.1369%
274.0	Gouty arthropathy	0.0832%	0.0582%	0.0847%	0.0732%
825.25	Fracture of metatarsal bone(s), closed	0.0902%	0.0485%	0.0640%	0.0573%
659.71	Abnormality in fetal heart rate or rhythm, delivered, with o	0.1105%	0.0048%	0.0062%	0.0318%
475	Peritonsillar abscess	0.0999%	0.0291%	0.0393%	0.0382%
153.0	Malignant neoplasm of hepatic flexure colon	0.0717%	0.0921%	0.1157%	0.0701%
456.4	Scrotal varices	0.1027%	0.0194%	0.0227%	0.0446%
807.03	Fracture of three ribs, closed	0.0735%	0.0533%	0.1054%	0.0924%
840.8	Sprains and strains of other specified sites of shoulder and	0.0888%	0.0727%	0.0496%	0.0573%
813.23	Fracture of shaft of radius with ulna, closed	0.1027%	0.0145%	0.0331%	0.0127%
427.0	Paroxysmal supraventricular tachycardia	0.0869%	0.0727%	0.0269%	0.0924%
239.5	Neoplasm of unspecified nature of other genitourinary orga	0.0828%	0.0485%	0.0516%	0.0955%
724.6	Disorders of sacrum	0.0592%	0.0872%	0.1240%	0.1083%
569.83	Perforation of intestine	0.0726%	0.0630%	0.0909%	0.0828%
354.0	Carpal tunnel syndrome	0.0671%	0.0727%	0.0909%	0.1146%
250.70	Diabetes with peripheral circulatory disorders, Type II [nor	0.0504%	0.1163%	0.1467%	0.1146%
147.9	Malignant neoplasm of nasopharynx, unspecified	0.0398%	0.1939%	0.1632%	0.1083%
717.84	Old disruption of posterior cruciate ligament	0.0814%	0.0291%	0.0826%	0.0510%
614.6	Pelvic peritoneal adhesions, female（postoperative）(posti	0.0911%	0.0291%	0.0227%	0.0764%
885.0	Traumatic amputation of thumb (complete) (partial) withou	0.0717%	0.0485%	0.1384%	0.0127%
852.06	Subarachnoid hemorrhage following injury without mentio	0.0671%	0.0630%	0.1364%	0.0382%
845.09	Sprains and strains of other specified site of ankle	0.0902%	0.0339%	0.0455%	0.0318%
648.91	Other current conditions classifiable elsewhere in the moth	0.0976%	0.0048%	0.0103%	0.0414%
150.4	Malignant neoplasm of middle third of esophagus	0.0296%	0.2569%	0.2025%	0.0478%
821.23	Supracondylar fracture of femur, closed	0.0675%	0.0436%	0.1033%	0.0764%
532.40	Duodenal ulcer, chronic or unspecified with hemorrhage, w	0.0606%	0.0969%	0.0930%	0.0987%
151.8	Malignant neoplasm of other specified sites of stomach	0.0606%	0.0824%	0.1240%	0.0573%
813.21	Fracture of shaft of radius(alone), closed	0.0828%	0.0145%	0.0537%	0.0510%
575.6	Cholesterolosis of gallbladder	0.0879%	0.0194%	0.0186%	0.0669%
223.0	Benign neoplasm of kidney, except pelvis	0.0731%	0.0630%	0.0702%	0.0541%
197.6	Malignant neoplasm of retroperitoneum and peritoneum	0.0379%	0.1939%	0.1529%	0.0828%
511.9	Pleurisy, unspecified pleural effusion	0.0439%	0.1842%	0.0909%	0.1401%

ICD-9-CM	ICD-9-CM	PureO	ContiB	NewB	PastB
226	Benign neoplasm of thyroid glands	0.0851%	0.0291%	0.0227%	0.0637%
864.05	Unspecified laceration to liver, without mention of open wo	0.0782%	0.0145%	0.0826%	0.0223%
738.19	Other specified acquired deformity of head	0.0569%	0.1503%	0.0661%	0.1051%
593.3	Stricture or kinking of ureter	0.0657%	0.0388%	0.0764%	0.0955%
513.0	Abscess of lung	0.0597%	0.0969%	0.0950%	0.0701%
410.11	Acute myocardial infarction of other anterior wall, initial e	0.0546%	0.0872%	0.1219%	0.0701%
038.42	Septicemia due to Escherichia coli〔E. Coli〕	0.0449%	0.1648%	0.0661%	0.1688%
558.9	Other and unspecified noninfectious gastroenteritis and coli	0.0721%	0.0436%	0.0537%	0.0732%
840.6	Sprains and strains of supraspinatus (muscle) (tendon)	0.0610%	0.0339%	0.0764%	0.1146%
663.31	Other and unspecified cord entanglement, without mention	0.0925%	0.0000%	0.0041%	0.0318%
852.21	Subdural hemorrhage following injury without mention of	0.0541%	0.1115%	0.0930%	0.0828%
533.50	Peptic ulcer, site unspecified, chronic or unspecified with p	0.0661%	0.0339%	0.0682%	0.0860%
567.2	Other suppurative peritonitis	0.0504%	0.0485%	0.1157%	0.1083%
531.90	Gastric ulcer, unspecified as acute or chronic, without men	0.0509%	0.1212%	0.0888%	0.0987%
823.30	Fracture of shaft of tibia alone, open	0.0735%	0.0097%	0.0826%	0.0223%
802.26	Fracture of mandible, closed, symphysis of body	0.0763%	0.0436%	0.0558%	0.0191%
820.22	Fracture of subtrochanteric section of femur, closed	0.0587%	0.0775%	0.0930%	0.0541%
644.21	Early onset of delivery, delivered, with or without mention	0.0897%	0.0000%	0.0062%	0.0255%
250.82	Diabetes with other specified manifestations, Type II [non-	0.0486%	0.1212%	0.0930%	0.0955%
145.6	Malignant neoplasm of retromolar area	0.0250%	0.0824%	0.2562%	0.0318%
146.0	Malignant neoplasm of tonsil	0.0453%	0.0582%	0.1715%	0.0382%
174.5	Malignant neoplasm of female breast, lower-outer quadrant	0.0550%	0.0727%	0.1095%	0.0573%
706.2	Sebaceous cyst	0.0846%	0.0048%	0.0124%	0.0446%
660.01	Obstruction caused by malposition of fetus at onset of labo	0.0874%	0.0097%	0.0041%	0.0350%
150.5	Malignant neoplasm of lower third of esophagus	0.0324%	0.1648%	0.1549%	0.0796%
802.6	Fracture of orbital floor (blow-out), closed	0.0772%	0.0291%	0.0413%	0.0318%
656.41	Intrauterine death affecting management of mother, deliver	0.0879%	0.0097%	0.0124%	0.0127%
441.4	Abdominal aneurysm without mention of rupture	0.0610%	0.1018%	0.0640%	0.0573%
242.00	Toxic diffuse goiter without mention of thyrotoxic crisis or	0.0726%	0.0533%	0.0165%	0.0828%
891.0	Open wound of knee, leg (except thigh) and ankle, without	0.0708%	0.0145%	0.0702%	0.0350%
528.9	Other and unspecified diseases of the oral soft tissues	0.0647%	0.0533%	0.0661%	0.0573%
621.3	Endometrial cystic hyperplasia	0.0680%	0.0436%	0.0310%	0.0955%
810.00	Fracture in unspecified part of clavicle, closed	0.0666%	0.0485%	0.0640%	0.0446%
785.6	Enlargement of lymph nodes	0.0731%	0.0291%	0.0331%	0.0605%
214.8	Lipoma of other specified sites	0.0768%	0.0145%	0.0289%	0.0510%
658.21	Delayed delivery after spontaneous or unspecified rupture c	0.0888%	0.0048%	0.0021%	0.0127%
824.6	Fracture of trimalleolar of ankle, closed	0.0643%	0.0291%	0.0702%	0.0573%
642.41	Mild or unspecified pre-eclampsia, delivered, with or witho	0.0832%	0.0097%	0.0145%	0.0255%
812.03	Fracture of greater tuberosity of humerus, closed	0.0573%	0.0679%	0.0806%	0.0573%

ICD-9-CM	ICD-9-CM	PureO	ContiB	NewB	PastB
836.1	Tear of lateral cartilage or meniscus of knee, current	0.0754%	0.0291%	0.0372%	0.0255%
836.0	Tear of medial cartilage or meniscus of knee, current	0.0708%	0.0436%	0.0393%	0.0446%
354.2	Lesion of ulnar nerve	0.0657%	0.0291%	0.0516%	0.0669%
865.09	Other injury to spleen, without mention of open wound int	0.0615%	0.0291%	0.0930%	0.0287%
820.03	Fracture of base of neck of femur, closed	0.0467%	0.0582%	0.0971%	0.1019%
603.9	Hydrocele, unspecified	0.0740%	0.0242%	0.0207%	0.0541%
117.9	Other and unspecified mycoses	0.0647%	0.0291%	0.0248%	0.1083%
162.4	Malignant neoplasm of middle lobe, bronchus or lung	0.0398%	0.1357%	0.1219%	0.0541%
591	Hydronephrosis	0.0583%	0.0679%	0.0372%	0.0987%
038.49	Septicemia due to other Gram-negative organisms	0.0388%	0.1260%	0.0868%	0.1178%
V58.0	Encounter for radiotherapy	0.0176%	0.3587%	0.1178%	0.0573%
821.11	Fracture of shaft of femur, open	0.0587%	0.0291%	0.1054%	0.0096%
585	Chronic renal failure	0.0379%	0.1309%	0.0868%	0.1115%
824.0	Fracture of medial malleolus of ankle, closed	0.0680%	0.0145%	0.0413%	0.0478%
726.10	Disorders of bursae and tendons in shoulder region, unspec	0.0504%	0.0872%	0.0496%	0.1083%
682.1	Other cellulitis and abscess, neck	0.0629%	0.0242%	0.0620%	0.0446%
386.00	Meniere's disease, unspecified	0.0282%	0.1066%	0.1281%	0.1274%
756.11	Spondylolysis, lumbosacral region	0.0513%	0.0436%	0.0764%	0.0860%
820.09	Other fracture of neck of femur, closed	0.0532%	0.0630%	0.0661%	0.0732%
852.01	Subarachnoid hemorrhage following injury without mentio	0.0518%	0.0436%	0.1012%	0.0414%
202.88	Other lymphomas, lymph nodes of multiple sites	0.0287%	0.1309%	0.1384%	0.0860%
780.50	Sleep disturbances, unspecified	0.0777%	0.0145%	0.0103%	0.0191%
560.9	Unspecified intestinal obstruction	0.0555%	0.0727%	0.0434%	0.0828%
611.8	Other specified disorders of breast	0.0462%	0.0582%	0.0909%	0.0828%
410.41	Acute myocardial infarction of other inferior wall, initial e	0.0439%	0.0679%	0.1178%	0.0510%
427.81	Sinoatrial node dysfunction	0.0370%	0.1066%	0.0393%	0.1943%
759.2	Anomalies of other endocrine glands	0.0763%	0.0048%	0.0124%	0.0255%
151.4	Malignant neoplasm of body of stomach	0.0462%	0.0533%	0.0888%	0.0796%
852.26	Subdural hemorrhage following injury without mention of	0.0444%	0.0630%	0.1240%	0.0287%
807.04	Fracture of four ribs, closed	0.0532%	0.0388%	0.0826%	0.0478%
385.30	Cholesteatoma, unspecified	0.0703%	0.0291%	0.0310%	0.0159%
455.5	External hemorrhoids with other complication	0.0661%	0.0242%	0.0227%	0.0573%
822.1	Fracture of patella, open	0.0592%	0.0097%	0.0847%	0.0159%
812.09	Fracture in other part of upper end of humerus, closed	0.0532%	0.0436%	0.0682%	0.0573%
754.81	Pectus excavatum	0.0786%	0.0000%	0.0083%	0.0032%
709.8	Other specified disorders of skin	0.0573%	0.0582%	0.0537%	0.0414%
572.2	Hepatic coma	0.0333%	0.0969%	0.0413%	0.1975%
478.5	Other diseases of vocal cords	0.0578%	0.0436%	0.0269%	0.0860%
541	Appendicitis, unqualified	0.0689%	0.0048%	0.0227%	0.0382%

ICD-9-CM	ICD-9-CM	PureO	ContiB	NewB	PastB
567.9	Unspecified peritonitis	0.0467%	0.0630%	0.0682%	0.0796%
141.0	Malignant neoplasm of base of tongue	0.0291%	0.0921%	0.1570%	0.0446%
730.16	Chronic osteomyelitis, lower leg	0.0555%	0.0291%	0.0578%	0.0541%
744.46	Preauricular sinus or fistula	0.0777%	0.0000%	0.0000%	0.0096%
633.8	Other ectopic pregnancy	0.0698%	0.0097%	0.0145%	0.0350%
242.90	Thyrotoxicosis without mention of goiter or other cause, w	0.0592%	0.0339%	0.0165%	0.0860%
928.10	Crushing injury of lower leg	0.0444%	0.0436%	0.1178%	0.0223%
156.2	Malignant neoplasm of Ampulla of Vater	0.0379%	0.0679%	0.1219%	0.0446%
802.0	Fracture of nasal bones, closed	0.0643%	0.0145%	0.0351%	0.0287%
745.4	Ventricular septal defect	0.0735%	0.0097%	0.0021%	0.0127%
682.2	Other cellulitis and abscess, trunk	0.0462%	0.0436%	0.0682%	0.0764%
711.06	Pyogenic arthritis, lower leg	0.0435%	0.0727%	0.0868%	0.0478%
557.9	Unspecified vascular insufficiency of intestine	0.0467%	0.0485%	0.0620%	0.0796%
240.9	Goiter, unspecified	0.0573%	0.0145%	0.0289%	0.0796%
573.8	Other specified disorders of liver	0.0518%	0.0582%	0.0475%	0.0573%
203.00	Multiple myeloma, without mention of remission	0.0227%	0.1696%	0.1446%	0.0350%
786.09	Other dyspnea and respiratory abnormalities	0.0597%	0.0485%	0.0186%	0.0510%
648.21	Anemia conditions in the mother classifiable elsewhere, bu	0.0717%	0.0000%	0.0041%	0.0223%
717.3	Other and unspecified derangement of medial meniscus	0.0467%	0.0533%	0.0393%	0.1051%
941.30	Burn of face and head, unspecified site, full-thickness skin	0.0560%	0.0291%	0.0661%	0.0127%
617.3	Endometriosis of pelvic peritoneum	0.0583%	0.0242%	0.0269%	0.0605%
473.0	Chronic sinusitis of maxillary	0.0541%	0.0388%	0.0351%	0.0669%
707.0	Decubitus ulcer	0.0361%	0.0872%	0.0537%	0.1274%
150.9	Malignant neoplasm of esophagus, unspecified	0.0314%	0.0921%	0.1219%	0.0510%
891.1	Open wound of knee, leg (except thigh) and ankle, complic	0.0523%	0.0339%	0.0558%	0.0446%
801.26	Fracture of base of skull, closed with subarachnoid, subdur	0.0509%	0.0388%	0.0764%	0.0159%
560.1	Paralytic ileus	0.0407%	0.0872%	0.0413%	0.1083%
813.05	Head of radius fractures, closed	0.0564%	0.0339%	0.0413%	0.0287%
228.09	Hemangioma, other sites	0.0573%	0.0339%	0.0351%	0.0318%
252.0	Hyperparathyroidism	0.0472%	0.0582%	0.0207%	0.1083%
140.1	Malignant neoplasm of lower lip, vermilion border	0.0277%	0.0775%	0.1384%	0.0478%
421.0	Acute and subacute bacterial endocarditis	0.0347%	0.1357%	0.0868%	0.0350%
941.39	Burn of multiple sites (except with eye) of face, head and n	0.0578%	0.0048%	0.0578%	0.0000%
632	Missed abortion	0.0647%	0.0145%	0.0124%	0.0127%
816.01	Fracture of middle or proximal phalanx or phalanges, close	0.0601%	0.0194%	0.0289%	0.0127%
288.0	Agranulocytosis	0.0236%	0.1115%	0.1074%	0.0764%
826.1	Fracture of one or more phalanges of foot, open	0.0523%	0.0145%	0.0558%	0.0191%
808.2	Fracture of pubis, closed	0.0379%	0.0388%	0.0950%	0.0382%
714.0	Rheumatoid arthritis	0.0412%	0.0921%	0.0537%	0.0446%

ICD-9-CM	ICD-9-CM	PureO	ContiB	NewB	PastB
161.0	Malignant neoplasm of glottis	0.0481%	0.0291%	0.0599%	0.0287%
150.8	Malignant neoplasm of other specified part of esophagus	0.0166%	0.2036%	0.1260%	0.0223%
599.7	Hematuria	0.0407%	0.0679%	0.0434%	0.0701%
574.60	Calculus of gallbladder and bile duct with acute cholecystit	0.0499%	0.0145%	0.0289%	0.0637%
618.2	Uterovaginal prolapse, incomplete	0.0453%	0.0339%	0.0475%	0.0541%
427.31	Atrial fibrillation	0.0324%	0.0872%	0.0578%	0.0924%
053.9	Herpes zoster without mention of complication	0.0328%	0.0969%	0.0599%	0.0796%
658.41	Infection of amniotic cavity, delivered, with or without me	0.0624%	0.0000%	0.0021%	0.0255%
225.3	Benign neoplasm of spinal cord	0.0393%	0.0485%	0.0847%	0.0255%
744.23	Microtia	0.0657%	0.0000%	0.0021%	0.0000%
553.21	Incisional ventral hernia	0.0462%	0.0291%	0.0289%	0.0701%
527.2	Sialoadenitis	0.0486%	0.0388%	0.0289%	0.0446%
427.89	Other specified cardiac dysrhythmias	0.0435%	0.0775%	0.0310%	0.0510%
654.91	Other and unspecified abnormality of organs adn soft tissue	0.0597%	0.0000%	0.0062%	0.0223%
527.5	Sialolithiasis	0.0569%	0.0097%	0.0124%	0.0255%
151.0	Malignant neoplasm of cardia of stomach	0.0347%	0.0145%	0.0888%	0.0573%
144.9	Malignant neoplasm of floor of mouth, part unspecified	0.0120%	0.0921%	0.1756%	0.0255%
807.02	Fracture of two ribs, closed	0.0425%	0.0339%	0.0496%	0.0446%
682.4	Other cellulitis and abscess, hand, except fingers and thuml	0.0425%	0.0291%	0.0496%	0.0478%
621.0	Polyp of corpus uteri	0.0532%	0.0242%	0.0041%	0.0478%
574.51	Calculus of bile duct without mention of cholecystitis with	0.0425%	0.0485%	0.0413%	0.0478%
463	Acute tonsillitis	0.0467%	0.0436%	0.0289%	0.0414%
790.7	Bacteremia	0.0314%	0.1115%	0.0537%	0.0605%
654.11	Tumors of body of uterus, delivered, with or without menti	0.0597%	0.0048%	0.0000%	0.0191%
239.2	Neoplasm of unspecified nature of bone, soft tissue and ski	0.0481%	0.0194%	0.0289%	0.0446%
813.22	Fracture of shaft of ulna (alone), closed	0.0509%	0.0194%	0.0227%	0.0318%
823.02	Fracture of upper end of tibia and fibula, closed	0.0356%	0.0388%	0.0806%	0.0350%
230.3	Carcinoma in situ of colon	0.0379%	0.0436%	0.0496%	0.0637%
717.40	Derangement of lateral meniscus, unspecified	0.0453%	0.0485%	0.0227%	0.0446%
528.7	Other disturbances of oral epithelium, including tongue	0.0384%	0.0727%	0.0475%	0.0382%
441.2	Thoracic aneurysm without mention of rupture	0.0314%	0.0582%	0.0744%	0.0541%
228.04	Hemangioma of intra-abdominal structures	0.0439%	0.0388%	0.0393%	0.0318%
478.1	Other diseases of nasal cavity and sinuses	0.0476%	0.0339%	0.0248%	0.0287%
213.7	Benign neoplasm of long bones of lower limb	0.0555%	0.0000%	0.0124%	0.0159%
250.02	Diabetes mellitus without mention of complication, Type II	0.0199%	0.1503%	0.0310%	0.1306%
577.1	Chronic pancreatitis	0.0245%	0.0969%	0.0909%	0.0382%
426.0	Atrioventricular block, complete	0.0393%	0.0921%	0.0227%	0.0446%
576.2	Obstruction of bile duct	0.0319%	0.0727%	0.0599%	0.0478%
219.9	Benign neoplasm of uterus, part unspecified	0.0430%	0.0339%	0.0413%	0.0255%

ICD-9-CM	ICD-9-CM	PureO	ContiB	NewB	PastB
145.3	Malignant neoplasm of soft palate	0.0231%	0.0485%	0.1157%	0.0382%
157.2	Malignant neoplasm of tail of pancreas	0.0291%	0.0485%	0.0868%	0.0382%
808.8	Unspecified fracture of pelvis, closed	0.0365%	0.0291%	0.0682%	0.0255%
353.0	Brachial plexus lesions	0.0398%	0.0242%	0.0640%	0.0127%
153.7	Malignant neoplasm of splenic flexure	0.0347%	0.0436%	0.0682%	0.0287%
812.42	Fracture of lateral condyle of humerus, closed	0.0541%	0.0000%	0.0103%	0.0096%
824.9	Unspecified fracture of ankle, open	0.0351%	0.0048%	0.0868%	0.0191%
780.6	Fever	0.0268%	0.1018%	0.0516%	0.0637%
659.11	Failed medical or unspecified induction, delivered, with or	0.0541%	0.0000%	0.0000%	0.0223%
666.14	Other immediate postpartum hemorrhage,postpartum condi	0.0555%	0.0000%	0.0083%	0.0000%
815.03	Fracture of shaft of metacarpal bone(s), closed	0.0481%	0.0145%	0.0227%	0.0159%
150.3	Malignant neoplasm of upper third of esophagus	0.0162%	0.1503%	0.0930%	0.0382%
955.6	Injury to digital nerve	0.0509%	0.0048%	0.0145%	0.0127%
821.33	Supracondylar fracture of femur, open	0.0324%	0.0145%	0.0909%	0.0159%
574.70	Calculus of gallbladder and bile duct with other cholecystit	0.0421%	0.0339%	0.0165%	0.0510%
198.89	Secondary malignant neoplasm of other specified sites	0.0236%	0.0727%	0.0847%	0.0478%
186.9	Malignant neoplasm of other and unspecified testis	0.0449%	0.0048%	0.0434%	0.0064%
527.6	Mucocele	0.0472%	0.0048%	0.0207%	0.0223%
239.7	Neoplasm of unspecified nature of endocrine glands, and o	0.0435%	0.0436%	0.0207%	0.0223%
755.63	Other congenital deformity of hip (joint)	0.0365%	0.0291%	0.0475%	0.0318%
156.1	Malignant neoplasm of extrahepatic bile ducts	0.0296%	0.0630%	0.0640%	0.0318%
814.01	Fracture of navicular (scaphoid) of wrist, closed	0.0467%	0.0000%	0.0186%	0.0223%
474.11	Hypertrophy of tonsils alone	0.0481%	0.0097%	0.0103%	0.0191%
823.92	Fracture of unspecified part of tibia and fibula, open	0.0328%	0.0097%	0.0826%	0.0096%
727.51	Rupture of synovial cyst of popliteal space	0.0375%	0.0533%	0.0145%	0.0541%
611.1	Hypertrophy of breast	0.0467%	0.0000%	0.0145%	0.0255%
174.3	Malignant neoplasm of female breast, lower-inner quadrant	0.0291%	0.0582%	0.0640%	0.0318%
823.10	Fracture of upper end of tibia alone, open	0.0287%	0.0242%	0.0868%	0.0191%
728.89	Other disorders of muscle, ligament, and fascia	0.0301%	0.0679%	0.0537%	0.0318%
710.0	Systemic lupus erythematosus	0.0222%	0.1212%	0.0599%	0.0414%
530.11	Reflux esophagitis	0.0393%	0.0533%	0.0186%	0.0318%
598.9	Urethral stricture, unspecified	0.0342%	0.0533%	0.0124%	0.0764%
235.2	Neoplasm of uncertain behavior of stomach, intestines and	0.0393%	0.0291%	0.0227%	0.0414%
158.0	Malignant neoplasm of retroperitoneum	0.0328%	0.0485%	0.0558%	0.0223%
664.21	Third-degree perineal laceration, delivered, with or without	0.0481%	0.0000%	0.0041%	0.0255%
996.1	Mechanical complication of other vascular device, implant	0.0203%	0.1018%	0.0331%	0.0987%
823.82	Fracture of unspecified part of tibia and fibula, closed	0.0370%	0.0145%	0.0455%	0.0223%
789.00	Abdominal pain, unspecified site	0.0282%	0.0485%	0.0599%	0.0350%
227.3	Benign neoplasm of pituitary gland and craniopharyngeal d	0.0333%	0.0582%	0.0372%	0.0287%

ICD-9-CM	ICD-9-CM	PureO	ContiB	NewB	PastB
038.11	Staphylococcal aureus septicemia	0.0282%	0.0630%	0.0516%	0.0382%
996.62	Infection and inflammatory reaction due to other vascular d	0.0162%	0.1454%	0.0310%	0.0955%
174.1	Malignant neoplasm of female breast, central portion	0.0301%	0.0291%	0.0475%	0.0510%
715.26	Osteoarthrosis, localized, secondary, lower leg	0.0310%	0.0388%	0.0310%	0.0605%
618.8	Other specified genital prolapse	0.0314%	0.0533%	0.0248%	0.0573%
515	Postinflammatory pulmonary fibrosis	0.0333%	0.0485%	0.0269%	0.0446%
142.0	Malignant neoplasm of parotid gland	0.0351%	0.0194%	0.0496%	0.0159%
752.61	Hypospadias	0.0495%	0.0000%	0.0021%	0.0000%
722.83	Postlaminectomy syndrome, lumbar region	0.0203%	0.0630%	0.0702%	0.0541%
156.0	Malignant neoplasm of gallbladder	0.0222%	0.0679%	0.0599%	0.0541%
820.20	Unspecified fracture of trochanteric section of femur, close	0.0222%	0.0582%	0.0537%	0.0669%
738.0	Acquired deformity of nose	0.0425%	0.0097%	0.0124%	0.0223%
749.23	Complete cleft palate with cleft lip, bilateral	0.0490%	0.0000%	0.0021%	0.0000%
204.00	Acute lymphoid leukemia, without mention of remission	0.0217%	0.0969%	0.0640%	0.0287%
945.34	Burn of lower leg, full-thickness skin loss (third degree NC	0.0351%	0.0291%	0.0289%	0.0318%
802.8	Fracture of other facial bones, closed	0.0347%	0.0291%	0.0372%	0.0223%
786.6	Swelling, mass, or lump in chest	0.0314%	0.0485%	0.0351%	0.0350%
646.61	Infections of genitourinary tract in pregnancy, delivered, w	0.0467%	0.0000%	0.0000%	0.0159%
526.0	Developmental odontogenic cysts	0.0384%	0.0291%	0.0103%	0.0382%
574.91	Calculus of gallbladder and bile duct without cholecystitis	0.0402%	0.0291%	0.0083%	0.0287%
153.9	Malignant neoplasm of colon, unspecified	0.0305%	0.0339%	0.0496%	0.0287%
614.9	Unspecified inflammatory disease of female pelvic organs	0.0365%	0.0194%	0.0289%	0.0255%
427.1	Paroxysmal ventricular tachycardia	0.0264%	0.0872%	0.0269%	0.0541%
722.93	Lumbar intervertebral disc disorder	0.0333%	0.0291%	0.0413%	0.0191%
239.1	Neoplasm of unspecified nature of respiratory system	0.0370%	0.0194%	0.0227%	0.0287%
171.3	Malignant neoplasm of connective and other soft tissue of	0.0324%	0.0339%	0.0434%	0.0191%
802.21	Fracture of mandible, closed, condylar process	0.0365%	0.0291%	0.0331%	0.0064%
802.36	Fracture of mandible, open, symphysis of body	0.0356%	0.0145%	0.0434%	0.0064%
807.05	Fracture of five ribs, closed	0.0305%	0.0291%	0.0516%	0.0191%
780.59	Other sleep disturbances	0.0458%	0.0097%	0.0021%	0.0032%
717.7	Chondromalacia of patella	0.0356%	0.0242%	0.0248%	0.0287%
727.43	Ganglion, unspecified	0.0375%	0.0145%	0.0165%	0.0350%
611.0	Inflammatory disease of breast	0.0370%	0.0145%	0.0289%	0.0191%
593.70	Vesicoureteral reflux , unspecified or without reflux nephro	0.0467%	0.0000%	0.0041%	0.0000%
478.4	Polyp of vocal cord or larynx	0.0305%	0.0339%	0.0227%	0.0605%
428.9	Heart failure, unspecified	0.0222%	0.0679%	0.0393%	0.0701%
941.20	Burn of face and head, unspecified site, blisters, epidermal	0.0384%	0.0145%	0.0207%	0.0191%
729.1	Myalgia and myositis, unspecified	0.0199%	0.1018%	0.0455%	0.0510%
415.19	Other pulmonary embolism and infarction	0.0236%	0.0630%	0.0413%	0.0573%

ICD-9-CM	ICD-9-CM	PureO	ContiB	NewB	PastB
225.2	Benign neoplasm of cerebral meninges	0.0250%	0.0727%	0.0537%	0.0223%
212.6	Benign neoplasm of thymus	0.0342%	0.0436%	0.0269%	0.0191%
385.32	Cholesteatoma of middle ear	0.0356%	0.0291%	0.0289%	0.0127%
802.25	Fracture of mandible, closed, angle of jaw	0.0361%	0.0194%	0.0351%	0.0032%
600.1	Nodular prostate	0.0314%	0.0194%	0.0289%	0.0446%
011.93	Pulmonary tuberculosis, unspecified, tubercle bacilli found	0.0236%	0.0824%	0.0455%	0.0318%
682.3	Other cellulitis and abscess, upper arm and forearm	0.0273%	0.0242%	0.0351%	0.0573%
618.5	Prolapse of vaginal vault after hysterectomy	0.0328%	0.0242%	0.0289%	0.0287%
524.10	Unspecified anomaly of relationship of jaw to cranial base	0.0439%	0.0000%	0.0083%	0.0000%
530.81	Esophageal reflux	0.0342%	0.0388%	0.0186%	0.0255%
478.79	Other diseases of larynx	0.0310%	0.0339%	0.0248%	0.0414%
511.8	Pleurisy, other specified forms of effusion, except tubercul	0.0305%	0.0388%	0.0248%	0.0414%
453.8	Embolism and thrombosis of other specified veins	0.0217%	0.0582%	0.0475%	0.0541%
250.72	Diabetes with peripheral circulatory disorders, Type II [nor	0.0203%	0.0630%	0.0578%	0.0446%
785.4	Gangrene	0.0227%	0.0436%	0.0558%	0.0414%
608.83	Vascular disorders of male genital organs	0.0277%	0.0242%	0.0496%	0.0287%
212.0	Benign neoplasm of nasal cavities, middle ear and accessor	0.0384%	0.0194%	0.0021%	0.0318%
883.2	Open wound of finger(s), with tendon involvement	0.0365%	0.0145%	0.0269%	0.0064%
722.73	Lumbar intervertebral disc disorder with myelopathy	0.0254%	0.0630%	0.0496%	0.0159%
211.1	Benign neoplasm of stomach	0.0296%	0.0339%	0.0207%	0.0510%
161.1	Malignant neoplasm of supraglottis	0.0157%	0.0242%	0.1095%	0.0159%
188.2	Malignant neoplasm of lateral wall of urinary bladder	0.0305%	0.0388%	0.0186%	0.0446%
784.2	Swelling, mass, or lump in head and neck	0.0347%	0.0194%	0.0124%	0.0350%
752.51	Undescended testis	0.0430%	0.0000%	0.0041%	0.0032%
528.3	Cellulitis and abscess of oral soft tissues	0.0282%	0.0388%	0.0331%	0.0318%
815.00	Fracture in unspecified site of metacarpal bone(s), closed	0.0333%	0.0145%	0.0248%	0.0223%
560.2	Volvulus	0.0296%	0.0145%	0.0186%	0.0573%
171.5	Malignant neoplasm of connective and other soft tissue of	0.0296%	0.0194%	0.0351%	0.0287%
953.4	Injury to brachial plexus	0.0305%	0.0145%	0.0475%	0.0032%
813.33	Fracture of shaft of radius with ulna, open	0.0361%	0.0000%	0.0269%	0.0064%
821.21	Fracture of condyle of femur, closed	0.0305%	0.0194%	0.0331%	0.0223%
726.2	Other affections of shoulder region, not elsewhere classifie	0.0217%	0.0436%	0.0496%	0.0414%
437.3	Cerebral aneurysm, nonruptured	0.0157%	0.0969%	0.0537%	0.0414%
952.03	C1-C4 level with central cord syndrome	0.0171%	0.0291%	0.0930%	0.0127%
745.5	Ostium secundum type atrial septal defect	0.0347%	0.0339%	0.0124%	0.0127%
722.91	Cervical intervertebral disc disorder	0.0250%	0.0242%	0.0413%	0.0414%
389.9	Unspecified hearing loss	0.0347%	0.0145%	0.0186%	0.0159%
805.02	Fracture of second cervical vertebra, closed	0.0227%	0.0485%	0.0537%	0.0191%
780.2	Syncope and collapse	0.0176%	0.0824%	0.0516%	0.0350%

ICD-9-CM	ICD-9-CM	PureO	ContiB	NewB	PastB
145.2	Malignant neoplasm of hard palate	0.0125%	0.0485%	0.0992%	0.0191%
173.3	Malignant neoplasm of skin of other and unspecified parts	0.0296%	0.0242%	0.0227%	0.0350%
927.10	Crushing injury of forearm	0.0259%	0.0048%	0.0661%	0.0032%
816.11	Fracture of middle or proximal phalanx or phalanges, open	0.0310%	0.0388%	0.0227%	0.0127%
575.11	Chronic cholecystitis	0.0338%	0.0097%	0.0124%	0.0287%
825.35	Fracture of metatarsal bone(s), open	0.0324%	0.0097%	0.0310%	0.0064%
642.71	Pre-eclampsia or eclampsia superimposed on pre-existing h	0.0370%	0.0000%	0.0062%	0.0191%
576.8	Other specified disorders of biliary tract	0.0231%	0.0388%	0.0393%	0.0382%
219.1	Benign neoplasm of corpus uteri	0.0310%	0.0242%	0.0145%	0.0318%
952.00	C1-C4 level with unspecified spinal cord injury	0.0134%	0.0630%	0.0806%	0.0223%
485	Bronchopneumonia, organism unspecified	0.0129%	0.0921%	0.0351%	0.0764%
276.1	Hyposmolality and/or hyponatremia	0.0120%	0.1115%	0.0227%	0.0892%
881.20	Open wound of forearm, with tendon involvement	0.0282%	0.0145%	0.0455%	0.0032%
866.02	Laceration to kidney, without mention of open wound into	0.0342%	0.0097%	0.0227%	0.0000%
744.42	Branchial cleft cyst	0.0375%	0.0000%	0.0124%	0.0000%
718.31	Recurrent dislocation of joint, shoulder region	0.0310%	0.0097%	0.0269%	0.0159%
622.1	Dysplasia of cervix (uteri)	0.0291%	0.0145%	0.0124%	0.0478%
394.0	Mitral stenosis	0.0185%	0.0339%	0.0248%	0.0892%
164.0	Malignant neoplasm of thymus	0.0185%	0.0727%	0.0393%	0.0414%
853.06	Other and unspecified intracranial hemorrhage following in	0.0250%	0.0291%	0.0434%	0.0159%
652.31	Transverse or oblique presentation, delivered, with or witho	0.0384%	0.0000%	0.0021%	0.0064%
999.3	Complication of medical care, other infection	0.0088%	0.1163%	0.0289%	0.0892%
751.69	Other anomalies of gallbladder, bile ducts, and liver	0.0347%	0.0194%	0.0103%	0.0032%
569.1	Rectal prolapse	0.0245%	0.0339%	0.0248%	0.0414%
642.31	Transient hypertension of pregnancy, delivered, with or wit	0.0379%	0.0000%	0.0000%	0.0096%
620.5	Torsion of ovary, ovarian pedicle or fallopian tube	0.0356%	0.0048%	0.0103%	0.0064%
351.0	Bell's palsy	0.0236%	0.0145%	0.0537%	0.0159%
146.9	Malignant neoplasm of oropharynx, unspecified	0.0176%	0.0339%	0.0764%	0.0096%
806.4	Fracture of lumbar with spinal cord injury, closed	0.0153%	0.0291%	0.0826%	0.0159%
751.61	Biliary atresia	0.0388%	0.0000%	0.0000%	0.0000%
343.0	Diplegic infantile cerebral palsy	0.0365%	0.0048%	0.0041%	0.0064%
784.0	Headache	0.0166%	0.0824%	0.0413%	0.0318%
478.22	Parapharyngeal abscess	0.0310%	0.0000%	0.0248%	0.0127%
228.01	Hemangioma of skin and subcutaneous tissue	0.0356%	0.0097%	0.0021%	0.0096%
379.23	Vitreous hemorrhage	0.0291%	0.0097%	0.0124%	0.0350%
711.05	Pyogenic arthritis, pelvic region and thigh	0.0180%	0.0339%	0.0475%	0.0382%
560.0	Intussusception	0.0370%	0.0000%	0.0000%	0.0032%
239.4	Neoplasm of unspecified nature of bladder	0.0213%	0.0194%	0.0351%	0.0446%
996.67	Infection and inflammatory reaction due to other internal o	0.0236%	0.0339%	0.0331%	0.0191%

ICD-9-CM	ICD-9-CM	PureO	ContiB	NewB	PastB
826.0	Fracture of one or more phalanges of foot, closed	0.0273%	0.0048%	0.0248%	0.0255%
730.15	Chronic osteomyelitis, pelvic region and thigh	0.0208%	0.0291%	0.0455%	0.0223%
726.0	Adhesive capsulitis of shoulder	0.0231%	0.0339%	0.0227%	0.0382%
648.81	Abnormal glucose tolerance in the mother classifiable elsev	0.0356%	0.0048%	0.0021%	0.0032%
522.8	Radicular cyst	0.0324%	0.0339%	0.0000%	0.0096%
423.9	Unspecified disease of pericardium	0.0148%	0.0679%	0.0434%	0.0414%
211.4	Benign neoplasm of rectum and anal canal	0.0245%	0.0145%	0.0269%	0.0350%
358.0	Myasthenia gravis	0.0208%	0.0533%	0.0248%	0.0382%
824.5	Fracture of bimalleolar of ankle, open	0.0250%	0.0097%	0.0393%	0.0127%
532.90	Duodenal ulcer, unspecified as acute or chronic, without m	0.0231%	0.0145%	0.0372%	0.0255%
955.1	Injury to median nerve	0.0264%	0.0000%	0.0413%	0.0032%
989.4	Toxic effect of other pesticides, not elsewhere classified	0.0143%	0.0388%	0.0599%	0.0318%
955.2	Injury to ulnar nerve	0.0287%	0.0048%	0.0310%	0.0000%
998.83	Non-healing surgical wound	0.0245%	0.0291%	0.0207%	0.0287%
998.11	Hemorrhage complicating a procedure	0.0240%	0.0291%	0.0186%	0.0350%
574.71	Calculus of gallbladder and bile duct with other cholecystit	0.0282%	0.0097%	0.0165%	0.0223%
375.56	Stenosis of nasolacrimal duct, acquired	0.0305%	0.0097%	0.0083%	0.0191%
038.0	Streptococcal septicemia	0.0166%	0.0485%	0.0372%	0.0446%
935.1	Foreign body in esophagus	0.0305%	0.0097%	0.0103%	0.0127%
553.1	Umbilical hernia	0.0264%	0.0194%	0.0145%	0.0287%
228.1	Lymphangioma, any site	0.0319%	0.0000%	0.0041%	0.0191%
331.3	Communicating hydrocephalus	0.0185%	0.0679%	0.0248%	0.0350%
009.0	Infectious colitis, enteritis and gastroenteritis	0.0199%	0.0339%	0.0227%	0.0510%
871.0	Ocular laceration without prolapse of intraocular tissue	0.0264%	0.0048%	0.0227%	0.0223%
754.0	Certain congenital musculoskeletal deformities of skull, fac	0.0333%	0.0048%	0.0021%	0.0064%
413.9	Other and unspecified angina pectoris	0.0120%	0.1018%	0.0186%	0.0637%
143.0	Malignant neoplasm of upper gum	0.0079%	0.0679%	0.0868%	0.0096%
892.1	Open wound of foot except toe(s) alone, complicated	0.0254%	0.0242%	0.0207%	0.0159%
753.10	Cystic kidney disease, unspecified	0.0222%	0.0242%	0.0227%	0.0350%
801.21	Fracture of base of skull, closed with subarachnoid, subdur	0.0227%	0.0194%	0.0413%	0.0064%
660.21	Obstruction by abnormal pelvic soft tissues, delivered, with	0.0342%	0.0000%	0.0000%	0.0032%
595.1	Chronic interstitial cystitis	0.0134%	0.0727%	0.0372%	0.0414%
560.89	Other specified intestinal obstruction	0.0250%	0.0097%	0.0269%	0.0191%
482.1	Pneumonia due to Pseudomonas	0.0143%	0.0533%	0.0227%	0.0701%
239.8	Neoplasm of unspecified nature of other specified sites	0.0277%	0.0194%	0.0103%	0.0191%
172.7	Malignant melanoma of skin of lower limb including hip	0.0217%	0.0291%	0.0269%	0.0287%
808.41	Fracture of ilium, closed	0.0208%	0.0242%	0.0434%	0.0096%
753.21	Congenital obstruction of ureteropelvic junction	0.0310%	0.0048%	0.0062%	0.0096%
686.9	Unspecified local infections of skin and subcutaneous tissu	0.0240%	0.0145%	0.0269%	0.0191%

ICD-9-CM	ICD-9-CM	PureO	ContiB	NewB	PastB
641.21	Premature separation of placenta, delivered, with or withou	0.0319%	0.0000%	0.0000%	0.0159%
212.5	Benign neoplasm of mediastinum	0.0268%	0.0145%	0.0207%	0.0096%
331.4	Obstructive hydrocephalus	0.0153%	0.0436%	0.0372%	0.0446%
213.1	Benign neoplasm of lower jaw bone	0.0199%	0.0291%	0.0413%	0.0159%
211.5	Benign neoplasm of liver and biliary passages	0.0273%	0.0242%	0.0062%	0.0191%
238.0	Neoplasm of uncertain behavior of bone and articular cartil	0.0296%	0.0048%	0.0103%	0.0096%
861.21	Contusion of lung,without mention of open wound into tho	0.0240%	0.0097%	0.0351%	0.0032%
723.8	Other syndromes affecting cervical region	0.0180%	0.0242%	0.0372%	0.0318%
718.44	Contracture of joint, hand	0.0153%	0.0145%	0.0744%	0.0000%
656.61	Excessive fetal growth affecting management of mother, de	0.0301%	0.0000%	0.0000%	0.0223%
287.3	Primary thrombocytopenia	0.0213%	0.0436%	0.0207%	0.0223%
157.1	Malignant neoplasm of body of pancreas	0.0153%	0.0485%	0.0475%	0.0191%
883.0	Open wound of finger(s), without mention of complication	0.0273%	0.0000%	0.0186%	0.0096%
V55.3	Attention to colostomy	0.0250%	0.0145%	0.0124%	0.0255%
593.9	Unspecified disorder of kidney and ureter	0.0213%	0.0145%	0.0165%	0.0446%
457.1	Other lymphedema	0.0139%	0.0339%	0.0558%	0.0223%
478.29	Other diseases of pharynx	0.0254%	0.0194%	0.0165%	0.0127%
221.0	Benign neoplasm of fallopian tube and uterine ligaments	0.0310%	0.0048%	0.0062%	0.0000%
173.5	Malignant neoplasm of skin of trunk, except scrotum	0.0240%	0.0048%	0.0289%	0.0127%
813.54	Fracture of radius with ulna, lower end, open	0.0194%	0.0194%	0.0269%	0.0350%
844.8	Sprains and strains of other specified sites of knee and leg	0.0250%	0.0145%	0.0165%	0.0159%
813.52	Other fractures of distal end of radius (alone), open	0.0222%	0.0097%	0.0331%	0.0127%
836.2	Other tear of cartilage or meniscus of knee, current	0.0287%	0.0048%	0.0083%	0.0096%
608.4	Other inflammatory disorders of male genital organs	0.0245%	0.0242%	0.0165%	0.0127%
535.50	Unspecified gastritis and gastroduodenitis, without mention	0.0264%	0.0097%	0.0083%	0.0223%
482.83	Pneumonia due to other gram-negative bacteria	0.0116%	0.0436%	0.0186%	0.0860%
215.3	Benign neoplasm of connective and other soft tissue of low	0.0277%	0.0000%	0.0083%	0.0191%
955.3	Injury to radial nerve	0.0231%	0.0097%	0.0310%	0.0064%
997.4	Digestive system complications	0.0199%	0.0194%	0.0351%	0.0159%
655.81	Other known or suspected fetal abnormality, not elsewhere	0.0305%	0.0000%	0.0041%	0.0032%
664.31	Fourth-degree perineal laceration, delivered, with or withou	0.0310%	0.0000%	0.0000%	0.0064%
493.92	Asthma, unspecified, with acute exacerbation	0.0083%	0.0921%	0.0207%	0.0701%
864.04	Major laceration to liver, without mention of open wound i	0.0227%	0.0000%	0.0351%	0.0064%
812.40	Fracture in unspecified part of lower end of humerus, close	0.0259%	0.0097%	0.0124%	0.0127%
611.72	Lump or mass in breast	0.0250%	0.0097%	0.0145%	0.0159%
553.20	Ventral hernia, unspecified	0.0240%	0.0048%	0.0062%	0.0382%
433.10	Occlusion and stenosis of carotid artery without mention of	0.0125%	0.0775%	0.0269%	0.0382%
047.9	Unspecified viral meningitis	0.0217%	0.0145%	0.0248%	0.0191%
807.06	Fracture of six ribs, closed	0.0203%	0.0242%	0.0289%	0.0127%

ICD-9-CM	ICD-9-CM	PureO	ContiB	NewB	PastB
754.30	Congenital dislocation of hip, unilateral	0.0291%	0.0048%	0.0041%	0.0032%
605	Redundant prepuce and phimosis	0.0291%	0.0048%	0.0021%	0.0064%
432.1	Subdural hemorrhage	0.0166%	0.0388%	0.0248%	0.0350%
441.3	Abdominal aneurysm, ruptured	0.0203%	0.0145%	0.0289%	0.0191%
289.3	Lymphadenitis, unspecified, except mesenteric	0.0231%	0.0145%	0.0207%	0.0127%
324.1	Intraspinal abscess	0.0129%	0.0582%	0.0475%	0.0127%
042	Human immunodeficiency virus（HIV）infection disease	0.0102%	0.0727%	0.0434%	0.0287%
152.0	Malignant neoplasm of duodenum	0.0148%	0.0194%	0.0455%	0.0287%
941.29	Burn of multiple sites (except with eye) of face, head and n	0.0264%	0.0000%	0.0165%	0.0032%
891.2	Open wound of knee, leg (except thigh) and ankle, with ter	0.0190%	0.0048%	0.0289%	0.0287%
815.04	Fracture of neck of metacarpal bone(s), closed	0.0250%	0.0097%	0.0062%	0.0191%
695.1	Erythema multiforme	0.0213%	0.0242%	0.0165%	0.0191%
529.8	Other specified conditions of the tongue	0.0199%	0.0194%	0.0145%	0.0318%
570	Acute and subacute necrosis of liver	0.0180%	0.0194%	0.0186%	0.0382%
574.30	Calculus of bile duct with acute cholecystitis, without ment	0.0203%	0.0194%	0.0145%	0.0287%
800.26	Fracture of vault of skull, closed with subarachnoid, subdur	0.0231%	0.0048%	0.0207%	0.0064%
682.5	Other cellulitis and abscess, buttock	0.0199%	0.0145%	0.0248%	0.0159%
528.5	Diseases of lips	0.0222%	0.0145%	0.0145%	0.0159%
593.89	Other specified disorders of kidney and ureter	0.0213%	0.0194%	0.0227%	0.0064%
239.3	Neoplasm of unspecified nature of breast	0.0217%	0.0097%	0.0103%	0.0287%
892.0	Open wound of foot except toe(s) alone, without mention c	0.0236%	0.0000%	0.0186%	0.0064%
936	Foreign body in intestine and colon	0.0217%	0.0145%	0.0124%	0.0191%
749.01	Complete cleft palate, unilateral	0.0287%	0.0000%	0.0000%	0.0000%
736.81	Unequal leg length (acquired)	0.0273%	0.0000%	0.0041%	0.0032%
726.19	Rotator cuff syndrome of shoulder and allied disorders, oth	0.0125%	0.0291%	0.0331%	0.0414%
721.42	Lumbar spondylosis with myelopathy	0.0166%	0.0145%	0.0372%	0.0159%
590.80	Pyelonephritis, unspecified	0.0162%	0.0339%	0.0248%	0.0255%
215.0	Benign neoplasm of connective and other soft tissue of hea	0.0250%	0.0048%	0.0103%	0.0064%
242.20	Toxic multinodular goiter without mention of thyrotoxic cr	0.0208%	0.0097%	0.0083%	0.0350%
989.5	Toxic effect of venom	0.0199%	0.0291%	0.0165%	0.0127%
945.32	Burn of foot, full-thickness skin loss (third degree NOS)	0.0217%	0.0048%	0.0124%	0.0223%
802.28	Fracture of mandible, closed, body, other and unspecified	0.0236%	0.0145%	0.0103%	0.0064%
824.3	Fracture of lateral malleolus of ankle, open	0.0213%	0.0000%	0.0227%	0.0127%
715.37	Osteoarthrosis, localized, not specified whether primary or	0.0208%	0.0097%	0.0165%	0.0191%
482.0	Pneumonia due to Klebsiella pneumoniae	0.0079%	0.0582%	0.0289%	0.0573%
426.13	Other second degree atrioventricular block	0.0166%	0.0291%	0.0207%	0.0287%
396.3	Mitral valve insufficiency and aortic valve insufficiency	0.0111%	0.0485%	0.0351%	0.0318%
332.0	Paralysis agitans	0.0051%	0.0921%	0.0083%	0.0860%
211.6	Benign neoplasm of pancreas, except islets of Langerhans	0.0153%	0.0339%	0.0351%	0.0127%

ICD-9-CM	ICD-9-CM	PureO	ContiB	NewB	PastB
887.0	Traumatic amputation, unilateral, below elbow, without me	0.0111%	0.0194%	0.0661%	0.0000%
959.01	Head injury, unspecified	0.0162%	0.0145%	0.0289%	0.0255%
892.2	Open wound of foot except toe(s) alone, with tendon involv	0.0208%	0.0000%	0.0269%	0.0064%
736.42	Genu varum (acquired)	0.0259%	0.0000%	0.0083%	0.0000%
604.90	Orchitis and epididymitis, unspecified	0.0180%	0.0242%	0.0227%	0.0159%
426.7	Anomalous atrioventricular excitation	0.0268%	0.0048%	0.0021%	0.0000%
455.3	External hemorrhoids without mention of complication	0.0231%	0.0048%	0.0145%	0.0064%
148.1	Malignant neoplasm of pyriform sinus of hypopharynx	0.0102%	0.0533%	0.0537%	0.0032%
853.01	Other and unspecified intracranial hemorrhage following in	0.0143%	0.0291%	0.0372%	0.0127%
805.6	Fracture of sacrum and coccyx, closed	0.0134%	0.0242%	0.0475%	0.0064%
745.2	Tetralogy of fallot	0.0259%	0.0048%	0.0041%	0.0000%
780.39	Other convulsions	0.0102%	0.0727%	0.0165%	0.0446%
749.22	Incomplete cleft palate with cleft lip, unilateral	0.0268%	0.0000%	0.0021%	0.0000%
681.00	Finger cellulitis and abscess, unspecified	0.0203%	0.0145%	0.0186%	0.0096%
565.0	Anal fissure	0.0231%	0.0145%	0.0041%	0.0127%
456.8	Varices of other sites	0.0153%	0.0242%	0.0145%	0.0446%
343.9	Infantile cerebral palsy, unspecified	0.0240%	0.0097%	0.0041%	0.0096%
184.4	Malignant neoplasm of vulva, unspecified	0.0162%	0.0242%	0.0331%	0.0096%
V26.0	Tuboplasty or vasoplasty after previous sterilization	0.0254%	0.0000%	0.0000%	0.0096%
996.73	Other complications due to renal dialysis device, implant, a	0.0092%	0.0679%	0.0124%	0.0573%
820.02	Fracture of midcervical section of femur, closed	0.0166%	0.0242%	0.0269%	0.0127%
802.5	Fracture of malar and maxillary bones, open	0.0190%	0.0048%	0.0289%	0.0064%
733.43	Aseptic necrosis of medial femoral condyle	0.0171%	0.0048%	0.0248%	0.0255%
664.11	Second-degree perineal laceration, delivered, with or witho	0.0264%	0.0000%	0.0000%	0.0032%
718.81	Other joint derangement，not elsewhere classified, should	0.0217%	0.0048%	0.0124%	0.0127%
716.16	Traumatic arthropathy, lower leg	0.0153%	0.0291%	0.0207%	0.0287%
557.0	Acute vascular insufficiency of intestine	0.0180%	0.0097%	0.0103%	0.0382%
619.1	Digestive-genital tract fistula, female	0.0166%	0.0242%	0.0207%	0.0223%
464.30	Acute epiglottitis without mention of obstruction	0.0213%	0.0097%	0.0124%	0.0127%
943.31	Burn of forearm, full-thickness skin loss (third degree NOS	0.0231%	0.0048%	0.0103%	0.0032%
922.1	Contusion of chest wall	0.0148%	0.0194%	0.0351%	0.0127%
813.11	Fracture of olecranon process of ulna, open	0.0180%	0.0097%	0.0207%	0.0191%
732.7	Osteochondritis dissecans	0.0213%	0.0145%	0.0083%	0.0127%
707.15	Ulcer of other part of foot	0.0143%	0.0194%	0.0269%	0.0287%
535.00	Acute gastritis, without mention of hemorrhage	0.0227%	0.0194%	0.0041%	0.0064%
528.4	Cysts of oral soft tissues	0.0213%	0.0000%	0.0041%	0.0287%
215.2	Benign neoplasm of connective and other soft tissue of upp	0.0217%	0.0048%	0.0124%	0.0096%
284.8	Other specified aplastic anemias	0.0143%	0.0388%	0.0227%	0.0223%
890.0	Open wound of hip and thigh, without mention of complica	0.0194%	0.0097%	0.0207%	0.0064%

ICD-9-CM	ICD-9-CM	PureO	ContiB	NewB	PastB
815.01	Fracture of base of thumb (first) metacarpal, closed	0.0203%	0.0097%	0.0103%	0.0159%
729.9	Other and unspecified disorders of soft tissue	0.0134%	0.0291%	0.0227%	0.0318%
730.27	Unspecified osteomyelitis, ankle and foot	0.0157%	0.0339%	0.0165%	0.0223%
569.41	Ulcer of rectum and anus	0.0157%	0.0388%	0.0124%	0.0255%
250.40	Diabetes with renal manifestations, Type II [non-insulin de	0.0106%	0.0679%	0.0227%	0.0255%
238.7	Neoplasm of uncertain behavior of other lymphatic and hen	0.0111%	0.0436%	0.0186%	0.0446%
361.81	Traction detachment of retina	0.0199%	0.0048%	0.0165%	0.0127%
174.0	Malignant neoplasm of female breast, nipple and areola	0.0143%	0.0194%	0.0310%	0.0191%
815.02	Fracture of base of other metacarpal bone(s), closed	0.0222%	0.0048%	0.0062%	0.0096%
807.01	Fracture of one rib, closed	0.0203%	0.0097%	0.0124%	0.0096%
728.0	Infective myositis	0.0139%	0.0097%	0.0413%	0.0096%
727.89	Other disorders of synovium, tendon, and bursa	0.0143%	0.0097%	0.0310%	0.0223%
947.2	Burn of esophagus	0.0129%	0.0242%	0.0269%	0.0255%
881.00	Open wound of forearm, without mention of complicatiom	0.0199%	0.0097%	0.0165%	0.0032%
863.89	Injury to other gastrointestinal sites, without mention of op	0.0157%	0.0097%	0.0351%	0.0032%
852.22	Subdural hemorrhage following injury without mention of	0.0079%	0.0436%	0.0455%	0.0191%
852.04	Subarachnoid hemorrhage following injury without mentio	0.0143%	0.0145%	0.0310%	0.0159%
730.26	Unspecified osteomyelitis, lower leg	0.0176%	0.0048%	0.0227%	0.0127%
656.51	Poor fetal growth affecting management of mother, deliver	0.0217%	0.0000%	0.0041%	0.0159%
524.04	Mandibular hypoplasia	0.0240%	0.0000%	0.0000%	0.0064%
378.15	Alternating exotropia	0.0236%	0.0000%	0.0021%	0.0064%
238.1	Neoplasm of uncertain behavior of connective and other so	0.0199%	0.0000%	0.0103%	0.0191%
117.5	Cryptococcosis	0.0125%	0.0388%	0.0186%	0.0318%
942.34	Burn of back (any part), full-thickness skin loss (third degr	0.0185%	0.0048%	0.0227%	0.0032%
945.36	Burn of thigh (any part), full-thickness skin loss (third degr	0.0213%	0.0000%	0.0083%	0.0096%
V58.49	Other specified aftercare following surgery	0.0199%	0.0048%	0.0103%	0.0127%
821.31	Fracture of condyle of femur, open	0.0171%	0.0048%	0.0310%	0.0000%
733.14	Pathologic fracture of neck of femur	0.0120%	0.0145%	0.0289%	0.0318%
757.6	Specified anomalies of breast	0.0190%	0.0097%	0.0103%	0.0159%
780.09	Other alteration of consciousness	0.0088%	0.0388%	0.0331%	0.0318%
724.3	Sciatica	0.0143%	0.0194%	0.0165%	0.0318%
524.3	Anomalies of tooth position	0.0222%	0.0000%	0.0000%	0.0159%
526.89	Other specified diseases of the jaws	0.0116%	0.0339%	0.0413%	0.0032%
621.5	Intrauterine synechiae	0.0222%	0.0000%	0.0041%	0.0096%
210.4	Benign neoplasm of other and unspecified parts of mouth	0.0180%	0.0194%	0.0145%	0.0096%
151.1	Malignant neoplasm of pylorus of stomach	0.0153%	0.0339%	0.0227%	0.0064%
173.7	Malignant neoplasm of skin of lower limb, including hip	0.0162%	0.0339%	0.0083%	0.0223%
746.4	Congenital insufficiency of aortic valve	0.0111%	0.0388%	0.0289%	0.0191%
681.10	Toe cellulitis and abscess, unspecified	0.0134%	0.0291%	0.0165%	0.0287%

ICD-9-CM	ICD-9-CM	PureO	ContiB	NewB	PastB
654.43	Other abnormalities in shape or position of gravid uterus ar	0.0231%	0.0000%	0.0021%	0.0032%
607.1	Balanoposthitis	0.0240%	0.0000%	0.0000%	0.0000%
562.10	Diverticulosis of colon (without mention of hemorrhage)	0.0157%	0.0242%	0.0165%	0.0159%
494.0	Bronchiectasis without acute exacerbation	0.0129%	0.0582%	0.0062%	0.0287%
079.99	Unspecified virus infection in condition classified elsewher	0.0120%	0.0291%	0.0269%	0.0223%
881.22	Open wound of wrist, with tendon involvement	0.0162%	0.0097%	0.0186%	0.0159%
873.43	Open wound of lip, without mention of complication	0.0208%	0.0048%	0.0041%	0.0096%
724.4	Thoracic or lumbosacral neuritis or radiculitis, unspecified	0.0088%	0.0485%	0.0351%	0.0159%
717.5	Derangement of meniscus, not elsewhere classified	0.0180%	0.0097%	0.0083%	0.0191%
524.09	Other specified anomaly of jaw size	0.0227%	0.0000%	0.0021%	0.0032%
569.49	Other specified disorders of rectum and anus	0.0139%	0.0194%	0.0124%	0.0350%
211.8	Benign neoplasm of retroperitoneum and peritoneum	0.0171%	0.0145%	0.0186%	0.0064%
230.4	Carcinoma in situ of rectum	0.0153%	0.0242%	0.0124%	0.0223%
882.0	Open wound of hand except finger(s) alone, without mentio	0.0171%	0.0048%	0.0207%	0.0064%
863.20	Injury to small intestine,without mention of open wound in	0.0148%	0.0242%	0.0207%	0.0096%
852.02	Subarachnoid hemorrhage following injury without mentio	0.0143%	0.0097%	0.0248%	0.0159%
730.17	Chronic osteomyelitis, ankle and foot	0.0171%	0.0145%	0.0165%	0.0064%
755.01	Polydactyly of fingers	0.0231%	0.0000%	0.0000%	0.0000%
759.6	Other hamartoses, not elsewhere classified	0.0176%	0.0145%	0.0124%	0.0096%
727.41	Ganglion of joint	0.0190%	0.0048%	0.0083%	0.0127%
724.01	Spinal stenosis, thoracic region	0.0088%	0.0388%	0.0351%	0.0191%
550.93	Inguinal hernia, without mention of obstruction or gangren	0.0171%	0.0145%	0.0083%	0.0191%
464.0	Acute laryngitis	0.0194%	0.0048%	0.0062%	0.0127%
225.4	Benign neoplasm of spinal meninges	0.0102%	0.0339%	0.0372%	0.0096%
V51	Aftercare involving the use of plastic surgery	0.0139%	0.0145%	0.0227%	0.0159%
816.12	Fracture of distal phalanx or phalanges, open	0.0180%	0.0048%	0.0103%	0.0127%
807.09	Unspecified fracture of multiple ribs, closed	0.0116%	0.0145%	0.0248%	0.0287%
696.1	Other psoriasis	0.0079%	0.0485%	0.0227%	0.0350%
593.81	Vascular disorders of kidney	0.0116%	0.0194%	0.0248%	0.0255%
552.21	Incisional ventral hernia, with obstrution	0.0143%	0.0097%	0.0207%	0.0191%
574.61	Calculus of gallbladder and bile duct with acute cholecystit	0.0166%	0.0097%	0.0103%	0.0191%
455.7	Unspecified thrombosed hemorrhoids	0.0153%	0.0194%	0.0186%	0.0096%
289.59	Other diseases of spleen	0.0185%	0.0048%	0.0083%	0.0127%
161.9	Malignant neoplasm of larynx, unspecified	0.0102%	0.0339%	0.0393%	0.0032%
882.2	Open wound of hand except finger(s) alone, with tendon in	0.0190%	0.0000%	0.0103%	0.0064%
812.43	Fracture of medial condyle of humerus, closed	0.0208%	0.0000%	0.0021%	0.0064%
755.02	Polydactyly of toes	0.0213%	0.0048%	0.0021%	0.0000%
749.02	Incomplete cleft palate, unilateral	0.0222%	0.0000%	0.0000%	0.0000%
750.29	Other specified anomalies of pharynx	0.0222%	0.0000%	0.0000%	0.0000%

ICD-9-CM	ICD-9-CM	PureO	ContiB	NewB	PastB
481	Pneumococcal pneumonia [streptococcus pneumoniae pneu	0.0157%	0.0048%	0.0186%	0.0127%
478.0	Hypertrophy of nasal turbinates	0.0171%	0.0097%	0.0083%	0.0159%
370.00	Corneal ulcer, unspecified	0.0116%	0.0388%	0.0145%	0.0255%
427.32	Atrial flutter	0.0153%	0.0242%	0.0062%	0.0223%
236.0	Neoplasm of uncertain behavior of uterus	0.0208%	0.0000%	0.0021%	0.0064%
307.81	Tension headache	0.0055%	0.0533%	0.0351%	0.0255%
823.80	Fracture of unspecified part of tibia alone, closed	0.0139%	0.0097%	0.0289%	0.0032%
865.04	Massive parenchyma disruption in spleen, without mention	0.0148%	0.0000%	0.0289%	0.0032%
852.24	Subdural hemorrhage following injury without mention of	0.0116%	0.0291%	0.0269%	0.0096%
813.07	Other and unspecified fractures of proximal end of radius (	0.0157%	0.0097%	0.0124%	0.0159%
825.1	Fracture of calcaneus, open	0.0125%	0.0097%	0.0351%	0.0032%
574.01	Calculus of gallbladder with acute cholecystitis with obstru	0.0180%	0.0000%	0.0041%	0.0191%
525.8	Other specified disorders of the teeth and supporting structu	0.0208%	0.0048%	0.0000%	0.0032%
580.9	Acute glomerulonephritis with unspecified pathological les	0.0079%	0.0339%	0.0227%	0.0382%
530.10	Esophagitis, unspecified	0.0213%	0.0048%	0.0000%	0.0000%
387.9	Otosclerosis, unspecified	0.0143%	0.0097%	0.0207%	0.0127%
385.31	Cholesteatoma of attic	0.0190%	0.0000%	0.0083%	0.0064%
157.8	Malignant neoplasm of other specified sites of pancreas	0.0106%	0.0339%	0.0331%	0.0032%
945.24	Burn of lower leg, blisters, epidermal loss (second degree)	0.0166%	0.0048%	0.0083%	0.0159%
865.03	Laceration extending into parenchyma of spleen, without m	0.0143%	0.0000%	0.0310%	0.0000%
813.03	Monteggia's fracture,closed	0.0190%	0.0000%	0.0083%	0.0032%
839.04	Dislocations of fourth cervical vertebra, closed	0.0106%	0.0339%	0.0269%	0.0096%
753.7	Anomalies of urachus	0.0185%	0.0097%	0.0041%	0.0064%
733.99	Other disorders of bone and cartilage	0.0139%	0.0097%	0.0103%	0.0287%
755.67	Anomalies of foot, not elsewhere classified	0.0194%	0.0000%	0.0041%	0.0064%
723.4	Brachial neuritis or radiculitis NOS	0.0079%	0.0291%	0.0351%	0.0191%
575.10	Cholecystitis, unspecified	0.0134%	0.0097%	0.0124%	0.0287%
610.2	Fibroadenosis of breast	0.0176%	0.0048%	0.0103%	0.0064%
396.8	Multiple involvement of mitral and aortic valves	0.0092%	0.0388%	0.0207%	0.0255%
250.60	Diabetes with neurological manifestations, Type II [non-ins	0.0069%	0.0485%	0.0269%	0.0255%
350.1	Trigeminal neuralgia	0.0074%	0.0339%	0.0351%	0.0191%
170.7	Malignant neoplasm of long bones of lower limb	0.0134%	0.0242%	0.0248%	0.0000%
151.5	Malignant neoplasm of lesser curvature of stomach, unspec	0.0106%	0.0145%	0.0310%	0.0159%
V59.4	Donors of kidney	0.0171%	0.0097%	0.0124%	0.0000%
895.0	Traumatic amputation of toe(s) (complete) (partial) without	0.0176%	0.0000%	0.0124%	0.0032%
942.32	Burn of chest wall, excluding breast and nipple, full-thickn	0.0180%	0.0000%	0.0103%	0.0032%
871.2	Rupture of eye with partial loss of intraocular tissue	0.0190%	0.0000%	0.0062%	0.0032%
821.20	Fracture of unspecified part of lower end of femur, closed	0.0088%	0.0097%	0.0351%	0.0223%
730.28	Unspecified osteomyelitis, other specified sites	0.0074%	0.0388%	0.0207%	0.0350%

ICD-9-CM	ICD-9-CM	PureO	ContiB	NewB	PastB
617.8	Endometriosis of other specified sites	0.0176%	0.0097%	0.0062%	0.0064%
435.9	Unspecified transient cerebral ischemia	0.0069%	0.0388%	0.0124%	0.0510%
487.0	Influenza with pneumonia	0.0097%	0.0339%	0.0207%	0.0223%
482.41	Pneumonia due to Staphylococcus aureus	0.0092%	0.0291%	0.0124%	0.0414%
376.52	Enophthalmos due to trauma or surgery	0.0148%	0.0048%	0.0145%	0.0159%
191.1	Malignant neoplasm of frontal lobe	0.0088%	0.0339%	0.0227%	0.0255%
715.34	Osteoarthrosis, localized, not specified whether primary or	0.0125%	0.0145%	0.0124%	0.0255%
723.7	Ossification of posterior longitudinal ligament in cervical r	0.0097%	0.0388%	0.0248%	0.0096%
537.0	Acquired hypertrophic pyloric stenosis	0.0148%	0.0097%	0.0062%	0.0223%
575.12	Acute and chronic cholecystitis	0.0153%	0.0097%	0.0083%	0.0159%
478.24	Retropharyngeal abscess	0.0134%	0.0097%	0.0248%	0.0032%
440.24	Atherosclerosis of the extremities with gangrene	0.0111%	0.0339%	0.0186%	0.0127%
196.3	Secondary and unspecified malignant neoplasm of lymph n	0.0092%	0.0339%	0.0165%	0.0287%
162.8	Malignant neoplasm of other parts of bronchus or lung	0.0092%	0.0339%	0.0289%	0.0096%
852.20	Subdural hemorrhage following injury without mention of	0.0106%	0.0048%	0.0248%	0.0223%
746.3	Congenital stenosis of aortic valve	0.0111%	0.0388%	0.0145%	0.0127%
628.9	Infertility, female, of unspecified origin	0.0180%	0.0048%	0.0021%	0.0064%
568.0	Peritoneal adhesions(postoperative)(postinfection)	0.0111%	0.0291%	0.0186%	0.0127%
596.0	Bladder neck obstruction	0.0069%	0.0145%	0.0165%	0.0541%
371.00	Corneal opacity, unspecified	0.0171%	0.0000%	0.0062%	0.0096%
518.4	Acute edema of lung, unspecified	0.0069%	0.0291%	0.0186%	0.0414%
394.2	Mitral stenosis with insufficiency	0.0069%	0.0242%	0.0165%	0.0478%
324.0	Intracranial abscess	0.0088%	0.0533%	0.0103%	0.0255%
343.2	Quadriplegic infantile cerebral palsy	0.0153%	0.0048%	0.0083%	0.0159%
198.6	Secondary malignant neoplasm of ovary	0.0102%	0.0242%	0.0248%	0.0127%
171.2	Malignant neoplasm of connective and other soft tissue of	0.0139%	0.0048%	0.0186%	0.0096%
117.3	Aspergillosis	0.0129%	0.0242%	0.0186%	0.0032%
942.33	Burn of abdominal wall, full-thickness skin loss (third degr	0.0153%	0.0000%	0.0165%	0.0032%
945.39	Burn of multiple sites of lower limb(s), full-thickness skin	0.0143%	0.0000%	0.0227%	0.0000%
942.39	Burn of other and multiple sites of trunk, full-thickness ski	0.0134%	0.0097%	0.0186%	0.0064%
733.90	Disorder of bone and cartilage, unspecified	0.0092%	0.0339%	0.0207%	0.0159%
718.46	Contracture of joint, lower leg	0.0162%	0.0097%	0.0041%	0.0096%
719.26	Villonodular synovitis, lower leg	0.0139%	0.0097%	0.0103%	0.0159%
593.2	Cyst of kidney, acquired	0.0148%	0.0145%	0.0041%	0.0159%
542	Other appendicitis	0.0176%	0.0000%	0.0021%	0.0096%
528.8	Oral submucosal fibrosis, including of tongue	0.0079%	0.0097%	0.0475%	0.0000%
550.11	Inguinal hernia, with obstruction, without mention of gangr	0.0166%	0.0048%	0.0041%	0.0096%
608.2	Torsion of testis	0.0185%	0.0048%	0.0021%	0.0000%
520.6	Disturbances in tooth eruption	0.0166%	0.0145%	0.0000%	0.0096%

ICD-9-CM	ICD-9-CM	PureO	ContiB	NewB	PastB
250.50	Diabetes with ophthalmic manifestations, Type II [non-insu	0.0139%	0.0145%	0.0103%	0.0127%
276.8	Hypopotassemia	0.0055%	0.0291%	0.0186%	0.0478%
214.3	Lipoma of intra-abdominal organs	0.0134%	0.0048%	0.0103%	0.0223%
205.10	Chronic myeloid leukemia, without mention of remission	0.0088%	0.0339%	0.0269%	0.0096%
802.22	Fracture of mandible, closed, subcondylar	0.0134%	0.0048%	0.0165%	0.0096%
852.46	Extradural hemorrhage following injury without mention o	0.0106%	0.0145%	0.0269%	0.0064%
718.05	Articular cartilage disorder, pelvic region and thigh	0.0176%	0.0000%	0.0000%	0.0096%
608.89	Other specified disorders of male genital organs	0.0134%	0.0000%	0.0103%	0.0223%
590.2	Renal and perinephric abscess	0.0097%	0.0048%	0.0207%	0.0287%
564.1	Irritable bowel syndrome	0.0134%	0.0145%	0.0124%	0.0096%
380.10	Infective otitis externa, unspecified	0.0143%	0.0048%	0.0083%	0.0159%
496	Chronic airways obstruction, not elsewhere classified	0.0069%	0.0339%	0.0207%	0.0287%
250.22	Diabetes with hyperosmolar coma, Type II [non-insulin dep	0.0092%	0.0242%	0.0124%	0.0318%
202.81	Other lymphomas, lymph nodes of head, face and neck	0.0092%	0.0145%	0.0248%	0.0191%
901.0	Injury to thoracic aorta	0.0111%	0.0000%	0.0331%	0.0000%
824.1	Fracture of medial malleolus of ankle, open	0.0153%	0.0048%	0.0103%	0.0032%
780.4	Dizziness and giddiness	0.0032%	0.0679%	0.0165%	0.0350%
727.83	Plica syndrome	0.0148%	0.0048%	0.0103%	0.0064%
528.6	Leukoplakia of oral mucosa, including tongue	0.0134%	0.0242%	0.0021%	0.0159%
533.90	Peptic ulcer, site unspecified, unspecified as acute or chron	0.0111%	0.0242%	0.0145%	0.0127%
610.8	Other specified benign mammary dysplasias	0.0125%	0.0097%	0.0083%	0.0223%
577.2	Cyst and pseudocyst of pancreas	0.0106%	0.0242%	0.0186%	0.0096%
537.89	Other specified disorders of stomach and duodenum	0.0097%	0.0097%	0.0227%	0.0191%
633.2	Ovarian pregnancy	0.0180%	0.0048%	0.0000%	0.0000%
478.31	Paralysis of vocal cords or larynx, unilateral, partial	0.0116%	0.0194%	0.0083%	0.0223%
212.3	Benign neoplasm of bronchus and lung	0.0134%	0.0242%	0.0083%	0.0064%
171.6	Malignant neoplasm of connective and other soft tissue of p	0.0125%	0.0097%	0.0186%	0.0064%
942.24	Burn of trunk, back (any part), blisters, epidermal loss (sec	0.0143%	0.0000%	0.0103%	0.0096%
V55.2	Attention to ileostomy	0.0125%	0.0000%	0.0083%	0.0255%
813.08	Fracture of radius with ulna, upper end (any part), closed	0.0125%	0.0194%	0.0103%	0.0096%
715.31	Osteoarthrosis, localized, not specified whether primary or	0.0111%	0.0145%	0.0103%	0.0223%
661.31	Precipitate labor, delivered, with or without mention of ant	0.0176%	0.0000%	0.0021%	0.0000%
669.81	Other complications of labor and delivery, not elsewhere cl	0.0162%	0.0000%	0.0000%	0.0127%
647.61	Other viral diseases in the mother classifiable elsewhere, bu	0.0162%	0.0000%	0.0041%	0.0064%
658.23	Delayed delivery after spontaneous or unspecified rupture o	0.0176%	0.0000%	0.0021%	0.0000%
701.4	Keloid scar	0.0162%	0.0097%	0.0021%	0.0032%
574.40	Calculus of bile duct with other cholecystitis without menti	0.0120%	0.0097%	0.0083%	0.0223%
471.8	Other polyp of sinus	0.0166%	0.0048%	0.0021%	0.0032%
378.10	Exotropia, unspecified	0.0153%	0.0097%	0.0021%	0.0096%

ICD-9-CM	ICD-9-CM	PureO	ContiB	NewB	PastB
455.1	Internal thrombosed hemorrhoids	0.0139%	0.0048%	0.0083%	0.0127%
411.1	Intermediate coronary syndrome	0.0055%	0.0485%	0.0145%	0.0318%
160.0	Malignant neoplasm of nasal cavities	0.0111%	0.0000%	0.0227%	0.0127%
011.96	Pulmonary tuberculosis, unspecified, tubercle bacilli not fo	0.0106%	0.0097%	0.0145%	0.0223%
928.21	Crushing injury of ankle	0.0148%	0.0000%	0.0103%	0.0032%
812.31	Fracture of shaft of humerus, open	0.0116%	0.0048%	0.0207%	0.0064%
813.43	Fracture of distal end of ulna (alone), closed	0.0111%	0.0048%	0.0145%	0.0191%
851.81	Other and unspecified cerebral laceration and contusion wi	0.0092%	0.0242%	0.0186%	0.0127%
842.09	Sprains and strains of other specified site, wrist	0.0139%	0.0000%	0.0103%	0.0096%
733.49	Aseptic necrosis of other bone	0.0102%	0.0194%	0.0207%	0.0064%
747.81	Anomalies of cerebrovascular system	0.0102%	0.0242%	0.0145%	0.0127%
648.61	Other cardiovascular diseases in the mother classifiable els	0.0157%	0.0048%	0.0021%	0.0064%
524.03	Maxillary hypoplasia	0.0166%	0.0000%	0.0000%	0.0064%
564.0	Constipation	0.0074%	0.0388%	0.0145%	0.0223%
593.4	Other ureteric obstruction	0.0129%	0.0194%	0.0062%	0.0096%
526.2	Other cysts of jaws	0.0139%	0.0242%	0.0021%	0.0064%
552.1	Umbilical hernia, with obstruction	0.0129%	0.0000%	0.0083%	0.0191%
595.9	Cystitis, unspecified	0.0088%	0.0194%	0.0145%	0.0255%
354.1	Other lesion of median nerve	0.0097%	0.0194%	0.0145%	0.0191%
145.8	Malignant neoplasm of other specified parts of mouth	0.0079%	0.0145%	0.0310%	0.0096%
152.9	Malignant neoplasm of small intestine, unspecified	0.0143%	0.0048%	0.0062%	0.0096%
070.32	Viral hepatitis B without mention of hepatic coma, chronic	0.0092%	0.0291%	0.0165%	0.0127%
943.39	Burn of multiple sites of upper limb, except wrist and hand	0.0143%	0.0048%	0.0103%	0.0000%
807.07	Fracture of seven ribs, closed	0.0092%	0.0097%	0.0269%	0.0064%
786.59	Other chest pain	0.0037%	0.0291%	0.0310%	0.0255%
752.2	Doubling of uterus	0.0162%	0.0000%	0.0000%	0.0064%
788.30	Urinary incontinence, unspecified	0.0106%	0.0097%	0.0103%	0.0223%
736.29	Other acquired deformities of finger	0.0116%	0.0291%	0.0083%	0.0064%
736.79	Other acquired deformities of ankle and foot	0.0134%	0.0048%	0.0124%	0.0032%
782.2	Localized superficial swelling, mass, or lump	0.0116%	0.0000%	0.0062%	0.0287%
726.33	Olecranon bursitis	0.0097%	0.0242%	0.0124%	0.0159%
669.51	Forceps or vacuum extractor delivery without mention of ir	0.0157%	0.0000%	0.0021%	0.0064%
535.40	Other specified gastritis, without mention of hemorrhage	0.0106%	0.0242%	0.0145%	0.0064%
568.89	Other specified disorders of peritoneum	0.0125%	0.0048%	0.0165%	0.0032%
474.10	Hypertrophy of tonsils with adenoids	0.0171%	0.0000%	0.0000%	0.0000%
199.1	Malignant neoplasm of unspecified site (primary) (seconda	0.0055%	0.0339%	0.0289%	0.0127%
952.05	C5-C7 level with unspecified spinal cord injury	0.0083%	0.0194%	0.0227%	0.0096%
998.2	Accidental puncture or laceration during a procedure	0.0097%	0.0194%	0.0103%	0.0191%
867.0	Injury to bladder and urethra, without metntion of open wo	0.0134%	0.0000%	0.0124%	0.0032%

ICD-9-CM	ICD-9-CM	PureO	ContiB	NewB	PastB
839.01	Dislocations of first cervical vertebra, closed	0.0088%	0.0097%	0.0124%	0.0287%
800.21	Fracture of vault of skull, closed with subarachnoid, subdu	0.0111%	0.0097%	0.0145%	0.0096%
767.6	Injury to brachial plexus	0.0153%	0.0048%	0.0041%	0.0000%
724.2	Lumbago	0.0069%	0.0242%	0.0207%	0.0191%
662.11	Prolonged labor, unspecified, delivered, with or without me	0.0162%	0.0000%	0.0000%	0.0032%
727.05	Other tenosynovitis of hand and wrist	0.0106%	0.0145%	0.0103%	0.0159%
662.01	Prolonged first stage, delivered, with or without mention of	0.0148%	0.0000%	0.0000%	0.0127%
552.00	Femoral hernia with obstruction, unilateral or unspecified (	0.0129%	0.0048%	0.0083%	0.0096%
530.2	Ulcer of esophagus	0.0092%	0.0339%	0.0103%	0.0127%
474.8	Other chronic disease of tonsils and adenoids	0.0139%	0.0000%	0.0062%	0.0096%
284.9	Aplastic anemia, unspecified	0.0055%	0.0291%	0.0310%	0.0096%
198.4	Secondary malignant neoplasm of other parts of nervous sy	0.0055%	0.0339%	0.0248%	0.0159%
197.8	Secondary malignant neoplasm of other digestive organs ar	0.0069%	0.0242%	0.0186%	0.0223%
173.6	Malignant neoplasm of skin of upper limb, including shoul	0.0092%	0.0145%	0.0227%	0.0064%
998.12	Hematoma complicating a procedure	0.0092%	0.0242%	0.0124%	0.0127%
997.69	Other late amputation stump complication	0.0079%	0.0291%	0.0207%	0.0064%
751.0	Meckel's diverticulum	0.0148%	0.0048%	0.0000%	0.0064%
749.04	Incomplete cleft palate, bilateral	0.0157%	0.0000%	0.0021%	0.0000%
788.33	Mixed incontinence (male)(female)	0.0102%	0.0194%	0.0021%	0.0255%
717.6	Loose body in knee	0.0125%	0.0097%	0.0021%	0.0159%
720.0	Ankylosing spondylitis	0.0106%	0.0048%	0.0103%	0.0191%
530.0	Achalasia and cardiospasm	0.0125%	0.0145%	0.0041%	0.0096%
596.8	Other specified disorders of bladder	0.0079%	0.0291%	0.0103%	0.0223%
556.9	Ulcerative colitis, unspecified	0.0092%	0.0242%	0.0103%	0.0159%
594.2	Calculus in urethra	0.0125%	0.0048%	0.0041%	0.0159%
288.8	Other specified disease of white blood cells	0.0032%	0.0339%	0.0248%	0.0287%
053.19	Herpes zoster with other nervous system complications	0.0069%	0.0339%	0.0207%	0.0096%
163.9	Malignant neoplasm of pleura, unspecified	0.0065%	0.0097%	0.0351%	0.0064%
152.1	Malignant neoplasm of jejunum	0.0092%	0.0097%	0.0227%	0.0064%
959.7	Injury of knee, leg, ankle and foot	0.0088%	0.0097%	0.0269%	0.0000%
996.69	Infection and inflammatory reaction due to other internal p	0.0111%	0.0097%	0.0124%	0.0064%
943.30	Burn of unspecified site of upper limb, full-thickness skin l	0.0134%	0.0000%	0.0083%	0.0032%
823.12	Fracture of upper end of tibia and fibula, open	0.0088%	0.0048%	0.0248%	0.0064%
852.00	Subarachnoid hemorrhage following injury without mentio	0.0097%	0.0097%	0.0227%	0.0000%
802.1	Fracture of nasal bones, open	0.0129%	0.0000%	0.0041%	0.0127%
836.3	Dislocation of patella, closed	0.0129%	0.0145%	0.0062%	0.0000%
789.06	Abdominal pain, epigastric	0.0092%	0.0097%	0.0186%	0.0096%
737.10	Kyphosis (acquired) (postural)	0.0079%	0.0194%	0.0186%	0.0127%
644.03	Threatened premature labor, antepartum condition or comp	0.0143%	0.0000%	0.0041%	0.0032%

ICD-9-CM	ICD-9-CM	PureO	ContiB	NewB	PastB
617.6	Endometriosis in scar of skin	0.0148%	0.0048%	0.0021%	0.0000%
245.2	Chronic lymphocytic thyroiditis	0.0120%	0.0097%	0.0021%	0.0159%
357.0	Acute infective polyneuritis	0.0046%	0.0485%	0.0269%	0.0032%
212.7	Benign neoplasm of heart	0.0074%	0.0097%	0.0248%	0.0127%
070.30	Viral hepatitis B without mention of hepatic coma, acute or	0.0111%	0.0097%	0.0103%	0.0096%
061	Dengue	0.0083%	0.0000%	0.0062%	0.0414%
153.5	Malignant neoplasm of appendix	0.0079%	0.0194%	0.0227%	0.0064%
943.21	Burn of forearm, blisters, epidermal loss (second degree)	0.0125%	0.0000%	0.0083%	0.0064%
996.81	Complications of transplanted kidney	0.0046%	0.0436%	0.0021%	0.0414%
879.2	Open wound of abdominal wall, anterior, without mention	0.0097%	0.0097%	0.0186%	0.0032%
944.30	Burn of unspecified site of hand(s), full-thickness skin loss	0.0116%	0.0048%	0.0103%	0.0064%
839.03	Dislocations of third cervical vertebra, closed	0.0074%	0.0145%	0.0248%	0.0064%
732.4	Juvenile osteochondrosis of lower extremity, excluding foo	0.0111%	0.0048%	0.0103%	0.0096%
730.25	Unspecified osteomyelitis, pelvic region and thigh	0.0102%	0.0242%	0.0124%	0.0000%
726.91	Exostosis of unspecified site	0.0111%	0.0000%	0.0062%	0.0191%
527.3	Abscess of salivary glands	0.0092%	0.0097%	0.0165%	0.0096%
362.54	Macular cyst, hole, or pseudohole	0.0111%	0.0000%	0.0083%	0.0159%
362.56	Macular puckering	0.0120%	0.0097%	0.0041%	0.0096%
255.8	Other specified disorder of adrenal glands	0.0120%	0.0000%	0.0103%	0.0064%
142.1	Malignant neoplasm of submandibular gland	0.0116%	0.0000%	0.0103%	0.0096%
158.9	Malignant neoplasm of peritoneum, unspecified	0.0042%	0.0388%	0.0227%	0.0159%
188.8	Malignant neoplasm of other specified sites of bladder	0.0083%	0.0097%	0.0165%	0.0159%
996.59	Mechanical complication due to other implant and internal	0.0111%	0.0145%	0.0000%	0.0159%
928.3	Crushing injury of toe(s)	0.0111%	0.0048%	0.0145%	0.0000%
852.41	Extradural hemorrhage following injury without mention of	0.0106%	0.0048%	0.0124%	0.0064%
807.08	Fracture of eight or more ribs, closed	0.0106%	0.0000%	0.0145%	0.0064%
871.7	Unspecified ocular penetration	0.0148%	0.0000%	0.0000%	0.0000%
754.2	Certain congenital musculoskeletal deformities of spine	0.0139%	0.0000%	0.0021%	0.0032%
724.8	Other symptoms referable to back	0.0065%	0.0339%	0.0165%	0.0096%
719.56	Stiffness of joint, not elsewhere classified, lower leg	0.0097%	0.0145%	0.0103%	0.0096%
726.65	Prepatellar bursitis	0.0097%	0.0097%	0.0062%	0.0191%
660.11	Obstruction by bony pelvis, delivered, with or without men	0.0129%	0.0048%	0.0021%	0.0064%
574.80	Calculus of gallbladder and bile duct with acute or chronic	0.0102%	0.0242%	0.0041%	0.0096%
474.12	Hepertrophy of adenoids alone	0.0148%	0.0000%	0.0000%	0.0000%
473.3	Chronic sinusitis of sphenoidal	0.0097%	0.0048%	0.0103%	0.0159%
346.90	Migraine, unspecified，without mention of intractable mig	0.0023%	0.0291%	0.0227%	0.0318%
198.1	Secondary malignant neoplasm of other urinary organs	0.0079%	0.0242%	0.0145%	0.0096%
883.1	Open wound of finger(s),complicated	0.0129%	0.0048%	0.0021%	0.0032%
863.29	Injury to other small intestine,without mention of open wo	0.0116%	0.0000%	0.0083%	0.0064%

ICD-9-CM	ICD-9-CM	PureO	ContiB	NewB	PastB
873.40	Open wound to unspecified site of face, without mention o	0.0102%	0.0145%	0.0103%	0.0032%
871.5	Penetration of eyeball with magnetic foreign body	0.0134%	0.0000%	0.0041%	0.0000%
873.42	Open wound of forehead, without mention of complication	0.0106%	0.0048%	0.0083%	0.0096%
813.83	Fracture in unspecified part of radius with ulna, closed	0.0125%	0.0000%	0.0021%	0.0096%
801.24	Fracture of base of skull, closed with subarachnoid, subdur	0.0088%	0.0000%	0.0207%	0.0064%
736.71	Acquired equinovarus deformity	0.0125%	0.0048%	0.0041%	0.0032%
736.6	Other acquired deformities of knee	0.0088%	0.0145%	0.0145%	0.0064%
756.0	Anomalies of skull and face bones	0.0139%	0.0048%	0.0000%	0.0000%
726.11	Calcifying tendinitis of shoulder	0.0074%	0.0194%	0.0103%	0.0191%
614.4	Chronic or unspecified parametritis and pelvic cellulitis	0.0088%	0.0097%	0.0124%	0.0127%
526.4	Inflammatory conditions	0.0074%	0.0242%	0.0062%	0.0223%
572.8	Other sequelae of chronic liver disease	0.0083%	0.0194%	0.0145%	0.0064%
213.5	Benign neoplasm of short bones of upper limb	0.0134%	0.0000%	0.0021%	0.0032%
250.10	Diabetes with ketoacidosis, Type II [non-insulin dependent	0.0074%	0.0194%	0.0103%	0.0191%
213.0	Benign neoplasm of bones of skull and face	0.0111%	0.0048%	0.0062%	0.0096%
197.5	Secondary malignant neoplasm of large intestine and rectur	0.0051%	0.0097%	0.0289%	0.0127%
194.0	Malignant neoplasm of adrenal gland	0.0111%	0.0097%	0.0103%	0.0000%
V56.2	Fitting and adjustment of peritoneal dialysis catheter	0.0060%	0.0145%	0.0124%	0.0255%
945.26	Burn of thigh (any part), blisters, epidermal loss (second de	0.0097%	0.0000%	0.0165%	0.0032%
V53.32	Fitting and adjustment of automatic implantable cardiac de	0.0079%	0.0145%	0.0000%	0.0318%
945.30	Burn of unspecified site of lower limb (leg), full-thickness	0.0079%	0.0097%	0.0186%	0.0064%
843.8	Sprains and strains of other specified sites of hip and thigh	0.0111%	0.0097%	0.0000%	0.0127%
812.51	Supracondylar fracture of humerus, open	0.0079%	0.0145%	0.0103%	0.0159%
868.03	Injury to peritoneum without mention of open wound into c	0.0088%	0.0194%	0.0103%	0.0064%
851.86	Other and unspecified cerebral laceration and contusion wi	0.0088%	0.0242%	0.0103%	0.0032%
813.02	Fracture of coronoid process of ulna, closed	0.0097%	0.0048%	0.0062%	0.0159%
805.01	Fracture of first cervical vertebra, closed	0.0051%	0.0291%	0.0248%	0.0032%
806.00	C1-C4 level fracture with unspecified spinal cord injury, cl	0.0037%	0.0436%	0.0269%	0.0000%
711.01	Pyogenic arthritis, shoulder region	0.0079%	0.0194%	0.0103%	0.0127%
683	Acute lymphadenitis	0.0116%	0.0097%	0.0062%	0.0000%
722.4	Degeneration of cervical intervertebral disc	0.0046%	0.0194%	0.0207%	0.0191%
654.53	Cervical incompetence, antepartum condition or complicati	0.0139%	0.0000%	0.0000%	0.0000%
553.3	Diaphragmatic hernia	0.0083%	0.0145%	0.0041%	0.0223%
427.69	Other premature beats	0.0083%	0.0145%	0.0062%	0.0191%
487.1	Influenza with other respiratory manifestations	0.0069%	0.0194%	0.0083%	0.0223%
455.4	External thrombosed hemorrhoids	0.0111%	0.0000%	0.0103%	0.0032%
492.0	Emphysematous bleb	0.0097%	0.0145%	0.0083%	0.0064%
246.2	Cyst of thyroid	0.0111%	0.0048%	0.0041%	0.0096%
173.2	Malignant neoplasm of skin of ear and external auditory ca	0.0097%	0.0291%	0.0062%	0.0000%

ICD-9-CM	ICD-9-CM	PureO	ContiB	NewB	PastB
874.8	Open wound to other and unspecified parts of neck, withou	0.0079%	0.0097%	0.0165%	0.0064%
947.1	Burn of larynx, trachea and lung	0.0102%	0.0000%	0.0124%	0.0032%
873.0	Other open wound of scalp, without mention of complicatio	0.0083%	0.0097%	0.0124%	0.0096%
816.02	Fracture of distal phalanx or phalanges, closed	0.0120%	0.0048%	0.0041%	0.0000%
805.06	Fracture of sixth cervical vertebra, closed	0.0074%	0.0097%	0.0207%	0.0032%
852.25	Subdural hemorrhage following injury without mention of	0.0060%	0.0000%	0.0207%	0.0191%
839.20	Dislocations of lumbar vertebra, closed	0.0065%	0.0145%	0.0248%	0.0000%
749.25	Cleft palate with cleft lip, other combination	0.0129%	0.0000%	0.0000%	0.0032%
751.3	Hirschsprung's disease and other congenital functional diso	0.0134%	0.0000%	0.0000%	0.0000%
646.81	Other specified complications of pregnancy, delivered, with	0.0120%	0.0048%	0.0000%	0.0064%
569.82	Ulceration of intestine	0.0088%	0.0194%	0.0083%	0.0064%
397.0	Diseases of tricuspid valve	0.0074%	0.0194%	0.0083%	0.0159%
478.33	Paralysis of vocal cords of larynx, bilateral, partial	0.0074%	0.0145%	0.0103%	0.0159%
435.3	Vertebrobasilar artery syndrome	0.0051%	0.0194%	0.0124%	0.0255%
371.23	Bullous keratopathy	0.0102%	0.0048%	0.0041%	0.0127%
401.9	Essential hypertension, unspecified	0.0046%	0.0145%	0.0227%	0.0159%
250.20	Diabetes with hyperosmolar coma, Type II [non-insulin dep	0.0060%	0.0097%	0.0124%	0.0255%
213.4	Benign neoplasm of scapula and long bones of upper limb	0.0125%	0.0000%	0.0000%	0.0064%
254.8	Other specified diseases of thymus gland	0.0079%	0.0242%	0.0021%	0.0191%
205.01	Acute myeloid leukemia, in remission	0.0042%	0.0388%	0.0165%	0.0127%
173.4	Malignant neoplasm of scalp and skin of neck	0.0097%	0.0048%	0.0083%	0.0096%
192.2	Malignant neoplasm of spinal cord	0.0055%	0.0291%	0.0207%	0.0032%
141.8	Malignant neoplasm of other sites of tongue	0.0042%	0.0145%	0.0310%	0.0064%
924.10	Contusion of lower leg	0.0097%	0.0048%	0.0041%	0.0127%
942.22	Burn of trunk, chest wall, excluding breast and nipple, blist	0.0116%	0.0048%	0.0041%	0.0000%
805.05	Fracture of fifth cervical vertebra, closed	0.0092%	0.0097%	0.0103%	0.0032%
749.12	Incomplete cleft lip, unilateral	0.0129%	0.0000%	0.0000%	0.0000%
786.50	Chest pain, unspecified	0.0051%	0.0388%	0.0124%	0.0096%
641.91	Unspecified antepartum hemorrhage, delivered, with or wit	0.0120%	0.0000%	0.0021%	0.0032%
581.9	Nephrotic syndrome with unspecified pathological lesion ir	0.0055%	0.0242%	0.0165%	0.0096%
524.12	Other jaw asymmetry	0.0120%	0.0000%	0.0041%	0.0000%
569.89	Other specified disorders of intestine	0.0083%	0.0048%	0.0165%	0.0032%
477.9	Allergic rhinitis cause unspecified	0.0116%	0.0000%	0.0000%	0.0096%
336.9	Unspecified diseases of spinal cord	0.0046%	0.0194%	0.0248%	0.0064%
282.4	Thalassemias	0.0037%	0.0097%	0.0289%	0.0127%
198.2	Secondary malignant neoplasm of skin	0.0046%	0.0097%	0.0227%	0.0159%
188.4	Malignant neoplasm of posterior wall of urinary bladder	0.0079%	0.0048%	0.0145%	0.0096%
140.0	Malignant neoplasm of upper lip, vermilion border	0.0060%	0.0000%	0.0289%	0.0032%
996.54	Mechanical complication due to breast prosthesis	0.0083%	0.0048%	0.0083%	0.0127%

ICD-9-CM	ICD-9-CM	PureO	ContiB	NewB	PastB
920	Contusion of face, scalp, and neck except eye(s)	0.0102%	0.0048%	0.0062%	0.0032%
958.8	Other early complications of trauma	0.0069%	0.0048%	0.0165%	0.0096%
821.30	Fracture of unspecified part of lower end of femur, open	0.0069%	0.0145%	0.0186%	0.0000%
808.43	Multiple pelvic fractures with disruption of pelvic circle, cl	0.0069%	0.0048%	0.0207%	0.0032%
839.05	Dislocations of fifth cervical vertebra, closed	0.0051%	0.0145%	0.0186%	0.0127%
737.39	Other kyphoscoliosis and scoliosis	0.0065%	0.0097%	0.0207%	0.0032%
734	Flat foot	0.0106%	0.0048%	0.0041%	0.0032%
786.3	Hemoptysis	0.0032%	0.0242%	0.0145%	0.0255%
655.11	Chromosomal abnormality in fetus, affecting management	0.0116%	0.0000%	0.0021%	0.0032%
574.21	Calculus of gallbladder with obstruction without mention o	0.0111%	0.0097%	0.0000%	0.0032%
555.9	Regional enteritis, unspecified site	0.0097%	0.0000%	0.0083%	0.0064%
569.69	Other complication of colostomy and enterostomy	0.0055%	0.0194%	0.0145%	0.0127%
599.2	Urethral diverticulum	0.0102%	0.0145%	0.0021%	0.0032%
575.8	Other specified disorders of gallbladder	0.0088%	0.0000%	0.0041%	0.0191%
530.3	Stricture and stenosis of esophagus	0.0055%	0.0145%	0.0124%	0.0191%
396.2	Mitral valve insufficiency and aortic valve stenosis	0.0042%	0.0194%	0.0165%	0.0191%
462	Acute pharyngitis	0.0032%	0.0242%	0.0269%	0.0064%
518.82	Other pulmonary insufficiency, not elsewhere classified	0.0055%	0.0000%	0.0124%	0.0287%
323.9	Unspecified cause of encephalitis	0.0060%	0.0145%	0.0145%	0.0127%
011.94	Pulmonary tuberculosis, unspecified, tubercle bacilli not fo	0.0055%	0.0145%	0.0145%	0.0159%
202.10	Mycosis fungoides, unspecified site, extranodal solid organ	0.0032%	0.0339%	0.0186%	0.0127%
191.8	Malignant neoplasm of other parts of brain	0.0069%	0.0194%	0.0124%	0.0064%
997.62	Late amputation stump infection (chronic)	0.0079%	0.0048%	0.0103%	0.0096%
921.3	Contusion of eyeball	0.0102%	0.0000%	0.0062%	0.0032%
945.22	Burn of foot, blisters, epidermal loss (second degree)	0.0092%	0.0048%	0.0021%	0.0127%
952.04	T1-T4 level with other specified spinal cord injury	0.0023%	0.0291%	0.0248%	0.0096%
813.32	Fracture of shaft of ulna (alone), open	0.0102%	0.0048%	0.0041%	0.0032%
813.04	Other and unspecified fractures of proximal end of ulna (al	0.0097%	0.0048%	0.0041%	0.0064%
825.21	Fracture of astragalus, closed	0.0097%	0.0000%	0.0083%	0.0032%
780.8	Hyperhidrosis	0.0106%	0.0048%	0.0021%	0.0032%
751.62	Congenital cystic disease of liver	0.0088%	0.0097%	0.0062%	0.0064%
728.85	Spasm of muscle	0.0102%	0.0000%	0.0041%	0.0064%
785.59	Other shock without mention of trauma	0.0060%	0.0097%	0.0145%	0.0127%
718.86	Other joint derangement, not elsewhere classified, lower le	0.0088%	0.0048%	0.0062%	0.0096%
718.36	Recurrent dislocation of joint, lower leg	0.0102%	0.0048%	0.0062%	0.0000%
723.5	Torticollis, unspecified	0.0116%	0.0000%	0.0000%	0.0032%
719.52	Stiffness of joint, not elsewhere classified, upper arm	0.0097%	0.0000%	0.0103%	0.0000%
619.0	Urinary-genital tract fistula, female	0.0065%	0.0097%	0.0124%	0.0127%
511.0	Pleurisy, without mention of effusion or current tuberculosi	0.0083%	0.0097%	0.0083%	0.0064%

ICD-9-CM	ICD-9-CM	PureO	ContiB	NewB	PastB
230.0	Carcinoma in situ of lip, oral cavity and pharynx	0.0069%	0.0145%	0.0062%	0.0159%
180.0	Malignant neoplasm of endocervix	0.0042%	0.0339%	0.0165%	0.0064%
154.3	Malignant neoplasm of anus, unspecified	0.0042%	0.0097%	0.0227%	0.0127%
003.1	Salmonella septicemia	0.0065%	0.0194%	0.0083%	0.0127%
152.2	Malignant neoplasm of ileum	0.0083%	0.0000%	0.0145%	0.0032%
171.4	Malignant neoplasm of connective and other soft tissue of t	0.0074%	0.0145%	0.0083%	0.0096%
162.2	Malignant neoplasm of main bronchus	0.0055%	0.0145%	0.0145%	0.0127%
160.2	Malignant neoplasm of maxillary sinus	0.0046%	0.0048%	0.0269%	0.0064%
996.79	Other complications due to other internal prosthetic device,	0.0074%	0.0097%	0.0103%	0.0064%
812.49	Other fracture of of lower end of humerus, closed	0.0097%	0.0000%	0.0062%	0.0032%
780.79	Other malaise and fatigue	0.0023%	0.0291%	0.0124%	0.0255%
727.63	Rupture of extensor tendons of hand and wrist	0.0097%	0.0000%	0.0041%	0.0064%
727.02	Giant cell tumor of tendon sheath	0.0102%	0.0048%	0.0000%	0.0064%
552.8	Hernia of other specified sites, with obstruction	0.0083%	0.0048%	0.0041%	0.0127%
533.40	Peptic ulcer, site unspecified, chronic or unspecified with h	0.0079%	0.0048%	0.0062%	0.0127%
574.31	Calculus of bile duct with acute cholecystitis, with obstruct	0.0079%	0.0048%	0.0062%	0.0127%
562.01	Diverticulitis of small intestine (without mention of hemorr	0.0083%	0.0097%	0.0083%	0.0032%
569.2	Stenosis of rectum and anus	0.0083%	0.0048%	0.0041%	0.0127%
529.0	Glossitis	0.0083%	0.0097%	0.0083%	0.0032%
599.1	Urethral fistula	0.0116%	0.0000%	0.0000%	0.0000%
425.4	Other primary cardiomyopathies	0.0060%	0.0145%	0.0103%	0.0127%
454.1	Varicose veins of lower extremities with inflammation	0.0102%	0.0000%	0.0000%	0.0096%
320.9	Meningitis due to unspecified bacterium	0.0069%	0.0194%	0.0103%	0.0032%
345.90	Unspecified epilepsy without mention of intractable epileps	0.0032%	0.0339%	0.0103%	0.0191%
280.9	Iron deficiency anemia, unspecified	0.0065%	0.0291%	0.0062%	0.0064%
287.5	Thrombocytopenia, unspecified	0.0060%	0.0242%	0.0041%	0.0159%
191.2	Malignant neoplasm of temporal lobe	0.0051%	0.0194%	0.0145%	0.0096%
945.29	Burn of multiple sites of lower limb(s), blisters, epidermal	0.0079%	0.0000%	0.0145%	0.0000%
996.72	Other complications due to other cardiac device, implant, a	0.0069%	0.0145%	0.0103%	0.0032%
959.5	Injury of finger	0.0079%	0.0097%	0.0083%	0.0032%
986	Toxic effect of carbon monoxide	0.0042%	0.0194%	0.0186%	0.0064%
996.39	Mechanical complication of other genitourinary device, imp	0.0079%	0.0000%	0.0062%	0.0127%
807.2	Fracture of sternum, closed	0.0074%	0.0048%	0.0083%	0.0096%
871.1	Ocular laceration with prolapse or exposure of intraocular t	0.0097%	0.0000%	0.0041%	0.0032%
802.38	Fracture of mandible, open, body, other and unspecified	0.0092%	0.0048%	0.0062%	0.0000%
868.00	Injury to unspecified intra-abdominal organs without menti	0.0055%	0.0048%	0.0165%	0.0096%
801.01	Fracture of base of skull, closed without mention of intracr	0.0097%	0.0000%	0.0041%	0.0032%
784.7	Epistaxis	0.0065%	0.0145%	0.0083%	0.0096%
744.89	Other specified anomalies of face and neck	0.0106%	0.0000%	0.0021%	0.0000%

ICD-9-CM	ICD-9-CM	PureO	ContiB	NewB	PastB
729.89	Other musculoskeletal symptoms referable to limbs	0.0060%	0.0194%	0.0103%	0.0064%
749.24	Incomplete cleft palate with cleft lip, bilateral	0.0102%	0.0000%	0.0021%	0.0032%
685.1	Pilonidal cyst, without mention of abscess	0.0083%	0.0000%	0.0103%	0.0032%
651.11	Triplet pregnancy, delivered, with or without mention of ar	0.0111%	0.0000%	0.0000%	0.0000%
568.81	Hemoperitoneum (nontraumatic)	0.0055%	0.0145%	0.0145%	0.0064%
607.89	Other disorders of penis	0.0074%	0.0097%	0.0041%	0.0127%
569.81	Fistula of intestine, excluding rectum and anus	0.0074%	0.0000%	0.0145%	0.0032%
569.0	Anal and rectal polyp	0.0092%	0.0048%	0.0021%	0.0064%
615.9	Unspecified inflammatory disease of uterus	0.0055%	0.0145%	0.0062%	0.0191%
577.8	Other specified diseases of pancreas	0.0074%	0.0097%	0.0103%	0.0032%
620.0	Follicular cyst of ovary	0.0097%	0.0000%	0.0021%	0.0064%
578.1	Blood in stool	0.0069%	0.0097%	0.0021%	0.0191%
642.01	Benign essential hypertension complicating pregnancy, chil	0.0102%	0.0048%	0.0000%	0.0032%
396.0	Mitral valve stenosis and aortic valve stenosis	0.0055%	0.0097%	0.0062%	0.0223%
441.1	Thoracic aneurysm, ruptured	0.0074%	0.0048%	0.0062%	0.0127%
380.21	Cholesteatoma of external ear	0.0092%	0.0048%	0.0041%	0.0032%
233.3	Carcinoma in situ of other and unspecified female genital o	0.0074%	0.0000%	0.0145%	0.0032%
237.71	Neurofibromatosis, Type Ⅰ〔von Recklinghausen's diseas	0.0092%	0.0048%	0.0062%	0.0000%
227.1	Benign neoplasm of parathyroid gland	0.0083%	0.0000%	0.0041%	0.0127%
237.70	Neurofibromatosis, unspecified	0.0088%	0.0097%	0.0041%	0.0032%
225.1	Benign neoplasm of cranial nerves	0.0074%	0.0048%	0.0103%	0.0064%
153.8	Malignant neoplasm of other specified sites of large intesti	0.0060%	0.0048%	0.0124%	0.0127%
998.89	Other specified complications	0.0060%	0.0242%	0.0021%	0.0127%
996.56	Mechanical complication due to peritoneal dialysis catheter	0.0051%	0.0145%	0.0041%	0.0223%
942.23	Burn of trunk, abdominal wall, blisters, epidermal loss (sec	0.0097%	0.0000%	0.0021%	0.0032%
996.51	Mechanical complication due to corneal graft	0.0097%	0.0048%	0.0000%	0.0032%
996.53	Mechanical complication due to ocular lens prosthesis	0.0083%	0.0000%	0.0021%	0.0127%
860.5	Pneumohemothorax with open wound into thorax	0.0074%	0.0000%	0.0124%	0.0032%
871.6	Penetration of eyeball with (nonmagnetic) foreign body	0.0088%	0.0000%	0.0083%	0.0000%
801.06	Fracture of base of skull, closed without mention of intracr	0.0074%	0.0000%	0.0124%	0.0032%
753.12	Polycystic kidney, unspecified type	0.0060%	0.0097%	0.0083%	0.0127%
733.29	Other cyst of bone	0.0088%	0.0048%	0.0021%	0.0064%
715.27	Osteoarthrosis, localized, secondary, ankle and foot	0.0097%	0.0048%	0.0000%	0.0032%
652.91	Unspecified malposition or malpresentation, delivered, with	0.0106%	0.0000%	0.0000%	0.0000%
680.2	Carbuncle and furuncle, trunk	0.0060%	0.0097%	0.0083%	0.0127%
646.83	Other specified complications of pregnancy, antepartum co	0.0097%	0.0000%	0.0021%	0.0032%
560.39	Other impaction of intestine	0.0042%	0.0242%	0.0083%	0.0159%
604.0	Orchitis, epididymitis and epididymo-orchitis, with abscess	0.0060%	0.0097%	0.0083%	0.0127%
618.9	Unspecified genital prolapse	0.0042%	0.0048%	0.0062%	0.0318%

ICD-9-CM	ICD-9-CM	PureO	ContiB	NewB	PastB
386.10	Peripheral vertigo, unspecified	0.0018%	0.0339%	0.0062%	0.0287%
442.2	Aneurysm of iliac artery	0.0069%	0.0145%	0.0062%	0.0064%
422.90	Acute myocarditis, unspecified	0.0074%	0.0048%	0.0083%	0.0064%
454.0	Varicose veins of lower extremities with ulcer	0.0097%	0.0000%	0.0021%	0.0032%
427.41	Ventricular fibrillation	0.0042%	0.0242%	0.0124%	0.0096%
235.4	Neoplasm of uncertain behavior of retroperitoneum and pe	0.0083%	0.0048%	0.0041%	0.0064%
287.0	Allergic purpura	0.0083%	0.0048%	0.0041%	0.0064%
322.9	Meningitis, unspecified	0.0069%	0.0194%	0.0041%	0.0064%
340	Multiple sclerosis	0.0023%	0.0194%	0.0103%	0.0287%
171.0	Malignant neoplasm of connective and other soft tissue of	0.0042%	0.0097%	0.0165%	0.0127%
187.4	Malignant neoplasm of penis, part unspecified	0.0055%	0.0000%	0.0165%	0.0096%
998.3	Disruption of operation wound	0.0069%	0.0000%	0.0103%	0.0064%
928.9	Crushing injury of lower limb, unspecified site	0.0069%	0.0097%	0.0103%	0.0000%
916.0	Superficial injury of hip, thigh, leg and ankle, abrasion or f	0.0092%	0.0000%	0.0000%	0.0064%
996.82	Complications of transplanted liver	0.0055%	0.0145%	0.0041%	0.0159%
903.3	Injury to ulnar blood vessels	0.0083%	0.0000%	0.0083%	0.0000%
928.00	Crushing injury of thigh	0.0060%	0.0000%	0.0124%	0.0096%
824.7	Fracture of trimalleolar of ankle, open	0.0065%	0.0048%	0.0145%	0.0000%
865.00	Unspecified injury to spleen, without mention of open wou	0.0074%	0.0000%	0.0124%	0.0000%
811.00	Fracture in unspecified part of scapula, closed	0.0065%	0.0097%	0.0103%	0.0032%
850.5	Concussion with loss of consciousness of unspecified durat	0.0083%	0.0097%	0.0041%	0.0000%
736.89	Other acquired deformities of other parts of limbs	0.0083%	0.0000%	0.0021%	0.0096%
789.5	Ascites	0.0051%	0.0242%	0.0103%	0.0032%
648.11	Thyroid dysfunction conditions in the mother classifiable e	0.0097%	0.0000%	0.0000%	0.0032%
694.4	Pemphigus	0.0046%	0.0194%	0.0124%	0.0064%
727.00	Synovitis and tenosynovitis, unspecified	0.0079%	0.0048%	0.0041%	0.0064%
718.83	Other joint derangement, not elsewhere classified, forearm	0.0079%	0.0048%	0.0021%	0.0096%
652.81	Other specified malposition or malpresentation, delivered,	0.0102%	0.0000%	0.0000%	0.0000%
707.19	Ulcer of other part of lower limb	0.0055%	0.0097%	0.0041%	0.0191%
574.11	Calculus of gallbladder with other cholecystitis with obstru	0.0074%	0.0048%	0.0021%	0.0127%
571.1	Acute alcoholic hepatitis	0.0037%	0.0194%	0.0145%	0.0096%
441.02	Dissection of aorta , abdominal	0.0060%	0.0000%	0.0124%	0.0096%
354.3	Lesion of radial nerve	0.0083%	0.0048%	0.0062%	0.0000%
242.10	Toxic uninodular goiter without mention of thyrotoxic crisi	0.0055%	0.0000%	0.0062%	0.0223%
285.9	Anemia, unspecified	0.0055%	0.0242%	0.0000%	0.0159%
164.2	Malignant neoplasm of anterior mediastinum	0.0051%	0.0242%	0.0083%	0.0064%
191.9	Malignant neoplasm of brain, unspecified	0.0069%	0.0048%	0.0021%	0.0159%
141.3	Malignant neoplasm of ventral surface of tongue	0.0060%	0.0097%	0.0145%	0.0000%
882.1	Open wound of hand except finger(s) alone, complicated	0.0069%	0.0145%	0.0062%	0.0000%

ICD-9-CM	ICD-9-CM	PureO	ContiB	NewB	PastB
945.33	Burn of ankle, full-thickness skin loss (third degree NOS)	0.0079%	0.0048%	0.0021%	0.0064%
952.08	C5-C7 level with central cord syndrome	0.0042%	0.0145%	0.0165%	0.0032%
944.38	Burn of multiple sites of wrist(s) and hand(s), full-thicknes	0.0079%	0.0048%	0.0062%	0.0000%
928.11	Crushing injury of knee	0.0065%	0.0000%	0.0145%	0.0000%
864.01	Hematoma and contusion to liver, without mention of open	0.0060%	0.0000%	0.0103%	0.0096%
806.25	T7-T12 level fracture with unspecified spinal cord injury, c	0.0032%	0.0048%	0.0248%	0.0032%
823.90	Fracture of unspecified part of tibia alone, open	0.0065%	0.0000%	0.0145%	0.0000%
811.01	Fracture in acromial process of scapula, closed	0.0051%	0.0000%	0.0124%	0.0127%
727.67	Rupture of achilles tendon	0.0055%	0.0048%	0.0083%	0.0127%
800.01	Fracture of vault of skull, closed without mention of intracr	0.0074%	0.0097%	0.0062%	0.0000%
693.0	Dermatitis due to drugs and medicines	0.0032%	0.0242%	0.0103%	0.0127%
647.81	Other specified infectious and parasitic diseases in the moth	0.0088%	0.0000%	0.0021%	0.0032%
718.45	Contracture of joint, pelvic region and thigh	0.0069%	0.0097%	0.0062%	0.0032%
675.24	Nonpurulent mastitis associated with childbirth, postpartum	0.0092%	0.0000%	0.0021%	0.0000%
726.5	Enthesopathy of hip region	0.0051%	0.0145%	0.0083%	0.0096%
719.65	Other symptoms referable to joint, pelvic region and thigh	0.0065%	0.0097%	0.0062%	0.0064%
614.0	Acute salpingitis and oophoritis	0.0055%	0.0000%	0.0124%	0.0096%
552.20	Ventral hernia, unspecified, with obstruction	0.0069%	0.0048%	0.0041%	0.0096%
569.85	Angiodysplasia of intestine with hemorrhage	0.0060%	0.0194%	0.0000%	0.0127%
530.4	Perforation of esophagus	0.0032%	0.0048%	0.0227%	0.0064%
543.9	Other and unspecified diseases of appendix	0.0083%	0.0048%	0.0021%	0.0032%
596.1	Intestinovesical fistula	0.0042%	0.0145%	0.0083%	0.0159%
564.89	Other functional disorders of intestine	0.0065%	0.0097%	0.0083%	0.0032%
428.1	Left heart failure	0.0037%	0.0097%	0.0083%	0.0223%
512.1	Iatrogenic pneumothorax	0.0065%	0.0000%	0.0124%	0.0032%
426.12	Mobitz (type II) atrioventricular block	0.0060%	0.0145%	0.0021%	0.0127%
229.8	Benign neoplasm of other specified sites	0.0079%	0.0048%	0.0021%	0.0064%
355.3	Lesion of lateral popliteal nerve	0.0060%	0.0048%	0.0103%	0.0064%
225.0	Benign neoplasm of brain	0.0060%	0.0194%	0.0062%	0.0032%
238.3	Neoplasm of uncertain behavior of breast	0.0079%	0.0097%	0.0041%	0.0000%
360.00	Purulent endophthalmitis unspecified	0.0060%	0.0097%	0.0103%	0.0032%
242.30	Toxic nodular goiter, unspecified, without mention of thyro	0.0074%	0.0000%	0.0000%	0.0159%
250.12	Diabetes with ketoacidosis, Type II [non-insulin dependent	0.0037%	0.0194%	0.0124%	0.0096%
280.0	Iron deficiency anemias, secondary to blood loss	0.0046%	0.0145%	0.0103%	0.0096%
183.2	Malignant neoplasm of fallopian tube	0.0005%	0.0291%	0.0289%	0.0000%
170.6	Malignant neoplasm of pelvic bones, sacrum and coccyx	0.0032%	0.0145%	0.0207%	0.0032%
078.11	Condyloma acuminatum viral warts	0.0074%	0.0097%	0.0000%	0.0096%
150.0	Malignant neoplasm of cervical esophagus	0.0014%	0.0145%	0.0269%	0.0064%
994.1	Effects of drowning and nonfatal submersion	0.0042%	0.0097%	0.0145%	0.0064%

ICD-9-CM	ICD-9-CM	PureO	ContiB	NewB	PastB
806.05	C5- C7 level fracture with unspecified spinal cord injury, c	0.0018%	0.0097%	0.0269%	0.0032%
812.44	Unspecified fracture of condyle(s) of humerus, closed	0.0069%	0.0000%	0.0021%	0.0127%
802.20	Fracture of mandible, closed, unspecified site	0.0088%	0.0048%	0.0000%	0.0000%
733.00	Osteoporosis, unspecified	0.0028%	0.0145%	0.0103%	0.0191%
747.10	Coarctation of aorta (preductal)(postductal)	0.0092%	0.0000%	0.0000%	0.0000%
733.15	Pathologic fracture of other specified part of femur	0.0042%	0.0097%	0.0145%	0.0064%
789.09	Abdominal pain, other specified site	0.0042%	0.0145%	0.0124%	0.0064%
666.24	Delayed and secondary postpartum hemorrhage, postpartun	0.0074%	0.0000%	0.0062%	0.0032%
685.0	Pilonidal cyst, with abscess	0.0083%	0.0000%	0.0021%	0.0032%
686.1	Pyogenic granuloma	0.0079%	0.0048%	0.0041%	0.0000%
718.85	Other joint derangement, not elsewhere classified, pelvic re	0.0083%	0.0048%	0.0000%	0.0032%
648.71	Bone and joint disorders of back,pelvis and lower limbs in	0.0083%	0.0000%	0.0021%	0.0032%
524.00	Unspecified anomaly of jaw size	0.0083%	0.0000%	0.0021%	0.0032%
598.1	Traumatic urethral stricture	0.0079%	0.0000%	0.0021%	0.0064%
599.6	Urinary obstruction, unspecified	0.0032%	0.0097%	0.0165%	0.0096%
595.82	Irradiation cystitis	0.0042%	0.0097%	0.0103%	0.0127%
553.00	Femoral hernia, unilateral or unspecified (not specified as r	0.0074%	0.0000%	0.0021%	0.0096%
633.9	Unspecified ectopic pregnancy	0.0088%	0.0000%	0.0021%	0.0000%
526.81	Exostosis of jaw	0.0051%	0.0242%	0.0021%	0.0096%
528.0	Stomatitis	0.0046%	0.0048%	0.0145%	0.0064%
614.5	Acute or unspecified pelvic peritonitis, female	0.0065%	0.0000%	0.0041%	0.0127%
617.5	Endometriosis of intestine	0.0055%	0.0048%	0.0103%	0.0064%
376.01	Orbital cellulitis	0.0046%	0.0048%	0.0083%	0.0159%
366.10	Senile cataract, unspecified	0.0074%	0.0000%	0.0021%	0.0096%
351.8	Other facial nerve disorders	0.0046%	0.0048%	0.0124%	0.0096%
213.8	Benign neoplasm of short bones of lower limb	0.0083%	0.0000%	0.0021%	0.0032%
361.01	Recent retinal detachment, partial, with single defect	0.0083%	0.0048%	0.0000%	0.0032%
216.5	Benign neoplasm of skin of trunk, except scrotum	0.0079%	0.0000%	0.0021%	0.0064%
011.95	Pulmonary tuberculosis, unspecified, tubercle bacilli not fo	0.0055%	0.0145%	0.0041%	0.0096%
202.83	Other lymphomas, intra-abdominal lymph nodes	0.0032%	0.0145%	0.0124%	0.0127%
196.2	Secondary and unspecified malignant neoplasm of intra-abd	0.0042%	0.0048%	0.0145%	0.0096%
197.4	Secondary malignant neoplasm of small intestine, including	0.0032%	0.0194%	0.0165%	0.0032%
947.3	Burn of gastrointestinal tract	0.0065%	0.0000%	0.0041%	0.0096%
996.01	Mechanical complication due to cardiac pacemaker (electro	0.0046%	0.0145%	0.0103%	0.0032%
969.4	Poisoning by benzodiazepine-based tranquilizers	0.0018%	0.0145%	0.0145%	0.0159%
944.20	Burn of unspecified site hand(s), blisters, epidermal loss (se	0.0074%	0.0000%	0.0021%	0.0064%
806.29	T7- T12 level fracture with other specified spinal cord inju	0.0018%	0.0145%	0.0207%	0.0064%
840.5	Sprains and strains of subscapularis (muscle)	0.0065%	0.0000%	0.0041%	0.0096%
808.49	Fracture of other specified part of pelvis, closed	0.0051%	0.0000%	0.0165%	0.0000%

ICD-9-CM	ICD-9-CM	PureO	ContiB	NewB	PastB
811.03	Fracture in glenoid cavity and neck of scapula, closed	0.0055%	0.0048%	0.0124%	0.0000%
729.4	Fasciitis, unspecified	0.0065%	0.0000%	0.0103%	0.0000%
787.91	Diarrhea	0.0028%	0.0097%	0.0145%	0.0127%
730.06	Acute osteomyelitis, lower leg	0.0051%	0.0000%	0.0103%	0.0096%
705.83	Hidradenitis	0.0079%	0.0000%	0.0021%	0.0032%
707.10	Ulcer of lower limb, unspecified	0.0051%	0.0048%	0.0083%	0.0096%
694.5	Pemphigoid	0.0060%	0.0097%	0.0062%	0.0032%
665.41	High vaginal laceration, delivered, with or without mentior	0.0088%	0.0000%	0.0000%	0.0000%
727.09	Other synovitis and tenosynovitis	0.0060%	0.0097%	0.0083%	0.0000%
654.41	Other abnormalities in shape or position of gravid uterus ar	0.0083%	0.0000%	0.0000%	0.0032%
715.32	Osteoarthrosis, localized, not specified whether primary or	0.0079%	0.0000%	0.0021%	0.0032%
680.5	Carbuncle and furuncle, buttock	0.0051%	0.0048%	0.0083%	0.0096%
562.00	Diverticulosis of small intestine (without mention of hemor	0.0051%	0.0048%	0.0062%	0.0127%
610.3	Fibrosclerosis of breast	0.0055%	0.0145%	0.0021%	0.0096%
524.2	Anomalies of dental arch relationship	0.0088%	0.0000%	0.0000%	0.0000%
573.9	Unspecified disorder of liver	0.0055%	0.0097%	0.0041%	0.0096%
530.7	Gastroesophageal laceration-hemorrhage syndrome	0.0042%	0.0194%	0.0083%	0.0064%
617.9	Endometriosis, site unspecified	0.0042%	0.0194%	0.0124%	0.0000%
519.1	Other diseases of trachea and bronchus, not elsewhere class	0.0060%	0.0048%	0.0083%	0.0032%
434.11	Cerebral embolism with cerebral infarction	0.0055%	0.0097%	0.0062%	0.0064%
447.1	Stricture of artery	0.0051%	0.0048%	0.0062%	0.0127%
521.0	Dental caries	0.0065%	0.0145%	0.0021%	0.0032%
471.9	Unspecified nasal polyp	0.0079%	0.0000%	0.0000%	0.0064%
336.0	Syringomyelia and syringobulbia	0.0060%	0.0000%	0.0083%	0.0064%
188.6	Malignant ureteric orifice	0.0032%	0.0048%	0.0124%	0.0159%
012.05	Tuberculous pleurisy , tubercle bacilli not found by bacteri	0.0065%	0.0048%	0.0062%	0.0032%
038.8	Other specified septicemias	0.0032%	0.0097%	0.0083%	0.0191%
015.06	Vertebral column tuberculosis, tubercle bacilli not found by	0.0028%	0.0048%	0.0207%	0.0064%
184.0	Malignant neoplasm of vagina	0.0037%	0.0145%	0.0165%	0.0000%
038.43	Septicemia due to Pseudomonas	0.0046%	0.0145%	0.0021%	0.0159%
155.2	Malignant neoplasm of liver, not specified as primary or se	0.0060%	0.0097%	0.0083%	0.0000%
890.1	Open wound of hip and thigh, complicated	0.0074%	0.0000%	0.0041%	0.0000%
935.2	Foreign body in stomach	0.0065%	0.0000%	0.0021%	0.0096%
958.3	Post-traumatic wound infection, not elsewhere classified	0.0046%	0.0145%	0.0083%	0.0032%
897.0	Traumatic amputation, unilateral , below knee, without mer	0.0037%	0.0000%	0.0207%	0.0000%
951.4	Injury to facial nerve	0.0055%	0.0048%	0.0062%	0.0064%
941.38	Burn of neck, full-thickness skin loss (third degree NOS)	0.0051%	0.0000%	0.0124%	0.0032%
880.03	Open wound of upper arm, without mention of complicatio	0.0069%	0.0000%	0.0041%	0.0032%
836.50	Dislocation of knee, unspecified, closed	0.0060%	0.0000%	0.0103%	0.0000%

ICD-9-CM	ICD-9-CM	PureO	ContiB	NewB	PastB
842.10	Sprains and strains of unspecified site, hand	0.0060%	0.0048%	0.0041%	0.0064%
853.02	Other and unspecified intracranial hemorrhage following in	0.0028%	0.0048%	0.0186%	0.0064%
747.0	Patent ductus arteriosus	0.0065%	0.0097%	0.0000%	0.0064%
788.20	Retention of urine, unspecified	0.0051%	0.0000%	0.0062%	0.0127%
728.79	Other fibromatoses	0.0069%	0.0000%	0.0041%	0.0032%
800.31	Fracture of vault of skull, closed with other and unspecified	0.0055%	0.0145%	0.0062%	0.0000%
801.25	Fracture of base of skull, closed with subarchnoid, subdura	0.0069%	0.0000%	0.0062%	0.0000%
753.4	Other specified anomalies of ureter	0.0069%	0.0000%	0.0041%	0.0032%
781.0	Abnormal involuntary movements	0.0042%	0.0145%	0.0103%	0.0032%
752.65	Hidden penis	0.0083%	0.0000%	0.0000%	0.0000%
658.01	Oligohydramnios, delivered, with or without mention of an	0.0079%	0.0000%	0.0021%	0.0000%
654.13	Tumors of body of uterus, antepartum condition or complic	0.0083%	0.0000%	0.0000%	0.0000%
682.8	Other cellulitis and abscess, other specified sites	0.0009%	0.0291%	0.0103%	0.0159%
719.46	Pain in joint, lower leg	0.0028%	0.0291%	0.0083%	0.0064%
707.13	Ulcer of ankle	0.0037%	0.0000%	0.0165%	0.0064%
642.61	Eclampsia, delivered, with or without mention of antepartu	0.0079%	0.0000%	0.0021%	0.0000%
642.91	Unspecified hypertension complicating pregnancy, childbir	0.0083%	0.0000%	0.0000%	0.0000%
719.54	Stiffness of joint, not elsewhere classified, hand	0.0060%	0.0048%	0.0083%	0.0000%
711.07	Pyogenic arthritis, ankle and foot	0.0055%	0.0048%	0.0103%	0.0000%
625.8	Other specified symptoms associated with femal genital org	0.0069%	0.0048%	0.0021%	0.0032%
427.5	Cardiac arrest	0.0051%	0.0048%	0.0041%	0.0127%
519.2	Mediastinitis	0.0060%	0.0000%	0.0083%	0.0032%
482.2	Pneumonia due to Hemophilus influenzae〔H. Influenzae〕	0.0028%	0.0194%	0.0021%	0.0223%
374.30	Ptosis of eyelid, unspecified	0.0079%	0.0000%	0.0021%	0.0000%
215.5	Benign neoplasm of connective and other soft tissue of abd	0.0060%	0.0048%	0.0062%	0.0032%
356.9	Hereditary and idiopathic peripheral neuropathy, unspecifie	0.0028%	0.0242%	0.0041%	0.0159%
343.1	Hemiplegic infantile cerebral palsy	0.0083%	0.0000%	0.0000%	0.0000%
210.5	Benign neoplasm of tonsil	0.0060%	0.0048%	0.0062%	0.0032%
164.9	Malignant neoplasm of mediastinum, part unspecified	0.0042%	0.0194%	0.0083%	0.0032%
202.18	Mycosis fungoides, lymph nodes of multiple sites	0.0042%	0.0194%	0.0062%	0.0064%
135	Sarcoidosis	0.0055%	0.0048%	0.0103%	0.0000%
145.5	Malignant neoplasm of palate, unspecified	0.0028%	0.0000%	0.0227%	0.0032%
904.41	Injury to popliteal artery	0.0042%	0.0097%	0.0124%	0.0000%
959.09	Injury of face and neck	0.0055%	0.0000%	0.0083%	0.0032%
996.52	Mechanical complication due to graft of other tissue, not el	0.0051%	0.0048%	0.0062%	0.0064%
959.1	Injury of trunk	0.0042%	0.0048%	0.0103%	0.0064%
943.20	Burn of unspecified site of upper limb, blisters, epidermal l	0.0060%	0.0048%	0.0041%	0.0032%
943.29	Burn of multiple sites of upper limb, except wrist and hand	0.0065%	0.0000%	0.0041%	0.0032%
989.3	Toxic effect of organophosphate and carbamate	0.0032%	0.0291%	0.0041%	0.0064%

ICD-9-CM	ICD-9-CM	PureO	ContiB	NewB	PastB
823.21	Fracture of shaft of fibula alone, closed	0.0037%	0.0048%	0.0145%	0.0032%
873.49	Open wound to other and multiple sites of face, without me	0.0065%	0.0000%	0.0041%	0.0032%
825.29	Fracture of other tarsal and metatarsal bones, closed	0.0051%	0.0048%	0.0083%	0.0032%
813.53	Fracture of distal end of ulna (alone), open	0.0060%	0.0000%	0.0062%	0.0032%
852.05	Subarachnoid hemorrhage following injury without mentio	0.0051%	0.0000%	0.0083%	0.0064%
873.20	Open wound to unspecified part of nose, without mention o	0.0055%	0.0048%	0.0083%	0.0000%
832.00	Unspecified closed dislocation of elbow	0.0055%	0.0000%	0.0062%	0.0064%
736.39	Other acquired deformities of hip	0.0060%	0.0097%	0.0021%	0.0032%
754.51	Talipes equinovarus	0.0074%	0.0000%	0.0021%	0.0000%
736.41	Genu valgum (acquired)	0.0065%	0.0097%	0.0021%	0.0000%
751.5	Other anomalies of intestine	0.0069%	0.0000%	0.0000%	0.0064%
727.81	Contracture of tendon (sheath)	0.0055%	0.0048%	0.0062%	0.0032%
717.43	Derangement of posterior horn of lateral meniscus	0.0046%	0.0048%	0.0083%	0.0064%
718.07	Articular cartilage disorder, ankle and foot	0.0060%	0.0048%	0.0021%	0.0064%
707.8	Chronic ulcer of other specified sites of skin	0.0065%	0.0000%	0.0041%	0.0032%
525.2	Atrophy of edentulous alveolar ridge	0.0046%	0.0097%	0.0041%	0.0096%
523.4	Chronic periodontitis	0.0042%	0.0097%	0.0021%	0.0159%
621.8	Other specified disorders of uterus, not elsewhere classified	0.0060%	0.0000%	0.0021%	0.0096%
534.40	Gastrojejunal ulcer, chronic or unspecified with hemorrhag	0.0051%	0.0000%	0.0103%	0.0032%
640.03	Threatened abortion, antepartum condition or complication	0.0065%	0.0000%	0.0041%	0.0032%
429.0	Myocarditis, unspecified	0.0051%	0.0048%	0.0083%	0.0032%
383.1	Chronic mastoiditis	0.0060%	0.0097%	0.0000%	0.0064%
444.81	Embolism and thrombosis of iliac artery	0.0051%	0.0242%	0.0000%	0.0032%
438.89	Other late effects of cerebrovascular disease	0.0018%	0.0194%	0.0103%	0.0127%
378.05	Alternating esotropia	0.0074%	0.0000%	0.0000%	0.0032%
385.33	Cholesteatoma of middle ear and mastoid	0.0046%	0.0097%	0.0062%	0.0064%
441.00	Dissection of aorta, unspecified site	0.0042%	0.0048%	0.0103%	0.0064%
366.9	Unspecified cataract	0.0065%	0.0048%	0.0000%	0.0064%
447.0	Arteriovenous fistula, acquired	0.0051%	0.0000%	0.0083%	0.0064%
208.00	Acute leukemia of unspecified cell type, without mention o	0.0032%	0.0194%	0.0062%	0.0096%
216.3	Benign neoplasm of skin of other and unspecified parts of f	0.0074%	0.0000%	0.0021%	0.0000%
250.62	Diabetes with neurological manifestations, Type II [non-ins	0.0028%	0.0194%	0.0062%	0.0127%
254.0	Persistent hyperplasia of thymus	0.0069%	0.0000%	0.0000%	0.0064%
274.82	Gouty tophi of other sites	0.0055%	0.0048%	0.0021%	0.0096%
141.1	Malignant neoplasm of dorsal surface of tongue	0.0042%	0.0048%	0.0083%	0.0096%
191.6	Malignant neoplasm of cerebellum	0.0037%	0.0097%	0.0083%	0.0096%
053.10	Herpes zoster with unspecified nervous system complicatio	0.0037%	0.0097%	0.0041%	0.0159%
202.08	Nodular lymphoma, lymph nodes of multiple sites	0.0028%	0.0194%	0.0062%	0.0127%
078.10	Viral warts, unspecified	0.0065%	0.0000%	0.0041%	0.0032%

ICD-9-CM	ICD-9-CM	PureO	ContiB	NewB	PastB
160.9	Malignant neoplasm of accessory sinus, unspecified	0.0042%	0.0048%	0.0083%	0.0096%
996.2	Mechanical complication of nervous system device, implan	0.0060%	0.0048%	0.0021%	0.0032%
922.31	Contusion of back	0.0046%	0.0048%	0.0021%	0.0127%
922.4	Contusion of genital organs	0.0069%	0.0000%	0.0021%	0.0000%
924.11	Contusion of knee	0.0055%	0.0000%	0.0062%	0.0032%
942.29	Burn of other and multiple sites of trunk, blisters, epiderma	0.0069%	0.0000%	0.0021%	0.0000%
903.2	Injury to radial blood vessels	0.0046%	0.0048%	0.0062%	0.0064%
878.2	Open wound of scrotum and testes, without mention of cor	0.0069%	0.0000%	0.0000%	0.0032%
802.24	Fracture of mandible, closed, ramus, unspecified	0.0055%	0.0000%	0.0083%	0.0000%
843.9	Sprains and strains of unspecified site of hip and thigh	0.0074%	0.0000%	0.0000%	0.0000%
861.01	Contusion to heart,without mention of open wound into tho	0.0051%	0.0000%	0.0103%	0.0000%
813.06	Neck of radius fractures, closed	0.0069%	0.0048%	0.0000%	0.0000%
823.01	Fracture of upper end of fibula alone, closed	0.0060%	0.0000%	0.0041%	0.0032%
841.8	Sprains and strains of other specified sites of elbow and for	0.0060%	0.0000%	0.0021%	0.0064%
872.01	Open wound to auricle of ear, without mention of complica	0.0060%	0.0000%	0.0062%	0.0000%
802.35	Fracture of mandible, open, angle of jaw	0.0069%	0.0000%	0.0021%	0.0000%
736.20	Unspecified acquired deformity of finger	0.0042%	0.0048%	0.0124%	0.0000%
756.19	Other anomalies of spine	0.0055%	0.0048%	0.0041%	0.0032%
800.24	Fracture of vault of skull, closed with subarachnoid, subdur	0.0046%	0.0097%	0.0083%	0.0000%
752.49	Other anomalies of cervix, vagina, and external female gen	0.0065%	0.0000%	0.0041%	0.0000%
736.02	Cubitus varus (acquired)	0.0074%	0.0000%	0.0000%	0.0000%
752.69	Other penile anomalies	0.0060%	0.0048%	0.0021%	0.0032%
749.03	Complete cleft palate, bilateral	0.0069%	0.0000%	0.0021%	0.0000%
801.20	Fracture of base of skull, closed with subarachnoid, subdur	0.0051%	0.0000%	0.0083%	0.0032%
748.4	Congenital cystic lung	0.0069%	0.0000%	0.0021%	0.0000%
749.20	Cleft palate with cleft lip, unspecified	0.0074%	0.0000%	0.0000%	0.0000%
726.60	Enthesopathy of knee, unspecified	0.0051%	0.0048%	0.0021%	0.0096%
671.81	Other venous complications in pregnancy and the puerperiu	0.0074%	0.0000%	0.0000%	0.0000%
718.82	Other joint derangement, not elsewhere classified, upper ar	0.0051%	0.0048%	0.0021%	0.0096%
727.40	Synovial cyst, unspecified	0.0046%	0.0000%	0.0083%	0.0064%
718.01	Articular cartilage disorder, shoulder region	0.0051%	0.0048%	0.0041%	0.0064%
655.01	Central nervous system malformation in fetus, affecting ma	0.0074%	0.0000%	0.0000%	0.0000%
663.01	Prolapse of cord, delivered, with or without mention of ant	0.0074%	0.0000%	0.0000%	0.0000%
646.63	Infections of genitourinary tract in pregnancy, antepartum c	0.0065%	0.0000%	0.0000%	0.0064%
722.72	Thoracic intervertebral disc disorder with myelopathy	0.0018%	0.0000%	0.0227%	0.0032%
716.15	Traumatic arthropathy, pelvic region and thigh	0.0051%	0.0000%	0.0062%	0.0064%
571.6	Biliary cirrhosis	0.0028%	0.0097%	0.0103%	0.0096%
634.91	Abortion, without mention of complication, incomplete	0.0069%	0.0000%	0.0000%	0.0032%
555.0	Regional enteritis, small intestine	0.0046%	0.0048%	0.0041%	0.0096%

ICD-9-CM	ICD-9-CM	PureO	ContiB	NewB	PastB
611.9	Unspecified breast disorder	0.0051%	0.0048%	0.0000%	0.0127%
635.91	Legally induced abortion, without mention of complication	0.0074%	0.0000%	0.0000%	0.0000%
535.51	Unspecified gastritis and gastroduodenitis, with hemorrhag	0.0051%	0.0048%	0.0041%	0.0064%
526.1	Fissural cysts of jaw	0.0065%	0.0000%	0.0041%	0.0000%
500	Coal workers' pneumoconiosis	0.0037%	0.0145%	0.0062%	0.0064%
482.82	Pneumonia due to Escherichia coli 〔E. coli〕	0.0032%	0.0097%	0.0041%	0.0159%
459.0	Hemorrhage, unspecified	0.0042%	0.0145%	0.0062%	0.0032%
519.02	Mechanical complication of tracheostomy	0.0018%	0.0242%	0.0021%	0.0191%
348.0	Cerebral cysts	0.0046%	0.0145%	0.0041%	0.0032%
237.5	Neoplasm of uncertain behavior of brain and spinal cord	0.0037%	0.0145%	0.0062%	0.0064%
348.5	Cerebral edema	0.0037%	0.0097%	0.0062%	0.0096%
283.0	Autoimmune hemolytic anemias	0.0042%	0.0194%	0.0021%	0.0064%
031.0	Pulmonary diseases due to other mycobacteria	0.0023%	0.0291%	0.0083%	0.0032%
017.25	Tuberculosis of peripheral lymph nodes, tubercle bacilli no	0.0051%	0.0048%	0.0062%	0.0032%
202.00	Nodular lymphoma, unspecified site, extranodal solid orga	0.0046%	0.0000%	0.0103%	0.0032%
927.03	Crushing injury of upper arm	0.0037%	0.0048%	0.0103%	0.0032%
996.02	Mechanical complication due to heart valve prosthesis	0.0028%	0.0145%	0.0103%	0.0032%
956.3	Injury to peroneal nerve	0.0060%	0.0000%	0.0021%	0.0032%
941.28	Burn of neck, blisters, epidermal loss (second degree)	0.0046%	0.0048%	0.0062%	0.0032%
887.2	Traumatic amputation, unilateral, at or above elbow, witho	0.0042%	0.0000%	0.0124%	0.0000%
876.0	Open wound of back, without mention of complication	0.0051%	0.0000%	0.0083%	0.0000%
941.32	Burn of eye (with other parts of face, head and neck), full-t	0.0055%	0.0048%	0.0041%	0.0000%
884.0	Multiple and unspecified open wound of upper limb, witho	0.0055%	0.0048%	0.0041%	0.0000%
820.00	Unspecified fracture of intracapsular section of femur, clos	0.0032%	0.0097%	0.0062%	0.0096%
862.0	Injury to diaphragm,without mention of open wound into c	0.0042%	0.0000%	0.0124%	0.0000%
873.52	Open wound of forehead, complicated	0.0065%	0.0000%	0.0021%	0.0000%
866.01	Hematoma without rupture of capsule in kidney, without m	0.0046%	0.0000%	0.0103%	0.0000%
733.11	Pathologic fracture of humerus	0.0060%	0.0000%	0.0041%	0.0000%
801.16	Fracture of base of skull, closed with cerebral laceration an	0.0046%	0.0048%	0.0062%	0.0032%
748.5	Agenesis, hypoplasia and dysplasia of lung	0.0060%	0.0000%	0.0021%	0.0032%
752.52	Retractile testis	0.0069%	0.0000%	0.0000%	0.0000%
755.13	Syndactyly of toes without fusion of bone	0.0065%	0.0000%	0.0021%	0.0000%
746.89	Other specified anomalies of heart	0.0046%	0.0048%	0.0041%	0.0064%
787.01	Nausea with vomiting	0.0018%	0.0194%	0.0103%	0.0064%
754.70	Talipes, unspecified	0.0069%	0.0000%	0.0000%	0.0000%
755.11	Syndactyly of fingers without fusion of bone	0.0069%	0.0000%	0.0000%	0.0000%
674.34	Other complications of osatetrical surgical wounds, postpa	0.0060%	0.0000%	0.0000%	0.0064%
717.2	Derangement of posterior horn of medial meniscus	0.0037%	0.0000%	0.0083%	0.0096%
715.33	Osteoarthrosis, localized, not specified whether primary or	0.0055%	0.0000%	0.0021%	0.0064%

ICD-9-CM	ICD-9-CM	PureO	ContiB	NewB	PastB
710.3	Dermatomyositis	0.0023%	0.0145%	0.0103%	0.0064%
635.72	Legally induced abortion, with other specified complicatior	0.0055%	0.0048%	0.0041%	0.0000%
550.12	Inguinal hernia, with obstruction, without mention of gangi	0.0046%	0.0000%	0.0041%	0.0096%
553.8	Hernia of other specified sites	0.0046%	0.0048%	0.0021%	0.0096%
634.92	Abortion, without mention of complication, complete	0.0069%	0.0000%	0.0000%	0.0000%
635.12	Legally induced abortion, complicated by delayed or exces	0.0060%	0.0048%	0.0021%	0.0000%
593.71	Vesicoureteral reflux , with reflux nephropathy, unilateral	0.0069%	0.0000%	0.0000%	0.0000%
596.3	Diverticulum of bladder	0.0055%	0.0048%	0.0041%	0.0000%
447.6	Arteritis, unspecified	0.0051%	0.0048%	0.0041%	0.0032%
458.0	Orthostatic hypotension	0.0032%	0.0097%	0.0041%	0.0127%
456.0	Esophageal varices with bleeding	0.0046%	0.0145%	0.0021%	0.0032%
443.9	Peripheral vascular disease, unspecified	0.0014%	0.0145%	0.0124%	0.0096%
513.1	Abscess of mediastinum	0.0055%	0.0048%	0.0041%	0.0000%
378.20	Intermittent heterotropia, unspecified	0.0069%	0.0000%	0.0000%	0.0000%
378.00	Esotropia, unspecified	0.0065%	0.0000%	0.0000%	0.0032%
379.31	Aphakia	0.0065%	0.0048%	0.0000%	0.0000%
474.2	Adenoid vegetations	0.0069%	0.0000%	0.0000%	0.0000%
478.6	Edema of larynx	0.0051%	0.0194%	0.0000%	0.0000%
237.0	Neoplasm of uncertain behavior of pituitary gland and cran	0.0037%	0.0000%	0.0124%	0.0032%
255.1	Hyperaldosteronism	0.0051%	0.0000%	0.0041%	0.0064%
212.1	Benign neoplasm of larynx	0.0069%	0.0000%	0.0000%	0.0000%
276.7	Hyperpotassemia	0.0028%	0.0048%	0.0083%	0.0127%
255.4	Corticoadrenal insufficiency	0.0028%	0.0097%	0.0062%	0.0127%
215.6	Benign neoplasm of connective and other soft tissue of pel	0.0042%	0.0048%	0.0041%	0.0096%
210.1	Benign neoplasm of tongue	0.0055%	0.0097%	0.0000%	0.0032%
336.1	Vascular myelopathies	0.0023%	0.0145%	0.0124%	0.0032%
151.3	Malignant neoplasm of fundus of stomach	0.0055%	0.0000%	0.0062%	0.0000%
008.45	Intestinal infections due to Clostridium difficile	0.0014%	0.0145%	0.0041%	0.0223%
158.8	Malignant neoplasm of specified parts of peritoneum	0.0032%	0.0194%	0.0062%	0.0032%
175.9	Malignant neoplasm of other and unspecified sites of male	0.0042%	0.0097%	0.0041%	0.0064%
964.2	Poisoning by anticoagulants	0.0028%	0.0048%	0.0041%	0.0159%
997.5	Urinary complications	0.0042%	0.0048%	0.0021%	0.0096%
927.21	Crushing injury of wrist	0.0055%	0.0000%	0.0041%	0.0000%
821.00	Fracture of unspecified part of femur, closed	0.0018%	0.0097%	0.0083%	0.0127%
864.03	Moderate laceration to liver, without mention of open wou	0.0051%	0.0000%	0.0041%	0.0032%
805.04	Fracture of fourth cervical vertebra, closed	0.0032%	0.0048%	0.0103%	0.0032%
802.29	Fracture of mandible, closed, mutiple sites	0.0051%	0.0000%	0.0062%	0.0000%
825.22	Fracture of navicular〔scaphoid〕of foot, closed	0.0051%	0.0000%	0.0041%	0.0032%
853.00	Other and unspecified intracranial hemorrhage following ir	0.0023%	0.0097%	0.0103%	0.0064%

ICD-9-CM	ICD-9-CM	PureO	ContiB	NewB	PastB
862.29	Injury to other specified intrathoracic organs,without menti	0.0046%	0.0048%	0.0021%	0.0064%
844.1	Sprains and strains of medial collateral ligament of knee	0.0037%	0.0097%	0.0041%	0.0064%
852.23	Subdural hemorrhage following injury without mention of	0.0042%	0.0048%	0.0062%	0.0032%
873.59	Open wound to other and multiple sites of face, complicate	0.0046%	0.0048%	0.0062%	0.0000%
808.9	Unspecified fracture of pelvis, open	0.0028%	0.0000%	0.0145%	0.0032%
736.09	Other acquired deformities of forearm, excluding fingers	0.0055%	0.0048%	0.0021%	0.0000%
800.16	Fracture of vault of skull, closed with cerebral laceration a	0.0055%	0.0048%	0.0000%	0.0032%
730.18	Chronic osteomyelitis, other specified sites	0.0023%	0.0242%	0.0041%	0.0064%
748.1	Other anomalies of nose	0.0055%	0.0000%	0.0021%	0.0032%
735.8	Other acquired deformities of toe	0.0065%	0.0000%	0.0000%	0.0000%
732.1	Juvenile osteochondrosis of hip and pelvis	0.0055%	0.0000%	0.0041%	0.0000%
736.1	Mallet finger	0.0055%	0.0000%	0.0000%	0.0064%
801.23	Fracture of base of skull, closed with subarachnoid, subdur	0.0046%	0.0000%	0.0062%	0.0032%
789.03	Abdominal pain, right lower quadrant	0.0037%	0.0000%	0.0103%	0.0032%
800.22	Fracture of vault of skull, closed with subarachnoid, subdu	0.0046%	0.0048%	0.0021%	0.0064%
735.5	Claw toe (acquired)	0.0046%	0.0097%	0.0021%	0.0032%
800.36	Fracture of vault of skull, closed with other and unspecifie	0.0055%	0.0048%	0.0021%	0.0000%
701.3	Striae atrophicae	0.0051%	0.0048%	0.0000%	0.0064%
718.26	Pathological dislocation, lower leg	0.0042%	0.0048%	0.0041%	0.0064%
680.6	Carbuncle and furuncle, leg, except foot	0.0042%	0.0097%	0.0041%	0.0032%
722.11	Displacement of thoracic intervertebral disc without myelo	0.0018%	0.0291%	0.0062%	0.0032%
666.04	Third-stage hemorrhage, postpartum condition or complica	0.0055%	0.0000%	0.0041%	0.0000%
530.89	Other specified disorders of esophagus	0.0051%	0.0000%	0.0041%	0.0032%
620.9	Unspecified noninflammatory disorder of ovary, fallopian t	0.0051%	0.0000%	0.0021%	0.0064%
595.2	Other chronic cystitis	0.0028%	0.0097%	0.0062%	0.0096%
616.10	Vaginitis and vulvovaginitis, unspecified	0.0051%	0.0048%	0.0021%	0.0032%
626.2	Excessive or frequent menstruation	0.0046%	0.0000%	0.0000%	0.0127%
573.3	Hepatitis, unspecified	0.0042%	0.0097%	0.0021%	0.0064%
524.61	Adhesions and ankylosis (bony or fibrous)	0.0055%	0.0000%	0.0041%	0.0000%
423.8	Other specified diseases of pericardium	0.0032%	0.0194%	0.0041%	0.0032%
441.7	Thoracoabdominal aneurysm, without mention of rupture	0.0018%	0.0048%	0.0145%	0.0064%
492.8	Other emphysema	0.0046%	0.0145%	0.0021%	0.0000%
461.9	Acute sinusitis, unspecified	0.0042%	0.0000%	0.0021%	0.0127%
423.2	Constrictive pericarditis	0.0042%	0.0000%	0.0083%	0.0032%
385.82	Cholesterin granuloma	0.0060%	0.0000%	0.0021%	0.0000%
482.9	Bacterial pneumonia, unspecified	0.0023%	0.0048%	0.0103%	0.0096%
466.0	Acute bronchitis	0.0032%	0.0097%	0.0021%	0.0127%
493.90	Asthma, unspecified, without mention of status asthmaticus	0.0023%	0.0291%	0.0021%	0.0064%
383.32	Recurrent cholesteatoma of postmastoidectomy cavity	0.0046%	0.0048%	0.0021%	0.0064%

ICD-9-CM	ICD-9-CM	PureO	ContiB	NewB	PastB
370.06	Perforated corneal ulcer	0.0032%	0.0048%	0.0062%	0.0096%
478.74	Stenosis of larynx	0.0051%	0.0000%	0.0000%	0.0096%
360.01	Acute endophthalmitis	0.0046%	0.0000%	0.0021%	0.0096%
345.40	Partial epilepsy, with impairment of consciousness without	0.0018%	0.0145%	0.0021%	0.0191%
277.8	Other specified disorders of metabolism	0.0037%	0.0048%	0.0083%	0.0032%
239.6	Neoplasm of unspecified nature of brain	0.0028%	0.0097%	0.0041%	0.0127%
196.1	Secondary and unspecified malignant neoplasm of intrathor	0.0023%	0.0145%	0.0103%	0.0032%
202.01	Nodular lymphoma, lymph nodes of head, face and neck	0.0037%	0.0048%	0.0083%	0.0032%
143.9	Malignant neoplasm of gum, unspecified	0.0018%	0.0097%	0.0145%	0.0032%
141.4	Malignant neoplasm of anterior two-thirds of tongue, part u	0.0023%	0.0194%	0.0083%	0.0032%
053.11	Geniculate herpes zoster	0.0023%	0.0048%	0.0103%	0.0096%
188.1	Malignant neoplasm of dome of urinary bladder	0.0032%	0.0097%	0.0041%	0.0096%
924.5	Contusion of unspecified part of lower limb	0.0051%	0.0000%	0.0021%	0.0032%
922.2	Contusion of abdominal wall	0.0037%	0.0048%	0.0041%	0.0064%
945.20	Burn of unspecified site of lower limb (leg), blisters, epider	0.0037%	0.0048%	0.0062%	0.0032%
893.1	Open wound of toe(s), complicated	0.0037%	0.0097%	0.0041%	0.0032%
996.74	Other complications due to vascular device, implant, and gr	0.0037%	0.0000%	0.0041%	0.0096%
815.10	Fracture in unspecified site of metacarpal bone(s), open	0.0046%	0.0000%	0.0062%	0.0000%
865.02	Capsular tears to spleen, without major disruption of paren	0.0037%	0.0000%	0.0083%	0.0032%
812.50	Fracture in unspecified part of lower end of humerus, open	0.0046%	0.0000%	0.0041%	0.0032%
833.01	Closed dislocation of radioulnar (joint) of distal	0.0042%	0.0048%	0.0021%	0.0064%
863.21	Injury to duodenum,without mention of open wound into ca	0.0037%	0.0000%	0.0103%	0.0000%
844.9	Sprains and strains of unspecified site of knee and leg	0.0028%	0.0097%	0.0083%	0.0032%
813.31	Fracture of shaft of radius(alone), open	0.0042%	0.0000%	0.0041%	0.0064%
837.0	Dislocation of ankle, closed	0.0046%	0.0000%	0.0062%	0.0000%
868.04	Injury to retroperitoneum without mention of open wound i	0.0046%	0.0000%	0.0062%	0.0000%
835.00	Unspecified closed dislocation of hip	0.0046%	0.0000%	0.0062%	0.0000%
864.00	Unspecified injury to liver, without mention of open wound	0.0037%	0.0000%	0.0103%	0.0000%
747.69	Anomalies of other specified sites of peripheral vascular sy	0.0055%	0.0048%	0.0000%	0.0000%
787.2	Dysphagia	0.0014%	0.0145%	0.0083%	0.0096%
801.36	Fracture of base of skull, closed with other and unspecified	0.0037%	0.0097%	0.0041%	0.0032%
801.11	Fracture of base of skull, closed with cerebral laceration an	0.0037%	0.0000%	0.0103%	0.0000%
730.24	Unspecified osteomyelitis, hand	0.0042%	0.0000%	0.0062%	0.0032%
738.8	Acquired deformity of other specified site	0.0042%	0.0000%	0.0041%	0.0064%
736.00	Unspecified deformity of forearm	0.0037%	0.0000%	0.0083%	0.0032%
721.7	Traumatic spondylopathy	0.0028%	0.0048%	0.0103%	0.0032%
718.87	Other joint derangement, not elsewhere classified, ankle an	0.0046%	0.0048%	0.0021%	0.0032%
718.47	Contracture of joint, ankle and foot	0.0037%	0.0048%	0.0062%	0.0032%
715.24	Osteoarthrosis, localized, secondary, hand	0.0028%	0.0145%	0.0062%	0.0032%

ICD-9-CM	ICD-9-CM	PureO	ContiB	NewB	PastB
717.41	Bucket handle tear of lateral meniscus	0.0055%	0.0000%	0.0000%	0.0032%
715.94	Osteoarthrosis, unspecified whether generalized or localize	0.0028%	0.0097%	0.0041%	0.0096%
716.96	Arthropathy, unspecified, lower leg	0.0037%	0.0097%	0.0041%	0.0032%
666.12	Other immediate postpartum hemorrhage,delivered, with m	0.0055%	0.0000%	0.0000%	0.0032%
717.89	Other internal derangement of knee	0.0055%	0.0000%	0.0000%	0.0032%
523.8	Other specified periodontal diseases	0.0037%	0.0097%	0.0041%	0.0032%
534.50	Gastrojejunal ulcer, chronic or unspecified with perforation	0.0046%	0.0000%	0.0062%	0.0000%
602.1	Congestion or hemorrhage of prostate	0.0023%	0.0194%	0.0000%	0.0127%
616.4	Other abscess of vulva	0.0046%	0.0000%	0.0041%	0.0032%
533.10	Peptic ulcer, site unspecified, acute with perforation, witho	0.0046%	0.0000%	0.0041%	0.0032%
550.00	Inguinal hernia, with gangrene, unilateral or unspecified (n	0.0055%	0.0000%	0.0000%	0.0032%
583.9	Nephritis and nephropathy, not specified as acute or chroni	0.0042%	0.0000%	0.0041%	0.0064%
379.32	Subluxation of lens	0.0051%	0.0000%	0.0021%	0.0032%
493.22	Chronic obstructive asthma (with obstructive pulmonary di	0.0023%	0.0048%	0.0041%	0.0159%
514	Pulmonary congestion and hypostasis	0.0018%	0.0048%	0.0062%	0.0159%
413.1	Prinzmetal angina	0.0032%	0.0048%	0.0041%	0.0096%
388.2	Sudden hearing loss, unspecified	0.0028%	0.0145%	0.0021%	0.0096%
519.09	Other tracheostomy complications	0.0023%	0.0048%	0.0000%	0.0223%
512.0	Spontaneous tension pneumothorax	0.0051%	0.0048%	0.0000%	0.0032%
522.5	Periapical abscess without sinus	0.0037%	0.0048%	0.0062%	0.0032%
518.1	Interstitial emphysema	0.0046%	0.0000%	0.0041%	0.0032%
410.01	Acute myocardial infarction of anterolateral wall, initial ep	0.0046%	0.0048%	0.0041%	0.0000%
473.2	Chronic sinusitis of ethmoidal	0.0051%	0.0000%	0.0041%	0.0000%
348.3	Encephalopathy, unspecified	0.0023%	0.0194%	0.0021%	0.0096%
228.02	Hemangioma of intracranial structures	0.0028%	0.0194%	0.0041%	0.0032%
349.2	Disorders of meninges, not elsewhere classified	0.0046%	0.0048%	0.0021%	0.0032%
250.42	Diabetes with renal manifestations, Type II [non-insulin de	0.0014%	0.0242%	0.0041%	0.0096%
355.8	Unspecified mononeuritis of lower limb	0.0032%	0.0097%	0.0062%	0.0032%
293.0	Acute delirium	0.0009%	0.0194%	0.0062%	0.0127%
294.8	Other specified organic brain syndromes (chronic)	0.0023%	0.0048%	0.0041%	0.0159%
187.7	Malignant neoplasm of scrotum	0.0037%	0.0145%	0.0041%	0.0000%
188.5	Malignant neoplasm of bladder neck	0.0037%	0.0000%	0.0062%	0.0064%
012.06	Tuberculous pleurisy , tubercle bacilli not found by bacteri	0.0014%	0.0242%	0.0062%	0.0064%
038.3	Septicemia due to anaerobes	0.0032%	0.0145%	0.0000%	0.0096%
136.3	Pneumocystosis	0.0032%	0.0097%	0.0000%	0.0127%
161.2	Malignant neoplasm of subglottis	0.0023%	0.0097%	0.0062%	0.0096%
015.05	Vertebral column tuberculosis, tubercle bacilli not found by	0.0042%	0.0097%	0.0041%	0.0000%
V08	Asymptomatic human immunodeficiency virus 〔HIV〕in	0.0023%	0.0048%	0.0103%	0.0032%
893.2	Open wound of toe(s), with tendon involvement	0.0051%	0.0000%	0.0000%	0.0032%

ICD-9-CM	ICD-9-CM	PureO	ContiB	NewB	PastB
997.2	Peripheral vascular complications	0.0037%	0.0048%	0.0041%	0.0032%
996.32	Mechanical complication due to intrauterine contraceptive	0.0032%	0.0000%	0.0041%	0.0096%
863.84	Injury to multiple and unspecified site of pancreas, without	0.0037%	0.0000%	0.0062%	0.0032%
816.00	Unspecified fracture of phalanx or phalanges, closed	0.0037%	0.0000%	0.0083%	0.0000%
853.05	Other and unspecified intracranial hemorrhage following in	0.0032%	0.0000%	0.0062%	0.0064%
825.31	Fracture of astragalus, open	0.0042%	0.0048%	0.0021%	0.0032%
801.22	Fracture of base of skull, closed with subarachnoid, subdur	0.0032%	0.0000%	0.0103%	0.0000%
789.30	Abdominal or pelvic swelling, mass, or lump, unspecified s	0.0042%	0.0048%	0.0021%	0.0032%
801.31	Fracture of base of skull, closed with other and unspecified	0.0037%	0.0048%	0.0041%	0.0032%
751.2	Atresia and stenosis of large intestine, rectum and anal can	0.0055%	0.0000%	0.0000%	0.0000%
800.06	Fracture of vault of skull, closed without mention of intracr	0.0032%	0.0048%	0.0083%	0.0000%
782.3	Edema	0.0018%	0.0097%	0.0103%	0.0032%
733.22	Aneurysmal bone cyst	0.0051%	0.0000%	0.0021%	0.0000%
754.31	Congenital dislocation of hip, bilateral	0.0055%	0.0000%	0.0000%	0.0000%
733.41	Aseptic necrosis of head of humerus	0.0018%	0.0000%	0.0062%	0.0159%
710.1	Systemic sclerosis	0.0018%	0.0145%	0.0083%	0.0032%
719.66	Other symptoms referable to joint, lower leg	0.0037%	0.0000%	0.0041%	0.0064%
727.03	Trigger finger (acquired)	0.0028%	0.0000%	0.0041%	0.0127%
718.03	Articular cartilage disorder, forearm	0.0046%	0.0048%	0.0000%	0.0032%
721.8	Other allied disorders of spine	0.0037%	0.0048%	0.0062%	0.0000%
718.35	Recurrent dislocation of joint, pelvic region and thigh	0.0037%	0.0048%	0.0062%	0.0000%
648.01	Diabetes mellitus conditions in the mother classifiable else	0.0051%	0.0000%	0.0021%	0.0000%
710.2	Sicca syndrome	0.0018%	0.0194%	0.0041%	0.0064%
648.51	Congenital cardiovascular disorders in the mother classifial	0.0051%	0.0000%	0.0000%	0.0032%
719.51	Stiffness of joint, not elsewhere classified, shoulder region	0.0032%	0.0145%	0.0021%	0.0032%
581.1	Nephrotic syndrome, with lesion of membranous glomerulo	0.0028%	0.0000%	0.0083%	0.0064%
608.1	Spermatocele	0.0046%	0.0000%	0.0041%	0.0000%
596.54	Neurogenic bladder NOS	0.0023%	0.0145%	0.0041%	0.0064%
592.9	Urinary calculus, unspecified	0.0037%	0.0000%	0.0062%	0.0032%
611.4	Atrophy of breast	0.0042%	0.0000%	0.0021%	0.0064%
371.20	Corneal edema, unspecified	0.0046%	0.0000%	0.0000%	0.0064%
473.1	Chronic sinusitis of frontal	0.0037%	0.0000%	0.0000%	0.0127%
444.21	Arterial embolism and thrombosis of upper extremity	0.0028%	0.0000%	0.0062%	0.0096%
383.9	Unspecified mastoiditis	0.0018%	0.0097%	0.0083%	0.0064%
378.51	Third or oculomotor nerve palsy, partial	0.0032%	0.0097%	0.0062%	0.0000%
442.3	Aneurysm of artery of lower extremity	0.0037%	0.0048%	0.0041%	0.0032%
378.55	External ophthalmoplegia	0.0028%	0.0097%	0.0083%	0.0000%
480.9	Viral pneumonia, unspecified	0.0037%	0.0097%	0.0021%	0.0032%
250.52	Diabetes with ophthalmic manifestations, Type II [non-insu	0.0023%	0.0194%	0.0000%	0.0096%

ICD-9-CM	ICD-9-CM	PureO	ContiB	NewB	PastB
238.8	Neoplasm of uncertain behavior of other specified sites	0.0051%	0.0000%	0.0021%	0.0000%
216.7	Benign neoplasm of skin of lower limb, including hip	0.0055%	0.0000%	0.0000%	0.0000%
216.2	Benign neoplasm of skin of ear and external auditory canal	0.0037%	0.0048%	0.0000%	0.0096%
263.9	Unspecified protein-calorie malnutrition	0.0023%	0.0048%	0.0062%	0.0096%
213.2	Benign neoplasm of vertebral column, excluding sacrum ar	0.0042%	0.0000%	0.0021%	0.0064%
296.33	Major depressive disorder, recurrent episode, severe withou	0.0000%	0.0097%	0.0000%	0.0318%
282.0	Hereditary spherocytosis	0.0055%	0.0000%	0.0000%	0.0000%
348.8	Other conditions of brain	0.0009%	0.0048%	0.0083%	0.0159%
236.7	Neoplasm of uncertain behavior of bladder	0.0037%	0.0000%	0.0041%	0.0064%
230.2	Carcinoma in situ of stomach	0.0032%	0.0048%	0.0021%	0.0096%
156.9	Malignant neoplasm of biliary tract, part unspecified	0.0037%	0.0000%	0.0062%	0.0032%
038.19	Other staphylococcal septicemia	0.0023%	0.0097%	0.0041%	0.0096%
015.03	Vertebral column tuberculosis, tubercle bacilli found (in sp	0.0023%	0.0097%	0.0103%	0.0000%
136.9	Unspecified infectious and parasitic diseases	0.0028%	0.0048%	0.0062%	0.0064%
011.92	Pulmonary tuberculosis, unspecified, bacteriological or hist	0.0028%	0.0048%	0.0041%	0.0096%
170.1	Malignant neoplasm of mandible	0.0023%	0.0000%	0.0124%	0.0032%
204.10	Chronic lymphoid leukemia, without mention of remission	0.0028%	0.0048%	0.0083%	0.0032%
196.5	Secondary and unspecified malignant neoplasm of lymph n	0.0028%	0.0048%	0.0083%	0.0032%
996.64	Infection and inflammatory reaction due to indwelling urin	0.0018%	0.0097%	0.0041%	0.0096%
997.09	Other nervous system complications	0.0028%	0.0097%	0.0021%	0.0064%
881.02	Open wound of wrist, without mention of complication	0.0037%	0.0048%	0.0021%	0.0032%
881.01	Open wound of elbow, without mention of complication	0.0051%	0.0000%	0.0000%	0.0000%
886.1	Traumatic amputation of other fingers (complete) (partial),	0.0032%	0.0097%	0.0041%	0.0000%
878.0	Open wound of penis, without mention of complication, in	0.0037%	0.0048%	0.0041%	0.0000%
941.24	Burn of chin, blisters, epidermal loss (second degree)	0.0051%	0.0000%	0.0000%	0.0000%
V67.09	Following other surgery	0.0042%	0.0048%	0.0000%	0.0032%
860.3	Traumatic haemothorax with open wound into thorax	0.0032%	0.0000%	0.0083%	0.0000%
870.8	Other specified open wounds of ocular adnexa	0.0046%	0.0000%	0.0021%	0.0000%
860.1	Traumatic pneumothorax with open wound into thorax	0.0037%	0.0000%	0.0062%	0.0000%
873.44	Open wound of jaw, without mention of complication	0.0046%	0.0048%	0.0000%	0.0000%
852.03	Subarachnoid hemorrhage following injury without mentio	0.0046%	0.0000%	0.0021%	0.0000%
806.03	C1- C4 level fracture with central cord syndrome, closed	0.0009%	0.0000%	0.0186%	0.0000%
831.00	Unspecified closed dislocation of shoulder	0.0032%	0.0048%	0.0041%	0.0032%
806.20	T1-T6 level fracture with unspecified spinal cord injury, cl	0.0023%	0.0048%	0.0083%	0.0032%
815.13	Fracture of shaft of metacarpal bone(s), open	0.0046%	0.0000%	0.0021%	0.0000%
837.1	Dislocation of ankle, open	0.0028%	0.0000%	0.0083%	0.0032%
745.10	Complete transposition of great vessels	0.0046%	0.0000%	0.0021%	0.0000%
749.00	Cleft palate, unspecified	0.0046%	0.0000%	0.0021%	0.0000%
782.4	Jaundice, unspecified, not of newborn	0.0028%	0.0145%	0.0041%	0.0000%

ICD-9-CM	ICD-9-CM	PureO	ContiB	NewB	PastB
789.2	Splenomegaly	0.0023%	0.0048%	0.0041%	0.0096%
754.89	Other specified nonteratogenic anomalies	0.0051%	0.0000%	0.0000%	0.0000%
747.63	Upper limb vessel anomaly	0.0042%	0.0000%	0.0021%	0.0032%
729.2	Neuralgia, neuritis and radiculitis, unspecified	0.0000%	0.0145%	0.0124%	0.0064%
756.6	Anomalies of diaphragm	0.0046%	0.0048%	0.0000%	0.0000%
716.97	Arthropathy, unspecified, ankle and foot	0.0037%	0.0048%	0.0000%	0.0064%
654.51	Cervical incompetence, delivered, with or without mention	0.0051%	0.0000%	0.0000%	0.0000%
717.42	Derangement of anterior horn of lateral meniscus	0.0023%	0.0097%	0.0083%	0.0000%
716.17	Traumatic arthropathy, ankle and foot	0.0032%	0.0097%	0.0000%	0.0064%
659.51	Elderly primigravida, delivered, with or without mention o	0.0046%	0.0000%	0.0000%	0.0032%
659.61	Elderly multigravida, delivered, with or without mention of	0.0042%	0.0000%	0.0000%	0.0064%
623.8	Other specified noninflammatory disorders of vagina	0.0032%	0.0048%	0.0041%	0.0032%
614.8	Other specified inflammatory disease of female pelvic orga	0.0046%	0.0000%	0.0000%	0.0032%
596.6	Rupture of bladder, nontraumatic	0.0018%	0.0048%	0.0083%	0.0064%
574.41	Calculus of bile duct with other cholecystitis with obstructi	0.0028%	0.0097%	0.0000%	0.0096%
616.3	Abscess of bartholin's gland	0.0037%	0.0000%	0.0021%	0.0064%
530.82	Esophageal hemorrhage	0.0032%	0.0048%	0.0021%	0.0064%
625.3	Dysmenorrhea	0.0042%	0.0000%	0.0041%	0.0000%
583.89	Nephritis and nephropathy, not specified as acute or chroni	0.0037%	0.0097%	0.0021%	0.0000%
527.8	Other specified diseases of the salivary glands	0.0046%	0.0000%	0.0021%	0.0000%
529.9	Unspecified condition of the tongue	0.0042%	0.0048%	0.0021%	0.0000%
562.12	Diverticulosis of colon with hemorrhage	0.0037%	0.0000%	0.0062%	0.0000%
595.89	Other specified types of cystitis	0.0023%	0.0048%	0.0041%	0.0096%
414.9	Chronic ischemic heart disease, unspecified	0.0018%	0.0145%	0.0041%	0.0064%
471.0	Polyp of nasal cavity	0.0042%	0.0048%	0.0021%	0.0000%
474.02	Chronic tonsillitis and adenoiditis	0.0046%	0.0048%	0.0000%	0.0000%
510.0	Empyema, with fistula	0.0018%	0.0097%	0.0041%	0.0096%
380.00	Perichondritis of pinna, unspecified	0.0037%	0.0048%	0.0021%	0.0032%
442.89	Aneurysm of other specified artery	0.0037%	0.0000%	0.0062%	0.0000%
518.84	Acute and chronic respiratory failure	0.0023%	0.0048%	0.0062%	0.0064%
465.9	Acute upper respiretory infections of unspecified site	0.0023%	0.0145%	0.0021%	0.0064%
394.1	Rheumatic mitral insufficiency	0.0018%	0.0145%	0.0000%	0.0127%
385.23	Discontinuity or dislocation of ear ossicles	0.0042%	0.0000%	0.0021%	0.0032%
519.3	Other diseases of mediastinum, not elsewhere classified	0.0032%	0.0000%	0.0062%	0.0032%
519.8	Other diseases of respiratory system, not elsewhere classifi	0.0037%	0.0000%	0.0021%	0.0064%
402.91	Unspecified hypertensive heart disease with congestive hea	0.0023%	0.0048%	0.0062%	0.0064%
404.93	Unspecified hypertensive heart and renal disease with cong	0.0009%	0.0194%	0.0062%	0.0064%
276.2	Acidosis	0.0005%	0.0097%	0.0021%	0.0223%
238.4	Neoplasm of uncertain behavior of polycythemia vera	0.0032%	0.0097%	0.0021%	0.0032%

ICD-9-CM	ICD-9-CM	PureO	ContiB	NewB	PastB
355.0	Lesion of sciatic nerve	0.0014%	0.0000%	0.0083%	0.0127%
295.30	Schizophrenic disorders, paranoid type, unspecified	0.0000%	0.0194%	0.0000%	0.0223%
345.91	Unspecified epilepsy with intractable epilepsy	0.0009%	0.0000%	0.0062%	0.0191%
256.4	Polycystic ovaries	0.0051%	0.0000%	0.0000%	0.0000%
277.3	Amyloidosis	0.0023%	0.0048%	0.0062%	0.0064%
273.8	Other disorders of plasma protein metabolism	0.0014%	0.0097%	0.0041%	0.0127%
361.89	Other forms of retinal detachment	0.0042%	0.0048%	0.0000%	0.0032%
345.3	Grand mal status	0.0018%	0.0145%	0.0000%	0.0127%
216.4	Benign neoplasm of scalp and skin of neck	0.0037%	0.0000%	0.0041%	0.0032%
214.2	Lipoma of intrathoracic organs	0.0042%	0.0048%	0.0021%	0.0000%
142.2	Malignant neoplasm of sublingual gland	0.0028%	0.0000%	0.0083%	0.0032%
200.00	Reticulosarcoma, unspecified site, extranodal solid organ si	0.0009%	0.0048%	0.0145%	0.0032%
198.7	Secondary malignant neoplasm of adrenal gland	0.0009%	0.0048%	0.0083%	0.0127%
171.7	Malignant neoplasm of connective and other soft tissue of t	0.0023%	0.0097%	0.0083%	0.0000%
053.20	Herpes zoster dermatitis of eyelid	0.0023%	0.0097%	0.0041%	0.0064%
038.40	Septicemia due to Gram-negative organism, unspecified	0.0014%	0.0097%	0.0041%	0.0127%
170.4	Malignant neoplasm of scapula and long bones of upper lin	0.0032%	0.0000%	0.0083%	0.0000%
V30.00	Single liveborn, born in hospital, delivered without mentio	0.0046%	0.0000%	0.0000%	0.0000%
996.63	Infection and inflammatory reaction due to nervous system	0.0014%	0.0048%	0.0021%	0.0159%
996.68	Infection and inflammatory reaction due to peritoneal dialy	0.0009%	0.0194%	0.0000%	0.0127%
875.0	Open wound of chest(wall),without mention of complicatio	0.0042%	0.0000%	0.0021%	0.0000%
996.76	Other complications due to genitourinary device, implant, a	0.0042%	0.0048%	0.0000%	0.0000%
998.6	Persistent postoperative fistula	0.0023%	0.0097%	0.0062%	0.0000%
996.61	Infection and inflammatory reaction due to cardiac device,	0.0028%	0.0048%	0.0021%	0.0064%
927.11	Crushing injury of elbow	0.0028%	0.0048%	0.0062%	0.0000%
996.78	Other complications due to other internal orthopedic device	0.0018%	0.0048%	0.0062%	0.0064%
806.04	C1- C4 level fracture with other specified spinal cord injur	0.0014%	0.0000%	0.0145%	0.0000%
866.00	Unspecified injury to kidney, without mention of open wou	0.0042%	0.0000%	0.0000%	0.0032%
853.04	Other and unspecified intracranial hemorrhage following in	0.0023%	0.0000%	0.0083%	0.0032%
850.1	Concussion with brief loss of consciousness	0.0023%	0.0145%	0.0041%	0.0000%
811.02	Fracture in coracoid process of scapula, closed	0.0046%	0.0000%	0.0000%	0.0000%
823.31	Fracture of shaft of fibula alone, open	0.0042%	0.0000%	0.0021%	0.0000%
870.2	Laceration of eyelid involving lacrimal passages	0.0032%	0.0000%	0.0062%	0.0000%
813.93	Fracture in unspecified part of radius with ulna, open	0.0028%	0.0048%	0.0041%	0.0032%
842.13	Sprains and strains of interphalangeal (joint)	0.0037%	0.0000%	0.0021%	0.0032%
780.53	Hypersomnia with sleep apnea	0.0046%	0.0000%	0.0000%	0.0000%
747.64	Lower limb vessel anomaly	0.0046%	0.0000%	0.0000%	0.0000%
751.1	Atresia and stenosis of small intestine	0.0046%	0.0000%	0.0000%	0.0000%
727.69	Nontraumatic rupture of other tendon	0.0037%	0.0000%	0.0021%	0.0032%

ICD-9-CM	ICD-9-CM	PureO	ContiB	NewB	PastB
736.21	Boutonniere deformity	0.0005%	0.0145%	0.0103%	0.0032%
746.02	Congenital stenosis of pulmonary valve	0.0042%	0.0000%	0.0000%	0.0032%
750.3	Tracheoesophageal fistula, esophageal atresia and stenosis	0.0046%	0.0000%	0.0000%	0.0000%
743.61	Congenital ptosis	0.0046%	0.0000%	0.0000%	0.0000%
736.70	Unspecified deformity of ankle and foot, acquired	0.0042%	0.0000%	0.0021%	0.0000%
727.82	Calcium deposits in tendon and bursa	0.0028%	0.0000%	0.0041%	0.0064%
729.6	Residual foreign body in soft tissue	0.0037%	0.0000%	0.0021%	0.0032%
747.29	Other anomalies of aorta	0.0028%	0.0097%	0.0041%	0.0000%
749.14	Incomplete cleft lip, bilateral	0.0042%	0.0000%	0.0000%	0.0032%
717.0	Old bucket handle tear of medial meniscus	0.0042%	0.0000%	0.0000%	0.0032%
721.41	Thoracic spondylosis with myelopathy	0.0014%	0.0048%	0.0103%	0.0032%
717.81	Old disruption of lateral collateral ligament	0.0037%	0.0000%	0.0041%	0.0000%
711.96	Unspecified infective arthritis, lower leg	0.0023%	0.0048%	0.0062%	0.0032%
724.9	Other unspecified back disorders	0.0018%	0.0097%	0.0021%	0.0096%
715.93	Osteoarthrosis, unspecified whether generalized or localize	0.0042%	0.0048%	0.0000%	0.0000%
701.8	Other specified hypertrophic and atrophic conditions of ski	0.0032%	0.0000%	0.0041%	0.0032%
574.81	Calculus of gallbladder and bile duct with acute or chronic	0.0032%	0.0000%	0.0021%	0.0064%
524.8	Other specified dentofacial anomalies	0.0032%	0.0000%	0.0062%	0.0000%
617.2	Endometriosis of fallopian tube	0.0037%	0.0000%	0.0021%	0.0032%
593.72	Vesicoureteral reflux , with reflux nephropathy, bilateral	0.0046%	0.0000%	0.0000%	0.0000%
552.9	Hernia of unspecified site, with obstruction	0.0032%	0.0000%	0.0021%	0.0064%
610.0	Solitary cyst of breast	0.0028%	0.0097%	0.0041%	0.0000%
569.62	Mechanical complication of colostomy and enterostomy	0.0032%	0.0000%	0.0021%	0.0064%
562.02	Diverticulosis of small intestine with hemorrhage	0.0032%	0.0000%	0.0041%	0.0032%
567.8	Other specified peritonitis	0.0023%	0.0000%	0.0062%	0.0064%
611.79	Other signs and symptoms in breast	0.0046%	0.0000%	0.0000%	0.0000%
518.83	Chronic respiratory failure	0.0009%	0.0145%	0.0021%	0.0127%
520.1	Supernumerary teeth	0.0042%	0.0048%	0.0000%	0.0000%
398.91	Rheumatic heart failure (congestive)	0.0014%	0.0097%	0.0062%	0.0064%
518.5	Pulmonary insufficiency following trauma and surgery	0.0023%	0.0048%	0.0062%	0.0032%
458.8	Other specified hypotension	0.0028%	0.0048%	0.0062%	0.0000%
410.51	Acute myocardial infarction of other lateral wall, initial epi	0.0018%	0.0000%	0.0103%	0.0032%
425.1	Hypertrophic obstructive cardiomyopathy	0.0028%	0.0048%	0.0041%	0.0032%
231.0	Carcinoma in situ of larynx	0.0037%	0.0048%	0.0000%	0.0032%
345.10	Generalized convulsive epilepsy without mention of intract	0.0009%	0.0145%	0.0041%	0.0096%
251.2	Hypoglycemia, unspecified	0.0014%	0.0097%	0.0041%	0.0096%
211.2	Benign neoplasm of duodenum, jejunum, and ileum	0.0037%	0.0048%	0.0000%	0.0032%
255.9	Unspecified disorder of adrenal glands	0.0032%	0.0048%	0.0021%	0.0032%
216.6	Benign neoplasm of skin of upper limb, including shoulder	0.0046%	0.0000%	0.0000%	0.0000%

ICD-9-CM	ICD-9-CM	PureO	ContiB	NewB	PastB
215.7	Benign neoplasm of connective and other soft tissue of trur	0.0042%	0.0048%	0.0000%	0.0000%
288.3	Eosinophilia	0.0023%	0.0048%	0.0062%	0.0032%
344.60	Cauda equina syndrome without mention of neurogenic bla	0.0009%	0.0194%	0.0062%	0.0032%
275.42	Disorders of calcium metabolism, Hypercalcemia	0.0023%	0.0097%	0.0021%	0.0064%
213.6	Benign neoplasm of pelvic bones, sacrum and coccyx	0.0037%	0.0048%	0.0000%	0.0032%
272.6	Lipodystrophy	0.0028%	0.0048%	0.0041%	0.0032%
215.4	Benign neoplasm of connective and other soft tissue of tho	0.0037%	0.0000%	0.0041%	0.0000%
276.6	Fluid overload	0.0009%	0.0048%	0.0041%	0.0159%
277.1	Disorders of porphyrin metabolism	0.0018%	0.0000%	0.0103%	0.0032%
274.9	Gout, unspecified	0.0009%	0.0097%	0.0062%	0.0096%
202.87	Other lymphomas, spleen	0.0028%	0.0048%	0.0041%	0.0032%
172.5	Malignant melanoma of skin of trunk, except scrotum	0.0014%	0.0097%	0.0103%	0.0000%
013.06	Tuberculous meningitis, tubercle bacilli not found by bacte	0.0023%	0.0048%	0.0083%	0.0000%
148.3	Malignant neoplasm of posterior hypopharyngeal wall	0.0018%	0.0000%	0.0103%	0.0032%
201.51	Nodular sclerosis, lymph nodes of head, face, and neck	0.0046%	0.0000%	0.0000%	0.0000%
049.9	Unspecified non-arthropod-borne viral diseases of central n	0.0028%	0.0097%	0.0041%	0.0000%
188.7	Malignant neoplasm of urachus	0.0028%	0.0145%	0.0021%	0.0000%
181	Malignant neoplasm of placenta	0.0018%	0.0145%	0.0021%	0.0064%
204.01	Acute lymphoid leukemia, in remission	0.0018%	0.0194%	0.0041%	0.0000%
053.12	Postherpetic trigeminal neuralgia	0.0005%	0.0145%	0.0041%	0.0127%
192.1	Malignant neoplasm of cerebral meninges	0.0037%	0.0048%	0.0021%	0.0000%
188.3	Malignant neoplasm of anterior wall of urinary bladder	0.0028%	0.0097%	0.0021%	0.0032%
070.54	Chronic hepatitis C without mention of hepatic coma	0.0009%	0.0097%	0.0021%	0.0159%
078.5	Cytomegaloviral disease	0.0018%	0.0145%	0.0041%	0.0032%
151.6	Malignant neoplasm of greater curvature of stomach, unspe	0.0028%	0.0097%	0.0041%	0.0000%
017.95	Tuberculosis of other specified organ, tubercle bacilli not f	0.0032%	0.0000%	0.0021%	0.0064%
884.2	Multiple and unspecified open wound of upper limb, with t	0.0037%	0.0000%	0.0000%	0.0032%
903.1	Injury to brachial blood vessels	0.0032%	0.0000%	0.0041%	0.0000%
943.33	Burn of upper arm, full-thickness skin loss (third degree N	0.0032%	0.0048%	0.0021%	0.0000%
927.9	Crushing injury of upper limb, unspecified site	0.0028%	0.0048%	0.0041%	0.0000%
937	Foreign body in anus and rectum	0.0037%	0.0000%	0.0021%	0.0000%
959.4	Injury of hand, except finger	0.0032%	0.0000%	0.0041%	0.0000%
941.31	Burn of ear (any part), full-thickness skin loss (third degree	0.0032%	0.0048%	0.0021%	0.0000%
944.33	Burn two or more digits, not including thumb, full-thicknes	0.0032%	0.0097%	0.0000%	0.0000%
917.0	Superficial injury of foot and toe(s), abrasion or friction bu	0.0037%	0.0000%	0.0021%	0.0000%
851.84	Other and unspecified cerebral laceration and contusion wi	0.0018%	0.0000%	0.0103%	0.0000%
802.30	Fracture of mandible, open, unspecified site	0.0032%	0.0000%	0.0041%	0.0000%
861.32	Laceration of lung,with open wound into thorax	0.0028%	0.0000%	0.0062%	0.0000%
862.8	Injury to multiple and unspecified intrathoracic organs,with	0.0028%	0.0000%	0.0062%	0.0000%

ICD-9-CM	ICD-9-CM	PureO	ContiB	NewB	PastB
851.82	Other and unspecified cerebral laceration and contusion wi	0.0028%	0.0000%	0.0062%	0.0000%
812.20	Fracture of unspecified part of humerus, closed	0.0018%	0.0048%	0.0021%	0.0096%
870.0	Laceration of skin of eyelid and periocular area	0.0042%	0.0000%	0.0000%	0.0000%
802.27	Fracture of mandible, closed, alveolar border of body	0.0042%	0.0000%	0.0000%	0.0000%
861.22	Laceration of lung,without mention of open wound into tho	0.0028%	0.0000%	0.0062%	0.0000%
839.06	Dislocations of sixth cervical vertebra, closed	0.0005%	0.0097%	0.0103%	0.0032%
813.81	Fracture in unspecified part of radius (alone), closed	0.0032%	0.0000%	0.0041%	0.0000%
873.1	Other open wound of scalp, complicated	0.0014%	0.0048%	0.0083%	0.0032%
810.01	Fracture in sternal end of clavicle, closed	0.0037%	0.0000%	0.0021%	0.0000%
816.10	Unspecified fracture of phalanx or phalanges, open	0.0018%	0.0048%	0.0083%	0.0000%
800.51	Fracture of vault of skull, open without mention of intracra	0.0032%	0.0000%	0.0041%	0.0000%
752.3	Other anomalies of uterus	0.0042%	0.0000%	0.0000%	0.0000%
751.7	Anomalies of pancreas	0.0032%	0.0000%	0.0041%	0.0000%
733.20	Cyst of bone (localized), unspecified	0.0037%	0.0000%	0.0021%	0.0000%
728.12	Traumatic myositis ossificans	0.0032%	0.0048%	0.0000%	0.0032%
789.01	Abdominal pain, right upper quadrant	0.0023%	0.0048%	0.0041%	0.0032%
746.83	Infundibular pulmonic stenosis	0.0037%	0.0000%	0.0021%	0.0000%
727.62	Rupture of tendons of biceps (long head)	0.0023%	0.0048%	0.0021%	0.0064%
785.51	Cardiogenic shock	0.0028%	0.0048%	0.0041%	0.0000%
781.2	Abnormality of gait	0.0028%	0.0097%	0.0021%	0.0000%
746.6	Congenital mitral insufficiency	0.0018%	0.0048%	0.0041%	0.0064%
745.60	Endocardial cushion defect, unspecified type	0.0042%	0.0000%	0.0000%	0.0000%
787.03	Vomiting alone	0.0023%	0.0000%	0.0041%	0.0064%
755.59	Other anomalies of upper limb, including shoulder girdle	0.0032%	0.0048%	0.0021%	0.0000%
755.64	Congenital deformity of knee (joint)	0.0037%	0.0000%	0.0000%	0.0032%
719.85	Other specified disorders of joint, pelvic region and thigh	0.0028%	0.0000%	0.0062%	0.0000%
686.8	Other specified local infections of skin and subcutaneous ti	0.0037%	0.0000%	0.0021%	0.0000%
722.92	Thoracic intervertebral disc disorder	0.0018%	0.0000%	0.0083%	0.0032%
648.93	Other current conditions classifiable elsewhere in the moth	0.0032%	0.0000%	0.0000%	0.0064%
692.9	Contact dermatitis and other eczema, unspecified cause	0.0028%	0.0048%	0.0021%	0.0032%
718.12	Loose body in joint, upper arm	0.0037%	0.0048%	0.0000%	0.0000%
716.93	Arthropathy, unspecified, forearm	0.0037%	0.0000%	0.0000%	0.0032%
707.14	Ulcer of heel and midfoot	0.0014%	0.0048%	0.0021%	0.0127%
724.5	Backache, unspecified	0.0009%	0.0097%	0.0041%	0.0096%
718.52	Ankylosis of joint, upper arm	0.0042%	0.0000%	0.0000%	0.0000%
714.30	Polyarticular juvenile rheumatoid arthritis, chronic or unspe	0.0028%	0.0048%	0.0041%	0.0000%
726.79	Other enthesopathy of ankle and tarsus	0.0037%	0.0000%	0.0021%	0.0000%
696.0	Psoriatic arthropathy	0.0014%	0.0097%	0.0062%	0.0032%
720.2	Sacroiliitis, not elsewhere classified	0.0014%	0.0097%	0.0062%	0.0032%

ICD-9-CM	ICD-9-CM	PureO	ContiB	NewB	PastB
535.10	Atrophic gastritis, without mention of hemorrhage	0.0023%	0.0000%	0.0062%	0.0032%
523.3	Acute periodontitis	0.0037%	0.0048%	0.0000%	0.0000%
569.3	Hemorrhage of rectum and anus	0.0023%	0.0000%	0.0021%	0.0096%
531.91	Gastric ulcer, unspecified as acute or chronic, without men	0.0037%	0.0000%	0.0000%	0.0032%
599.84	Other specified disorders of urethra	0.0028%	0.0048%	0.0041%	0.0000%
626.8	Other disorders of menstruation and other abnormal bleedi	0.0037%	0.0000%	0.0000%	0.0032%
558.1	Gastroenteritis and colitis due to radiation	0.0023%	0.0048%	0.0021%	0.0064%
537.3	Other obstruction of duodenum	0.0028%	0.0000%	0.0041%	0.0032%
524.19	Other specified anomaly of relationship of jaw to cranial ba	0.0042%	0.0000%	0.0000%	0.0000%
530.19	Other esophagitis	0.0018%	0.0048%	0.0041%	0.0064%
531.10	Gastric ulcer, acute with perforation, without mention of ol	0.0037%	0.0000%	0.0021%	0.0000%
630	Hydatidiform mole	0.0032%	0.0000%	0.0041%	0.0000%
641.13	Hemorrhage from placenta previa, antepartum condition or	0.0032%	0.0000%	0.0021%	0.0032%
631	Other abnormal product of conception	0.0037%	0.0000%	0.0021%	0.0000%
617.4	Endometriosis of rectovaginal septum and vagina	0.0037%	0.0000%	0.0021%	0.0000%
531.00	Gastric ulcer, acute with hemorrhage, without mention of c	0.0014%	0.0097%	0.0062%	0.0032%
532.70	Duodenal ulcer, chronic without mention of hemorrhage or	0.0018%	0.0048%	0.0041%	0.0064%
599.3	Urethral caruncle	0.0032%	0.0048%	0.0021%	0.0000%
381.10	Chronic serous otitis media, simple or unspecified	0.0028%	0.0000%	0.0041%	0.0032%
416.0	Primary pulmonary hypertension	0.0018%	0.0048%	0.0000%	0.0127%
378.54	Sixth or abducens nerve palsy	0.0028%	0.0097%	0.0021%	0.0000%
457.8	Other noninfectious disorders of lymphatic channels	0.0032%	0.0048%	0.0021%	0.0000%
378.17	Alternating exotropia with V pattern	0.0042%	0.0000%	0.0000%	0.0000%
443.0	Raynaud's syndrome	0.0023%	0.0048%	0.0062%	0.0000%
457.0	Postmastectomy lymphedema syndrome	0.0005%	0.0048%	0.0083%	0.0096%
483.0	Pneumonia due to Mycoplasma pneumoniae	0.0028%	0.0000%	0.0041%	0.0032%
429.89	Other ill-defined heart diseases	0.0014%	0.0048%	0.0062%	0.0064%
516.8	Other specified alveolar and parietoalveolar pneumonopath	0.0014%	0.0145%	0.0062%	0.0000%
518.3	Pulmonary eosinophilia	0.0028%	0.0000%	0.0041%	0.0032%
443.1	Thromboangiitis obliterans [Buerger's disease]	0.0023%	0.0000%	0.0062%	0.0032%
238.2	Neoplasm of uncertain behavior of skin	0.0037%	0.0000%	0.0000%	0.0032%
354.9	Mononeuritis of upper limb, unspecified	0.0028%	0.0048%	0.0041%	0.0000%
211.0	Benign neoplasm of esophagus	0.0032%	0.0000%	0.0021%	0.0032%
256.1	Other ovarian hyperfunction	0.0028%	0.0000%	0.0000%	0.0096%
349.89	Other specified disorders of nervous system	0.0018%	0.0048%	0.0083%	0.0000%
272.8	Other disorders of lipoid metabolism	0.0014%	0.0048%	0.0041%	0.0096%
231.2	Carcinoma in situ of bronchus and lung	0.0023%	0.0048%	0.0041%	0.0032%
210.6	Benign neoplasm of other parts of oropharynx	0.0037%	0.0000%	0.0000%	0.0032%
211.7	Benign neoplasm of islets of Langerhans	0.0032%	0.0000%	0.0021%	0.0032%

ICD-9-CM	ICD-9-CM	PureO	ContiB	NewB	PastB
112.2	Candidiasis of other urogenital sites	0.0009%	0.0145%	0.0021%	0.0096%
162.0	Malignant neoplasm of trachea	0.0014%	0.0048%	0.0083%	0.0032%
202.82	Other lymphomas, intrathoracic lymph nodes	0.0023%	0.0097%	0.0041%	0.0000%
012.02	Tuberculous pleurisy, bacteriological or histological exami	0.0023%	0.0000%	0.0021%	0.0096%
170.2	Malignant neoplasm of vertebral column, excluding sacrum	0.0032%	0.0048%	0.0021%	0.0000%
154.2	Malignant neoplasm of anal canal	0.0023%	0.0000%	0.0083%	0.0000%
150.1	Malignant neoplasm of thoracic esophagus	0.0018%	0.0000%	0.0103%	0.0000%
200.20	Burkitt's tumor or lymphoma, unspecified site, extranodal s	0.0018%	0.0048%	0.0083%	0.0000%
195.3	Malignant neoplasm of other and ill-defined sites of pelvis	0.0009%	0.0048%	0.0083%	0.0064%
190.5	Malignant neoplasm of retina	0.0042%	0.0000%	0.0000%	0.0000%
154.8	Malignant neoplasm of rectum, rectosigmoid junction, and	0.0018%	0.0097%	0.0041%	0.0032%
191.3	Malignant neoplasm of parietal lobe	0.0009%	0.0097%	0.0083%	0.0032%
955.9	Injury to unspecified nerve of shoulder girdle and upper lin	0.0028%	0.0000%	0.0021%	0.0032%
941.22	Burn of eye (with other parts of face, head and neck), bliste	0.0014%	0.0048%	0.0062%	0.0032%
902.53	Injury to iliac artery	0.0014%	0.0048%	0.0062%	0.0032%
924.00	Contusion of thigh	0.0014%	0.0048%	0.0083%	0.0000%
996.89	Complications of other specified transplanted organ	0.0037%	0.0000%	0.0000%	0.0000%
V59.8	Donors of other specified organ or tissue	0.0028%	0.0000%	0.0021%	0.0032%
900.82	Injury to multiple blood vessels of head and neck	0.0014%	0.0048%	0.0062%	0.0032%
880.00	Open wound of shoulder region, without mention of compl	0.0032%	0.0048%	0.0000%	0.0000%
944.35	Burn of palm, full-thickness skin loss (third degree NOS)	0.0028%	0.0000%	0.0041%	0.0000%
880.02	Open wound of axillary region, without mention of complic	0.0028%	0.0000%	0.0021%	0.0032%
998.13	Seroma complicating a procedure	0.0032%	0.0048%	0.0000%	0.0000%
934.1	Foreign body in main bronchus	0.0028%	0.0048%	0.0000%	0.0032%
885.1	Traumatic amputation of thumb (complete) (partial), compl	0.0018%	0.0097%	0.0041%	0.0000%
983.1	Toxic effect of acids	0.0018%	0.0048%	0.0041%	0.0032%
958.7	Traumatic subcutaneous emphysema	0.0018%	0.0000%	0.0062%	0.0032%
933.0	Foreign body in pharynx	0.0028%	0.0000%	0.0021%	0.0032%
873.53	Open wound of lip, complicated	0.0028%	0.0000%	0.0041%	0.0000%
834.01	Closed dislocation of metacarpophalangeal (joint)	0.0028%	0.0000%	0.0021%	0.0032%
833.09	Other closed dislocation of wrist	0.0032%	0.0000%	0.0021%	0.0000%
806.26	T7- T12 level fracture with complete lesion of cord, closed	0.0009%	0.0000%	0.0124%	0.0000%
861.00	Unspecified injury to heart,without mention of open wound	0.0028%	0.0000%	0.0041%	0.0000%
805.07	Fracture of seventh cervical vertebra, closed	0.0028%	0.0048%	0.0021%	0.0000%
820.01	Fracture of epiphysis (separation) (upper)of femur, closed	0.0028%	0.0000%	0.0021%	0.0032%
823.91	Fracture of unspecified part of fibula alone, open	0.0014%	0.0000%	0.0103%	0.0000%
873.41	Open wound of cheek, without mention of complication	0.0023%	0.0000%	0.0062%	0.0000%
823.81	Fracture of unspecified part of fibula alone, closed	0.0037%	0.0000%	0.0000%	0.0000%
805.03	Fracture of third cervical vertebra, closed	0.0023%	0.0048%	0.0021%	0.0032%

ICD-9-CM	ICD-9-CM	PureO	ContiB	NewB	PastB
841.1	Sprains and strains of ulnar collateral ligament	0.0023%	0.0000%	0.0021%	0.0064%
845.00	Sprains and strains of ankle,unspecified site	0.0023%	0.0000%	0.0000%	0.0096%
871.4	Unspecified laceration of eye	0.0028%	0.0000%	0.0041%	0.0000%
863.42	Injury to transverse colon,without mention of open wound	0.0032%	0.0000%	0.0021%	0.0000%
751.4	Anomalies of intestinal fixation	0.0037%	0.0000%	0.0000%	0.0000%
747.62	Renal vessel anomaly	0.0028%	0.0048%	0.0021%	0.0000%
748.8	Other specified anomalies of respiratory system	0.0028%	0.0000%	0.0021%	0.0032%
729.5	Pain in limb	0.0005%	0.0048%	0.0062%	0.0096%
747.82	Spinal vessel anomaly	0.0014%	0.0000%	0.0103%	0.0000%
727.64	Rupture of flexor tendons of hand and wrist	0.0018%	0.0048%	0.0041%	0.0032%
736.72	Equinus deformity of foot, acquired	0.0028%	0.0097%	0.0000%	0.0000%
753.13	Polycystic kidney, autosomal dominant	0.0014%	0.0097%	0.0041%	0.0032%
732.3	Juvenile osteochondrosis of upper extremity	0.0037%	0.0000%	0.0000%	0.0000%
738.5	Other acquired deformity of back or spine	0.0028%	0.0048%	0.0021%	0.0000%
759.0	Anomalies of spleen	0.0032%	0.0048%	0.0000%	0.0000%
746.85	Coronary artery anomaly	0.0005%	0.0048%	0.0083%	0.0064%
733.19	Pathologic fracture of other specified site	0.0023%	0.0048%	0.0041%	0.0000%
717.9	Unspecified internal derangement of knee	0.0018%	0.0048%	0.0021%	0.0064%
722.51	Degeneration of thoracic or thoracolumbar intervertebral di	0.0032%	0.0000%	0.0021%	0.0000%
664.51	Vulval and perineal hematoma, delivered, with or without	0.0037%	0.0000%	0.0000%	0.0000%
651.31	Twin pregnancy with fetal loss and retention of one fetus,	0.0037%	0.0000%	0.0000%	0.0000%
719.67	Other symptoms referable to joint, ankle and foot	0.0023%	0.0048%	0.0021%	0.0032%
715.22	Osteoarthrosis, localized, secondary, upper arm	0.0028%	0.0000%	0.0021%	0.0032%
717.1	Derangement of anterior horn of medial meniscus	0.0018%	0.0000%	0.0041%	0.0064%
657.01	Polyhydramnios, delivered, with or without mention of ant	0.0032%	0.0000%	0.0021%	0.0000%
726.32	Lateral epicondylitis	0.0032%	0.0000%	0.0000%	0.0032%
682.9	Other cellulitis and abscess, unspecified site	0.0014%	0.0000%	0.0062%	0.0064%
658.13	Premature rupture of membranes, antepartum condition or	0.0037%	0.0000%	0.0000%	0.0000%
719.45	Pain in joint, pelvic region and thigh	0.0018%	0.0048%	0.0021%	0.0064%
719.16	Hemarthrosis, lower leg	0.0014%	0.0048%	0.0021%	0.0096%
597.0	Urethral abscess	0.0023%	0.0000%	0.0062%	0.0000%
532.60	Duodenal ulcer, chronic or unspecified with hemorrhage an	0.0032%	0.0000%	0.0000%	0.0032%
641.81	Other antepartum hemorrhage, delivered, with or without n	0.0032%	0.0000%	0.0000%	0.0032%
587	Renal sclerosis, unspecified	0.0028%	0.0000%	0.0021%	0.0032%
550.13	Inguinal hernia, with obstruction, without mention of gang	0.0032%	0.0048%	0.0000%	0.0000%
590.00	Chronic pyelonephritis without lesion of renal medullary n	0.0023%	0.0048%	0.0000%	0.0064%
569.5	Abscess of intestine	0.0032%	0.0000%	0.0000%	0.0032%
527.1	Hypertrophy of salivary gland	0.0023%	0.0000%	0.0021%	0.0064%
614.3	Acute parametritis and pelvic cellulitis	0.0028%	0.0000%	0.0041%	0.0000%

ICD-9-CM	ICD-9-CM	PureO	ContiB	NewB	PastB
562.13	Diverticulitis of colon with hemorrhage	0.0037%	0.0000%	0.0000%	0.0000%
366.20	Traumatic cataract, unspecified	0.0032%	0.0048%	0.0000%	0.0000%
381.4	Nonsuppurative otitis media, not specified as acute or chro	0.0037%	0.0000%	0.0000%	0.0000%
494.1	Bronchiectasis with acute exacerbation	0.0014%	0.0145%	0.0000%	0.0064%
490	Bronchitis, not specified as acute or chronic	0.0018%	0.0000%	0.0021%	0.0096%
389.00	Conductive hearing loss, unspecified	0.0037%	0.0000%	0.0000%	0.0000%
380.32	Acquired deformities of auricle or pinna	0.0032%	0.0000%	0.0021%	0.0000%
452	Portal vein thrombosis	0.0028%	0.0000%	0.0000%	0.0064%
410.21	Acute myocardial infarction of inferolateral wall, initial epi	0.0028%	0.0000%	0.0041%	0.0000%
371.40	Corneal degeneration, unspecified	0.0032%	0.0000%	0.0021%	0.0000%
459.81	Venous (peripheral) insufficiency, unspecified	0.0032%	0.0048%	0.0000%	0.0000%
374.00	Entropion, unspecified	0.0032%	0.0048%	0.0000%	0.0000%
440.23	Atherosclerosis of the extremities with ulceration	0.0023%	0.0048%	0.0041%	0.0000%
446.4	Wegener's granulomatosis	0.0014%	0.0145%	0.0021%	0.0032%
296.34	Major depressive disorder, recurrent episode, severe specifi	0.0000%	0.0000%	0.0021%	0.0223%
221.8	Benign neoplasm f other specified sites of female genital o	0.0028%	0.0048%	0.0021%	0.0000%
253.8	Other disorders of the pituitary and other syndromes of die	0.0014%	0.0000%	0.0062%	0.0064%
355.9	Mononeuritis of unspecified site	0.0009%	0.0097%	0.0062%	0.0032%
295.70	Schizo-affective type, unspecified	0.0000%	0.0097%	0.0000%	0.0191%
233.7	Carcinoma in situ of bladder	0.0005%	0.0048%	0.0041%	0.0127%
212.4	Benign neoplasm of pleura	0.0023%	0.0048%	0.0000%	0.0064%
348.4	Compression of brain	0.0018%	0.0000%	0.0062%	0.0032%
359.9	Myopathy, unspecified	0.0014%	0.0048%	0.0083%	0.0000%
223.3	Benign neoplasm of bladder	0.0028%	0.0000%	0.0041%	0.0000%
053.79	Herpes zoster with other specified complications	0.0009%	0.0048%	0.0062%	0.0064%
152.8	Malignant neoplasm of other specified sites of small intesti	0.0023%	0.0000%	0.0041%	0.0032%
038.10	Staphylococcal septicemia, unspecified	0.0014%	0.0048%	0.0021%	0.0096%
070.9	Unspecified viral hepatitis without mention of hepatic com	0.0037%	0.0000%	0.0000%	0.0000%
146.2	Malignant neoplasm of tonsillar pillars (anterior) (posterior	0.0018%	0.0000%	0.0062%	0.0032%
031.8	Other specified mycobacteria diseases	0.0028%	0.0000%	0.0000%	0.0064%
198.82	Secondary malignant neoplasm of genital organs	0.0028%	0.0000%	0.0041%	0.0000%
190.1	Malignant neoplasm of orbit	0.0032%	0.0048%	0.0000%	0.0000%
173.1	Malignant neoplasm of skin of eyelid, including canthus	0.0037%	0.0000%	0.0000%	0.0000%
031.9	Unspecified diseases due to mycobacteria	0.0018%	0.0000%	0.0062%	0.0032%
191.5	Malignant neoplasm of ventricles	0.0028%	0.0000%	0.0041%	0.0000%
172.4	Malignant melanoma of skin of scalp and neck	0.0009%	0.0000%	0.0103%	0.0032%
943.23	Burn of upper arm, blisters, epidermal loss (second degree)	0.0032%	0.0000%	0.0000%	0.0000%
995.0	Other anaphylactic shock	0.0023%	0.0000%	0.0021%	0.0032%
881.11	Open wound of elbow, complicated	0.0028%	0.0000%	0.0021%	0.0000%

ICD-9-CM	ICD-9-CM	PureO	ContiB	NewB	PastB
925.1	Crushing injury of face and scalp	0.0023%	0.0000%	0.0021%	0.0032%
884.1	Multiple and unspecified open wound of upper limb, comp	0.0018%	0.0000%	0.0041%	0.0032%
956.5	Injury to other specified nerve(s) of pelvic girdle and lowe	0.0023%	0.0000%	0.0041%	0.0000%
943.32	Burn of elbow, full-thickness skin loss (third degree NOS)	0.0018%	0.0000%	0.0021%	0.0064%
944.28	Burn of multiple sites of wrist(s) and hand(s), blisters, epid	0.0028%	0.0000%	0.0021%	0.0000%
944.31	Burn of single digit (finger nail) other than thumb, full-thic	0.0028%	0.0000%	0.0021%	0.0000%
808.51	Fracture of ilium, open	0.0023%	0.0000%	0.0041%	0.0000%
813.13	Monteggia's fracture,open	0.0032%	0.0000%	0.0000%	0.0000%
865.01	Hematoma without rupture of capsule in spleen, without m	0.0018%	0.0000%	0.0041%	0.0032%
807.5	Fracture of larynx and trachea, closed	0.0032%	0.0000%	0.0000%	0.0000%
806.08	C5- C7 level fracture with central cord syndrome, closed	0.0023%	0.0000%	0.0041%	0.0000%
842.19	Sprains and strains of other specified site, hand	0.0028%	0.0000%	0.0021%	0.0000%
833.04	Closed dislocation of carpometacarpal (joint)	0.0014%	0.0048%	0.0062%	0.0000%
871.9	Unspecified open wound of eyeball	0.0028%	0.0000%	0.0021%	0.0000%
852.44	Extradural hemorrhage following injury without mention o	0.0014%	0.0000%	0.0062%	0.0032%
825.24	Fracture of cuneiform of foot, closed	0.0032%	0.0000%	0.0000%	0.0000%
831.01	Closed anterior dislocation of humerus	0.0018%	0.0048%	0.0041%	0.0000%
872.8	Open wound to unspecified part of ear, without mention of	0.0009%	0.0048%	0.0062%	0.0032%
814.00	Unspecified fracture of carpal bone, closed	0.0032%	0.0000%	0.0000%	0.0000%
813.18	Fracture of radius with ulna, upper end (any part), open	0.0014%	0.0048%	0.0062%	0.0000%
868.13	Injury to peritoneum with open wound into cavity	0.0023%	0.0000%	0.0041%	0.0000%
850.0	Concussion with no loss of consciousness	0.0018%	0.0000%	0.0062%	0.0000%
851.80	Other and unspecified cerebral laceration and contusion wi	0.0018%	0.0000%	0.0062%	0.0000%
833.00	Closed dislocation of unspecified part of wrist	0.0028%	0.0000%	0.0000%	0.0032%
753.3	Other specified anomalies of kidney	0.0032%	0.0000%	0.0000%	0.0000%
757.33	Congenital pigmentary anomalies of skin	0.0032%	0.0000%	0.0000%	0.0000%
755.69	Other anomalies of lower limb, including pelvic girdle	0.0018%	0.0000%	0.0041%	0.0032%
749.11	Complete cleft lip, unilateral	0.0032%	0.0000%	0.0000%	0.0000%
728.71	Plantar fascial fibromatosis	0.0028%	0.0000%	0.0000%	0.0032%
730.08	Acute osteomyelitis, other specified sites	0.0005%	0.0097%	0.0021%	0.0096%
752.63	Congenital chordee	0.0032%	0.0000%	0.0000%	0.0000%
754.82	Pectus carinatum	0.0032%	0.0000%	0.0000%	0.0000%
782.0	Disturbance of skin sensation	0.0009%	0.0097%	0.0062%	0.0000%
770.8	Other respiratory problem after birth	0.0032%	0.0000%	0.0000%	0.0000%
745.11	Double outlet right ventricle	0.0032%	0.0000%	0.0000%	0.0000%
652.41	Face or brow presentation, delivered, with or without ment	0.0028%	0.0000%	0.0000%	0.0032%
715.89	Osteoarthrosis, involving or with mention of more than one	0.0028%	0.0048%	0.0000%	0.0000%
716.94	Arthropathy, unspecified, hand	0.0028%	0.0000%	0.0021%	0.0000%
653.01	Major abnormality of bony pelvis, not further specified, de	0.0028%	0.0048%	0.0000%	0.0000%

ICD-9-CM	ICD-9-CM	PureO	ContiB	NewB	PastB
715.96	Osteoarthrosis, unspecified whether generalized or localize	0.0018%	0.0048%	0.0021%	0.0032%
653.51	Unusually large fetus causing disproportion, delivered, witl	0.0028%	0.0000%	0.0021%	0.0000%
675.14	Abscess of breast associated with childbirth, postpartum co	0.0032%	0.0000%	0.0000%	0.0000%
710.4	Polymyositis	0.0028%	0.0000%	0.0021%	0.0000%
719.86	Other specified disorders of joint, lower leg	0.0014%	0.0097%	0.0041%	0.0000%
717.82	Old disruption of medial collateral ligament	0.0028%	0.0000%	0.0000%	0.0032%
663.11	Cord around neck, with compression, delivered, with or wi	0.0028%	0.0000%	0.0021%	0.0000%
707.12	Ulcer of calf	0.0018%	0.0000%	0.0041%	0.0032%
724.00	Spinal stenosis, unspecified region	0.0005%	0.0194%	0.0041%	0.0000%
714.9	Unspecified inflammatory polyarthropathy	0.0023%	0.0097%	0.0000%	0.0000%
718.37	Recurrent dislocation of joint, ankle and foot	0.0023%	0.0000%	0.0041%	0.0000%
727.42	Ganglion of tendon sheath	0.0023%	0.0000%	0.0000%	0.0064%
637.91	Unspecified abortion, without mention of complication, inc	0.0032%	0.0000%	0.0000%	0.0000%
622.5	Incompetence of cervix	0.0032%	0.0000%	0.0000%	0.0000%
601.2	Abscess of prostate	0.0014%	0.0048%	0.0041%	0.0032%
557.1	Chronic vascular insufficiency of intestine	0.0014%	0.0048%	0.0021%	0.0064%
582.9	Chronic glomerulonephritis with unspecified pathological l	0.0014%	0.0145%	0.0021%	0.0000%
568.9	Unspecified disorder of peritoneum	0.0014%	0.0000%	0.0021%	0.0096%
600.2	Benign localized hyperplasia of prostate	0.0023%	0.0000%	0.0000%	0.0064%
593.0	Nephroptosis	0.0023%	0.0000%	0.0021%	0.0032%
595.81	Cystitis cystica	0.0018%	0.0000%	0.0021%	0.0064%
373.13	Abscess of eyelid	0.0014%	0.0048%	0.0062%	0.0000%
518.0	Pulmonary collapse	0.0023%	0.0048%	0.0000%	0.0032%
442.81	Aneurysm of artery of neck	0.0009%	0.0000%	0.0021%	0.0127%
365.60	Glaucoma associated with unspecified ocular disorder	0.0023%	0.0000%	0.0000%	0.0064%
459.89	Other specified disorders of circulatory system	0.0009%	0.0145%	0.0041%	0.0000%
508.0	Acute pulmonary manifestations due to radiation	0.0005%	0.0145%	0.0021%	0.0064%
466.19	Acute bronchiolitis due to other infectious organisms	0.0018%	0.0097%	0.0000%	0.0032%
456.1	Esophageal varices without mention of bleeding	0.0023%	0.0097%	0.0000%	0.0000%
416.8	Other chronic pulmonary heart diseases	0.0009%	0.0000%	0.0062%	0.0064%
433.01	Occlusion and stenosis of basilar artery with cerebral infarc	0.0009%	0.0097%	0.0041%	0.0032%
437.5	Moyamoya disease	0.0018%	0.0048%	0.0000%	0.0064%
380.50	Acquired stenosis of external ear canal, unspecified as to ca	0.0028%	0.0000%	0.0021%	0.0000%
459.9	Unspecified circulatory system disorder	0.0014%	0.0048%	0.0021%	0.0064%
414.8	Other specified forms of chronic ischemic heart disease	0.0014%	0.0048%	0.0000%	0.0096%
442.83	Aneurysm of splenic artery	0.0023%	0.0000%	0.0041%	0.0000%
361.05	Recent retinal detachment, total or subtotal	0.0028%	0.0000%	0.0021%	0.0000%
325	Phlebitis and thrombophlebitis of intracranial venous sinus	0.0023%	0.0000%	0.0021%	0.0032%
235.5	Neoplasm of uncertain behavior of other and unspecified di	0.0023%	0.0000%	0.0021%	0.0032%

ICD-9-CM	ICD-9-CM	PureO	ContiB	NewB	PastB
210.9	Benign neoplasm of pharynx, unspecified	0.0032%	0.0000%	0.0000%	0.0000%
237.3	Neoplasm of uncertain behavior of paraganglia	0.0028%	0.0000%	0.0000%	0.0032%
289.51	Chronic congestive splenomegaly	0.0023%	0.0000%	0.0021%	0.0032%
355.5	Tarsal tunnel syndrome	0.0018%	0.0000%	0.0041%	0.0032%
291.81	Alcohol withdrawal	0.0005%	0.0145%	0.0021%	0.0064%
341.0	Neuromyelitis optica	0.0005%	0.0194%	0.0041%	0.0000%
003.0	Salmonella gastroenteritis	0.0009%	0.0048%	0.0000%	0.0127%
144.0	Malignant neoplasm of floor of mouth, anterior portion	0.0000%	0.0097%	0.0103%	0.0000%
201.58	Nodular sclerosis, lymph nodes of multiple sites	0.0023%	0.0048%	0.0021%	0.0000%
189.3	Malignant neoplasm of urethra	0.0023%	0.0048%	0.0021%	0.0000%
075	Infectious mononucleosis	0.0023%	0.0097%	0.0000%	0.0000%
070.51	Hepatitis C without mention of hepatic coma, acute or unsp	0.0009%	0.0145%	0.0041%	0.0000%
201.50	Nodular sclerosis, unspecified site, extranodal solid organ s	0.0014%	0.0145%	0.0021%	0.0000%
195.2	Malignant neoplasm of other and ill-defined sites of abdom	0.0018%	0.0048%	0.0041%	0.0000%
052.9	Varicella without mention of complication	0.0023%	0.0000%	0.0000%	0.0064%
172.6	Malignant melanoma of skin of upper limb including shoul	0.0018%	0.0048%	0.0041%	0.0000%
202.85	Other lymphomas, lymph nodes of inguinal region and low	0.0028%	0.0000%	0.0000%	0.0032%
172.9	Malignant melanoma of skin, site unspecified	0.0014%	0.0048%	0.0062%	0.0000%
146.1	Malignant neoplasm of tonsillar fossa	0.0014%	0.0048%	0.0041%	0.0032%
112.5	Candidiasis of disseminated	0.0014%	0.0000%	0.0021%	0.0096%
972.1	Poisoning by cardiotonic glycosides and drugs of similar ac	0.0009%	0.0048%	0.0021%	0.0064%
997.3	Respiratory complications	0.0018%	0.0048%	0.0000%	0.0032%
996.65	Infection and inflammatory reaction due to other genitourin	0.0023%	0.0000%	0.0021%	0.0000%
942.30	Burn of trunk, unspecified site, full-thickness skin loss (thi	0.0018%	0.0000%	0.0041%	0.0000%
938	Foreign body in digestive system, unspecified	0.0028%	0.0000%	0.0000%	0.0000%
945.23	Burn of ankle, blisters, epidermal loss (second degree)	0.0023%	0.0000%	0.0021%	0.0000%
924.01	Contusion of hip	0.0005%	0.0048%	0.0062%	0.0032%
945.35	Burn of knee, full-thickness skin loss (third degree NOS)	0.0028%	0.0000%	0.0000%	0.0000%
881.10	Open wound of forearm, complicated	0.0018%	0.0000%	0.0041%	0.0000%
947.9	Burn of unspecified internal organs	0.0018%	0.0048%	0.0021%	0.0000%
933.1	Foreign body in larynx	0.0023%	0.0000%	0.0000%	0.0032%
922.32	Contusion of buttock	0.0023%	0.0000%	0.0000%	0.0032%
941.00	Burn of face and head, unspecified site, unspecified degree	0.0023%	0.0000%	0.0021%	0.0000%
879.4	Open wound of abdominal wall, lateral, without mention of	0.0018%	0.0048%	0.0021%	0.0000%
958.6	Volkmann's ischemic contracture	0.0023%	0.0048%	0.0000%	0.0000%
863.82	Injury to body of pancreas, without mention of open wound	0.0018%	0.0048%	0.0021%	0.0000%
834.12	Open dislocation of interphalangeal (joint) of hand	0.0023%	0.0000%	0.0000%	0.0032%
812.02	Fracture of anatomical neck of humerus, closed	0.0023%	0.0000%	0.0021%	0.0000%
838.05	Closed dislocation, metatarsophalangeal(joint)	0.0028%	0.0000%	0.0000%	0.0000%

ICD-9-CM	ICD-9-CM	PureO	ContiB	NewB	PastB
873.64	Open wound of tongue and floor of mouth, without mentio	0.0028%	0.0000%	0.0000%	0.0000%
838.16	Open dislocation, interphalangeal(joint), foot	0.0014%	0.0000%	0.0041%	0.0032%
864.02	Minor laceration to liver, without mention of open wound i	0.0028%	0.0000%	0.0000%	0.0000%
816.13	Fracture in multiple sites of phalanges, open	0.0023%	0.0000%	0.0021%	0.0000%
868.10	Injury to unspecified intra-abdominal organs with open wo	0.0014%	0.0048%	0.0000%	0.0064%
802.9	Fracture of other facial bones, open	0.0023%	0.0000%	0.0000%	0.0032%
815.14	Fracture of neck of metacarpal bone(s), open	0.0023%	0.0000%	0.0021%	0.0000%
807.4	Flail chest	0.0018%	0.0000%	0.0041%	0.0000%
863.39	Injury to other small intestine,with open wound into cavity	0.0014%	0.0000%	0.0062%	0.0000%
841.9	Sprains and strains of unspecified site of elbow and forearn	0.0028%	0.0000%	0.0000%	0.0000%
863.83	Injury to tail of pancreas, without mention of open wound i	0.0014%	0.0000%	0.0062%	0.0000%
842.00	Sprains and strains of unspecified site of wrist	0.0018%	0.0000%	0.0041%	0.0000%
864.15	Unspecified injury to liver, with open wound into cavity	0.0018%	0.0000%	0.0041%	0.0000%
842.01	Sprains and strains of carpal (joint)	0.0009%	0.0048%	0.0021%	0.0064%
832.02	Closed posterior dislocation of elbow	0.0000%	0.0048%	0.0041%	0.0096%
820.32	Fracture of subtrochanteric section of femur, open	0.0009%	0.0048%	0.0041%	0.0032%
815.09	Fracture in multiple sites of metacarpus, closed	0.0028%	0.0000%	0.0000%	0.0000%
813.14	Other and unspecified fractures of proximal end of ulna(alc	0.0023%	0.0000%	0.0000%	0.0032%
872.00	Open wound to unspecified site of external ear, without me	0.0023%	0.0000%	0.0021%	0.0000%
806.24	T1-T6 level fracture with other specified spinal cord injury	0.0014%	0.0000%	0.0062%	0.0000%
873.54	Open wound of jaw, complicated	0.0028%	0.0000%	0.0000%	0.0000%
811.09	Fracture in other part of scapula, closed	0.0014%	0.0048%	0.0021%	0.0032%
863.1	Injury to stomach,with open wound into cavity	0.0009%	0.0048%	0.0021%	0.0064%
862.22	Injury to esophagus,without mention of open wound into ca	0.0009%	0.0000%	0.0083%	0.0000%
750.7	Other specified anomalies of stomach	0.0023%	0.0000%	0.0021%	0.0000%
756.59	Other osteodystrophies	0.0023%	0.0000%	0.0021%	0.0000%
749.10	Cleft lip, unspecified	0.0023%	0.0000%	0.0021%	0.0000%
730.95	Unspecified infection of bone, pelvic region and thigh	0.0028%	0.0000%	0.0000%	0.0000%
800.23	Fracture of vault of skull, closed with subarachnoid, subdu	0.0018%	0.0000%	0.0041%	0.0000%
730.05	Acute osteomyelitis, pelvic region and thigh	0.0018%	0.0000%	0.0000%	0.0064%
791.9	Other nonspecific findings on examination of urine	0.0009%	0.0097%	0.0021%	0.0032%
783.0	Anorexia	0.0018%	0.0048%	0.0000%	0.0032%
781.99	Other symptoms involving nervous and musculoskeketal sy	0.0009%	0.0000%	0.0083%	0.0000%
738.9	Acquired deformity of unspecified site	0.0023%	0.0048%	0.0000%	0.0000%
744.29	Other specified anomalies of ear	0.0028%	0.0000%	0.0000%	0.0000%
729.81	Swelling of limb	0.0009%	0.0000%	0.0041%	0.0064%
747.41	Total anomalous pulmonary venous connection	0.0028%	0.0000%	0.0000%	0.0000%
753.0	Renal agenesis and dysgenesis	0.0028%	0.0000%	0.0000%	0.0000%
755.52	Congenital elevation of scapula	0.0028%	0.0000%	0.0000%	0.0000%

ICD-9-CM	ICD-9-CM	PureO	ContiB	NewB	PastB
741.93	Spina bifida without mention of hydrocephalus, lumbar reg	0.0028%	0.0000%	0.0000%	0.0000%
746.86	Congenital heart block	0.0028%	0.0000%	0.0000%	0.0000%
759.89	Other specified anomalies	0.0028%	0.0000%	0.0000%	0.0000%
735.1	Hallux varus (acquired)	0.0028%	0.0000%	0.0000%	0.0000%
789.05	Abdominal pain, periumbilic	0.0018%	0.0048%	0.0000%	0.0032%
755.65	Macrodactylia of toes	0.0023%	0.0000%	0.0021%	0.0000%
665.54	Other injury to pelvic organs, postpartum condition or com	0.0028%	0.0000%	0.0000%	0.0000%
695.2	Erythema nodosum	0.0014%	0.0000%	0.0062%	0.0000%
648.41	Mental disorders conditions in the mother classifiable elsev	0.0009%	0.0048%	0.0021%	0.0064%
718.42	Contracture of joint, upper arm	0.0018%	0.0048%	0.0000%	0.0032%
718.27	Pathological dislocation, ankle and foot	0.0023%	0.0000%	0.0000%	0.0032%
718.84	Other joint derangement, not elsewhere classified, hand	0.0018%	0.0000%	0.0021%	0.0032%
726.12	Bicipital tenosynovitis	0.0009%	0.0000%	0.0041%	0.0064%
666.34	Postpartum coagulation defects, postpartum condition or co	0.0023%	0.0000%	0.0021%	0.0000%
726.71	Achilles bursitis or tendinitis	0.0009%	0.0145%	0.0021%	0.0000%
709.3	Degenerative skin disorders	0.0005%	0.0000%	0.0062%	0.0064%
715.16	Osteoarthrosis, localized, primary, lower leg	0.0009%	0.0000%	0.0021%	0.0096%
652.11	Breech or other malpresentation successfully converted to c	0.0023%	0.0000%	0.0000%	0.0032%
571.8	Other chronic nonalcoholic liver disease	0.0018%	0.0048%	0.0021%	0.0000%
635.02	Legally induced abortion, complicated by genital tract and	0.0018%	0.0000%	0.0041%	0.0000%
534.90	Gastrojejunal ulcer, unspecified as acute or chronic, withou	0.0018%	0.0000%	0.0000%	0.0064%
595.0	Acute cystitis	0.0009%	0.0048%	0.0041%	0.0032%
639.0	Complications following abortion and ectopic and molar pr	0.0018%	0.0000%	0.0041%	0.0000%
601.0	Acute prostatitis	0.0005%	0.0097%	0.0041%	0.0032%
633.0	Abdominal pregnancy	0.0023%	0.0000%	0.0021%	0.0000%
607.2	Other inflammatory disorders of penis	0.0023%	0.0000%	0.0000%	0.0032%
601.9	Prostatitis, unspecified	0.0005%	0.0048%	0.0062%	0.0032%
610.4	Mammary duct ectasia	0.0018%	0.0000%	0.0000%	0.0064%
535.60	Duodenitis, without mention of hemorrhage	0.0018%	0.0000%	0.0041%	0.0000%
530.6	Diverticulum of esophagus, acquired	0.0018%	0.0000%	0.0021%	0.0032%
532.10	Duodenal ulcer, acute with perforation, without mention of	0.0023%	0.0000%	0.0021%	0.0000%
624.8	Other specified noninflammatory disorders of vulva and pe	0.0028%	0.0000%	0.0000%	0.0000%
569.9	Unspecified disorder of intestine	0.0023%	0.0000%	0.0000%	0.0032%
575.9	Unspecified disorders of gallbladder	0.0023%	0.0048%	0.0000%	0.0000%
604.99	Other orchitis and epididymitis	0.0023%	0.0048%	0.0000%	0.0000%
404.92	Unspecified hypertensive heart and renal disease with renal	0.0005%	0.0097%	0.0041%	0.0032%
368.2	Diplopia	0.0014%	0.0097%	0.0021%	0.0000%
410.31	Acute myocardial infarction of inferoposterior wall, initial	0.0018%	0.0000%	0.0041%	0.0000%
365.63	Glaucoma associated with vascular disorders	0.0018%	0.0000%	0.0021%	0.0032%

ICD-9-CM	ICD-9-CM	PureO	ContiB	NewB	PastB
478.20	Unspecified disease of pharynx	0.0018%	0.0000%	0.0021%	0.0032%
380.14	Malignant otitis externa	0.0023%	0.0000%	0.0021%	0.0000%
383.00	Acute mastoiditis without complications	0.0018%	0.0048%	0.0021%	0.0000%
522.6	Chronic apical periodontitis	0.0018%	0.0048%	0.0021%	0.0000%
454.2	Varicose veins of lower extremities with ulcer and inflamm	0.0018%	0.0000%	0.0021%	0.0032%
442.84	Aneurysm of other visceral artery	0.0018%	0.0097%	0.0000%	0.0000%
437.6	Nonpyogenic thrombosis of intracranial venous sinus	0.0009%	0.0000%	0.0083%	0.0000%
370.05	Mycotic corneal ulcer	0.0014%	0.0000%	0.0041%	0.0032%
440.22	Atherosclerosis of the extremities with rest pain	0.0014%	0.0000%	0.0041%	0.0032%
422.91	Idiopathic myocarditis	0.0014%	0.0048%	0.0041%	0.0000%
380.01	Acute perichondritis of pinna	0.0018%	0.0000%	0.0021%	0.0032%
380.39	Other noninfectious disorders of pinna	0.0018%	0.0048%	0.0000%	0.0032%
388.8	Other disorders of ear	0.0018%	0.0000%	0.0021%	0.0032%
458.9	Hypotension, unspecified	0.0014%	0.0048%	0.0021%	0.0032%
379.09	Other scleritis and episcleritis	0.0014%	0.0097%	0.0000%	0.0032%
379.34	Posterior dislocation of lens	0.0018%	0.0000%	0.0041%	0.0000%
435.8	Other specified transient cerebral ischemias	0.0005%	0.0048%	0.0021%	0.0096%
348.1	Anoxic brain damage	0.0009%	0.0048%	0.0021%	0.0064%
358.9	Myoneural disorders, unspecified	0.0028%	0.0000%	0.0000%	0.0000%
275.49	Other disorders of calcium metabolism	0.0009%	0.0097%	0.0000%	0.0064%
224.1	Benign neoplasm of orbit	0.0023%	0.0048%	0.0000%	0.0000%
361.02	Recent retinal detachment, partial, with multiple defects	0.0028%	0.0000%	0.0000%	0.0000%
289.9	Unspecified diseases of blood and blood-forming organs	0.0018%	0.0048%	0.0000%	0.0032%
250.11	Diabetes with ketoacidosis, Type I [insulin dependent type]	0.0023%	0.0000%	0.0021%	0.0000%
296.20	Major depressive disorder, single episode, unspecified	0.0000%	0.0194%	0.0000%	0.0064%
276.5	Volume depletion	0.0005%	0.0097%	0.0021%	0.0064%
235.3	Neoplasm of uncertain behavior of liver and biliary passage	0.0018%	0.0048%	0.0000%	0.0032%
279.4	Autoimmune disease, not elsewhere classified	0.0005%	0.0048%	0.0083%	0.0000%
286.0	Congenital factor VIII disorder	0.0023%	0.0048%	0.0000%	0.0000%
346.20	Variants of migraine，without mention of intractable migr	0.0014%	0.0048%	0.0000%	0.0064%
235.7	Neoplasm of uncertain behavior of trachea, bronchus and lu	0.0018%	0.0000%	0.0021%	0.0032%
273.2	Other paraproteinemias	0.0009%	0.0048%	0.0021%	0.0064%
287.4	Secondary thrombocytopenia	0.0005%	0.0097%	0.0000%	0.0096%
354.8	Other mononeuritis of upper limb	0.0018%	0.0048%	0.0021%	0.0000%
335.20	Amyotrophic lateral sclerosis	0.0009%	0.0145%	0.0000%	0.0032%
237.4	Neoplasm of uncertain behavior of other and unspecified en	0.0023%	0.0000%	0.0021%	0.0000%
255.0	Cushing's syndrome	0.0000%	0.0097%	0.0041%	0.0064%
222.0	Benign neoplasm of testis	0.0023%	0.0000%	0.0021%	0.0000%
281.9	Unspecified deficiency anemia	0.0009%	0.0048%	0.0041%	0.0032%

ICD-9-CM	ICD-9-CM	PureO	ContiB	NewB	PastB
215.8	Benign neoplasm of connective and other soft tissue of oth	0.0023%	0.0000%	0.0021%	0.0000%
336.8	Other myelopathy	0.0014%	0.0048%	0.0000%	0.0064%
232.5	Carcinoma in situ of skin of trunk, except scrotum	0.0005%	0.0048%	0.0041%	0.0064%
344.1	Paraplegia	0.0009%	0.0048%	0.0062%	0.0000%
053.21	Herpes zoster keratoconjunctivitis	0.0028%	0.0000%	0.0000%	0.0000%
013.02	Tuberculous meningitis, bacteriological or histological exa	0.0005%	0.0000%	0.0021%	0.0127%
201.90	Hodgkin's disease, unspecified, unspecified site, extranodal	0.0018%	0.0000%	0.0000%	0.0064%
170.0	Malignant neoplasm of bones of skull and face, except ma	0.0009%	0.0048%	0.0021%	0.0064%
187.2	Malignant neoplasm of glans penis	0.0014%	0.0097%	0.0021%	0.0000%
197.1	Secondary malignant neoplasm of mediastinum	0.0014%	0.0048%	0.0041%	0.0000%
017.96	Tuberculosis of other specified organ, tubercle bacilli not f	0.0009%	0.0145%	0.0021%	0.0000%
198.0	Secondary malignant neoplasm of kidney	0.0014%	0.0000%	0.0041%	0.0032%
201.91	Hodgkin's disease, unspecified, lymph nodes of head, face	0.0018%	0.0000%	0.0041%	0.0000%
017.26	Tuberculosis of peripheral lymph nodes, tubercle bacilli no	0.0028%	0.0000%	0.0000%	0.0000%
039.8	Actinomycotic infections of other specified sites	0.0028%	0.0000%	0.0000%	0.0000%
200.28	Burkitt's tumor or lymphoma, lymph nodes of multiple site	0.0014%	0.0048%	0.0041%	0.0000%
149.0	Malignant neoplasm of pharynx, unspecified	0.0005%	0.0097%	0.0041%	0.0032%
140.9	Malignant neoplasm of lip, unspecified, vermilion border	0.0009%	0.0048%	0.0062%	0.0000%
008.61	Enteritis due to Rotavirus	0.0028%	0.0000%	0.0000%	0.0000%
009.2	Infectious diarrhea	0.0009%	0.0000%	0.0021%	0.0096%
012.04	Tuberculous pleurisy , tubercle bacilli not found (in sputum	0.0009%	0.0048%	0.0041%	0.0032%
053.71	Otitis externa due to herpes zoster	0.0018%	0.0000%	0.0041%	0.0000%
140.4	Malignant neoplasm of lower lip, inner aspect	0.0009%	0.0097%	0.0041%	0.0000%
989.6	Toxic effect of soaps and detergents	0.0018%	0.0048%	0.0000%	0.0000%
928.8	Crushing injury of multiple sites of lower limb	0.0018%	0.0000%	0.0021%	0.0000%
893.0	Open wound of toe(s), without mention of complication	0.0023%	0.0000%	0.0000%	0.0000%
904.51	Injury to anterior tibial artery	0.0014%	0.0000%	0.0041%	0.0000%
900.89	Other injury to blood vessels of head and neck	0.0014%	0.0000%	0.0041%	0.0000%
941.34	Burn chin, full-thickness skin loss (third degree NOS)	0.0023%	0.0000%	0.0000%	0.0000%
890.2	Open wound of hip and thigh, with tendon involvement	0.0023%	0.0000%	0.0000%	0.0000%
904.53	Injury to posterior tibial artery	0.0023%	0.0000%	0.0000%	0.0000%
894.1	Multiple and unspecified open wound of lower limb, comp	0.0014%	0.0000%	0.0021%	0.0032%
945.42	Burn of foot, deep necrosis of underlying tissues (deep thir	0.0009%	0.0000%	0.0021%	0.0064%
932	Foreign body in nose	0.0023%	0.0000%	0.0000%	0.0000%
945.44	Burn of lower leg, deep necrosis of underlying tissues (dee	0.0018%	0.0000%	0.0021%	0.0000%
942.35	Burn of genitalia, full-thickness skin loss (third degree NO	0.0018%	0.0000%	0.0021%	0.0000%
947.0	Burn of mouth and pharynx	0.0014%	0.0000%	0.0041%	0.0000%
944.25	Burn of palm, blisters, epidermal loss (second degree)	0.0018%	0.0000%	0.0000%	0.0032%
942.25	Burn of trunk, genitalia, blisters, epidermal loss (second de	0.0023%	0.0000%	0.0000%	0.0000%

ICD-9-CM	ICD-9-CM	PureO	ContiB	NewB	PastB
877.1	Open wound of buttock, complicated	0.0014%	0.0000%	0.0041%	0.0000%
952.15	T7-T12 level with unspecified spinal cord injury	0.0009%	0.0000%	0.0062%	0.0000%
996.71	Other complications due to heart valve prosthesis	0.0005%	0.0048%	0.0062%	0.0000%
877.0	Open wound of buttock, without mention of complication	0.0014%	0.0048%	0.0000%	0.0032%
896.0	Traumatic amputation of foot (complete) (partial) , unilater	0.0014%	0.0000%	0.0041%	0.0000%
917.1	Superficial injury of foot and toe(s), abrasion or friction bu	0.0009%	0.0000%	0.0041%	0.0032%
903.8	Injury to other specified blood vessels of upper extremity	0.0023%	0.0000%	0.0000%	0.0000%
955.7	Injury to other specified nerve(s) of shoulder girdle and up	0.0023%	0.0000%	0.0000%	0.0000%
897.4	Traumatic amputation of leg(s) (complete) (partial), unilate	0.0014%	0.0000%	0.0041%	0.0000%
919.0	Superficial injury of other, multiple and unspecified sites, a	0.0018%	0.0000%	0.0021%	0.0000%
V71.89	Observation for other specified suspected conditions	0.0023%	0.0000%	0.0000%	0.0000%
901.1	Injury to innominate and subclavian arteries	0.0014%	0.0000%	0.0041%	0.0000%
987.9	Toxic effect of unspecified gas, fume, or vapor	0.0023%	0.0000%	0.0000%	0.0000%
812.52	Fracture of lateral condyle of humerus, open	0.0018%	0.0000%	0.0021%	0.0000%
839.08	Dislocations of multiple cervical vertebrae, closed	0.0009%	0.0097%	0.0021%	0.0000%
835.01	Closed posterior dislocation of hip	0.0023%	0.0000%	0.0000%	0.0000%
845.01	Sprains and strains of deltoid (ligament),ankle	0.0023%	0.0000%	0.0000%	0.0000%
807.00	Unspecified fracture of rib(s), closed	0.0018%	0.0048%	0.0000%	0.0000%
845.03	Sprains and strains of tibiofibular (ligament),distal	0.0018%	0.0000%	0.0000%	0.0032%
834.02	Closed dislocation of interphalangeal (joint) of hand	0.0023%	0.0000%	0.0000%	0.0000%
848.8	Sprains and strains of other specified sites	0.0018%	0.0048%	0.0000%	0.0000%
863.30	Injury to small intestine,unspecified site,with open wound i	0.0014%	0.0000%	0.0021%	0.0032%
850.9	Concussion,unspecified	0.0014%	0.0000%	0.0041%	0.0000%
840.3	Sprains and strains of infraspinatus (muscle) (tendon)	0.0014%	0.0000%	0.0021%	0.0032%
806.06	C5- C7 level fracture with complete lesion of cord, closed	0.0009%	0.0000%	0.0062%	0.0000%
832.01	Closed anterior dislocation of elbow	0.0023%	0.0000%	0.0000%	0.0000%
852.42	Extradural hemorrhage following injury without mention o	0.0009%	0.0000%	0.0062%	0.0000%
802.7	Fracture of orbital floor (blow-out), open	0.0018%	0.0000%	0.0021%	0.0000%
853.03	Other and unspecified intracranial hemorrhage following in	0.0009%	0.0048%	0.0021%	0.0032%
838.06	Closed dislocation, interphalangeal(joint), foot	0.0018%	0.0000%	0.0000%	0.0032%
802.31	Fracture of mandible, open, condylar process	0.0018%	0.0000%	0.0021%	0.0000%
815.19	Fracture in multiple sites of metacarpus, open	0.0018%	0.0000%	0.0000%	0.0032%
854.01	Other and unspecified intracranial injury without mention c	0.0009%	0.0048%	0.0041%	0.0000%
873.50	Open wound to unspecified site of face, complicated	0.0023%	0.0000%	0.0000%	0.0000%
750.5	Congenital hypertrophic pyloric stenosis	0.0023%	0.0000%	0.0000%	0.0000%
745.61	Ostium primum defect	0.0018%	0.0048%	0.0000%	0.0000%
800.71	Fracture of vault of skull, open with subarachnoid, subdura	0.0018%	0.0000%	0.0021%	0.0000%
728.6	Contracture of palmar fascia	0.0014%	0.0000%	0.0021%	0.0032%
747.3	Anomalies of pulmonary artery	0.0018%	0.0000%	0.0000%	0.0032%

ICD-9-CM	ICD-9-CM	PureO	ContiB	NewB	PastB
753.22	Congenital obstruction of ureterovesical junction	0.0023%	0.0000%	0.0000%	0.0000%
732.2	Nontraumatic slipped upper femoral epiphysis	0.0023%	0.0000%	0.0000%	0.0000%
789.04	Abdominal pain, left lower quadrant	0.0014%	0.0000%	0.0041%	0.0000%
780.9	Other general symptoms	0.0009%	0.0000%	0.0041%	0.0032%
730.13	Chronic osteomyelitis, forearm	0.0009%	0.0048%	0.0000%	0.0064%
801.34	Fracture of base of skull, closed with other and unspecified	0.0009%	0.0097%	0.0000%	0.0032%
742.59	Other specified anomalies of spinal cord	0.0009%	0.0000%	0.0041%	0.0032%
754.32	Congenital subluxation of hip, unilateral	0.0023%	0.0000%	0.0000%	0.0000%
755.30	Unspecified reduction deformity of lower limb	0.0023%	0.0000%	0.0000%	0.0000%
745.3	Common ventricle	0.0023%	0.0000%	0.0000%	0.0000%
738.3	Acquired deformity of chest and rib	0.0014%	0.0048%	0.0000%	0.0032%
747.42	Partial anomalous pulmonary venous connection	0.0018%	0.0000%	0.0021%	0.0000%
755.56	Accessory carpal bones	0.0023%	0.0000%	0.0000%	0.0000%
801.32	Fracture of base of skull, closed with other and unspecified	0.0018%	0.0000%	0.0021%	0.0000%
800.00	Fracture of vault of skull, closed without mention of intracr	0.0009%	0.0048%	0.0021%	0.0032%
787.3	Flatulence, eructation, and gas pain	0.0005%	0.0097%	0.0021%	0.0032%
743.30	Congenital cataract, unspecified	0.0023%	0.0000%	0.0000%	0.0000%
736.32	Coxa vara (acquired)	0.0023%	0.0000%	0.0000%	0.0000%
785.50	Shock, unspecified	0.0014%	0.0048%	0.0000%	0.0032%
756.4	Chondrodystrophy	0.0023%	0.0000%	0.0000%	0.0000%
800.11	Fracture of vault of skull, closed with cerebral laceration ar	0.0005%	0.0048%	0.0062%	0.0000%
728.9	Unspecified disorder of muscle, ligament, and fascia	0.0018%	0.0000%	0.0021%	0.0000%
686.01	Pyoderma gangrenosum	0.0009%	0.0048%	0.0041%	0.0000%
680.3	Carbuncle and furuncle, upper arm and forearm	0.0009%	0.0097%	0.0000%	0.0032%
716.85	Other specified arthropathy, pelvic region and thigh	0.0018%	0.0000%	0.0021%	0.0000%
708.9	Urticaria, unspecified	0.0014%	0.0000%	0.0000%	0.0064%
669.14	Shock during of following labor and delivery, postpartum c	0.0018%	0.0000%	0.0021%	0.0000%
651.03	Twin pregnancy, antepartum condition or complication	0.0023%	0.0000%	0.0000%	0.0000%
646.71	Liver disorders in pregnancy, delivered, with or without me	0.0023%	0.0000%	0.0000%	0.0000%
655.13	Chromosomal abnormality in fetus, affecting management c	0.0023%	0.0000%	0.0000%	0.0000%
716.95	Arthropathy, unspecified, pelvic region and thigh	0.0014%	0.0000%	0.0021%	0.0032%
667.02	Retained placenta without hemorrhage, delivered, with mer	0.0023%	0.0000%	0.0000%	0.0000%
727.3	Other bursitis	0.0014%	0.0000%	0.0021%	0.0032%
656.43	Intrauterine death affecting management of mother, antepar	0.0023%	0.0000%	0.0000%	0.0000%
719.81	Other specified disorders of joint, shoulder region	0.0014%	0.0000%	0.0041%	0.0000%
695.89	Other specified erythematous conditions	0.0014%	0.0000%	0.0000%	0.0064%
704.8	Other specified diseases of hair and hair follicles	0.0018%	0.0000%	0.0021%	0.0000%
711.95	Unspecified infective arthritis, pelvic region and thigh	0.0023%	0.0000%	0.0000%	0.0000%
717.49	Other derangement of lateral meniscus	0.0018%	0.0000%	0.0000%	0.0032%

ICD-9-CM	ICD-9-CM	PureO	ContiB	NewB	PastB
656.71	Other placental conditions affecting management of mother	0.0023%	0.0000%	0.0000%	0.0000%
726.30	Enthesopathy of elbow, unspecified	0.0023%	0.0000%	0.0000%	0.0000%
709.4	Foreign body granuloma of skin and subcutaneous tissue	0.0018%	0.0000%	0.0021%	0.0000%
659.21	Maternal pyrexia during labor, unspecified, delivered, with	0.0023%	0.0000%	0.0000%	0.0000%
718.43	Contracture of joint, forearm	0.0023%	0.0000%	0.0000%	0.0000%
716.92	Arthropathy, unspecified, upper arm	0.0009%	0.0000%	0.0021%	0.0064%
715.97	Osteoarthrosis, unspecified whether generalized or localize	0.0005%	0.0097%	0.0041%	0.0000%
721.2	Thoracic spondylosis without myelopathy	0.0014%	0.0000%	0.0041%	0.0000%
665.71	Pelvic hematoma, delivered, with or without mention of an	0.0023%	0.0000%	0.0000%	0.0000%
715.95	Osteoarthrosis, unspecified whether generalized or localize	0.0014%	0.0048%	0.0000%	0.0032%
655.83	Other known or suspected fetal abnormality, not elsewhere	0.0023%	0.0000%	0.0000%	0.0000%
719.83	Other specified disorders of joint, forearm	0.0018%	0.0048%	0.0000%	0.0000%
639.1	Complications following abortion and ectopic and molar pr	0.0023%	0.0000%	0.0000%	0.0000%
551.00	Femoral hernia with gangrene, unilateral or unspecified (nc	0.0018%	0.0000%	0.0000%	0.0032%
625.9	Unspecified symptom associated with femal genital organs	0.0018%	0.0000%	0.0021%	0.0000%
524.02	Mandibular hyperplasia	0.0023%	0.0000%	0.0000%	0.0000%
551.9	Hernia of unspecified site, with gangrene	0.0018%	0.0000%	0.0000%	0.0032%
572.3	Portal hypertension	0.0018%	0.0048%	0.0000%	0.0000%
596.9	Unspecified disorder of bladder	0.0009%	0.0000%	0.0021%	0.0064%
535.41	Other specified gastritis, with hemorrhage	0.0018%	0.0000%	0.0000%	0.0032%
626.6	Metrorrhagia	0.0014%	0.0000%	0.0041%	0.0000%
551.8	Hernia of other specified sites, with gangrene	0.0014%	0.0000%	0.0021%	0.0032%
571.49	Other chronic hepatitis	0.0014%	0.0000%	0.0041%	0.0000%
532.71	Duodenal ulcer, chronic without mention of hemorrhage or	0.0009%	0.0048%	0.0021%	0.0032%
607.0	Leukoplakia of penis	0.0023%	0.0000%	0.0000%	0.0000%
536.8	Dyspepsia and other specified disorders of function of ston	0.0009%	0.0048%	0.0021%	0.0032%
524.01	Maxillary hyperplasia	0.0018%	0.0000%	0.0021%	0.0000%
611.3	Fat necrosis of breast	0.0023%	0.0000%	0.0000%	0.0000%
426.10	Atrioventricular block, unspecified	0.0009%	0.0048%	0.0000%	0.0064%
384.20	Perforation of tympanic membrane, unspecified	0.0009%	0.0000%	0.0021%	0.0064%
446.6	Thrombotic microangiopathy	0.0009%	0.0000%	0.0041%	0.0032%
396.1	Mitral valve stenosis and aortic valve insufficiency	0.0009%	0.0097%	0.0021%	0.0000%
374.46	Blepharophimosis	0.0023%	0.0000%	0.0000%	0.0000%
437.0	Cerebral atherosclerosis	0.0000%	0.0097%	0.0041%	0.0032%
377.39	Other optic neuritis	0.0014%	0.0000%	0.0041%	0.0000%
379.00	Scleritis, unspecified	0.0009%	0.0000%	0.0062%	0.0000%
371.89	Other corneal disorders	0.0023%	0.0000%	0.0000%	0.0000%
385.10	Adhesive middle ear disease, unspecified as to involvemen	0.0014%	0.0048%	0.0000%	0.0032%
433.30	Occlusion and stenosis of multiple and bilateral precerebral	0.0014%	0.0048%	0.0021%	0.0000%

ICD-9-CM	ICD-9-CM	PureO	ContiB	NewB	PastB
378.11	Monocular exotropia	0.0018%	0.0048%	0.0000%	0.0000%
403.90	Unspecified hypertensive renal disease without mention of	0.0014%	0.0048%	0.0021%	0.0000%
474.9	Unspecified chronic disease of tonsils and adenoids	0.0018%	0.0000%	0.0021%	0.0000%
444.89	Arterial embolism and thrombosis of other specified artery	0.0018%	0.0048%	0.0000%	0.0000%
370.9	Unspecified keratitis	0.0023%	0.0000%	0.0000%	0.0000%
442.1	Aneurysm of renal artery	0.0018%	0.0000%	0.0000%	0.0032%
444.0	Arterial embolism and thrombosis of abdominal aorta	0.0023%	0.0000%	0.0000%	0.0000%
459.2	Compression of vein	0.0018%	0.0048%	0.0000%	0.0000%
381.20	Chronic mucoid otitis media, simple or unspecified	0.0018%	0.0000%	0.0021%	0.0000%
389.10	Sensorineural hearing loss, unspecified	0.0023%	0.0000%	0.0000%	0.0000%
433.11	Occlusion and stenosis of carotid artery with cerebral infar	0.0009%	0.0000%	0.0021%	0.0064%
478.21	Cellulitis of pharynx or nasopharynx	0.0014%	0.0000%	0.0021%	0.0032%
436	Acute, but ill-defined, cerebrovascular disease	0.0005%	0.0048%	0.0041%	0.0032%
355.6	Lesion of plantar nerve	0.0005%	0.0000%	0.0021%	0.0096%
223.1	Benign neoplasm of renal pelvis	0.0018%	0.0000%	0.0000%	0.0032%
245.1	Subacute thyroiditis	0.0018%	0.0048%	0.0000%	0.0000%
245.9	Thyroiditis, unspecified	0.0023%	0.0000%	0.0000%	0.0000%
296.40	Bipolar affective disorder, manic, unspecified	0.0000%	0.0097%	0.0000%	0.0096%
210.0	Benign neoplasm of lip	0.0014%	0.0048%	0.0021%	0.0000%
361.03	Recent retinal detachment, partial, with giant tear	0.0018%	0.0000%	0.0000%	0.0032%
283.9	Acquired hemolytic anemia, unspecified	0.0009%	0.0048%	0.0041%	0.0000%
296.30	Major depressive disorder, recurrent episode, unspecified	0.0000%	0.0097%	0.0021%	0.0064%
246.8	Other specified disorders of thyroid	0.0023%	0.0000%	0.0000%	0.0000%
255.2	Adrenogenital disorders	0.0023%	0.0000%	0.0000%	0.0000%
246.9	Unspecified disorder of thyroid	0.0014%	0.0000%	0.0000%	0.0064%
289.2	Nonspecific mesenteric lymphadenitis	0.0018%	0.0000%	0.0000%	0.0032%
250.03	Diabetes mellitus without mention of complication, Type I	0.0009%	0.0048%	0.0000%	0.0064%
230.8	Carcinoma in situ of liver and biliary system	0.0018%	0.0000%	0.0021%	0.0000%
213.3	Benign neoplasm of ribs, sternum and clavicle	0.0023%	0.0000%	0.0000%	0.0000%
206.00	Acute monocytic leukemia, without mention of remission	0.0018%	0.0000%	0.0021%	0.0000%
210.7	Benign neoplasm of nasopharynx	0.0023%	0.0000%	0.0000%	0.0000%
333.82	Orofacial dyskinesia	0.0000%	0.0000%	0.0103%	0.0000%
214.0	Lipoma of skin and subcutaneous tissue of face	0.0023%	0.0000%	0.0000%	0.0000%
219.0	Benign neoplasm of cervix uteri	0.0023%	0.0000%	0.0000%	0.0000%
221.2	Benign neoplasm of vulva	0.0023%	0.0000%	0.0000%	0.0000%
333.81	Blepharospasm	0.0018%	0.0000%	0.0000%	0.0032%
145.4	Malignant neoplasm of uvula	0.0000%	0.0000%	0.0083%	0.0032%
008.43	Intestinal infections due to Campylobacter	0.0018%	0.0000%	0.0021%	0.0000%
190.3	Malignant neoplasm of conjunctiva	0.0009%	0.0000%	0.0021%	0.0064%

ICD-9-CM	ICD-9-CM	PureO	ContiB	NewB	PastB
008.8	Intestinal infections due to other organism, not elsewhere c	0.0023%	0.0000%	0.0000%	0.0000%
054.9	Herpes simplex without mention of complication	0.0023%	0.0000%	0.0000%	0.0000%
065.4	Mosquito-borne hemorrhagic fever	0.0014%	0.0000%	0.0000%	0.0064%
200.08	Reticulosarcoma, lymph nodes of multiple sites	0.0018%	0.0000%	0.0021%	0.0000%
031.1	Cutaneous diseases due to other mycobacteria	0.0014%	0.0000%	0.0041%	0.0000%
195.0	Malignant neoplasm of other and ill-defined sites of head, f	0.0000%	0.0048%	0.0062%	0.0032%
202.05	Nodular lymphoma, lymph nodes of inguinal region and lo	0.0005%	0.0097%	0.0021%	0.0032%
148.0	Malignant neoplasm of postcricoid region of hypopharynx	0.0014%	0.0048%	0.0000%	0.0032%
053.0	Herpes zoster with meningitis	0.0014%	0.0000%	0.0021%	0.0032%
170.3	Malignant neoplasm of ribs, sternum and clavicle	0.0023%	0.0000%	0.0000%	0.0000%
172.8	Malignant melanoma of other specified sites of skin	0.0009%	0.0000%	0.0041%	0.0032%
039.1	Pulmonary diseases due to actinomycotic infections	0.0018%	0.0000%	0.0021%	0.0000%
V70.8	Other specified general medical examination	0.0018%	0.0000%	0.0000%	0.0000%
952.09	C5-C7 level with other specified spinal cord injury	0.0005%	0.0000%	0.0062%	0.0000%
875.1	Open wound of chest(wall), complicated	0.0014%	0.0000%	0.0021%	0.0000%
945.04	Burn of lower leg, unspecified degree	0.0014%	0.0000%	0.0021%	0.0000%
V55.0	Attention to tracheostomy	0.0009%	0.0048%	0.0000%	0.0032%
946.2	Burns of multiple specified sites, blisters, epidermal loss (s	0.0018%	0.0000%	0.0000%	0.0000%
V72.83	Other specified pre-operative examination	0.0009%	0.0000%	0.0041%	0.0000%
953.0	Injury to cervical root	0.0018%	0.0000%	0.0000%	0.0000%
923.00	Contusion of shoulder region	0.0014%	0.0048%	0.0000%	0.0000%
995.1	Angioneurotic edema	0.0009%	0.0048%	0.0021%	0.0000%
V53.09	Fitting and adjustment of other devices related to nervous s	0.0009%	0.0048%	0.0000%	0.0032%
995.55	Shaken infant syndrome	0.0018%	0.0000%	0.0000%	0.0000%
V59.02	Donors of stem cells	0.0018%	0.0000%	0.0000%	0.0000%
996.04	Mechanical complication due to automatic implantable card	0.0005%	0.0048%	0.0000%	0.0064%
983.2	Toxic effect of caustic alkalis	0.0009%	0.0048%	0.0000%	0.0032%
941.36	Burn of scalp (any part), full-thickness skin loss (third degr	0.0014%	0.0048%	0.0000%	0.0000%
996.85	Complications of transplanted bone marrow	0.0005%	0.0000%	0.0000%	0.0096%
910.0	Superficial injury of face, neck and scalp except eye, abras	0.0014%	0.0000%	0.0021%	0.0000%
914.0	Superficial injury of hand(s) except finger(s) alone, abrasio	0.0014%	0.0000%	0.0021%	0.0000%
903.5	Injury to digital blood vessels	0.0014%	0.0000%	0.0021%	0.0000%
944.34	Burn of two or more digits, including thumb, full-thickness	0.0018%	0.0000%	0.0000%	0.0000%
944.32	Burn of thumb (nail), full-thickness skin loss (third degree	0.0009%	0.0000%	0.0021%	0.0032%
904.1	Injury to superficial femoral artery	0.0009%	0.0000%	0.0041%	0.0000%
945.49	Burn of multiple sites of lower limb(s), deep necrosis of un	0.0014%	0.0000%	0.0021%	0.0000%
944.37	Burn of wrist, full-thickness skin loss (third degree NOS)	0.0014%	0.0000%	0.0021%	0.0000%
913.0	Superficial injury of elbow, forearm and wrist, abrasion or	0.0018%	0.0000%	0.0000%	0.0000%
V57.1	Other physical therapy	0.0000%	0.0048%	0.0062%	0.0000%

ICD-9-CM	ICD-9-CM	PureO	ContiB	NewB	PastB
894.0	Multiple and unspecified open wound of lower limb, witho	0.0005%	0.0000%	0.0062%	0.0000%
965.4	Poisoning by aromatic analgesics, not elsewhere classified	0.0014%	0.0000%	0.0021%	0.0000%
956.0	Injury to sciatic nerve	0.0009%	0.0000%	0.0041%	0.0000%
943.25	Burn of shoulder, blisters, epidermal loss (second degree)	0.0009%	0.0000%	0.0041%	0.0000%
878.6	Open wound of vagina, without mention of complication, i	0.0018%	0.0000%	0.0000%	0.0000%
958.4	Traumatic shock	0.0009%	0.0000%	0.0041%	0.0000%
962.3	Poisoning by insulins and antidiabetic agents	0.0000%	0.0048%	0.0041%	0.0032%
812.10	Fracture in unspecified part of upper end of humerus, open	0.0009%	0.0000%	0.0021%	0.0032%
802.34	Fracture of mandible, open, ramus, unspecified	0.0014%	0.0000%	0.0000%	0.0032%
874.01	Open wound of larynx, without mention of complication	0.0005%	0.0000%	0.0041%	0.0032%
836.51	Anterior dislocation of tibia, proximal end, closed	0.0014%	0.0000%	0.0021%	0.0000%
867.6	Injury to other specified pelvic organs, without mention of	0.0005%	0.0000%	0.0062%	0.0000%
816.03	Fracture in multiple sites of phalanges, closed	0.0005%	0.0000%	0.0041%	0.0032%
850.2	Concussion with moderate loss of consciousness	0.0009%	0.0048%	0.0021%	0.0000%
873.63	Open wound of tooth (broken), without mention of complic	0.0014%	0.0048%	0.0000%	0.0000%
863.0	Injury to stomach,without mention of open wound into cav	0.0014%	0.0048%	0.0000%	0.0000%
863.43	Injury to descending (left) colon, without mention of open	0.0009%	0.0048%	0.0021%	0.0000%
851.85	Other and unspecified cerebral laceration and contusion wi	0.0014%	0.0000%	0.0021%	0.0000%
844.0	Sprains and strains of lateral collateral ligament of knee	0.0018%	0.0000%	0.0000%	0.0000%
801.76	Fracture of base of skull, open with subarachnoid, subdural	0.0009%	0.0048%	0.0021%	0.0000%
863.45	Injury to rectum,without mention of open wound into cavit	0.0018%	0.0000%	0.0000%	0.0000%
870.4	Penetrating wound of orbit,with foreign body	0.0014%	0.0000%	0.0021%	0.0000%
852.43	Extradural hemorrhage following injury without mention o	0.0005%	0.0048%	0.0041%	0.0000%
861.10	Unspecified injury to heart,with open wound into thorax	0.0018%	0.0000%	0.0000%	0.0000%
863.99	Injury to other gastrointestinal sites, with open wound into	0.0014%	0.0000%	0.0021%	0.0000%
815.12	Fracture of base of other metacarpal bone(s), open	0.0014%	0.0048%	0.0000%	0.0000%
841.0	Sprains and strains of radial collateral ligament	0.0009%	0.0000%	0.0021%	0.0032%
825.30	Fracture of unspecified bone(s) of foot〔except toes〕, ope	0.0014%	0.0000%	0.0021%	0.0000%
810.12	Fracture in shaft of clavicle, open	0.0014%	0.0000%	0.0021%	0.0000%
806.21	T1- T6 level fracture with complete lesion of cord, closed	0.0005%	0.0000%	0.0062%	0.0000%
808.42	Fracture of ischium, closed	0.0014%	0.0000%	0.0021%	0.0000%
840.1	Sprains and strains of coracoclavicular (ligament)	0.0014%	0.0000%	0.0000%	0.0032%
873.62	Open wound of gum (alveolar process), without mention of	0.0014%	0.0000%	0.0021%	0.0000%
863.40	Injury to colon,unspecified site,without mention of open wo	0.0014%	0.0048%	0.0000%	0.0000%
820.31	Fracture of intertrochanteric section of femur, open	0.0005%	0.0048%	0.0041%	0.0000%
825.32	Fracture of navicular〔scaphoid〕of foot, open	0.0009%	0.0000%	0.0041%	0.0000%
863.44	Injury to sigmoid colon,without mention of open wound int	0.0014%	0.0048%	0.0000%	0.0000%
748.0	Choanal atresia	0.0009%	0.0048%	0.0021%	0.0000%
788.41	Urinary frequency	0.0018%	0.0000%	0.0000%	0.0000%

ICD-9-CM	ICD-9-CM	PureO	ContiB	NewB	PastB
759.82	Marfan’ s syndrome	0.0009%	0.0000%	0.0041%	0.0000%
789.34	Abdominal or pelvic swelling, mass, or lump, left lower qu	0.0014%	0.0000%	0.0000%	0.0032%
780.52	Other insomnia	0.0009%	0.0000%	0.0021%	0.0032%
789.39	Abdominal or pelvic swelling, mass, or lump, other specifi	0.0009%	0.0048%	0.0000%	0.0032%
736.31	Coxa valga (acquired)	0.0018%	0.0000%	0.0000%	0.0000%
786.01	Hyperventilation	0.0000%	0.0097%	0.0021%	0.0032%
800.66	Fracture of vault of skull, open with cerebral laceration and	0.0009%	0.0000%	0.0041%	0.0000%
790.92	Abnormal coagulation profile	0.0009%	0.0000%	0.0000%	0.0064%
789.02	Abdominal pain, left upper quadrant	0.0009%	0.0000%	0.0021%	0.0032%
791.0	Proteinuria	0.0014%	0.0000%	0.0000%	0.0032%
730.21	Unspecified osteomyelitis, shoulder region	0.0009%	0.0000%	0.0041%	0.0000%
730.22	Unspecified osteomyelitis, upper arm	0.0005%	0.0000%	0.0021%	0.0064%
730.14	Chronic osteomyelitis, hand	0.0009%	0.0000%	0.0021%	0.0032%
799.4	Cachexia	0.0005%	0.0000%	0.0000%	0.0096%
736.75	Cavovarus deformity of foot, acquired	0.0018%	0.0000%	0.0000%	0.0000%
744.47	Preauricular cyst	0.0009%	0.0000%	0.0000%	0.0064%
753.11	Congenital single renal cyst	0.0014%	0.0000%	0.0021%	0.0000%
757.32	Vascular hamartomas	0.0018%	0.0000%	0.0000%	0.0000%
752.11	Embryonic cyst of fallopian tubes and broad ligaments	0.0014%	0.0000%	0.0000%	0.0032%
786.52	Painful respiration	0.0005%	0.0048%	0.0000%	0.0064%
753.15	Renal dysplasia	0.0009%	0.0000%	0.0021%	0.0032%
742.3	Congenital hydrocephalus	0.0018%	0.0000%	0.0000%	0.0000%
746.1	Tricuspid atresia and stenosis, congenital	0.0018%	0.0000%	0.0000%	0.0000%
800.14	Fracture of vault of skull, closed with cerebral laceration ar	0.0014%	0.0000%	0.0021%	0.0000%
730.12	Chronic osteomyelitis, upper arm	0.0009%	0.0048%	0.0021%	0.0000%
752.8	Other specified anomalies of genital organs	0.0014%	0.0000%	0.0000%	0.0032%
728.3	Other specific muscle disorders	0.0014%	0.0048%	0.0000%	0.0000%
800.20	Fracture of vault of skull, closed with subarachnoid, subdur	0.0014%	0.0048%	0.0000%	0.0000%
745.69	Other endocardial cushion defects	0.0018%	0.0000%	0.0000%	0.0000%
744.41	Branchial cleft sinus or fistula	0.0018%	0.0000%	0.0000%	0.0000%
733.40	Aseptic necrosis of bone, site unspecified	0.0005%	0.0000%	0.0041%	0.0032%
674.84	Other complications of the puerperium, not elsewaere class	0.0014%	0.0000%	0.0021%	0.0000%
727.49	Other ganglion and cyst of synovium, tendon, and bursa	0.0014%	0.0000%	0.0021%	0.0000%
667.04	Retained placenta without hemorrhage, postpartum conditio	0.0018%	0.0000%	0.0000%	0.0000%
642.53	Severe pre-eclampsia, antepartum condition or complicatio	0.0018%	0.0000%	0.0000%	0.0000%
708.0	Allergic urticaria	0.0014%	0.0048%	0.0000%	0.0000%
711.02	Pyogenic arthritis, upper arm	0.0014%	0.0000%	0.0000%	0.0032%
653.11	Generally contracted pelvis, delivered,with or without men	0.0018%	0.0000%	0.0000%	0.0000%
718.17	Loose body in joint, ankle and foot	0.0009%	0.0000%	0.0041%	0.0000%

ICD-9-CM	ICD-9-CM	PureO	ContiB	NewB	PastB
648.22	Anemia conditions in the mother classifiable elsewhere, bu	0.0014%	0.0000%	0.0000%	0.0032%
718.25	Pathological dislocation, pelvic region and thigh	0.0018%	0.0000%	0.0000%	0.0000%
726.69	Other enthesopathy of knee	0.0014%	0.0000%	0.0021%	0.0000%
719.87	Other specified disorders of joint, ankle and foot	0.0014%	0.0000%	0.0000%	0.0032%
727.06	Tenosynovitis of foot and ankle	0.0014%	0.0000%	0.0000%	0.0032%
680.1	Carbuncle and furuncle, neck	0.0009%	0.0097%	0.0000%	0.0000%
718.56	Ankylosis of joint, lower leg	0.0018%	0.0000%	0.0000%	0.0000%
665.31	Laceration of cervix, delivered, with or without mention of	0.0018%	0.0000%	0.0000%	0.0000%
654.61	Other congenital or acquired abnormality of cervix, deliver	0.0018%	0.0000%	0.0000%	0.0000%
666.02	Thire-stage hemorrhage, delivered, with mention of postpa	0.0018%	0.0000%	0.0000%	0.0000%
718.97	Unspecified derangement of joint, ankle and foot	0.0018%	0.0000%	0.0000%	0.0000%
718.34	Recurrent dislocation of joint, hand	0.0009%	0.0000%	0.0021%	0.0032%
692.82	Dermatitis due to other radiation	0.0018%	0.0000%	0.0000%	0.0000%
701.9	Unspecified hypertrophic and atrophic conditions of skin	0.0018%	0.0000%	0.0000%	0.0000%
719.25	Villonodular synovitis, pelvic region and thigh	0.0014%	0.0048%	0.0000%	0.0000%
680.8	Carbuncle and furuncle, other specified sites	0.0009%	0.0000%	0.0021%	0.0032%
726.70	Enthesopathy of ankle and tarsus, unspecified	0.0018%	0.0000%	0.0000%	0.0000%
654.01	Congenital abnormalities of uterus, delivered, with or with	0.0018%	0.0000%	0.0000%	0.0000%
674.82	Other complications of the puerperium, not elsewaere class	0.0018%	0.0000%	0.0000%	0.0000%
707.11	Ulcer of thigh	0.0005%	0.0000%	0.0021%	0.0064%
727.1	Bunion	0.0018%	0.0000%	0.0000%	0.0000%
715.23	Osteoarthrosis, localized, secondary, forearm	0.0009%	0.0000%	0.0021%	0.0032%
718.55	Ankylosis of joint, pelvic region and thigh	0.0009%	0.0048%	0.0000%	0.0032%
723.1	Cervicalgia	0.0000%	0.0000%	0.0021%	0.0096%
635.11	Legally induced abortion, complicated by delayed or exces	0.0018%	0.0000%	0.0000%	0.0000%
581.89	Nephrotic syndrome, with other specified pathological lesi	0.0009%	0.0048%	0.0000%	0.0032%
639.2	Complications following abortion and ectopic and molar pr	0.0018%	0.0000%	0.0000%	0.0000%
523.1	Chronic gingivitis	0.0014%	0.0000%	0.0000%	0.0032%
531.60	Gastric ulcer, chronic or unspecified with hemorrhage and	0.0009%	0.0000%	0.0041%	0.0000%
572.4	Hepatorenal syndrome	0.0018%	0.0000%	0.0000%	0.0000%
527.9	Unspecified disease of the salivary glands	0.0014%	0.0048%	0.0000%	0.0000%
641.93	Unspecified antepartum hemorrhage, antepartum condition	0.0018%	0.0000%	0.0000%	0.0000%
537.4	Fistula of stomach or duodenum	0.0014%	0.0000%	0.0000%	0.0032%
616.2	Cyst of bartholin's gland	0.0018%	0.0000%	0.0000%	0.0000%
620.3	Acquired atrophy of ovary and fallopian tube	0.0018%	0.0000%	0.0000%	0.0000%
618.6	Vaginal enterocele, congenital or acquired	0.0014%	0.0000%	0.0021%	0.0000%
607.84	Impotence of organic origin	0.0014%	0.0000%	0.0021%	0.0000%
537.83	Angiodysplasia of stomach and duodenum with hemorrhag	0.0009%	0.0000%	0.0000%	0.0064%
596.59	Other functional disorder of bladder	0.0005%	0.0048%	0.0021%	0.0032%

ICD-9-CM	ICD-9-CM	PureO	ContiB	NewB	PastB
578.0	Hematemesis	0.0009%	0.0048%	0.0021%	0.0000%
524.11	Maxillary asymmetry	0.0014%	0.0000%	0.0000%	0.0032%
627.1	Postmenopausal bleeding	0.0018%	0.0000%	0.0000%	0.0000%
622.7	Mucous polyp of cervix	0.0014%	0.0000%	0.0021%	0.0000%
524.69	Other specified temporomandibular joint disorders	0.0014%	0.0048%	0.0000%	0.0000%
553.29	Other ventral hernia	0.0018%	0.0000%	0.0000%	0.0000%
596.7	Hemorrhage into bladder wall	0.0014%	0.0000%	0.0000%	0.0032%
524.06	Microgenia	0.0018%	0.0000%	0.0000%	0.0000%
371.03	Central opacity of cornea	0.0014%	0.0048%	0.0000%	0.0000%
374.41	Lid retraction or lag	0.0014%	0.0000%	0.0000%	0.0032%
365.9	Unspecified glaucoma	0.0014%	0.0000%	0.0021%	0.0000%
386.11	Benign paroxysmal positional vertigo	0.0005%	0.0000%	0.0041%	0.0032%
478.70	Unspecified disease of larynx	0.0014%	0.0000%	0.0021%	0.0000%
386.12	Vestibular neuronitis	0.0005%	0.0097%	0.0000%	0.0032%
427.61	Supraventricular premature beats	0.0000%	0.0097%	0.0021%	0.0032%
371.70	Corneal deformity, unspecified	0.0009%	0.0000%	0.0041%	0.0000%
381.7	Patulous Eustachian tube	0.0009%	0.0000%	0.0000%	0.0064%
446.29	Other specified hypersensitivity angiitis	0.0014%	0.0048%	0.0000%	0.0000%
382.3	Unspecified chronic suppurative otitis media	0.0009%	0.0000%	0.0021%	0.0032%
447.2	Rupture of artery	0.0005%	0.0048%	0.0041%	0.0000%
383.01	Subperiosteal abscess of mastoid	0.0000%	0.0048%	0.0000%	0.0096%
447.8	Other specified disorders of arteries and arterioles	0.0014%	0.0000%	0.0000%	0.0032%
505	Pneumoconiosis, unspecified	0.0009%	0.0000%	0.0021%	0.0032%
380.30	Disorders of pinna, unspecified	0.0009%	0.0000%	0.0000%	0.0064%
427.9	Cardiac dysrhythmia, unspecified	0.0009%	0.0000%	0.0021%	0.0032%
376.47	Deformity due to trauma or surgery	0.0014%	0.0000%	0.0021%	0.0000%
414.00	Coronary atherosclerosis of unspecified type vessel, native	0.0009%	0.0000%	0.0021%	0.0032%
410.61	Acute true posterior wall myocardial infarction, initial epis	0.0009%	0.0048%	0.0021%	0.0000%
278.00	Obesity unspecified	0.0014%	0.0000%	0.0000%	0.0032%
236.91	Neoplasm of uncertain behavior of kidney and ureter	0.0014%	0.0000%	0.0000%	0.0032%
246.3	Hemorrhage and infarction of thyroid	0.0009%	0.0000%	0.0021%	0.0032%
324.9	Intracranial and intraspinal abscess, unspecified site	0.0014%	0.0000%	0.0021%	0.0000%
310.2	Postconcussion syndrome	0.0009%	0.0000%	0.0021%	0.0032%
331.0	Alzheimer's disease	0.0005%	0.0048%	0.0021%	0.0032%
295.34	Schizophrenic disorders, paranoid type, chronic with acute	0.0000%	0.0097%	0.0000%	0.0064%
221.1	Benign neoplasm of vagina	0.0014%	0.0000%	0.0000%	0.0032%
275.1	Disorders of copper metabolism	0.0014%	0.0000%	0.0021%	0.0000%
332.1	Secondary Parkinsonism	0.0000%	0.0097%	0.0021%	0.0032%
276.9	Electrolyte and fluid disorders, not elsewhere classified	0.0000%	0.0145%	0.0021%	0.0000%

ICD-9-CM	ICD-9-CM	PureO	ContiB	NewB	PastB
333.0	Other degenerative diseases of the basal ganglia	0.0009%	0.0048%	0.0021%	0.0000%
311	Depressive disorder, not elsewhere classified	0.0005%	0.0000%	0.0062%	0.0000%
223.81	Benign neoplasm of urethra	0.0018%	0.0000%	0.0000%	0.0000%
295.32	Schizophrenic disorders, paranoid type, chronic	0.0000%	0.0048%	0.0000%	0.0096%
219.8	Benign neoplasm of other specified parts of uterus	0.0018%	0.0000%	0.0000%	0.0000%
205.30	Myeloid sarcoma, without mention of remission	0.0000%	0.0048%	0.0021%	0.0064%
215.9	Benign neoplasm of connective and other soft tissue, site u	0.0009%	0.0048%	0.0000%	0.0032%
296.24	Major depressive disorder, single episode, severe specified	0.0000%	0.0145%	0.0000%	0.0032%
290.0	Senile dementia, uncomplicated	0.0000%	0.0048%	0.0021%	0.0064%
250.13	Diabetes with ketoacidosis, Type I [insulin dependent type]	0.0014%	0.0000%	0.0000%	0.0032%
235.8	Neoplasm of uncertain behavior of pleura, thymus and med	0.0018%	0.0000%	0.0000%	0.0000%
356.8	Other specified idiopathic peripheral neuropathy	0.0005%	0.0000%	0.0062%	0.0000%
235.9	Neoplasm of uncertain behavior of other and unspecified re	0.0014%	0.0048%	0.0000%	0.0000%
357.8	Other inflammatory and toxic neuropathy	0.0009%	0.0048%	0.0021%	0.0000%
205.20	Subacute myeloid leukemia, without mention of remission	0.0000%	0.0048%	0.0062%	0.0000%
237.6	Neoplasm of uncertain behavior of meninges	0.0009%	0.0000%	0.0000%	0.0064%
214.9	Lipoma, unspecified site	0.0009%	0.0000%	0.0021%	0.0032%
360.41	Blind hypotensive eye	0.0018%	0.0000%	0.0000%	0.0000%
251.8	Other specified disorders of pancreatic internal secretion	0.0005%	0.0048%	0.0021%	0.0032%
236.3	Neoplasm of uncertain behavior of other and unspecified fe	0.0009%	0.0000%	0.0000%	0.0064%
233.9	Carcinoma in situ of other and unspecified urinary organs	0.0009%	0.0000%	0.0000%	0.0064%
230.1	Carcinoma in situ of esophagus	0.0014%	0.0048%	0.0000%	0.0000%
176.8	Kaposi's sarcoma of other specified sites	0.0014%	0.0000%	0.0000%	0.0032%
017.92	Tuberculosis of other specified organ, bacteriological or his	0.0009%	0.0048%	0.0021%	0.0000%
194.3	Malignant neoplasm of pituitary gland and craniopharyngea	0.0009%	0.0000%	0.0000%	0.0064%
011.90	Pulmonary tuberculosis, unspecified	0.0009%	0.0048%	0.0021%	0.0000%
011.63	Tuberculous pneumonia〔any form〕, tubercle bacilli foun	0.0005%	0.0048%	0.0021%	0.0032%
117.7	Zygomycosis〔Phycomycosis or Mucormycosis〕	0.0009%	0.0048%	0.0021%	0.0000%
191.0	Malignant neoplasm of cerebrum, except lobes and ventricl	0.0009%	0.0000%	0.0041%	0.0000%
136.1	Behcet's syndrome	0.0005%	0.0097%	0.0021%	0.0000%
197.3	Secondary malignant neoplasm of other respiratory organs	0.0009%	0.0048%	0.0021%	0.0000%
159.1	Malignant neoplasm of spleen, not elsewhere classified	0.0005%	0.0000%	0.0021%	0.0064%
202.11	Mycosis fungoides, lymph nodes of head, face and neck	0.0018%	0.0000%	0.0000%	0.0000%
159.9	Malignant neoplasm of ill-defined sites within the digestive	0.0009%	0.0000%	0.0041%	0.0000%
203.80	Other immunoproliferative neoplasms, without mention of	0.0009%	0.0000%	0.0021%	0.0032%
160.3	Malignant neoplasm of ethmoidal sinus	0.0009%	0.0000%	0.0021%	0.0032%
146.7	Malignant neoplasm of posterior wall of oropharynx	0.0005%	0.0000%	0.0062%	0.0000%
006.3	Amebic liver abscess	0.0018%	0.0000%	0.0000%	0.0000%
191.4	Malignant neoplasm of occipital lobe	0.0005%	0.0097%	0.0000%	0.0032%

ICD-9-CM	ICD-9-CM	PureO	ContiB	NewB	PastB
006.9	Amebiasis, unspecified	0.0014%	0.0048%	0.0000%	0.0000%
014.05	Tuberculous peritonitis, tubercle bacilli not found by bacte	0.0018%	0.0000%	0.0000%	0.0000%
171.9	Malignant neoplasm of connective and other soft tissue, sit	0.0005%	0.0048%	0.0041%	0.0000%
112.4	Candidiasis of lung	0.0000%	0.0097%	0.0041%	0.0000%
173.0	Malignant neoplasm of skin of lip	0.0014%	0.0000%	0.0021%	0.0000%
201.98	Hodgkin's disease, unspecified, lymph nodes of multiple si	0.0005%	0.0000%	0.0021%	0.0064%
048	Other enterovirus diseases of central nervous system	0.0014%	0.0000%	0.0000%	0.0032%
202.30	Malignant histiocytosis, unspecified site, extranodal solid c	0.0009%	0.0048%	0.0000%	0.0032%
013.00	Tuberculous meningitis, unspecified	0.0000%	0.0000%	0.0062%	0.0032%
203.10	Plasma cell leukemia, without mention of remission	0.0009%	0.0000%	0.0041%	0.0000%
175.0	Malignant neoplasm of male breast, nipple and areola	0.0014%	0.0000%	0.0000%	0.0032%
176.0	Kaposi's sarcoma of skin	0.0005%	0.0048%	0.0041%	0.0000%
008.5	Bacterial enteritis, unspecified	0.0014%	0.0000%	0.0000%	0.0032%
897.2	Traumatic amputation, unilateral, at or above knee, without	0.0000%	0.0000%	0.0062%	0.0000%
924.20	Contusion of foot	0.0009%	0.0000%	0.0021%	0.0000%
V55.1	Attention to gastrostomy	0.0005%	0.0048%	0.0000%	0.0032%
941.37	Burn of forehead and cheek, full-thickness skin loss (third	0.0014%	0.0000%	0.0000%	0.0000%
941.40	Burn of face and head, unspecified site deep necrosis of un	0.0014%	0.0000%	0.0000%	0.0000%
908.9	Late effect of unspecified injury	0.0005%	0.0000%	0.0021%	0.0032%
V25.2	Sterilization	0.0009%	0.0000%	0.0000%	0.0032%
947.8	Burn of other internal organs	0.0014%	0.0000%	0.0000%	0.0000%
941.26	Burn of scalp (any part), blisters, epidermal loss (second de	0.0009%	0.0048%	0.0000%	0.0000%
950.0	Optic nerve injury	0.0009%	0.0000%	0.0021%	0.0000%
927.09	Crushing injury of multiple sites of shoulder and upper arm	0.0014%	0.0000%	0.0000%	0.0000%
952.01	C1-C4 level with complete lesion of spinal cord	0.0000%	0.0048%	0.0041%	0.0000%
997.61	Neuroma of amputation stump	0.0014%	0.0000%	0.0000%	0.0000%
902.41	Injury to renal artery	0.0009%	0.0000%	0.0021%	0.0000%
998.4	Foreign body accidentally left during a procedure	0.0014%	0.0000%	0.0000%	0.0000%
955.4	Injury to musculocutaneous nerve	0.0005%	0.0000%	0.0041%	0.0000%
940.2	Alkaline chemical burn of cornea and conjunctival sac	0.0014%	0.0000%	0.0000%	0.0000%
902.0	Injury to abdominal aorta	0.0014%	0.0000%	0.0000%	0.0000%
944.43	Burn two or more digits, not including thumb, deep necrosi	0.0009%	0.0000%	0.0021%	0.0000%
921.2	Contusion of orbital tissues	0.0014%	0.0000%	0.0000%	0.0000%
995.2	Unspecified adverse effect of drug, medicinal and biologica	0.0009%	0.0000%	0.0000%	0.0032%
902.89	Other injury to other specified blood vessels of abdomen an	0.0014%	0.0000%	0.0000%	0.0000%
880.23	Open wound of upper arm, with tendon involvement	0.0009%	0.0000%	0.0021%	0.0000%
959.6	Injury of hip and thigh	0.0014%	0.0000%	0.0000%	0.0000%
881.21	Open wound of elbow, with tendon involvement	0.0009%	0.0000%	0.0021%	0.0000%
943.35	Burn of shoulder, full-thickness skin loss (third degree NO	0.0009%	0.0000%	0.0000%	0.0032%

ICD-9-CM	ICD-9-CM	PureO	ContiB	NewB	PastB
874.02	Open wound of trachea, without mention of complication	0.0014%	0.0000%	0.0000%	0.0000%
963.3	Poisoning by alkalizing agents	0.0014%	0.0000%	0.0000%	0.0000%
997.99	Other complications affecting other specified body system,	0.0009%	0.0000%	0.0021%	0.0000%
943.40	Burn of unspecified site of upper limb, deep necrosis of un	0.0009%	0.0000%	0.0021%	0.0000%
935.0	Foreign body in mouth	0.0009%	0.0000%	0.0021%	0.0000%
966.1	Poisoning by hydantoin derivatives	0.0000%	0.0000%	0.0041%	0.0032%
998.82	Cataract fragments in eye following cataract surgery	0.0014%	0.0000%	0.0000%	0.0000%
966.4	Poisoning by anti-Parkinsonism drugs	0.0005%	0.0000%	0.0041%	0.0000%
V30.01	Single liveborn, born in hospital, delivered by cesarean del	0.0014%	0.0000%	0.0000%	0.0000%
967.8	Poisoning by other sedatives and hypnotics	0.0005%	0.0000%	0.0021%	0.0032%
V52.4	Fitting and adjustment of breast prosthesis and implant	0.0014%	0.0000%	0.0000%	0.0000%
967.9	Poisoning by unspecified sedative or hypnotic	0.0000%	0.0000%	0.0041%	0.0032%
900.03	Injury to internal carotid artery	0.0005%	0.0000%	0.0021%	0.0032%
923.3	Contusion of finger	0.0014%	0.0000%	0.0000%	0.0000%
900.81	Injury to external jugular vein	0.0009%	0.0000%	0.0021%	0.0000%
945.02	Burn of foot, unspecified degree	0.0009%	0.0000%	0.0000%	0.0032%
941.27	Burn of forehead and cheek, blisters, epidermal loss (secon	0.0009%	0.0000%	0.0021%	0.0000%
990	Effects of radiation, unspecified	0.0009%	0.0000%	0.0021%	0.0000%
887.4	Traumatic amputation of arm and hand (complete) (partial)	0.0014%	0.0000%	0.0000%	0.0000%
821.39	Other fracture of lower end of femur, open	0.0005%	0.0000%	0.0041%	0.0000%
872.61	Open wound of ear drum, without mention of complication	0.0009%	0.0000%	0.0000%	0.0032%
833.03	Closed dislocation of midcarpal (joint)	0.0005%	0.0048%	0.0000%	0.0032%
862.1	Injury to diaphragm,with open wound into cavity	0.0005%	0.0000%	0.0041%	0.0000%
873.30	Open wound to unspecified site of nose, complicated	0.0005%	0.0048%	0.0021%	0.0000%
838.09	Other closed dislocation of foot	0.0014%	0.0000%	0.0000%	0.0000%
806.62	Fracture of sacrum and coccyx with other cauda equina inj	0.0005%	0.0097%	0.0000%	0.0000%
814.08	Fracture of hamate (unciform) bone, closed	0.0009%	0.0000%	0.0000%	0.0032%
812.53	Fracture of medial condyle of humerus, open	0.0005%	0.0000%	0.0041%	0.0000%
863.31	Injury to duodenum,with open wound into cavity	0.0005%	0.0048%	0.0021%	0.0000%
839.69	Dislocations of other location, closed	0.0000%	0.0000%	0.0062%	0.0000%
813.17	Other and unspecified fractures of proximal end of radius(a	0.0014%	0.0000%	0.0000%	0.0000%
813.91	Fracture in unspecified part of radius (alone), open	0.0009%	0.0000%	0.0021%	0.0000%
806.09	C5-C7 level fracture with other specified spinal cord injury	0.0000%	0.0097%	0.0021%	0.0000%
873.61	Open wound of buccal mucosa, without mention of compli	0.0014%	0.0000%	0.0000%	0.0000%
847.9	Sprains and strains of unspecified site of back	0.0005%	0.0048%	0.0021%	0.0000%
873.79	Open wound to other and multiple sites of mouth, complica	0.0014%	0.0000%	0.0000%	0.0000%
863.53	Injury to descending (left) colon,with open wound into cav	0.0009%	0.0000%	0.0021%	0.0000%
821.29	Other fracture of lower end of femur, closed	0.0009%	0.0000%	0.0000%	0.0032%
863.81	Injury to head of pancreas, without mention of open wound	0.0009%	0.0048%	0.0000%	0.0000%

ICD-9-CM	ICD-9-CM	PureO	ContiB	NewB	PastB
854.04	Other and unspecified intracranial injury without mention c	0.0009%	0.0000%	0.0021%	0.0000%
839.61	Dislocations of sternum, closed	0.0014%	0.0000%	0.0000%	0.0000%
854.06	Other and unspecified intracranial injury without mention c	0.0014%	0.0000%	0.0000%	0.0000%
805.8	Unspecified fracture of vertebra column, closed	0.0009%	0.0000%	0.0000%	0.0032%
873.22	Open wound of nasal cavity, without mention of complicat	0.0014%	0.0000%	0.0000%	0.0000%
825.33	Fracture of cuboid, open	0.0014%	0.0000%	0.0000%	0.0000%
813.82	Fracture in unspecified part of ulna (alone), closed	0.0014%	0.0000%	0.0000%	0.0000%
825.39	Fracture of other tarsal and metatarsal bones, open	0.0009%	0.0000%	0.0021%	0.0000%
873.51	Open wound of cheek, complicated	0.0014%	0.0000%	0.0000%	0.0000%
814.10	unspecified fracture of carpal bone, open	0.0005%	0.0000%	0.0041%	0.0000%
842.12	Sprains and strains of metacarpophalangeal (joint)	0.0014%	0.0000%	0.0000%	0.0000%
821.22	Fracture of epiphysis of lower end (separation) of femur, cl	0.0009%	0.0000%	0.0021%	0.0000%
861.20	Unspecified injury to lung,without mention of open wound	0.0009%	0.0000%	0.0021%	0.0000%
866.03	Complete disruption of kidney parenchyma, without mentic	0.0009%	0.0000%	0.0021%	0.0000%
814.02	Fracture of lunate (semilunar) bone of wrist, closed	0.0009%	0.0000%	0.0021%	0.0000%
832.10	Unspecified open dislocation of elbow	0.0009%	0.0000%	0.0021%	0.0000%
825.23	Fracture of cuboid, closed	0.0014%	0.0000%	0.0000%	0.0000%
807.6	Fracture of larynx and trachea, open	0.0009%	0.0000%	0.0021%	0.0000%
728.83	Rupture of muscle, nontraumatic	0.0014%	0.0000%	0.0000%	0.0000%
733.16	Pathologic fracture of tibia or fibula	0.0005%	0.0000%	0.0021%	0.0032%
754.33	Congenital subluxation of hip, bilateral	0.0009%	0.0000%	0.0021%	0.0000%
755.29	Longitudinal deficiency, phalanges, complete or partial	0.0014%	0.0000%	0.0000%	0.0000%
747.22	Atresia and stenosis of aorta	0.0014%	0.0000%	0.0000%	0.0000%
742.4	Other specified anomalies of brain	0.0014%	0.0000%	0.0000%	0.0000%
753.8	Other specified anomalies of bladder and urethra	0.0005%	0.0000%	0.0041%	0.0000%
755.36	Longitudinal deficiency, tibia, complete or parital(with or v	0.0014%	0.0000%	0.0000%	0.0000%
801.00	Fracture of base of skull, closed without mention of intracr	0.0014%	0.0000%	0.0000%	0.0000%
755.37	Longitudinal deficiency, fibular, complete or parital(with o	0.0009%	0.0000%	0.0021%	0.0000%
748.69	Other anomalies of lung	0.0014%	0.0000%	0.0000%	0.0000%
755.50	Unspecified anomaly of upper limb	0.0014%	0.0000%	0.0000%	0.0000%
788.9	Other symptoms involving urinary system	0.0014%	0.0000%	0.0000%	0.0000%
755.57	Macrodactylia (fingers)	0.0014%	0.0000%	0.0000%	0.0000%
753.5	Exstrophy of urinary bladder	0.0014%	0.0000%	0.0000%	0.0000%
755.66	Other anomalies of toes	0.0014%	0.0000%	0.0000%	0.0000%
800.02	Fracture of vault of skull, closed without mention of intracr	0.0009%	0.0000%	0.0021%	0.0000%
756.51	Osteogenesis imperfecta	0.0014%	0.0000%	0.0000%	0.0000%
800.33	Fracture of vault of skull, closed with other and unspecifiec	0.0005%	0.0000%	0.0041%	0.0000%
730.96	Unspecified infection of bone, lower leg	0.0014%	0.0000%	0.0000%	0.0000%
754.71	Talipes cavus	0.0014%	0.0000%	0.0000%	0.0000%

ICD-9-CM	ICD-9-CM	PureO	ContiB	NewB	PastB
727.65	Rupture of quadriceps tendon	0.0009%	0.0000%	0.0000%	0.0032%
747.11	Interruption of aortic arch	0.0014%	0.0000%	0.0000%	0.0000%
767.5	Facial nerve injury	0.0009%	0.0000%	0.0021%	0.0000%
788.1	Dysuria	0.0009%	0.0048%	0.0000%	0.0000%
771.8	Other infection specific to the perinatal period	0.0014%	0.0000%	0.0000%	0.0000%
744.3	Unspecified anomaly of ear	0.0009%	0.0000%	0.0021%	0.0000%
779.5	Drug withdrawal syndrome in newborn	0.0014%	0.0000%	0.0000%	0.0000%
753.19	Other specified cystic kidney disease	0.0014%	0.0000%	0.0000%	0.0000%
727.66	Rupture of patellar tendon	0.0009%	0.0000%	0.0021%	0.0000%
747.61	Gastrointestinal vessel anomaly	0.0009%	0.0000%	0.0000%	0.0032%
746.2	Ebstein's anomaly	0.0009%	0.0000%	0.0000%	0.0032%
790.99	Other nonspecific findings on examination of blood	0.0005%	0.0048%	0.0000%	0.0032%
743.62	Congenital deformities of eyelids	0.0014%	0.0000%	0.0000%	0.0000%
730.11	Chronic osteomyelitis, shoulder region	0.0014%	0.0000%	0.0000%	0.0000%
750.9	Unspecified anomalies of upper alimentary tract	0.0014%	0.0000%	0.0000%	0.0000%
800.04	Fracture of vault of skull, closed without mention of intracr	0.0009%	0.0048%	0.0000%	0.0000%
735.9	Unspecified acquired deformity of toe	0.0014%	0.0000%	0.0000%	0.0000%
754.41	Congenital dislocation of knee (with genu recurvatum)	0.0009%	0.0000%	0.0021%	0.0000%
744.04	Anomalies of ear ossicles	0.0014%	0.0000%	0.0000%	0.0000%
754.61	Congenital pes planus	0.0014%	0.0000%	0.0000%	0.0000%
727.68	Rupture of other tendons of foot and ankle	0.0014%	0.0000%	0.0000%	0.0000%
801.02	Fracture of base of skull, closed without mention of intracr	0.0014%	0.0000%	0.0000%	0.0000%
732.5	Juvenile osteochondrosis of foot	0.0014%	0.0000%	0.0000%	0.0000%
728.10	Muscular calcification and ossification, unspecified	0.0005%	0.0000%	0.0021%	0.0032%
730.23	Unspecified osteomyelitis, forearm	0.0009%	0.0000%	0.0021%	0.0000%
730.07	Acute osteomyelitis, ankle and foot	0.0014%	0.0000%	0.0000%	0.0000%
728.82	Foreign body granuloma of muscle	0.0005%	0.0048%	0.0000%	0.0032%
755.12	Syndactyly of fingers with fusion of bone	0.0014%	0.0000%	0.0000%	0.0000%
660.61	Failed trail of labor, unspecified, delivered, with or without	0.0014%	0.0000%	0.0000%	0.0000%
726.39	Other enthesopathy of elbow region	0.0009%	0.0000%	0.0000%	0.0032%
700	Corns and callosities	0.0005%	0.0048%	0.0000%	0.0032%
711.03	Pyogenic arthritis, forearm	0.0005%	0.0000%	0.0041%	0.0000%
681.11	Onychia and paronychia of toe	0.0009%	0.0048%	0.0000%	0.0000%
667.14	Retained portions of placenta or membranes, without hemo	0.0014%	0.0000%	0.0000%	0.0000%
722.82	Postlaminectomy syndrome, thoracic region	0.0009%	0.0048%	0.0000%	0.0000%
710.9	Unspecified diffuse connective tissue disease	0.0005%	0.0097%	0.0000%	0.0000%
716.91	Arthropathy, unspecified, shoulder region	0.0009%	0.0000%	0.0000%	0.0032%
719.61	Other symptoms referable to joint, shoulder region	0.0005%	0.0000%	0.0041%	0.0000%
664.14	Second-degree perineal laceration, postpartum condition or	0.0014%	0.0000%	0.0000%	0.0000%

ICD-9-CM	ICD-9-CM	PureO	ContiB	NewB	PastB
658.03	Oligohydramnios, antepartum condition or complication	0.0014%	0.0000%	0.0000%	0.0000%
655.71	Decreased fetal movement, affecting management of mothe	0.0014%	0.0000%	0.0000%	0.0000%
660.81	Other causes of obstructed labor, delivered, with or withou	0.0014%	0.0000%	0.0000%	0.0000%
727.04	Radial styloid tenosynovitis	0.0009%	0.0000%	0.0000%	0.0032%
719.64	Other symptoms referable to joint, hand	0.0009%	0.0048%	0.0000%	0.0000%
651.81	Other specified multiple gestation,delivered, with or withou	0.0014%	0.0000%	0.0000%	0.0000%
674.14	Disruption of cesarean wound, postpartum condition or cor	0.0009%	0.0000%	0.0021%	0.0000%
722.2	Displacement of intervertebral disc, site unspecified, witho	0.0005%	0.0000%	0.0021%	0.0032%
702.19	Other seborrheic keratosis	0.0009%	0.0000%	0.0000%	0.0032%
719.27	Villonodular synovitis, ankle and foot	0.0009%	0.0000%	0.0021%	0.0000%
718.91	Unspecified derangement of joint, shoulder region	0.0014%	0.0000%	0.0000%	0.0000%
659.13	Failed medical or unspecified induction, antepartum condit	0.0009%	0.0048%	0.0000%	0.0000%
718.95	Unspecified derangement of joint, pelvic region and thigh	0.0009%	0.0000%	0.0021%	0.0000%
715.92	Osteoarthrosis, unspecified whether generalized or localize	0.0005%	0.0000%	0.0021%	0.0032%
719.91	Unspecified disorder of joint, shoulder region	0.0014%	0.0000%	0.0000%	0.0000%
719.49	Pain in joint, multiple sites	0.0009%	0.0048%	0.0000%	0.0000%
716.59	Unspecified polyarthropathy or polyarthritis, multiple sites	0.0014%	0.0000%	0.0000%	0.0000%
726.90	Enthesopathy of unspecified site	0.0009%	0.0000%	0.0021%	0.0000%
716.66	Unspecified monoarthritis, lower leg	0.0009%	0.0000%	0.0000%	0.0032%
719.53	Stiffness of joint, not elsewhere classified, forearm	0.0009%	0.0048%	0.0000%	0.0000%
719.82	Other specified disorders of joint, upper arm	0.0014%	0.0000%	0.0000%	0.0000%
663.21	Other and unspecified cord entanglement, with compressio	0.0014%	0.0000%	0.0000%	0.0000%
721.90	Spondylosis of unspecified site, without mention of myelop	0.0009%	0.0048%	0.0000%	0.0000%
643.03	Mild hyperemesis gravidarum, antepartum condition or cor	0.0009%	0.0000%	0.0000%	0.0032%
719.06	Effusion of joint, lower leg	0.0014%	0.0000%	0.0000%	0.0000%
718.04	Articular cartilage disorder, hand	0.0014%	0.0000%	0.0000%	0.0000%
665.74	Pelvic hematoma, postpartum condition or complication	0.0014%	0.0000%	0.0000%	0.0000%
647.83	Other specified infectious and parasitic diseases in the motl	0.0014%	0.0000%	0.0000%	0.0000%
658.43	Infection of amniotic cavity, antepartum condition or comp	0.0009%	0.0000%	0.0000%	0.0032%
670.04	Major puerperal infection, postpartum condition or complic	0.0009%	0.0048%	0.0000%	0.0000%
718.88	Other joint derangement, not elsewhere classified, other sp	0.0005%	0.0000%	0.0041%	0.0000%
525.9	Unspecified disorder of the teeth and supporting structures	0.0009%	0.0000%	0.0000%	0.0032%
596.51	Hypertonicity of bladder	0.0005%	0.0000%	0.0021%	0.0032%
536.9	Unspecified functional disorder of stomach	0.0005%	0.0000%	0.0041%	0.0000%
536.3	Gastroparesis	0.0014%	0.0000%	0.0000%	0.0000%
621.6	Malposition of uterus	0.0009%	0.0000%	0.0021%	0.0000%
625.5	Pelvic congestion syndrome	0.0009%	0.0000%	0.0021%	0.0000%
637.71	Unspecified abortion, with other specified complications, i	0.0014%	0.0000%	0.0000%	0.0000%
532.91	Duodenal ulcer, unspecified as acute or chronic, without m	0.0009%	0.0000%	0.0021%	0.0000%

ICD-9-CM	ICD-9-CM	PureO	ContiB	NewB	PastB
623.0	Dysplasia of vagina	0.0014%	0.0000%	0.0000%	0.0000%
553.9	Hernia of unspecified site	0.0005%	0.0000%	0.0021%	0.0032%
635.01	Legally induced abortion, complicated by genital tract and	0.0014%	0.0000%	0.0000%	0.0000%
556.8	Other ulcerative colitis	0.0009%	0.0000%	0.0000%	0.0032%
635.22	Legally induced abortion, complicated by damage to pelvic	0.0014%	0.0000%	0.0000%	0.0000%
606.9	Male infertility, unspecified	0.0014%	0.0000%	0.0000%	0.0000%
536.2	Persistent vomiting	0.0014%	0.0000%	0.0000%	0.0000%
608.3	Atrophy of testis	0.0014%	0.0000%	0.0000%	0.0000%
532.00	Duodenal ulcer, acute with hemorrhage, without mention o	0.0014%	0.0000%	0.0000%	0.0000%
593.5	Hydroureter	0.0009%	0.0000%	0.0000%	0.0032%
535.01	Acute gastritis, with hemorrhage	0.0014%	0.0000%	0.0000%	0.0000%
627.0	Premenopausal menorrhagia	0.0009%	0.0000%	0.0000%	0.0032%
601.1	Chronic prostatitis	0.0005%	0.0048%	0.0021%	0.0000%
524.9	Unspecified dentofacial anomalies	0.0014%	0.0000%	0.0000%	0.0000%
575.4	Perforation of gallbladder	0.0009%	0.0000%	0.0021%	0.0000%
594.0	Calculus in diverticulum of bladder	0.0014%	0.0000%	0.0000%	0.0000%
525.3	Retained dental root	0.0009%	0.0000%	0.0000%	0.0032%
628.8	Infertility, female, of other specified origin	0.0009%	0.0000%	0.0000%	0.0032%
583.4	Nephritis and nephropathy, not specified as acute or chroni	0.0000%	0.0145%	0.0000%	0.0000%
590.9	Infection of kidney, unspecified	0.0005%	0.0000%	0.0021%	0.0032%
635.71	Legally induced abortion, with other specified complicatior	0.0014%	0.0000%	0.0000%	0.0000%
629.8	Other specified disorders of female genital organs	0.0009%	0.0000%	0.0021%	0.0000%
635.90	Legally induced abortion, without mention of complication	0.0014%	0.0000%	0.0000%	0.0000%
530.84	Tracheoesophageal fistula	0.0005%	0.0000%	0.0041%	0.0000%
556.2	Ulcerative (chronic) proctitis	0.0005%	0.0000%	0.0000%	0.0064%
569.42	Rectal or anal pain	0.0005%	0.0048%	0.0000%	0.0032%
553.01	Femoral hernia, unilateral or unspecified, recurrent	0.0009%	0.0000%	0.0021%	0.0000%
531.51	Gastric ulcer, chronic or unspecified with perforation, with	0.0005%	0.0000%	0.0041%	0.0000%
622.6	Hypertrophic elongation of cervix	0.0005%	0.0000%	0.0041%	0.0000%
524.60	Temporomandibular joint disorders, unspecified	0.0005%	0.0000%	0.0000%	0.0064%
608.9	Unspecified disorders of male genital organs	0.0014%	0.0000%	0.0000%	0.0000%
560.31	Gallstone ileus	0.0009%	0.0000%	0.0021%	0.0000%
622.8	Other specified noninflammatory disorders of cervix	0.0005%	0.0000%	0.0021%	0.0032%
552.3	Diaphragmatic hernia, with obstruction	0.0005%	0.0000%	0.0041%	0.0000%
564.7	Megacolon, other than Hirschsprung's	0.0009%	0.0000%	0.0000%	0.0032%
634.01	Abortion, complicated by genital tract and pelvic infection,	0.0005%	0.0000%	0.0021%	0.0032%
616.50	Ulceration of vulva, unspecified	0.0014%	0.0000%	0.0000%	0.0000%
634.02	Abortion, complicated by genital tract and pelvic infection,	0.0009%	0.0048%	0.0000%	0.0000%
555.2	Regional enteritis, small intestine with large intestine	0.0014%	0.0000%	0.0000%	0.0000%

ICD-9-CM	ICD-9-CM	PureO	ContiB	NewB	PastB
599.89	Other specified disorders of urinary tract	0.0014%	0.0000%	0.0000%	0.0000%
482.84	Legionnaires' disease	0.0009%	0.0000%	0.0021%	0.0000%
376.81	Orbital cysts	0.0014%	0.0000%	0.0000%	0.0000%
511.1	Pleurisy, with effusion, with mention of a bacterial cause o	0.0005%	0.0048%	0.0021%	0.0000%
434.01	Cerebral thrombosis with cerebral infarction	0.0014%	0.0000%	0.0000%	0.0000%
374.10	Ectropion, unspecified	0.0014%	0.0000%	0.0000%	0.0000%
376.30	Exophthalmos, unspecified	0.0009%	0.0048%	0.0000%	0.0000%
389.04	Conductive hearing loss, inner ear	0.0009%	0.0000%	0.0000%	0.0032%
435.2	Subclavian steal syndrome	0.0005%	0.0048%	0.0021%	0.0000%
371.57	Endothelial corneal dystrophy	0.0014%	0.0000%	0.0000%	0.0000%
451.2	Phlebitis and thrombophlebitis of lower extremities, unspec	0.0005%	0.0000%	0.0021%	0.0032%
440.29	Other atherosclerosis of native arteries of the extremities	0.0014%	0.0000%	0.0000%	0.0000%
374.14	Cicatricial ectropion	0.0014%	0.0000%	0.0000%	0.0000%
482.39	Pneumonia due to other Streptococcus	0.0014%	0.0000%	0.0000%	0.0000%
453.9	Embolism and thrombosis of unspecified site	0.0005%	0.0000%	0.0021%	0.0032%
383.02	Acute mastoiditis with other complications	0.0009%	0.0048%	0.0000%	0.0000%
438.20	Hemiplegia affecting unspecified side, late effects of cerebr	0.0005%	0.0000%	0.0021%	0.0032%
426.89	Other specified conduction disorders	0.0014%	0.0000%	0.0000%	0.0000%
438.82	Dysphagia, late effects of cerebrovascular disease	0.0005%	0.0048%	0.0000%	0.0032%
395.0	Rheumatic aortic stenosis	0.0009%	0.0000%	0.0000%	0.0032%
405.91	Unspecified renovascular secondary hypertension	0.0005%	0.0048%	0.0000%	0.0032%
366.00	Nonsenile cataract, unspecified	0.0009%	0.0000%	0.0021%	0.0000%
405.99	Other unspecified secondary hypertension	0.0005%	0.0000%	0.0041%	0.0000%
440.21	Atherosclerosis of the extremities with intermittent claudica	0.0009%	0.0000%	0.0000%	0.0032%
364.3	Unspecified iridocyclitis	0.0005%	0.0000%	0.0041%	0.0000%
372.63	Symblepharon	0.0014%	0.0000%	0.0000%	0.0000%
376.50	Enophthalmos, unspecified as to cause	0.0009%	0.0000%	0.0000%	0.0032%
482.30	Pneumonia due to Streptococcus, unspecified	0.0014%	0.0000%	0.0000%	0.0000%
371.60	Keratoconus, unspecified	0.0005%	0.0000%	0.0041%	0.0000%
424.90	Endocarditis, valve unspecified , unspecified cause	0.0000%	0.0048%	0.0041%	0.0000%
433.20	Occlusion and stenosis of vertebral artery without mention	0.0005%	0.0000%	0.0021%	0.0032%
482.89	Other specified bacteria	0.0009%	0.0000%	0.0021%	0.0000%
433.21	Occlusion and stenosis of carotid artery with cerebral infarc	0.0009%	0.0000%	0.0000%	0.0032%
378.31	Hypertropia	0.0014%	0.0000%	0.0000%	0.0000%
381.3	Other and unspecified chronic nonsuppurative otitis media	0.0014%	0.0000%	0.0000%	0.0000%
383.89	Other disorders of mastoid	0.0009%	0.0000%	0.0021%	0.0000%
438.9	Unspecified late effects of cerebrovascular disease	0.0005%	0.0048%	0.0021%	0.0000%
441.9	Aortic aneurysm of unspecified site without mention of rup	0.0009%	0.0000%	0.0021%	0.0000%
370.8	Other forms of keratitis	0.0014%	0.0000%	0.0000%	0.0000%

ICD-9-CM	ICD-9-CM	PureO	ContiB	NewB	PastB
442.0	Aneurysm of artery of upper extremity	0.0005%	0.0000%	0.0000%	0.0064%
440.0	Atherosclerosis of aorta	0.0009%	0.0000%	0.0000%	0.0032%
429.4	Functional disturbances following cardiac surgery	0.0000%	0.0097%	0.0000%	0.0032%
420.90	Acute pericarditis, unspecified	0.0009%	0.0000%	0.0000%	0.0032%
437.2	Hypertensive encephalopathy	0.0009%	0.0000%	0.0021%	0.0000%
440.20	Atherosclerosis of the extremities, unspecified	0.0009%	0.0000%	0.0000%	0.0032%
432.0	Nontraumatic extradural hemorrhage	0.0014%	0.0000%	0.0000%	0.0000%
478.26	Cyst of pharynx or nasopharynx	0.0009%	0.0000%	0.0021%	0.0000%
437.4	Cerebral arteritis	0.0005%	0.0048%	0.0021%	0.0000%
478.30	Paralysis of vocal cords or larynx, unspecified	0.0005%	0.0000%	0.0021%	0.0032%
461.0	Acute sinusitis of maxillary	0.0014%	0.0000%	0.0000%	0.0000%
411.89	Other acute and subacute forms of ischemic heart disease	0.0009%	0.0000%	0.0000%	0.0032%
250.83	Diabetes with other specified manifestations, Type I [insuli	0.0000%	0.0000%	0.0021%	0.0064%
237.72	Neurofibromatosis, Type Ⅱ [acoustic neurofibromatosis]	0.0005%	0.0000%	0.0021%	0.0032%
250.41	Diabetes with renal manifestations, Type I [insulin depende	0.0009%	0.0048%	0.0000%	0.0000%
286.7	Acquired coagulation factor deficiency	0.0005%	0.0000%	0.0041%	0.0000%
298.9	Unspecified psychosis	0.0000%	0.0145%	0.0000%	0.0000%
236.1	Neoplasm of uncertain behavior of placenta	0.0014%	0.0000%	0.0000%	0.0000%
210.3	Benign neoplasm of floor of mouth	0.0014%	0.0000%	0.0000%	0.0000%
289.0	Polycythemia, secondary	0.0000%	0.0097%	0.0000%	0.0032%
320.82	Meningitis due to Gram-negative bacteria, not elsewhere cl	0.0005%	0.0048%	0.0021%	0.0000%
252.8	Other specified disorders of parathyroid gland	0.0014%	0.0000%	0.0000%	0.0000%
296.44	Bipolar affective disorder, manic, severe specified as with	0.0000%	0.0048%	0.0000%	0.0064%
348.2	Benign intracranial hypertension	0.0000%	0.0048%	0.0021%	0.0032%
306.1	Psychogenic respiratory malfunction	0.0000%	0.0048%	0.0021%	0.0032%
333.2	Myoclonus	0.0000%	0.0000%	0.0000%	0.0096%
292.0	Drug withdrawal syndrome	0.0000%	0.0000%	0.0041%	0.0032%
211.9	Benign neoplasm of other and unspecified site of digestive	0.0005%	0.0000%	0.0000%	0.0064%
345.00	Generalized nonconvulsive epilepsy without mention of int	0.0005%	0.0048%	0.0021%	0.0000%
362.12	Exudative retinopathy	0.0014%	0.0000%	0.0000%	0.0000%
250.90	Diabetes with unspecified complication, Type II [non-insul	0.0009%	0.0000%	0.0000%	0.0032%
273.3	Macroglobulinemia	0.0014%	0.0000%	0.0000%	0.0000%
242.01	Toxic diffuse goiter with mention of thyrotoxic crisis or sto	0.0009%	0.0000%	0.0021%	0.0000%
348.9	Unspecified condition of brain	0.0000%	0.0048%	0.0041%	0.0000%
216.1	Benign neoplasm of eyelid, including canthus	0.0009%	0.0000%	0.0021%	0.0000%
349.0	Reaction to spinal or lumbar puncture	0.0009%	0.0000%	0.0021%	0.0000%
298.1	Excitative type psychosis	0.0000%	0.0145%	0.0000%	0.0000%
333.5	Other choreas	0.0005%	0.0000%	0.0021%	0.0032%
300.4	Neurotic depression	0.0000%	0.0048%	0.0021%	0.0032%

ICD-9-CM	ICD-9-CM	PureO	ContiB	NewB	PastB
349.81	Cerebrospinal fluid rhinorrhea	0.0005%	0.0097%	0.0000%	0.0000%
291.9	Unspecified alcoholic psychosis	0.0000%	0.0097%	0.0021%	0.0000%
238.6	Neoplasm of uncertain behavior of plasma cells	0.0005%	0.0048%	0.0021%	0.0000%
356.2	Hereditary sensory neuropathy	0.0005%	0.0048%	0.0021%	0.0000%
296.23	Major depressive disorder, single episode, severe without n	0.0000%	0.0048%	0.0000%	0.0064%
224.3	Benign neoplasm of conjunctiva	0.0014%	0.0000%	0.0000%	0.0000%
350.8	Other specified trigeminal nerve disorders	0.0009%	0.0048%	0.0000%	0.0000%
285.21	Anemia in end-stage renal disease	0.0000%	0.0048%	0.0000%	0.0064%
286.9	Other and unspecified coagulation defects	0.0005%	0.0000%	0.0021%	0.0032%
294.9	Unspecified organic brain syndrome (chronic)	0.0000%	0.0145%	0.0000%	0.0000%
352.6	Multiple cranial nerve palsies	0.0000%	0.0000%	0.0041%	0.0032%
245.0	Acute thyroiditis	0.0009%	0.0048%	0.0000%	0.0000%
281.3	Other specified megaloblastic anemias, not elsewhere class	0.0000%	0.0048%	0.0000%	0.0064%
345.50	Partial epilepsy, without mention of impairment of conscio	0.0005%	0.0000%	0.0000%	0.0064%
341.9	Demyelinating disease of central nervous system, unspecifi	0.0009%	0.0000%	0.0021%	0.0000%
281.0	Pernicious anemia	0.0005%	0.0000%	0.0021%	0.0032%
275.41	Disorders of calcium metabolism, Hypocalcemia	0.0014%	0.0000%	0.0000%	0.0000%
323.8	Other causes of encephalitis	0.0000%	0.0048%	0.0021%	0.0032%
253.2	Panhypopituitarism	0.0005%	0.0000%	0.0000%	0.0064%
266.2	Other B -complex deficiencies	0.0005%	0.0097%	0.0000%	0.0000%
253.6	Other disorders of neurohypophysis	0.0000%	0.0097%	0.0000%	0.0032%
273.1	Monoclonal paraproteinemia	0.0005%	0.0000%	0.0000%	0.0064%
250.81	Diabetes with other specified manifestations, Type I [insuli	0.0000%	0.0000%	0.0041%	0.0032%
295.90	Unspecified schizophrenia, unspecified	0.0000%	0.0048%	0.0000%	0.0064%
289.8	Other specified diseases of blood and blood-forming organs	0.0005%	0.0048%	0.0021%	0.0000%
112.84	Candidal esophagitis	0.0005%	0.0000%	0.0021%	0.0032%
196.6	Secondary and unspecified malignant neoplasm of intrapelv	0.0014%	0.0000%	0.0000%	0.0000%
161.3	Malignant neoplasm of laryngeal cartilages	0.0005%	0.0000%	0.0021%	0.0032%
074.3	Hand, foot and mouth disease	0.0009%	0.0048%	0.0000%	0.0000%
015.00	Vertebral column tuberculosis, unspecified	0.0005%	0.0000%	0.0041%	0.0000%
156.8	Malignant neoplasm of other specified sites of gallbladder a	0.0005%	0.0000%	0.0041%	0.0000%
070.1	Viral hepatitis A without mention of hepatic coma	0.0009%	0.0048%	0.0000%	0.0000%
083.9	Rickettsiosis, unspecified	0.0000%	0.0048%	0.0021%	0.0032%
194.4	Malignant neoplasm of pineal gland	0.0014%	0.0000%	0.0000%	0.0000%
173.8	Malignant neoplasm of other specified sites of skin	0.0009%	0.0000%	0.0021%	0.0000%
127.2	Strongyloidiasis	0.0000%	0.0048%	0.0021%	0.0032%
142.9	Malignant neoplasm of salivary gland, unspecified	0.0009%	0.0000%	0.0021%	0.0000%
200.18	Lymphosarcoma, lymph nodes of multiple sites	0.0009%	0.0000%	0.0021%	0.0000%
174.6	Malignant neoplasm of female breast, axillary tail	0.0009%	0.0048%	0.0000%	0.0000%

ICD-9-CM	ICD-9-CM	PureO	ContiB	NewB	PastB
201.61	Mixed cellularity, lymph nodes of head, face and neck	0.0009%	0.0000%	0.0000%	0.0032%
157.3	Malignant neoplasm of pancreatic duct	0.0005%	0.0000%	0.0000%	0.0064%
070.20	Viral hepatitis B with hepatic coma, acute or unspecified, v	0.0005%	0.0000%	0.0021%	0.0032%
179	Malignant neoplasm of uterus, part unspecified	0.0014%	0.0000%	0.0000%	0.0000%
027.0	Listeriosis	0.0005%	0.0048%	0.0000%	0.0032%
180.8	Malignant neoplasm of other specified sites of cervix	0.0014%	0.0000%	0.0000%	0.0000%
054.3	Herpetic meningoencephalitis	0.0005%	0.0048%	0.0000%	0.0032%
013.04	Tuberculous meningitis, tubercle bacilli not found (in sputu	0.0000%	0.0048%	0.0041%	0.0000%
014.06	Tuberculous peritonitis, tubercle bacilli not found by bacte	0.0014%	0.0000%	0.0000%	0.0000%
145.1	Malignant neoplasm of vestibule of mouth	0.0005%	0.0000%	0.0041%	0.0000%
054.43	Herpes simplex disciform keratitis	0.0014%	0.0000%	0.0000%	0.0000%
188.0	Malignant neoplasm of trigone of urinary bladder	0.0009%	0.0000%	0.0000%	0.0032%
200.10	Lymphosarcoma, unspecified site, extranodal and solid org	0.0005%	0.0048%	0.0021%	0.0000%
018.93	Miliary tuberculosis, unspecified, tubercle bacilli found (in	0.0009%	0.0048%	0.0000%	0.0000%
200.21	Burkitt's tumor or lymphoma, lymph nodes of head face an	0.0005%	0.0097%	0.0000%	0.0000%
094.9	Neurosyphilis, unspecified	0.0000%	0.0097%	0.0000%	0.0032%
201.60	Mixed cellularity, unspecified site, extranodal solid organ s	0.0009%	0.0000%	0.0021%	0.0000%
053.29	Herpes zoster with other ophthalmic complication	0.0005%	0.0048%	0.0000%	0.0032%
112.89	Candidiasis of other specified sites	0.0005%	0.0000%	0.0021%	0.0032%
189.8	Malignant neoplasm of other specified sites of urinary orga	0.0005%	0.0000%	0.0021%	0.0032%
038.2	Pneumococcal septicemia	0.0014%	0.0000%	0.0000%	0.0000%
146.3	Malignant neoplasm of vallecula	0.0009%	0.0000%	0.0021%	0.0000%
015.04	Vertebral column tuberculosis, tubercle bacilli not found (i	0.0014%	0.0000%	0.0000%	0.0000%
012.03	Tuberculous pleurisy, tubercle bacilli found (in sputum) by	0.0014%	0.0000%	0.0000%	0.0000%
054.0	Eczema herpeticum	0.0014%	0.0000%	0.0000%	0.0000%
015.55	Limb bones tuberculosis, tubercle bacilli not found by bact	0.0014%	0.0000%	0.0000%	0.0000%
V50.49	Prophylactic other organ removal	0.0005%	0.0000%	0.0000%	0.0032%
881.12	Open wound of wrist, complicated	0.0005%	0.0000%	0.0021%	0.0000%
926.0	Crushing injury of external genitalia	0.0009%	0.0000%	0.0000%	0.0000%
915.0	Superficial injury of finger(s), abrasion or friction burn wit	0.0009%	0.0000%	0.0000%	0.0000%
934.8	Foreign body in trachea, bronchus, and lung, other specifie	0.0005%	0.0048%	0.0000%	0.0000%
952.10	T1-T6 level with unspecified spinal cord injury	0.0000%	0.0000%	0.0041%	0.0000%
952.02	C1-C4 level with anterior cord syndrome	0.0000%	0.0000%	0.0041%	0.0000%
952.2	Lumbar cord injury without evidence of spinal bone injury	0.0005%	0.0000%	0.0021%	0.0000%
927.00	Crushing injury of shoulder region	0.0009%	0.0000%	0.0000%	0.0000%
952.9	Unspecified site of spinal cord injury without evidence of s	0.0000%	0.0048%	0.0021%	0.0000%
996.83	Complications of transplanted heart	0.0005%	0.0048%	0.0000%	0.0000%
916.1	Superficial injury of hip, thigh, leg and ankle, abrasion or f	0.0005%	0.0048%	0.0000%	0.0000%
999.2	Complication of medical care, other vascular complications	0.0000%	0.0000%	0.0041%	0.0000%

ICD-9-CM	ICD-9-CM	PureO	ContiB	NewB	PastB
955.5	Injury to cutaneous sensory nerve, upper limb	0.0009%	0.0000%	0.0000%	0.0000%
940.4	Other burn of cornea and conjunctival sac	0.0009%	0.0000%	0.0000%	0.0000%
945.25	Burn of knee, blisters, epidermal loss (second degree)	0.0009%	0.0000%	0.0000%	0.0000%
924.21	Contusion of ankle	0.0005%	0.0000%	0.0021%	0.0000%
955.8	Injury to multiple nerves of shoulder girdle and upper limb	0.0009%	0.0000%	0.0000%	0.0000%
996.55	Mechanical complication due to artificial skin graft and dec	0.0009%	0.0000%	0.0000%	0.0000%
956.2	Injury to posterior tibial nerve	0.0000%	0.0048%	0.0021%	0.0000%
944.21	Burn of single digit (finger nail) other than thumb, blisters,	0.0009%	0.0000%	0.0000%	0.0000%
880.09	Open wound to multiple sites of shoulder and upper arm, w	0.0009%	0.0000%	0.0000%	0.0000%
944.24	Burn of two or more digits, including thumb, blisters, epide	0.0005%	0.0000%	0.0021%	0.0000%
957.8	Injury to multiple nerves in several parts	0.0005%	0.0048%	0.0000%	0.0000%
930.1	Foreign body in conjunctival sac	0.0009%	0.0000%	0.0000%	0.0000%
904.6	Injury to deep plantar blood vessels	0.0009%	0.0000%	0.0000%	0.0000%
942.44	Burn of back (any part), deep necrosis of underlying tissues	0.0005%	0.0048%	0.0000%	0.0000%
921.9	Unspecified contusion of eye	0.0000%	0.0048%	0.0000%	0.0032%
V24.0	Immediately care and examination after delivery	0.0009%	0.0000%	0.0000%	0.0000%
880.12	Open wound of axillary region, complicated	0.0005%	0.0000%	0.0021%	0.0000%
V52.8	Fitting and adjustment of other specified prosthetic device	0.0009%	0.0000%	0.0000%	0.0000%
959.2	Injury of shoulder and upper arm	0.0009%	0.0000%	0.0000%	0.0000%
V55.4	Attention to other artificial opening of digestive tract	0.0000%	0.0000%	0.0000%	0.0064%
959.3	Injury of elbow, forearm and wrist	0.0005%	0.0000%	0.0021%	0.0000%
945.00	Burn of unspecified site of lower limb (leg), unspecified de	0.0005%	0.0000%	0.0021%	0.0000%
904.7	Injury to other specified blood vessels of lower extremity	0.0000%	0.0000%	0.0041%	0.0000%
952.06	C5-C7 level with complete lesion of spinal cord	0.0000%	0.0000%	0.0041%	0.0000%
945.31	Burn of toe(s) (nail), full-thickness skin loss (third degree N	0.0009%	0.0000%	0.0000%	0.0000%
926.12	Crushing injury of buttock	0.0005%	0.0048%	0.0000%	0.0000%
962.0	Poisoning by adrenal cortical steroids	0.0005%	0.0048%	0.0000%	0.0000%
926.19	Other crushing injury of trunk	0.0005%	0.0000%	0.0021%	0.0000%
965.00	Poisoning by opium (alkaloids), unspecified	0.0005%	0.0000%	0.0000%	0.0032%
874.9	Open wound to other and unspecified parts of neck, compli	0.0009%	0.0000%	0.0000%	0.0000%
965.01	Poisoning by heroin	0.0000%	0.0000%	0.0041%	0.0000%
878.3	Open wound of scrotum and testes, complicated, including	0.0009%	0.0000%	0.0000%	0.0000%
965.09	Poisoning by other opiates and related narcotics	0.0005%	0.0000%	0.0000%	0.0032%
946.3	Burns of multiple specified sites, full-thickness skin loss (tl	0.0009%	0.0000%	0.0000%	0.0000%
966.3	Poisoning by other and unspecified anticonvulsants	0.0000%	0.0000%	0.0000%	0.0064%
944.26	Burn of back of hand, blisters, epidermal loss (second degr	0.0009%	0.0000%	0.0000%	0.0000%
969.0	Poisoning by antidepressants	0.0009%	0.0000%	0.0000%	0.0000%
996.93	Complications of reattached finger(s)	0.0005%	0.0000%	0.0021%	0.0000%
923.10	Contusion of forearm	0.0009%	0.0000%	0.0000%	0.0000%

ICD-9-CM	ICD-9-CM	PureO	ContiB	NewB	PastB
945.09	Burn of multiple sites of lower limb(s), unspecified degree	0.0009%	0.0000%	0.0000%	0.0000%
969.7	Poisoning by psychostimulants	0.0005%	0.0000%	0.0021%	0.0000%
944.36	Burn of back of hand, full-thickness skin loss (third degree	0.0009%	0.0000%	0.0000%	0.0000%
977.9	Poisoning by unspecified drug or medicinal substance	0.0000%	0.0048%	0.0021%	0.0000%
902.20	Injury to celiac and mesenteric arteries, unspecified	0.0009%	0.0000%	0.0000%	0.0000%
980.9	Toxic effect of unspecified alcohol	0.0005%	0.0048%	0.0000%	0.0000%
939.2	Foreign body in vulva and vagina	0.0009%	0.0000%	0.0000%	0.0000%
923.20	Contusion of hand(s)	0.0009%	0.0000%	0.0000%	0.0000%
951.1	Injury to trochlear nerve	0.0009%	0.0000%	0.0000%	0.0000%
943.49	Burn of multiple sites of upper limb, except wrist and hand	0.0005%	0.0000%	0.0021%	0.0000%
911.0	Superficial injury of trunk, abrasion or friction burn withou	0.0009%	0.0000%	0.0000%	0.0000%
923.9	Contusion of unspecified part of upper limb	0.0005%	0.0000%	0.0021%	0.0000%
V53.01	Fitting and adjustment of cerebral ventricular (communicat	0.0005%	0.0000%	0.0021%	0.0000%
902.21	Injury to gastric artery	0.0005%	0.0000%	0.0021%	0.0000%
V53.39	Fitting and adjustment of other cardiac device	0.0005%	0.0000%	0.0000%	0.0032%
944.00	Burn of unspecified site of hand(s), unspecified degree	0.0009%	0.0000%	0.0000%	0.0000%
V55.6	Attention to other artificial opening of urinary tract	0.0005%	0.0048%	0.0000%	0.0000%
994.8	Electrocution and nonfatal effects of electric current	0.0009%	0.0000%	0.0000%	0.0000%
941.21	Burn of ear (any part), blisters, epidermal loss (second deg	0.0009%	0.0000%	0.0000%	0.0000%
945.43	Burn of ankle, deep necrosis of underlying tissues (deep thi	0.0009%	0.0000%	0.0000%	0.0000%
902.26	Injury to primary branches of superior mesenteric artery	0.0009%	0.0000%	0.0000%	0.0000%
996.31	Mechanical complication due to urethral (indwelling) cathe	0.0005%	0.0000%	0.0000%	0.0032%
836.62	Posterior dislocation of tibia, proximal end, open	0.0000%	0.0000%	0.0041%	0.0000%
872.9	Open wound to unspecified part of ear, complicated	0.0009%	0.0000%	0.0000%	0.0000%
838.04	Closed dislocation, metatarsal (bone), joint, unspecified	0.0005%	0.0000%	0.0021%	0.0000%
802.23	Fracture of mandible, closed, coronoid process	0.0000%	0.0000%	0.0041%	0.0000%
814.09	Other fracture of carpal bone(s), closed	0.0009%	0.0000%	0.0000%	0.0000%
806.01	C1- C4 level fracture with complete lesion of cord, closed	0.0000%	0.0048%	0.0021%	0.0000%
864.14	Major laceration to liver, with open wound into cavity	0.0009%	0.0000%	0.0000%	0.0000%
863.49	Injury to other site of colon or rectum,without mention of c	0.0009%	0.0000%	0.0000%	0.0000%
873.70	Open wound to unspecified site of mouth, complicated	0.0009%	0.0000%	0.0000%	0.0000%
868.01	Injury to adrenal gland without mention of open wound int	0.0005%	0.0000%	0.0021%	0.0000%
852.09	Subarachnoid hemorrhage following injury without mentio	0.0005%	0.0048%	0.0000%	0.0000%
868.02	Injury to bile duct and gallbladder without mention of open	0.0000%	0.0000%	0.0041%	0.0000%
840.9	Sprains and strains of unspecified site of shoulder and uppe	0.0009%	0.0000%	0.0000%	0.0000%
852.45	Extradural hemorrhage following injury without mention o	0.0009%	0.0000%	0.0000%	0.0000%
852.10	Subarachnoid hemorrhage following injury with mention o	0.0000%	0.0000%	0.0041%	0.0000%
868.09	Injury to other and multiple intra-abdominal organs withou	0.0009%	0.0000%	0.0000%	0.0000%
838.01	Closed dislocation, tarsal (bone), joint, unspecified	0.0005%	0.0000%	0.0021%	0.0000%

ICD-9-CM	ICD-9-CM	PureO	ContiB	NewB	PastB
839.00	Dislocations of cervical vertebra, unspecified, closed	0.0005%	0.0000%	0.0000%	0.0032%
810.10	Fracture in unspecified part of clavicle, open	0.0009%	0.0000%	0.0000%	0.0000%
810.13	Fracture in acromial end of clavicle, open	0.0009%	0.0000%	0.0000%	0.0000%
821.10	Fracture of unspecified part of femur, open	0.0005%	0.0000%	0.0021%	0.0000%
868.14	Injury to retroperitoneum with open wound into cavity	0.0009%	0.0000%	0.0000%	0.0000%
820.30	Unspecified fracture of trochanteric section of femur, open	0.0000%	0.0000%	0.0041%	0.0000%
870.1	Laceration of eyelid,full thickness,not involving lacrimal pa	0.0009%	0.0000%	0.0000%	0.0000%
802.37	Fracture of mandible, open, alveolar border of body	0.0009%	0.0000%	0.0000%	0.0000%
863.51	Injury to ascending (right) colon,with open wound into cav	0.0005%	0.0000%	0.0021%	0.0000%
863.41	Injury to ascending (right) colon,without mention of open w	0.0000%	0.0048%	0.0021%	0.0000%
814.03	Fracture of triquetral (cuneiform) bone of wrist, closed	0.0009%	0.0000%	0.0000%	0.0000%
836.52	Posterior dislocation of tibia, proximal end, closed	0.0005%	0.0000%	0.0021%	0.0000%
802.39	Fracture of mandible, open, multiple sites	0.0009%	0.0000%	0.0000%	0.0000%
811.11	Fracture in acromial process of scapula, open	0.0009%	0.0000%	0.0000%	0.0000%
815.11	Fracture of base of thumb (first) metacarpal, open	0.0009%	0.0000%	0.0000%	0.0000%
812.30	Fracture of unspecified part of humerus, open	0.0009%	0.0000%	0.0000%	0.0000%
806.02	C1- C4 level fracture with anterior cord syndrome, closed	0.0005%	0.0000%	0.0000%	0.0032%
852.40	Extradural hemorrhage following injury without mention of	0.0000%	0.0000%	0.0041%	0.0000%
820.9	Fracture of unspecified part of neck of femur, open	0.0005%	0.0000%	0.0000%	0.0032%
873.60	Open wound to unspecified site of mouth, without mention	0.0005%	0.0000%	0.0021%	0.0000%
863.54	Injury to sigmoid colon,with open wound into cavity	0.0009%	0.0000%	0.0000%	0.0000%
831.02	Closed posterior dislocation of humerus	0.0009%	0.0000%	0.0000%	0.0000%
813.15	Head of radius fractures, open	0.0009%	0.0000%	0.0000%	0.0000%
865.10	Unspecified injury to spleen, with open wound into cavity	0.0005%	0.0000%	0.0021%	0.0000%
872.10	Open wound to unspecified site of external ear, complicate	0.0009%	0.0000%	0.0000%	0.0000%
873.72	Open wound of gum (alveolar process), complicated	0.0009%	0.0000%	0.0000%	0.0000%
862.9	Injury to multiple and unspecified intrathoracic organs,with	0.0009%	0.0000%	0.0000%	0.0000%
840.0	Sprains and strains of acromioclavicular (joint) (ligament)	0.0009%	0.0000%	0.0000%	0.0000%
808.1	Fracture of acetabulum, open	0.0009%	0.0000%	0.0000%	0.0000%
861.30	Unspecified injury to lung,with open wound into thorax	0.0005%	0.0000%	0.0021%	0.0000%
801.15	Fracture of base of skull, closed with cerebral laceration an	0.0009%	0.0000%	0.0000%	0.0000%
745.0	Common truncus	0.0009%	0.0000%	0.0000%	0.0000%
757.0	Hereditary edema of legs	0.0009%	0.0000%	0.0000%	0.0000%
757.39	Other specified anomalies of skin	0.0005%	0.0000%	0.0021%	0.0000%
800.76	Fracture of vault of skull, open with subarachnoid, subdura	0.0005%	0.0048%	0.0000%	0.0000%
744.1	Accessory auricle	0.0009%	0.0000%	0.0000%	0.0000%
801.33	Fracture of base of skull, closed with other and unspecified	0.0009%	0.0000%	0.0000%	0.0000%
786.03	Apnea	0.0005%	0.0000%	0.0021%	0.0000%
746.5	Congenital mitral stenosis	0.0009%	0.0000%	0.0000%	0.0000%

ICD-9-CM	ICD-9-CM	PureO	ContiB	NewB	PastB
786.05	Shortness of breath	0.0005%	0.0000%	0.0000%	0.0032%
781.3	Lack of coordination	0.0000%	0.0097%	0.0000%	0.0000%
750.10	Anomaly of tongue, unspecified	0.0009%	0.0000%	0.0000%	0.0000%
801.05	Fracture of base of skull, closed without mention of intracr	0.0009%	0.0000%	0.0000%	0.0000%
742.9	Unspecified anomalies of brain, spinal cord and nervous sy	0.0005%	0.0048%	0.0000%	0.0000%
755.60	Unspecified anomaly of lower limb	0.0009%	0.0000%	0.0000%	0.0000%
801.56	Fracture of base of skull, open without mention of intracrar	0.0009%	0.0000%	0.0000%	0.0000%
756.89	Other specified anomalies of muscle, tendon, fascia, and cc	0.0009%	0.0000%	0.0000%	0.0000%
738.10	Unspecified acquired deformity of head	0.0005%	0.0000%	0.0021%	0.0000%
800.10	Fracture of vault of skull, closed with cerebral laceration ar	0.0000%	0.0000%	0.0041%	0.0000%
750.26	Other specified anomalies of mouth	0.0009%	0.0000%	0.0000%	0.0000%
732.0	Juvenile osteochondrosis of spine	0.0009%	0.0000%	0.0000%	0.0000%
765.18	Other preterm immaturity,2000-2499g	0.0009%	0.0000%	0.0000%	0.0000%
800.32	Fracture of vault of skull, closed with other and unspecified	0.0000%	0.0000%	0.0041%	0.0000%
767.0	Subdural and cerebral hemorrhage	0.0009%	0.0000%	0.0000%	0.0000%
800.61	Fracture of vault of skull, open with cerebral laceration and	0.0009%	0.0000%	0.0000%	0.0000%
801.46	Fracture of base of skull, closed with intracranial injury of	0.0005%	0.0000%	0.0021%	0.0000%
746.81	Subaortic stenosis	0.0009%	0.0000%	0.0000%	0.0000%
767.3	Other injuries to skeleton	0.0009%	0.0000%	0.0000%	0.0000%
801.13	Fracture of base of skull, closed with cerebral laceration an	0.0000%	0.0000%	0.0041%	0.0000%
730.19	Chronic osteomyelitis, multiple sites	0.0005%	0.0048%	0.0000%	0.0000%
782.7	Spontaneous ecchymoses	0.0009%	0.0000%	0.0000%	0.0000%
741.03	Spina bifida with hydrocephalus, lumbar region	0.0009%	0.0000%	0.0000%	0.0000%
745.19	Other transposition of great vessels	0.0009%	0.0000%	0.0000%	0.0000%
788.31	Urge incontinence	0.0009%	0.0000%	0.0000%	0.0000%
756.79	Other congenital anomalies of abdominal wall	0.0009%	0.0000%	0.0000%	0.0000%
733.44	Aseptic necrosis of talus	0.0005%	0.0000%	0.0021%	0.0000%
800.03	Fracture of vault of skull, closed without mention of intracr	0.0005%	0.0000%	0.0021%	0.0000%
733.21	Solitary bone cyst	0.0009%	0.0000%	0.0000%	0.0000%
744.83	Macrostomia	0.0005%	0.0000%	0.0021%	0.0000%
730.01	Acute osteomyelitis, shoulder region	0.0005%	0.0000%	0.0000%	0.0032%
728.2	Muscular wasting and disuse atrophy, not elsewhere classif	0.0005%	0.0000%	0.0021%	0.0000%
741.92	Spina bifida without mention of hydrocephalus, dorsal (tho	0.0000%	0.0048%	0.0000%	0.0032%
754.35	Congenital dislocation of one hip with subluxation of other	0.0009%	0.0000%	0.0000%	0.0000%
774.4	Perinatal jaundice due to hepatocellular damage	0.0009%	0.0000%	0.0000%	0.0000%
800.25	Fracture of vault of skull, closed with subarchnoid, subdura	0.0009%	0.0000%	0.0000%	0.0000%
777.8	Other specified perinatal disorders of digestive system	0.0009%	0.0000%	0.0000%	0.0000%
736.76	Other calcaneus deformity	0.0009%	0.0000%	0.0000%	0.0000%
747.49	Other anomalies of great veins	0.0005%	0.0000%	0.0000%	0.0032%

ICD-9-CM	ICD-9-CM	PureO	ContiB	NewB	PastB
781.1	Disturbances of sensation of smell and taste	0.0009%	0.0000%	0.0000%	0.0000%
730.20	Unspecified osteomyelitis, unspecified site	0.0009%	0.0000%	0.0000%	0.0000%
800.56	Fracture of vault of skull, open without mention of intracra	0.0005%	0.0000%	0.0021%	0.0000%
789.33	Abdominal or pelvic swelling, mass, or lump, right lower q	0.0009%	0.0000%	0.0000%	0.0000%
800.70	Fracture of vault of skull, open with subarachnoid, subdura	0.0005%	0.0048%	0.0000%	0.0000%
780.02	Transient alteration of awareness	0.0005%	0.0000%	0.0000%	0.0032%
800.81	Fracture of vault of skull, open with other and unspecified	0.0005%	0.0000%	0.0021%	0.0000%
790.2	Abnormal glucose tolerance test	0.0005%	0.0000%	0.0021%	0.0000%
782.1	Rash and other nonspecific skin eruption	0.0009%	0.0000%	0.0000%	0.0000%
730.03	Acute osteomyelitis, forearm	0.0009%	0.0000%	0.0000%	0.0000%
782.5	Cyanosis	0.0000%	0.0000%	0.0041%	0.0000%
728.13	Postoperative heterotopic calcification	0.0005%	0.0048%	0.0000%	0.0000%
801.14	Fracture of base of skull, closed with cerebral laceration an	0.0009%	0.0000%	0.0000%	0.0000%
729.39	Panniculitis, other site	0.0005%	0.0000%	0.0021%	0.0000%
748.3	Other anomalies of larynx, trachea, and bronchus	0.0009%	0.0000%	0.0000%	0.0000%
737.34	Thoracogenic scoliosis	0.0009%	0.0000%	0.0000%	0.0000%
745.12	Corrected transposition of great vessels	0.0000%	0.0000%	0.0000%	0.0064%
795.79	Other and unspecified nonspecific immunological findings	0.0005%	0.0000%	0.0021%	0.0000%
785.1	Palpitations	0.0005%	0.0000%	0.0021%	0.0000%
796.0	Nonspecific abnormal toxicological findings	0.0005%	0.0000%	0.0021%	0.0000%
755.10	Syndactyly of multiple and unspecified sites	0.0009%	0.0000%	0.0000%	0.0000%
799.0	Asphyxia	0.0005%	0.0000%	0.0000%	0.0032%
750.0	Tongue tie	0.0009%	0.0000%	0.0000%	0.0000%
799.1	Respiratory arrest	0.0005%	0.0000%	0.0000%	0.0032%
747.21	Anomalies of aortic arch	0.0009%	0.0000%	0.0000%	0.0000%
801.51	Fracture of base of skull, open without mention of intracra	0.0009%	0.0000%	0.0000%	0.0000%
787.99	Other symptoms involoing digestive system	0.0009%	0.0000%	0.0000%	0.0000%
801.71	Fracture of base of skull, open with subarachnoid, subdural	0.0005%	0.0000%	0.0021%	0.0000%
646.62	Infections of genitourinary tract in pregnancy, delivered, w	0.0009%	0.0000%	0.0000%	0.0000%
694.9	Unspecified bullous dermatoses	0.0005%	0.0000%	0.0021%	0.0000%
715.91	Osteoarthrosis, unspecified whether generalized or localize	0.0005%	0.0000%	0.0021%	0.0000%
665.44	High vaginal laceration, postpartum condition or complicat	0.0009%	0.0000%	0.0000%	0.0000%
719.97	Unspecified disorder of joint, ankle and foot	0.0009%	0.0000%	0.0000%	0.0000%
647.01	Syphilis in the mother classifiable elsewhere, but complica	0.0009%	0.0000%	0.0000%	0.0000%
718.23	Pathological dislocation, forearm	0.0005%	0.0048%	0.0000%	0.0000%
656.21	Isoimmunization from other and unspecified blood-group i	0.0009%	0.0000%	0.0000%	0.0000%
654.14	Tumors of body of uterus, postpartum condition or complic	0.0009%	0.0000%	0.0000%	0.0000%
681.01	Felon	0.0009%	0.0000%	0.0000%	0.0000%
653.61	Hydrocephalic fetus causing disproportion, delivered, with	0.0009%	0.0000%	0.0000%	0.0000%

ICD-9-CM	ICD-9-CM	PureO	ContiB	NewB	PastB
671.82	Other venous complications in pregnancy and the puerperiu	0.0009%	0.0000%	0.0000%	0.0000%
653.71	Other fetal abnormality causing disproportion, delivered, w	0.0009%	0.0000%	0.0000%	0.0000%
654.31	Retroverted and incarcerated gravid uterus, delivered, with	0.0009%	0.0000%	0.0000%	0.0000%
716.14	Traumatic arthropathy, hand	0.0000%	0.0000%	0.0041%	0.0000%
681.02	Onychia and paronychia of finger	0.0005%	0.0000%	0.0000%	0.0032%
691.8	Other atopic dermatitis and related conditions	0.0005%	0.0048%	0.0000%	0.0000%
707.9	Chronic ulcer of unspecified site of skin	0.0005%	0.0000%	0.0000%	0.0032%
718.32	Recurrent dislocation of joint, upper arm	0.0005%	0.0000%	0.0021%	0.0000%
651.21	Quadruplet pregnancy, delivered, with or without mention	0.0009%	0.0000%	0.0000%	0.0000%
663.81	Other umbilical cord complications, delivered, with or with	0.0009%	0.0000%	0.0000%	0.0000%
647.63	Other viral diseases in the mother classifiable elsewhere, bu	0.0005%	0.0000%	0.0000%	0.0032%
664.01	First-degree perineal laceration, delivered, with or without	0.0005%	0.0000%	0.0000%	0.0032%
666.32	Postpartum coagulation defects, delivered, with mention of	0.0005%	0.0000%	0.0021%	0.0000%
719.93	Unspecified disorder of joint, forearm	0.0009%	0.0000%	0.0000%	0.0000%
718.94	Unspecified derangement of joint, hand	0.0009%	0.0000%	0.0000%	0.0000%
695.9	Unspecified erythematous condition	0.0009%	0.0000%	0.0000%	0.0000%
722.81	Postlaminectomy syndrome, cervical region	0.0005%	0.0048%	0.0000%	0.0000%
718.48	Contracture of joint, other specified sites	0.0005%	0.0000%	0.0021%	0.0000%
717.85	Old disruption of other ligaments of knee	0.0009%	0.0000%	0.0000%	0.0000%
696.2	Parapsoriasis	0.0009%	0.0000%	0.0000%	0.0000%
702.8	Other specified dermatoses	0.0009%	0.0000%	0.0000%	0.0000%
671.31	Deep phlebothrombosis, antepartum, delivered, with or wit	0.0009%	0.0000%	0.0000%	0.0000%
664.34	Fourth-degree perineal laceration, postpartum condition or	0.0009%	0.0000%	0.0000%	0.0000%
715.21	Osteoarthrosis, localized, secondary, shoulder region	0.0005%	0.0000%	0.0021%	0.0000%
719.12	Hemarthrosis, upper arm	0.0005%	0.0000%	0.0000%	0.0032%
705.81	Dyshidrosis	0.0005%	0.0048%	0.0000%	0.0000%
715.15	Osteoarthrosis, localized, primary, pelvic region and thigh	0.0000%	0.0000%	0.0021%	0.0032%
716.99	Arthropathy, unspecified, multiple sites	0.0005%	0.0000%	0.0021%	0.0000%
719.22	Villonodular synovitis, upper arm	0.0009%	0.0000%	0.0000%	0.0000%
711.04	Pyogenic arthritis, hand	0.0009%	0.0000%	0.0000%	0.0000%
719.23	Villonodular synovitis, forearm	0.0005%	0.0000%	0.0021%	0.0000%
703.8	Other specified diseases of nail	0.0009%	0.0000%	0.0000%	0.0000%
716.89	Other specified arthropathy, multiple sites	0.0005%	0.0000%	0.0021%	0.0000%
718.38	Recurrent dislocation of joint, other specified sites	0.0005%	0.0000%	0.0000%	0.0032%
646.64	Infections of genitourinary tract in pregnancy, postpartum c	0.0009%	0.0000%	0.0000%	0.0000%
665.91	Unspecified obstetrical trauma, delivered, with or without r	0.0009%	0.0000%	0.0000%	0.0000%
674.24	Disruption of perineal wound, postpartum condition or com	0.0009%	0.0000%	0.0000%	0.0000%
646.41	Peripheral neuritis in pregnancy, delivered, with or without	0.0009%	0.0000%	0.0000%	0.0000%
716.90	Arthropathy, unspecified, unspecified site	0.0005%	0.0000%	0.0021%	0.0000%

ICD-9-CM	ICD-9-CM	PureO	ContiB	NewB	PastB
726.73	Calcaneal spur	0.0005%	0.0048%	0.0000%	0.0000%
723.9	Unsepcified musculoskeletal disorders and symptoms refer	0.0009%	0.0000%	0.0000%	0.0000%
719.96	Unspecified disorder of joint, lower leg	0.0005%	0.0000%	0.0000%	0.0032%
719.43	Pain in joint, forearm	0.0005%	0.0000%	0.0021%	0.0000%
701.5	Other abnormal granulation tissue	0.0005%	0.0000%	0.0021%	0.0000%
665.01	Rupture of uterus before onset of labor, delivered, with or	0.0009%	0.0000%	0.0000%	0.0000%
646.51	Asymptomatic bacteriuria in pregnancy, delivered, with or	0.0005%	0.0000%	0.0000%	0.0032%
655.21	Hereditary disease in family possibly affection fetus, affect	0.0009%	0.0000%	0.0000%	0.0000%
720.89	Other inflammatory spondylopathies	0.0009%	0.0000%	0.0000%	0.0000%
652.51	High head at term, delivered, with or without mention of a	0.0009%	0.0000%	0.0000%	0.0000%
718.51	Ankylosis of joint, shoulder region	0.0005%	0.0048%	0.0000%	0.0000%
686.00	Pyoderma, unspecified	0.0000%	0.0000%	0.0000%	0.0064%
715.98	Osteoarthrosis, unspecified whether generalized or localize	0.0005%	0.0048%	0.0000%	0.0000%
719.47	Pain in joint, ankle and foot	0.0005%	0.0048%	0.0000%	0.0000%
716.12	Traumatic arthropathy, upper arm	0.0005%	0.0048%	0.0000%	0.0000%
711.93	Unspecified infective arthritis, forearm	0.0005%	0.0000%	0.0000%	0.0032%
701.1	Keratoderma, acquired	0.0005%	0.0048%	0.0000%	0.0000%
648.94	Other current conditions classifiable elsewhere in the moth	0.0009%	0.0000%	0.0000%	0.0000%
709.09	Other dyschromia	0.0009%	0.0000%	0.0000%	0.0000%
715.38	Osteoarthrosis, localized, not specified whether primary or	0.0000%	0.0000%	0.0021%	0.0032%
655.03	Central nervous system malformation in fetus, affecting ma	0.0009%	0.0000%	0.0000%	0.0000%
708.8	Other specified urticaria	0.0000%	0.0048%	0.0000%	0.0032%
634.11	Abortion, complicated by delayed or excessive hemorrhage	0.0009%	0.0000%	0.0000%	0.0000%
628.3	Infertility, female, of uterine origin	0.0009%	0.0000%	0.0000%	0.0000%
637.92	Unspecified abortion, without mention of complication, co	0.0009%	0.0000%	0.0000%	0.0000%
526.9	Unspecified disease of the jaws	0.0009%	0.0000%	0.0000%	0.0000%
537.6	Hourglass stricture or stenosis of stomach	0.0005%	0.0000%	0.0021%	0.0000%
571.40	Chronic hepatitis, unspecified	0.0005%	0.0000%	0.0021%	0.0000%
552.02	Femoral hernia with obstruction, bilateral (not specified as	0.0005%	0.0000%	0.0000%	0.0032%
618.7	Old laceration of muscles of pelvic floor	0.0009%	0.0000%	0.0000%	0.0000%
588.0	Renal osteodystrophy	0.0009%	0.0000%	0.0000%	0.0000%
552.29	Other ventral hernia with obstruction	0.0009%	0.0000%	0.0000%	0.0000%
531.70	Gastric ulcer, chronic without mention of hemorrhage or p	0.0005%	0.0000%	0.0021%	0.0000%
619.2	Genital tract-skin fistula, female	0.0005%	0.0000%	0.0021%	0.0000%
526.5	Alveolitis of jaw	0.0009%	0.0000%	0.0000%	0.0000%
602.8	Other specified disorders of prostate	0.0005%	0.0000%	0.0000%	0.0032%
634.71	Abortion, with other specified complications, incomplete	0.0009%	0.0000%	0.0000%	0.0000%
602.9	Unspecified disorder of prostate	0.0009%	0.0000%	0.0000%	0.0000%
532.30	Duodenal ulcer, acute without mention of hemorrhage or p	0.0009%	0.0000%	0.0000%	0.0000%

ICD-9-CM	ICD-9-CM	PureO	ContiB	NewB	PastB
621.4	Hematometra	0.0005%	0.0048%	0.0000%	0.0000%
598.8	Other specified causes of urethral stricture	0.0009%	0.0000%	0.0000%	0.0000%
551.21	Incisional ventral hernia with gangrene	0.0005%	0.0000%	0.0000%	0.0032%
532.41	Duodenal ulcer, chronic or unspecified with hemorrhage, w	0.0005%	0.0000%	0.0000%	0.0032%
622.0	Erosion and ectropion of cervix	0.0005%	0.0000%	0.0021%	0.0000%
564.9	Unspecified functional disorder of intestine	0.0009%	0.0000%	0.0000%	0.0000%
527.0	Atrophy of salivary glands	0.0009%	0.0000%	0.0000%	0.0000%
581.3	Nephritic syndrome, with lesion of minimal change glomer	0.0005%	0.0048%	0.0000%	0.0000%
577.9	Unspecified disease of pancreas	0.0009%	0.0000%	0.0000%	0.0000%
555.1	Regional enteritis, large intestine	0.0009%	0.0000%	0.0000%	0.0000%
606.8	Infertity due to extratesticular causes	0.0009%	0.0000%	0.0000%	0.0000%
596.2	Vesical fistula, not elsewhere classified	0.0005%	0.0000%	0.0021%	0.0000%
623.2	Stricture or atresia of vagina	0.0009%	0.0000%	0.0000%	0.0000%
634.12	Abortion, complicated by delayed or excessive hemorrhage	0.0009%	0.0000%	0.0000%	0.0000%
593.73	Vesicoureteral reflux , with reflux nephropathy NOS	0.0009%	0.0000%	0.0000%	0.0000%
584.8	Acute renal failure, with other specified pathological lesion	0.0009%	0.0000%	0.0000%	0.0000%
526.3	Central giant cell (reparative) granuloma	0.0005%	0.0000%	0.0021%	0.0000%
550.01	Inguinal hernia, with gangrene, unilateral or unspecified, re	0.0005%	0.0000%	0.0000%	0.0032%
625.0	Dyspareunia	0.0009%	0.0000%	0.0000%	0.0000%
637.90	Unspecified abortion, without mention of complication, uns	0.0005%	0.0000%	0.0000%	0.0032%
593.82	Ureteral fistula	0.0009%	0.0000%	0.0000%	0.0000%
598.2	Postoperative urethral stricture	0.0009%	0.0000%	0.0000%	0.0000%
607.3	Priapism	0.0009%	0.0000%	0.0000%	0.0000%
640.93	Unspecified hemorrhage in early pregnancy, antepartum co	0.0005%	0.0000%	0.0021%	0.0000%
579.3	Other and unspecified postsurgical nonabsorption	0.0000%	0.0000%	0.0021%	0.0032%
641.31	Antepartum hemorrhage associated with coagulation defect	0.0009%	0.0000%	0.0000%	0.0000%
551.1	Umbilical hernia with gangrene	0.0009%	0.0000%	0.0000%	0.0000%
575.5	Fistula of gallbladder	0.0009%	0.0000%	0.0000%	0.0000%
532.51	Duodenal ulcer, chronic or unspecified with perforation, wi	0.0005%	0.0000%	0.0021%	0.0000%
536.49	Other gastrostomy complication	0.0005%	0.0000%	0.0000%	0.0032%
521.4	Pathological resorption	0.0000%	0.0048%	0.0021%	0.0000%
395.2	Rheumatic aortic stenosis with insufficiency	0.0005%	0.0048%	0.0000%	0.0000%
442.82	Aneurysm of subclavian artery	0.0009%	0.0000%	0.0000%	0.0000%
437.9	Ill-defined cerebrovascular disease, unspecified	0.0005%	0.0000%	0.0000%	0.0032%
377.41	Ischemic optic neuropathy	0.0000%	0.0000%	0.0041%	0.0000%
447.9	Unspecified disorders of arteries and arterioles	0.0000%	0.0048%	0.0021%	0.0000%
478.9	Other and unspecified diseases of upper respiratory tract	0.0000%	0.0048%	0.0021%	0.0000%
451.19	Phlebitis and thrombophlebitis of other deep vessels of low	0.0009%	0.0000%	0.0000%	0.0000%
426.53	Other bilateral bundle branch block	0.0009%	0.0000%	0.0000%	0.0000%

ICD-9-CM	ICD-9-CM	PureO	ContiB	NewB	PastB
380.23	Other chronic otitis externa	0.0009%	0.0000%	0.0000%	0.0000%
384.82	Atrophic nonflaccid tympanic membrane	0.0000%	0.0048%	0.0021%	0.0000%
453.2	Embolism and thrombosis of vena cava	0.0009%	0.0000%	0.0000%	0.0000%
519.4	Disorders of diaphragm	0.0000%	0.0000%	0.0041%	0.0000%
385.89	Other disorders of middle ear and mastoid	0.0000%	0.0000%	0.0000%	0.0064%
378.9	Unspecified disorder of eye movements	0.0005%	0.0048%	0.0000%	0.0000%
385.9	Unspecified disorder of middle ear and mastoid	0.0009%	0.0000%	0.0000%	0.0000%
389.2	Mixed conductive and sensorineural hearing loss	0.0005%	0.0000%	0.0000%	0.0032%
368.8	Other specified visual disturbances	0.0009%	0.0000%	0.0000%	0.0000%
383.81	Postauricular fistula	0.0009%	0.0000%	0.0000%	0.0000%
455.9	Residual hemorrhoidal skin tags	0.0005%	0.0000%	0.0000%	0.0032%
426.6	Other heart block	0.0005%	0.0000%	0.0000%	0.0032%
363.70	Choroidal detachment, unspecified	0.0009%	0.0000%	0.0000%	0.0000%
371.42	Recurrent erosion of cornea	0.0009%	0.0000%	0.0000%	0.0000%
375.53	Stenosis of lacrimal canaliculi	0.0005%	0.0000%	0.0021%	0.0000%
516.0	Pulmonary alveolar proteinosis	0.0005%	0.0000%	0.0021%	0.0000%
374.20	Lagophthalmos, unspecified	0.0005%	0.0048%	0.0000%	0.0000%
437.7	Transient global amnesia	0.0005%	0.0048%	0.0000%	0.0000%
380.51	Acquired stenosis of external ear canal, secondary to traum	0.0009%	0.0000%	0.0000%	0.0000%
433.00	Occlusion and stenosis of basilar artery without mention of	0.0000%	0.0048%	0.0000%	0.0032%
380.89	Other disorders of external ear	0.0009%	0.0000%	0.0000%	0.0000%
522.7	Periapical abscess with sinus	0.0009%	0.0000%	0.0000%	0.0000%
386.2	Vertigo of central origin	0.0000%	0.0048%	0.0000%	0.0032%
374.89	Other disorders of eyelid	0.0009%	0.0000%	0.0000%	0.0000%
386.30	Labyrinthitis, unspecified	0.0005%	0.0000%	0.0000%	0.0032%
378.30	Heterotropia, unspecified	0.0009%	0.0000%	0.0000%	0.0000%
461.1	Acute sinusitis of frontal	0.0005%	0.0000%	0.0000%	0.0032%
482.32	Pneumonia due to Streptococcus, Group B	0.0000%	0.0000%	0.0000%	0.0064%
365.04	Ocular hypertension	0.0005%	0.0000%	0.0021%	0.0000%
372.40	Pterygium, unspecified	0.0009%	0.0000%	0.0000%	0.0000%
410.72	Acute subendocardial infarction, subsequent episode of car	0.0009%	0.0000%	0.0000%	0.0000%
493.20	Chronic obstructive asthma (with obstructive pulmonary di	0.0005%	0.0048%	0.0000%	0.0000%
410.81	Acute myocardial infarction of other specified sites, initial	0.0009%	0.0000%	0.0000%	0.0000%
426.54	Trifascicular block	0.0009%	0.0000%	0.0000%	0.0000%
410.90	Acute myocardial infarction of unspecified site, episode of	0.0000%	0.0000%	0.0041%	0.0000%
365.62	Glaucoma associated with ocular inflammations	0.0005%	0.0000%	0.0000%	0.0032%
387.8	Other otosclerosis	0.0005%	0.0000%	0.0021%	0.0000%
362.81	Retinal hemorrhage	0.0005%	0.0000%	0.0021%	0.0000%
379.19	Other disorders of sclera	0.0009%	0.0000%	0.0000%	0.0000%

ICD-9-CM	ICD-9-CM	PureO	ContiB	NewB	PastB
384.21	Central perforation of tympanic membrane	0.0009%	0.0000%	0.0000%	0.0000%
370.60	Corneal neovascularization, unspecified	0.0009%	0.0000%	0.0000%	0.0000%
375.42	Chronic dacryocystitis	0.0009%	0.0000%	0.0000%	0.0000%
370.62	Pannus (corneal)	0.0009%	0.0000%	0.0000%	0.0000%
365.65	Glaucoma associated with ocular trauma	0.0005%	0.0048%	0.0000%	0.0000%
474.01	Chronic adenoiditis	0.0009%	0.0000%	0.0000%	0.0000%
446.21	Goodpasture's syndrome	0.0009%	0.0000%	0.0000%	0.0000%
414.11	Aneurysm of coronary vessels	0.0009%	0.0000%	0.0000%	0.0000%
363.61	Choroidal hemorrhage, unspecified	0.0005%	0.0000%	0.0000%	0.0032%
372.61	Granuloma of conjunctiva	0.0005%	0.0000%	0.0000%	0.0032%
432.9	Unspecified intracranial hemorrhage	0.0005%	0.0048%	0.0000%	0.0000%
381.81	Dysfunction of Eustachian tube	0.0005%	0.0000%	0.0021%	0.0000%
446.7	Takayasu's disease	0.0009%	0.0000%	0.0000%	0.0000%
437.1	Other generalized ischemic cerebrovascular disease	0.0005%	0.0000%	0.0021%	0.0000%
521.8	Other specified diseases of hard tissues of teeth	0.0005%	0.0000%	0.0000%	0.0032%
420.99	Other and unspecified acute pericarditis	0.0005%	0.0000%	0.0021%	0.0000%
365.51	Phacolytic glaucoma	0.0000%	0.0000%	0.0021%	0.0032%
374.87	Dermatochalasis	0.0009%	0.0000%	0.0000%	0.0000%
274.10	Gouty nephropathy, unspecified	0.0009%	0.0000%	0.0000%	0.0000%
351.9	Facial nerve disorder, unspecified	0.0000%	0.0000%	0.0021%	0.0032%
214.4	Lipoma of spermatic cord	0.0009%	0.0000%	0.0000%	0.0000%
296.50	Bipolar affective disorder, depressed, unspecified	0.0000%	0.0000%	0.0000%	0.0064%
292.9	Unspecified drug-induced mental disorder	0.0000%	0.0000%	0.0000%	0.0064%
297.9	Unspecified paranoid state	0.0000%	0.0048%	0.0000%	0.0032%
250.63	Diabetes with neurological manifestations, Type I [insulin	0.0005%	0.0000%	0.0000%	0.0032%
300.00	Anxiety state, unspecified	0.0000%	0.0048%	0.0021%	0.0000%
288.1	Functional disorders of polymorphonuclear neutrophils	0.0005%	0.0000%	0.0021%	0.0000%
300.81	Somatization disorder	0.0000%	0.0048%	0.0021%	0.0000%
291.2	Other alcoholic dementia	0.0005%	0.0000%	0.0000%	0.0032%
300.82	Undifferentiated somatoform disorder	0.0005%	0.0000%	0.0021%	0.0000%
236.4	Neoplasm of uncertain behavior of testis	0.0009%	0.0000%	0.0000%	0.0000%
302.70	Psychosexual dysfunction, unspecified	0.0009%	0.0000%	0.0000%	0.0000%
235.0	Neoplasm of uncertain behavior of major salivary glands	0.0009%	0.0000%	0.0000%	0.0000%
302.72	Psychosexual dysfunction with inhibited sexual excitement	0.0009%	0.0000%	0.0000%	0.0000%
210.8	Benign neoplasm of hypopharynx	0.0009%	0.0000%	0.0000%	0.0000%
307.1	Anorexia nervosa	0.0000%	0.0097%	0.0000%	0.0000%
250.30	Diabetes with other coma, Type II [non-insulin dependent	0.0005%	0.0048%	0.0000%	0.0000%
242.91	Thyrotoxicosis without mention of goiter or other cause, w	0.0005%	0.0048%	0.0000%	0.0000%
289.4	Hypersplenism	0.0005%	0.0000%	0.0021%	0.0000%

ICD-9-CM	ICD-9-CM	PureO	ContiB	NewB	PastB
307.89	Other psychalgia	0.0009%	0.0000%	0.0000%	0.0000%
353.2	Cervical root lesions, not elsewhere classified	0.0005%	0.0000%	0.0021%	0.0000%
316	Psychic factors associated with diseases classified elsewher	0.0000%	0.0000%	0.0041%	0.0000%
355.2	Lesion of femoral nerve	0.0005%	0.0000%	0.0021%	0.0000%
320.2	Streptococcal meningitis	0.0000%	0.0048%	0.0021%	0.0000%
250.43	Diabetes with renal manifestations, Type I [insulin depende	0.0000%	0.0048%	0.0021%	0.0000%
232.7	Carcinoma in situ of skin of lower limb, including hip	0.0009%	0.0000%	0.0000%	0.0000%
250.53	Diabetes with ophthalmic manifestations, Type I [insulin de	0.0005%	0.0048%	0.0000%	0.0000%
322.0	Nonpyogenic meningitis	0.0009%	0.0000%	0.0000%	0.0000%
360.19	Other endophthalmitis	0.0009%	0.0000%	0.0000%	0.0000%
237.2	Neoplasm of uncertain behavior of adrenal gland	0.0005%	0.0000%	0.0021%	0.0000%
275.0	Disorders of iron metabolism	0.0005%	0.0048%	0.0000%	0.0000%
222.2	Benign neoplasm of prostate	0.0005%	0.0000%	0.0000%	0.0032%
362.50	Macular degeneration (senile), unspecified	0.0005%	0.0000%	0.0000%	0.0032%
331.89	Other cerebral degeneration	0.0005%	0.0000%	0.0000%	0.0032%
345.80	Other forms of epilepsy without mention of intractable epil	0.0000%	0.0097%	0.0000%	0.0000%
253.1	Other and unspecified anterior pituitary hyperfunction	0.0000%	0.0000%	0.0041%	0.0000%
346.00	Classical migraine，without mention of intractable migrain	0.0005%	0.0048%	0.0000%	0.0000%
281.1	Other vitamin B12 deficiency anemia	0.0005%	0.0000%	0.0000%	0.0032%
346.80	Other forms of migraine，without mention of intractable m	0.0000%	0.0000%	0.0021%	0.0032%
333.7	Symptomatic torsion dystonia	0.0000%	0.0000%	0.0021%	0.0032%
259.3	Ectopic hormone secretion, not elsewhere classified	0.0009%	0.0000%	0.0000%	0.0000%
281.2	Folate-deficiency anemia	0.0005%	0.0000%	0.0000%	0.0032%
208.01	Acute leukemia of unspecified cell type, in remission	0.0005%	0.0048%	0.0000%	0.0000%
253.5	Diabetes insipidus	0.0000%	0.0000%	0.0021%	0.0032%
290.40	Arteriosclerotic dementia uncomplicated	0.0000%	0.0000%	0.0000%	0.0064%
333.90	Unspecified extrapyramidal diseases and abnormal movem	0.0000%	0.0048%	0.0021%	0.0000%
353.4	Lumbosacral root lesions, not elsewhere classified	0.0000%	0.0097%	0.0000%	0.0000%
233.2	Carcinoma in situ of other and unspecified parts of uterus	0.0005%	0.0000%	0.0021%	0.0000%
354.5	Mononeuritis multiplex	0.0000%	0.0048%	0.0000%	0.0032%
282.9	Hereditary hemolytic anemia, unspecified	0.0009%	0.0000%	0.0000%	0.0000%
292.81	Drug-induced delirium	0.0005%	0.0048%	0.0000%	0.0000%
216.9	Benign neoplasm of skin, site unspecified	0.0005%	0.0000%	0.0000%	0.0032%
230.7	Carcinoma in situ of other and unspecified parts of intestin	0.0009%	0.0000%	0.0000%	0.0000%
222.4	Benign neoplasm of scrotum	0.0009%	0.0000%	0.0000%	0.0000%
229.0	Benign neoplasm of lymph nodes	0.0009%	0.0000%	0.0000%	0.0000%
342.90	Hemiplegia, unspecified, affecting unspecified site	0.0005%	0.0048%	0.0000%	0.0000%
359.8	Other myopathies	0.0005%	0.0048%	0.0000%	0.0000%
222.8	Benign neoplasm of other specified sites of male genital or	0.0005%	0.0000%	0.0021%	0.0000%

ICD-9-CM	ICD-9-CM	PureO	ContiB	NewB	PastB
295.40	Acute schizophrenic episode, unspecified	0.0000%	0.0048%	0.0021%	0.0000%
207.00	Acute erythremia and erythroleukemia, without mention of	0.0000%	0.0048%	0.0021%	0.0000%
360.02	Panophthalmitis	0.0005%	0.0000%	0.0000%	0.0032%
344.00	Quadriplegia, unspecified	0.0000%	0.0000%	0.0041%	0.0000%
360.50	Foreign body, magnetic, intraocular, unspecified	0.0009%	0.0000%	0.0000%	0.0000%
344.40	Monoplegia of upper limb affecting unspecified side	0.0009%	0.0000%	0.0000%	0.0000%
296.22	Major depressive disorder, single episode, moderate	0.0000%	0.0000%	0.0000%	0.0064%
344.61	Cauda equina syndrome with neurogenic bladder	0.0005%	0.0000%	0.0021%	0.0000%
296.32	Major depressive disorder, recurrent episode, moderate	0.0000%	0.0000%	0.0000%	0.0064%
344.9	Paralysis, unspecified	0.0005%	0.0000%	0.0021%	0.0000%
286.6	Defibrination syndrome	0.0009%	0.0000%	0.0000%	0.0000%
083.0	Q-fever	0.0005%	0.0000%	0.0000%	0.0032%
184.1	Malignant neoplasm of labia majora	0.0000%	0.0097%	0.0000%	0.0000%
066.1	Tick-borne fever	0.0005%	0.0000%	0.0021%	0.0000%
003.8	Other specified salmonella infections	0.0000%	0.0000%	0.0041%	0.0000%
202.03	Nodular lymphoma, intra-abdominal lymph nodes	0.0005%	0.0000%	0.0000%	0.0032%
100.9	Leptospirosis,unspecified	0.0009%	0.0000%	0.0000%	0.0000%
072.9	Mumps without mention of complication	0.0009%	0.0000%	0.0000%	0.0000%
013.46	Tuberculoma of spinal cord, tubercle bacilli not found by b	0.0005%	0.0048%	0.0000%	0.0000%
015.86	Other specified joint tuberculosis, tubercle bacilli not found	0.0000%	0.0000%	0.0021%	0.0032%
015.76	Other specified bone tuberculosis, tubercle bacilli not found	0.0009%	0.0000%	0.0000%	0.0000%
186.0	Malignant neoplasm of undescended testis	0.0009%	0.0000%	0.0000%	0.0000%
190.0	Malignant neoplasm of eyeball, except conjunctiva, cornea,	0.0009%	0.0000%	0.0000%	0.0000%
094.2	Syphilitic meningitis	0.0000%	0.0000%	0.0041%	0.0000%
164.3	Malignant neoplasm of posterior mediastinum	0.0005%	0.0000%	0.0021%	0.0000%
015.94	Tuberculosis of unspecified bones and joints, tubercle bacil	0.0009%	0.0000%	0.0000%	0.0000%
190.2	Malignant neoplasm of lacrimal gland	0.0005%	0.0048%	0.0000%	0.0000%
198.81	Secondary malignant neoplasm of breast	0.0000%	0.0000%	0.0021%	0.0032%
025	Melioidosis	0.0005%	0.0000%	0.0021%	0.0000%
088.81	Lyme disease	0.0000%	0.0048%	0.0021%	0.0000%
136.2	Specific infections by free-living amebae	0.0009%	0.0000%	0.0000%	0.0000%
094.0	Tabes dorsalis	0.0000%	0.0000%	0.0021%	0.0032%
018.96	Miliary tuberculosis, unspecified, tubercle bacilli not found	0.0005%	0.0000%	0.0021%	0.0000%
160.8	Malignant neoplasm of nasal cavities, middle ear, and acce	0.0005%	0.0000%	0.0000%	0.0032%
017.22	Tuberculosis of peripheral lymph nodes, bacteriological or	0.0009%	0.0000%	0.0000%	0.0000%
201.92	Hodgkin's disease, unspecified, intrathoracic lymph node	0.0009%	0.0000%	0.0000%	0.0000%
191.7	Malignant neoplasm of brain stem	0.0005%	0.0000%	0.0021%	0.0000%
015.83	Other specified joint tuberculosis, tubercle bacilli found (in	0.0000%	0.0048%	0.0000%	0.0032%
053.8	Herpes zoster with unspecified complication	0.0009%	0.0000%	0.0000%	0.0000%

ICD-9-CM	ICD-9-CM	PureO	ContiB	NewB	PastB
078.3	Cat-scratch disease	0.0005%	0.0000%	0.0021%	0.0000%
034.0	Streptococcal sore throat	0.0009%	0.0000%	0.0000%	0.0000%
015.72	Other specified bone tuberculosis, bacteriological or histolo	0.0005%	0.0000%	0.0021%	0.0000%
018.92	Miliary tuberculosis, unspecified, bacteriological or histolo	0.0000%	0.0048%	0.0000%	0.0032%
161.8	Malignant neoplasm of other specified sites of larynx	0.0009%	0.0000%	0.0000%	0.0000%
140.3	Malignant neoplasm of upper lip, inner aspect	0.0005%	0.0000%	0.0021%	0.0000%
015.02	Vertebral column tuberculosis, bacteriological or histologic	0.0005%	0.0000%	0.0021%	0.0000%
192.3	Malignant neoplasm of spinal meninges	0.0005%	0.0000%	0.0021%	0.0000%
160.5	Malignant neoplasm of sphenoidal sinus	0.0005%	0.0048%	0.0000%	0.0000%
014.03	Tuberculous peritonitis, tubercle bacilli found (in sputum) b	0.0009%	0.0000%	0.0000%	0.0000%
200.01	Reticulosarcoma, lymph nodes of head, face and neck	0.0009%	0.0000%	0.0000%	0.0000%
054.2	Herpetic gingivostomatitis	0.0009%	0.0000%	0.0000%	0.0000%
200.11	Lymphosarcoma, lymph nodes of head, face and neck	0.0009%	0.0000%	0.0000%	0.0000%
012.15	Tuberculosis of intrathoracic lymph nodes, tubercle bacilli	0.0009%	0.0000%	0.0000%	0.0000%
074.0	Herpangina	0.0009%	0.0000%	0.0000%	0.0000%
015.80	Other specified joint tuberculosis, unspecified	0.0000%	0.0000%	0.0021%	0.0032%
200.23	Burkitt's tumor or lymphoma, intra-abdominal lymph nodes	0.0000%	0.0000%	0.0021%	0.0032%
008.69	Enteritis due to other viral enteritis	0.0009%	0.0000%	0.0000%	0.0000%
200.88	Other named variants lymphoma, lymph nodes of multiple	0.0000%	0.0048%	0.0000%	0.0032%
195.5	Malignant neoplasm of other and ill-defined sites of lower	0.0005%	0.0000%	0.0000%	0.0032%
039.2	Abdominal diseases due to actinomycotic infections	0.0009%	0.0000%	0.0000%	0.0000%
160.1	Malignant neoplasm of auditory tube, middle ear and masto	0.0009%	0.0000%	0.0000%	0.0000%
201.68	Mixed cellularity, lymph nodes of multiple sites	0.0005%	0.0000%	0.0021%	0.0000%
031.2	Disseminated diseases due to other mycobacteria	0.0009%	0.0000%	0.0000%	0.0000%
012.00	Tuberculous pleurisy, unspecified	0.0005%	0.0048%	0.0000%	0.0000%
017.24	Tuberculosis of peripheral lymph nodes, tubercle bacilli no	0.0005%	0.0048%	0.0000%	0.0000%
171.8	Malignant neoplasm of other specified sites of connective a	0.0005%	0.0000%	0.0021%	0.0000%
157.4	Malignant neoplasm of islets of langerhans	0.0005%	0.0000%	0.0021%	0.0000%
133.0	Scabies	0.0000%	0.0048%	0.0000%	0.0032%
196.8	Secondary and unspecified malignant neoplasm of lymph n	0.0009%	0.0000%	0.0000%	0.0000%
202.15	Mycosis fungoides, lymph nodes of inguinal region and low	0.0000%	0.0000%	0.0041%	0.0000%
035	Erysipelas	0.0005%	0.0000%	0.0021%	0.0000%
011.26	Tuberculosis of lung with cavitation, tubercle bacilli not fo	0.0009%	0.0000%	0.0000%	0.0000%
037	Tetanus	0.0000%	0.0097%	0.0000%	0.0000%
011.33	Tuberculosis of bronchus, tubercle bacilli found (in sputum	0.0000%	0.0000%	0.0021%	0.0032%
176.9	Kaposi's sarcoma of unspecified	0.0005%	0.0000%	0.0021%	0.0000%
202.84	Other lymphomas, lymph nodes of axilla and upper limb	0.0005%	0.0000%	0.0021%	0.0000%
164.1	Malignant neoplasm of heart	0.0009%	0.0000%	0.0000%	0.0000%
008.47	Intestinal infections due to other Gram-negative bacteria	0.0009%	0.0000%	0.0000%	0.0000%

ICD-9-CM	ICD-9-CM	PureO	ContiB	NewB	PastB
112.0	Candidiasis of mouth	0.0005%	0.0000%	0.0000%	0.0032%
204.90	Unspecified lymphoid leukemia, without mention of remiss	0.0009%	0.0000%	0.0000%	0.0000%
054.79	Herpes simplex with other specified complications	0.0005%	0.0000%	0.0000%	0.0032%
015.23	Knee tuberculosis, tubercle bacilli found (in sprtum) by mi	0.0000%	0.0000%	0.0041%	0.0000%
148.2	Malignant neoplasm of aryepiglottic fold, hypopharyngeal	0.0005%	0.0000%	0.0021%	0.0000%
018.95	Miliary tuberculosis, unspecified, tubercle bacilli not found	0.0005%	0.0048%	0.0000%	0.0000%
944.42	Burn of thumb (nail), deep necrosis of underlying tissues (	0.0000%	0.0000%	0.0021%	0.0000%
998.51	Infected postoperative seroma	0.0000%	0.0000%	0.0021%	0.0000%
997.1	Cardiac complications	0.0005%	0.0000%	0.0000%	0.0000%
980.1	Toxic effect of methyl alcohol	0.0000%	0.0000%	0.0021%	0.0000%
951.0	Injury to oculomotor nerve	0.0005%	0.0000%	0.0000%	0.0000%
902.87	Injury to multiple blood vessels of abdomen and pelvis	0.0005%	0.0000%	0.0000%	0.0000%
V58.81	Fitting and adjustment of vascular catheter	0.0005%	0.0000%	0.0000%	0.0000%
983.0	Toxic effect of corrosive aromatics	0.0005%	0.0000%	0.0000%	0.0000%
902.10	Injury to inferior vena cava, unspecified	0.0000%	0.0000%	0.0021%	0.0000%
923.11	Contusion of elbow	0.0005%	0.0000%	0.0000%	0.0000%
939.0	Foreign body in bladder and urethra	0.0005%	0.0000%	0.0000%	0.0000%
943.42	Burn of elbow, deep necrosis of underlying tissues (deep th	0.0000%	0.0000%	0.0021%	0.0000%
906.7	Late effect of burn of other extremities	0.0005%	0.0000%	0.0000%	0.0000%
983.9	Toxic effect of caustic, unspecified	0.0005%	0.0000%	0.0000%	0.0000%
V56.8	Encounter for other dialysis	0.0000%	0.0048%	0.0000%	0.0000%
984.9	Toxic effect of unspecified lead compound	0.0005%	0.0000%	0.0000%	0.0000%
V67.2	Follow-up examination of following chemotherapy	0.0000%	0.0000%	0.0000%	0.0032%
985.1	Toxic effect of arsenic and its compounds	0.0000%	0.0000%	0.0021%	0.0000%
942.21	Burn of trunk, breast, blisters, epidermal loss (second degr	0.0005%	0.0000%	0.0000%	0.0000%
956.1	Injury to femoral nerve	0.0000%	0.0000%	0.0021%	0.0000%
934.9	Foreign body in respiratory tree, unspecified	0.0000%	0.0000%	0.0000%	0.0032%
987.8	Toxic effect of other specified gases, fumes, or vapors	0.0005%	0.0000%	0.0000%	0.0000%
900.02	Injury to external carotid artery	0.0000%	0.0000%	0.0021%	0.0000%
952.19	T7-T12 level with other specified spinal cord injury	0.0000%	0.0000%	0.0021%	0.0000%
965.7	Poisoning by other non-narcotic analgesics	0.0005%	0.0000%	0.0000%	0.0000%
988.2	Toxic effect of berries and other plants	0.0000%	0.0000%	0.0000%	0.0032%
952.13	T1-T6 level with central cord syndrome	0.0000%	0.0000%	0.0021%	0.0000%
988.8	Toxic effect of other specified noxious substances eaten as	0.0005%	0.0000%	0.0000%	0.0000%
940.9	Unspecified burn of eye and adnexa	0.0005%	0.0000%	0.0000%	0.0000%
989.0	Toxic effect of hydrocyanic acid and cyanides	0.0005%	0.0000%	0.0000%	0.0000%
952.16	T7-T12 level with complete lesion of spinal cord	0.0000%	0.0000%	0.0021%	0.0000%
945.40	Burn of unspecified site of lower limb (leg), deep necrosis	0.0000%	0.0048%	0.0000%	0.0000%
900.1	Injury to internal jugular vein	0.0000%	0.0000%	0.0021%	0.0000%

ICD-9-CM	ICD-9-CM	PureO	ContiB	NewB	PastB
943.11	Burn of forearm, erythema (first degree)	0.0005%	0.0000%	0.0000%	0.0000%
943.02	Burn of elbow, unspecified degree	0.0005%	0.0000%	0.0000%	0.0000%
945.41	Burn of toe(s) (nail), deep necrosis of underlying tissues (d	0.0005%	0.0000%	0.0000%	0.0000%
902.59	Other injury to iliac blood vessels	0.0000%	0.0000%	0.0000%	0.0032%
904.54	Injury to posterior tibial vein	0.0000%	0.0000%	0.0000%	0.0032%
942.20	Burn of trunk, unspecified site, blisters, epidermal loss (sec	0.0005%	0.0000%	0.0000%	0.0000%
989.89	Toxic effect of other chiefly nonmedicinal as to source	0.0000%	0.0000%	0.0021%	0.0000%
963.1	Poisoning by antineoplastic and immunosuppressive drugs	0.0000%	0.0000%	0.0000%	0.0032%
989.9	Toxic effect of unspecified substance, chiefly nonmedicinal	0.0005%	0.0000%	0.0000%	0.0000%
910.1	Superficial injury of face, neck and scalp except eye, abras	0.0005%	0.0000%	0.0000%	0.0000%
902.24	Injury to other specified branches of celiac axis	0.0000%	0.0048%	0.0000%	0.0000%
923.03	Contusion of upper arm	0.0005%	0.0000%	0.0000%	0.0000%
991.1	Frostbite of hand	0.0005%	0.0000%	0.0000%	0.0000%
900.00	Injury to unspecified part of carotid artery	0.0005%	0.0000%	0.0000%	0.0000%
991.3	Frostbite of other and unspecified sites	0.0005%	0.0000%	0.0000%	0.0000%
998.9	Unspecified complication of procedure, not elsewhere class	0.0000%	0.0000%	0.0021%	0.0000%
992.1	Heat syncope	0.0000%	0.0000%	0.0021%	0.0000%
D25.9	#N/A	0.0005%	0.0000%	0.0000%	0.0000%
993.1	Barotrauma, sinus	0.0000%	0.0000%	0.0021%	0.0000%
939.3	Foreign body in penis	0.0000%	0.0000%	0.0021%	0.0000%
956.9	Injury to unspecified nerve of pelvic girdle and lower limb	0.0005%	0.0000%	0.0000%	0.0000%
905.8	Late effect of tendon injury	0.0000%	0.0048%	0.0000%	0.0000%
994.7	Sphyxiation and strangulation	0.0005%	0.0000%	0.0000%	0.0000%
902.23	Injury to splenic artery	0.0005%	0.0000%	0.0000%	0.0000%
952.4	Injury of cauda equina without evidence of spinal bone inju	0.0000%	0.0000%	0.0021%	0.0000%
874.4	Open wound of pharynx, without mention of complication	0.0000%	0.0000%	0.0021%	0.0000%
887.6	Traumatic amputation of arm and hand (complete) (partial)	0.0000%	0.0000%	0.0021%	0.0000%
V53.6	Fitting and adjustment of urinary devices	0.0000%	0.0048%	0.0000%	0.0000%
944.01	Burn of single digit (finger nail) other than thumb, unspeci	0.0005%	0.0000%	0.0000%	0.0000%
941.10	Burn of face and head, unspecified site, erythema (first deg	0.0000%	0.0000%	0.0021%	0.0000%
958.1	Fat embolism	0.0005%	0.0000%	0.0000%	0.0000%
V55.8	Attention to other specified artificial opening	0.0005%	0.0000%	0.0000%	0.0000%
995.3	Allergy, unspecified	0.0000%	0.0000%	0.0021%	0.0000%
972.9	Poisoning by other and unspecified agents primarily affecti	0.0000%	0.0048%	0.0000%	0.0000%
944.03	Burn of two or more digits, not including thumb, unspecifi	0.0005%	0.0000%	0.0000%	0.0000%
V58.41	Encounter for planned postoperative wound closure	0.0005%	0.0000%	0.0000%	0.0000%
995.86	Malignant hyperthermia	0.0005%	0.0000%	0.0000%	0.0000%
V58.89	Other specified aftercare	0.0005%	0.0000%	0.0000%	0.0000%
880.20	Open wound of shoulder region, with tendon involvement	0.0005%	0.0000%	0.0000%	0.0000%

ICD-9-CM	ICD-9-CM	PureO	ContiB	NewB	PastB
879.3	Open wound of abdominal wall, anterior, complicated	0.0005%	0.0000%	0.0000%	0.0000%
880.10	Open wound of shoulder region, complicated	0.0005%	0.0000%	0.0000%	0.0000%
944.56	Burn of back of hand, deep necrosis of underlying tissues (	0.0005%	0.0000%	0.0000%	0.0000%
944.05	Burn of palm, unspecified degree	0.0000%	0.0000%	0.0021%	0.0000%
929.9	Crushing injury of unspecified site	0.0005%	0.0000%	0.0000%	0.0000%
952.07	C5-C7 level with anterior cord syndrome	0.0005%	0.0000%	0.0000%	0.0000%
930.0	Corneal foreign body	0.0005%	0.0000%	0.0000%	0.0000%
924.3	Contusion of toe	0.0000%	0.0000%	0.0000%	0.0032%
879.7	Open wound to other and unspecified parts of trunk, compl	0.0005%	0.0000%	0.0000%	0.0000%
903.02	Injury to axillary vein	0.0005%	0.0000%	0.0000%	0.0000%
997.60	Late amputation stump unspecified complication	0.0000%	0.0000%	0.0000%	0.0032%
901.89	Other injury to blood vessels of thorax	0.0000%	0.0000%	0.0021%	0.0000%
897.1	Traumatic amputation, unilateral, below knee, complicated	0.0005%	0.0000%	0.0000%	0.0000%
924.8	Contusion of multiple sites of lower limb, NEC	0.0000%	0.0000%	0.0000%	0.0032%
942.42	Burn of chest wall, excluding breast and nipple, deep necro	0.0005%	0.0000%	0.0000%	0.0000%
944.07	Burn of wrist, unspecified degree	0.0005%	0.0000%	0.0000%	0.0000%
904.8	Injury to unspecified blood vessel of lower extremity	0.0005%	0.0000%	0.0000%	0.0000%
925.2	Crushing injury of neck	0.0000%	0.0048%	0.0000%	0.0000%
904.50	Injury to tibial vessel(s), unspecified	0.0005%	0.0000%	0.0000%	0.0000%
902.52	Injury to hypogastric vein	0.0005%	0.0000%	0.0000%	0.0000%
943.22	Burn of elbow, blisters, epidermal loss (second degree)	0.0000%	0.0000%	0.0021%	0.0000%
926.11	Crushing injury of back	0.0005%	0.0000%	0.0000%	0.0000%
897.6	Traumatic amputation of leg(s) (complete) (partial), bilatera	0.0000%	0.0000%	0.0021%	0.0000%
953.2	Injury to lumbar root	0.0000%	0.0000%	0.0021%	0.0000%
949.3	Burn, unspecified, full-thickness skin loss (third degree NO	0.0005%	0.0000%	0.0000%	0.0000%
880.13	Open wound of upper arm, complicated	0.0005%	0.0000%	0.0000%	0.0000%
879.5	Open wound of abdominal wall, lateral, complciated	0.0005%	0.0000%	0.0000%	0.0000%
887.1	Traumatic amputation, unilateral, below elbow, complicate	0.0000%	0.0000%	0.0021%	0.0000%
945.06	Burn of thigh (any part), unspecified degree	0.0005%	0.0000%	0.0000%	0.0000%
879.6	Open wound to other and unspecified parts of trunk, withou	0.0005%	0.0000%	0.0000%	0.0000%
999.5	Other serum reaction	0.0000%	0.0048%	0.0000%	0.0000%
944.10	Burn of unspecified site, hand, erythema (first degree)	0.0005%	0.0000%	0.0000%	0.0000%
905.2	Late effect of fracture of upper extremities	0.0005%	0.0000%	0.0000%	0.0000%
902.31	Injury to superior mesenteric vein and primary subdivisions	0.0005%	0.0000%	0.0000%	0.0000%
945.21	Burn of toe(s) (nail), blisters, epidermal loss (second degre	0.0005%	0.0000%	0.0000%	0.0000%
876.1	Open wound of back, complicated	0.0005%	0.0000%	0.0000%	0.0000%
940.1	Other burns of eyelids and periocular area	0.0005%	0.0000%	0.0000%	0.0000%
874.5	Open wound of pharynx, complicated	0.0000%	0.0000%	0.0021%	0.0000%
V31.00	Twin, mate liveborn, born in hospital, delivered without me	0.0005%	0.0000%	0.0000%	0.0000%

ICD-9-CM	ICD-9-CM	PureO	ContiB	NewB	PastB
879.9	Open wound(s) (multiple) of unspecified site(s), complicate	0.0005%	0.0000%	0.0000%	0.0000%
V50.42	Prophylactic ovary removal	0.0000%	0.0000%	0.0000%	0.0032%
953.8	Injury to nerve roots and spinal plexus, multiple sites	0.0005%	0.0000%	0.0000%	0.0000%
940.3	Acid chemical burn of cornea and conjunctival sac	0.0005%	0.0000%	0.0000%	0.0000%
880.19	Open wound to multiple sites of shoulder and upper arm, c	0.0005%	0.0000%	0.0000%	0.0000%
906.4	Late effect of crushing	0.0000%	0.0048%	0.0000%	0.0000%
922.9	Contusion of unspecified part of trunk	0.0005%	0.0000%	0.0000%	0.0000%
V53.02	Fitting and adjustment of neuropacemaker (brain)(periphera	0.0005%	0.0000%	0.0000%	0.0000%
944.22	Burn of thumb (nail), blister, epidermal loss (second degree	0.0005%	0.0000%	0.0000%	0.0000%
917.6	Superficial injury of foot and toe(s), superficial foreign boc	0.0005%	0.0000%	0.0000%	0.0000%
944.23	Burn of two or more digits, not including thumb, blisters, e	0.0005%	0.0000%	0.0000%	0.0000%
902.22	Injury to hepatic artery	0.0005%	0.0000%	0.0000%	0.0000%
878.4	Open wound of vulva, without mention of complication, in	0.0005%	0.0000%	0.0000%	0.0000%
944.40	Burn of unspecified site of hand(s), deep necrosis of underl	0.0005%	0.0000%	0.0000%	0.0000%
928.01	Crushing injury of hip	0.0000%	0.0048%	0.0000%	0.0000%
911.1	Superficial injury of trunk, abrasion or friction burn, infecte	0.0005%	0.0000%	0.0000%	0.0000%
953.9	Injury to unspecified site of nerve roots and spinal plexus	0.0000%	0.0000%	0.0021%	0.0000%
941.19	Burn of multiple sites (except with eye) of face, head and n	0.0005%	0.0000%	0.0000%	0.0000%
996.75	Other complications due to nervous system device, implant	0.0005%	0.0000%	0.0000%	0.0000%
969.8	Poisoning by other specified psychotropic agents	0.0000%	0.0048%	0.0000%	0.0000%
955.0	Injury to axillary nerve	0.0005%	0.0000%	0.0000%	0.0000%
901.82	Injury to internal mammary artery or vein	0.0005%	0.0000%	0.0000%	0.0000%
996.77	Other complications due to internal joint prosthesis	0.0005%	0.0000%	0.0000%	0.0000%
943.41	Burn of forearm, deep necrosis of underlying tissues (deep	0.0000%	0.0000%	0.0021%	0.0000%
946.4	Burns of multiple specified sites, deep necrosis of underlyi	0.0000%	0.0000%	0.0021%	0.0000%
V57.9	Unspecified rehabilitation procedures	0.0000%	0.0000%	0.0000%	0.0032%
959.8	Injury to other specified sites, including multiple	0.0000%	0.0000%	0.0000%	0.0032%
912.0	Superficial injury of shoulder and upper arm, abrasion or fr	0.0005%	0.0000%	0.0000%	0.0000%
903.4	Injury to palmar artery	0.0005%	0.0000%	0.0000%	0.0000%
941.25	Burn of nose (septum), blisters, epidermal loss (second deg	0.0005%	0.0000%	0.0000%	0.0000%
941.49	Burn of multiple sites (except with eye) of face, head and n	0.0005%	0.0000%	0.0000%	0.0000%
V58.83	Encounter for therapeutic drug monitoring	0.0000%	0.0000%	0.0000%	0.0032%
961.8	Poisoning by other antimycobacterial drugs	0.0000%	0.0048%	0.0000%	0.0000%
944.45	Burn of palm, deep necrosis of underlying tissues (deep thi	0.0005%	0.0000%	0.0000%	0.0000%
942.40	Burn of trunk, unspecified site, deep necrosis of underlying	0.0005%	0.0000%	0.0000%	0.0000%
943.09	Burn of multiple sites of upper limb, except wrist and hand	0.0000%	0.0000%	0.0021%	0.0000%
942.04	Burn of back (any part), unspecified degree	0.0005%	0.0000%	0.0000%	0.0000%
944.53	Burn of two or more digits, not including thumb, deep necr	0.0000%	0.0000%	0.0021%	0.0000%
952.11	T1-T6 level with complete lesion of spinal cord	0.0005%	0.0000%	0.0000%	0.0000%

ICD-9-CM	ICD-9-CM	PureO	ContiB	NewB	PastB
V67.59	Follow-up examination of following other treatment	0.0005%	0.0000%	0.0000%	0.0000%
996.94	Complications of reattached upper extremity, other and uns	0.0000%	0.0000%	0.0021%	0.0000%
975.2	Poisoning by skeletal muscle relaxants	0.0000%	0.0000%	0.0000%	0.0032%
997.01	Central nervous system complication	0.0000%	0.0048%	0.0000%	0.0000%
997.02	Iatrogenic cerebrovascular infarction or hemorrhage	0.0005%	0.0000%	0.0000%	0.0000%
943.53	Burn of upper arm, deep necrosis of underlying tissues (dee	0.0000%	0.0048%	0.0000%	0.0000%
845.10	Sprains and strains of foot,unspecified site	0.0005%	0.0000%	0.0000%	0.0000%
852.59	Extradural hemorrhage following injury with mention of op	0.0005%	0.0000%	0.0000%	0.0000%
836.60	Dislocation of knee, unspecified, open	0.0005%	0.0000%	0.0000%	0.0000%
825.34	Fracture of cuneiform of foot, open	0.0005%	0.0000%	0.0000%	0.0000%
833.12	Open dislocation of radiocarpal (joint)	0.0005%	0.0000%	0.0000%	0.0000%
839.21	Dislocations of thoracic vertebra, closed	0.0005%	0.0000%	0.0000%	0.0000%
846.8	Sprains and strains of other specified sites of sacroiliac reg	0.0005%	0.0000%	0.0000%	0.0000%
812.59	Other fracture of lower end of humerus,open	0.0000%	0.0000%	0.0000%	0.0032%
803.06	Other and unqualified skull fracture, closed without mentio	0.0000%	0.0000%	0.0021%	0.0000%
806.36	T7-t12 level fracture with complete lesion of cord, open	0.0005%	0.0000%	0.0000%	0.0000%
845.02	Sprains and strains of calcaneofibular (ligament)	0.0005%	0.0000%	0.0000%	0.0000%
803.21	Other and unqualified skull fracture, closed with subarachn	0.0005%	0.0000%	0.0000%	0.0000%
823.11	Fracture of upper end of fibula alone, open	0.0005%	0.0000%	0.0000%	0.0000%
853.09	Other and unspecified intracranial hemorrhage following in	0.0005%	0.0000%	0.0000%	0.0000%
814.05	Fracture of trapezium bone (larger multangular), closed	0.0000%	0.0000%	0.0021%	0.0000%
814.12	Fracture of lunate (semilunar) bone of wrist, open	0.0005%	0.0000%	0.0000%	0.0000%
847.2	Sprains and strains of lumbar	0.0000%	0.0000%	0.0021%	0.0000%
830.0	Closed dislocation of jaw	0.0005%	0.0000%	0.0000%	0.0000%
852.34	Subdural hemorrhage following injury with mention of ope	0.0005%	0.0000%	0.0000%	0.0000%
864.09	Other injury to liver, without mention of open wound into c	0.0005%	0.0000%	0.0000%	0.0000%
863.55	Injury to rectum,with open wound into cavity	0.0000%	0.0000%	0.0021%	0.0000%
864.10	Unspecified injury to liver, with open wound into cavity	0.0000%	0.0000%	0.0000%	0.0032%
874.00	Open wound of larynx with trachea, without mention of co	0.0005%	0.0000%	0.0000%	0.0000%
864.11	Hematoma and contusion to liver, with open wound into ca	0.0005%	0.0000%	0.0000%	0.0000%
812.13	Fracture of greater tuberosity of humerus, open	0.0000%	0.0000%	0.0000%	0.0032%
864.12	Minor laceration to liver, with open wound into cavity	0.0000%	0.0000%	0.0021%	0.0000%
842.11	Sprains and strains of carpometacarpal (joint)	0.0000%	0.0000%	0.0000%	0.0032%
853.14	Other and unspecified intracranial hemorrhage following in	0.0005%	0.0000%	0.0000%	0.0000%
838.12	Open dislocation, midtarsal(joint)	0.0000%	0.0000%	0.0021%	0.0000%
854.00	Other and unspecified intracranial injury without mention c	0.0005%	0.0000%	0.0000%	0.0000%
862.39	Injury to other specified intrathoracic organs,without menti	0.0000%	0.0000%	0.0021%	0.0000%
806.69	frracture of sacrum and coccyx with other spinal cord injur	0.0000%	0.0000%	0.0021%	0.0000%
812.19	Fracture in other part of upper end of humerus, open	0.0005%	0.0000%	0.0000%	0.0000%

ICD-9-CM	ICD-9-CM	PureO	ContiB	NewB	PastB
805.7	Fracture of sacrum and coccyx, open	0.0000%	0.0048%	0.0000%	0.0000%
814.06	Fracture of trapezoid bone (smaller multangular), closed	0.0005%	0.0000%	0.0000%	0.0000%
803.24	Other and unqualified skull fracture, closed with subarachn	0.0000%	0.0000%	0.0021%	0.0000%
819.0	Multiple fractures involving both upper limbs, and upper li	0.0000%	0.0000%	0.0000%	0.0032%
854.05	Other and unspecified intracranial injury without mention o	0.0005%	0.0000%	0.0000%	0.0000%
852.31	Subdural hemorrhage following injury with mention of ope	0.0005%	0.0000%	0.0000%	0.0000%
831.11	Open anterior dislocation of humerus	0.0005%	0.0000%	0.0000%	0.0000%
801.80	Fracture of base of skull, open with other and unspecified i	0.0005%	0.0000%	0.0000%	0.0000%
802.32	Fracture of mandible, open, subcondylar	0.0005%	0.0000%	0.0000%	0.0000%
850.3	Concussion with prolonged loss of consciousness and retur	0.0005%	0.0000%	0.0000%	0.0000%
851.83	Other and unspecified cerebral laceration and contusion wi	0.0000%	0.0000%	0.0000%	0.0032%
873.69	Open wound to other and multiple sites of mouth, without	0.0005%	0.0000%	0.0000%	0.0000%
865.11	Hematoma without rupture of capsule in spleen, with open	0.0005%	0.0000%	0.0000%	0.0000%
801.86	Fracture of base of skull, open with other and unspecified i	0.0000%	0.0000%	0.0021%	0.0000%
865.14	Massive parenchyma disruption in spleen, with open wound	0.0000%	0.0000%	0.0021%	0.0000%
813.51	Colles' fracture, open	0.0005%	0.0000%	0.0000%	0.0000%
865.19	Other injury to spleen, with open wound into cavity	0.0005%	0.0000%	0.0000%	0.0000%
833.05	Closed dislocation of metacarpal (bone) of proximal end of	0.0005%	0.0000%	0.0000%	0.0000%
854.11	Other and unspecified intracranial injury with mention of o	0.0000%	0.0000%	0.0021%	0.0000%
833.10	Open dislocation of unspecified part of wrist	0.0000%	0.0000%	0.0021%	0.0000%
813.80	Unspecified fracture of forearm, closed	0.0005%	0.0000%	0.0000%	0.0000%
838.02	Closed dislocation, midtarsal(joint)	0.0000%	0.0000%	0.0021%	0.0000%
832.04	Closed lateral dislocation of elbow	0.0005%	0.0000%	0.0000%	0.0000%
834.11	Open dislocation of metacarpophalangeal (joint)	0.0005%	0.0000%	0.0000%	0.0000%
839.40	Dislocations of vertebra, unspecified site, closed	0.0000%	0.0000%	0.0021%	0.0000%
808.59	Fracture of other specified part of pelvis, open	0.0005%	0.0000%	0.0000%	0.0000%
866.12	Laceration to kidney, with open wound into cavity	0.0005%	0.0000%	0.0000%	0.0000%
872.02	Open wound of auditory canal, without mention of complic	0.0005%	0.0000%	0.0000%	0.0000%
842.02	Sprains and strains of radiocarpal (joint) (ligament)	0.0005%	0.0000%	0.0000%	0.0000%
872.11	Open wound to auricle of ear, complicated	0.0005%	0.0000%	0.0000%	0.0000%
867.1	Injury to bladder and urethra, with open wound into cavity	0.0005%	0.0000%	0.0000%	0.0000%
872.69	Open wound to other and multiple sites of ear, without me	0.0005%	0.0000%	0.0000%	0.0000%
867.2	Injury to ureter, without mention of open wound into cavity	0.0005%	0.0000%	0.0000%	0.0000%
845.13	Sprains and strains of interphalangeal (joint),toe	0.0000%	0.0000%	0.0021%	0.0000%
867.4	Injury to uterus, without mention of open wound into cavity	0.0005%	0.0000%	0.0000%	0.0000%
806.18	C5-C7 level fracture with central cord syndrome, open	0.0000%	0.0048%	0.0000%	0.0000%
811.10	Fracture in unspecified part of scapula, open	0.0005%	0.0000%	0.0000%	0.0000%
873.21	Open wound of nasal septum, without mention of complica	0.0005%	0.0000%	0.0000%	0.0000%
867.7	Injury to other specified pelvic organs, with open wound in	0.0005%	0.0000%	0.0000%	0.0000%

ICD-9-CM	ICD-9-CM	PureO	ContiB	NewB	PastB
847.0	Sprains and strains of neck	0.0000%	0.0000%	0.0021%	0.0000%
832.12	Open posterior dislocation of elbow	0.0005%	0.0000%	0.0000%	0.0000%
805.08	Fracture of multiple cervical vertebra, closed	0.0000%	0.0000%	0.0021%	0.0000%
803.01	Other and unqualified skull fracture, closed without mentio	0.0005%	0.0000%	0.0000%	0.0000%
813.40	Unspecified fracture of lower end of forearm, closed	0.0005%	0.0000%	0.0000%	0.0000%
813.92	Fracture in unspecified part of ulna (alone), open	0.0005%	0.0000%	0.0000%	0.0000%
852.29	Subdural hemorrhage following injury without mention of	0.0005%	0.0000%	0.0000%	0.0000%
832.13	Open medial dislocation of elbow	0.0000%	0.0000%	0.0021%	0.0000%
852.32	Subdural hemorrhage following injury with mention of ope	0.0000%	0.0048%	0.0000%	0.0000%
813.12	Fracture of coronoid process of ulna, open	0.0005%	0.0000%	0.0000%	0.0000%
848.0	Sprains and strains of septal cartilage of nose	0.0005%	0.0000%	0.0000%	0.0000%
811.19	Fracture in other part of scapula, open	0.0005%	0.0000%	0.0000%	0.0000%
812.54	Unspecified fracture of condyle(s) of humerus, open	0.0005%	0.0000%	0.0000%	0.0000%
861.02	Laceration of heart without penetration of heart chambers,	0.0000%	0.0000%	0.0021%	0.0000%
805.5	Fracture of lumbar vertebra, open	0.0005%	0.0000%	0.0000%	0.0000%
868.12	Injury to bile duct and gallbladder with open wound into ca	0.0005%	0.0000%	0.0000%	0.0000%
850.4	Concussion with prolonged loss of consciousness,without r	0.0005%	0.0000%	0.0000%	0.0000%
861.03	Laceration of heart with penetration of heart chambers, wit	0.0005%	0.0000%	0.0000%	0.0000%
873.65	Open wound of palate, without mention of complication	0.0000%	0.0000%	0.0021%	0.0000%
805.00	Fracture in unspecified level of cervical vertebra, closed	0.0000%	0.0000%	0.0000%	0.0032%
863.80	Injury to gastrointestinal tract,unspecified site,without men	0.0000%	0.0000%	0.0021%	0.0000%
868.19	Injury to other and multiple intra-abdominal organs with op	0.0000%	0.0000%	0.0021%	0.0000%
873.74	Open wound of tongue and floor of mouth, complicated	0.0005%	0.0000%	0.0000%	0.0000%
833.02	Closed dislocation of radiocarpal (joint)	0.0005%	0.0000%	0.0000%	0.0000%
873.8	Other and unspecified open wound of head, without mentic	0.0005%	0.0000%	0.0000%	0.0000%
861.13	Laceration of heart with penetration of heart chambers, wit	0.0005%	0.0000%	0.0000%	0.0000%
813.50	Unspecified fracture of lower end of forearm, open	0.0005%	0.0000%	0.0000%	0.0000%
807.3	Fracture of sternum, open	0.0005%	0.0000%	0.0000%	0.0000%
825.20	Fracture of unspecified bone(s) of foot〔except toes〕, clo	0.0005%	0.0000%	0.0000%	0.0000%
758.89	Other conditions due to chromosome anomalies	0.0005%	0.0000%	0.0000%	0.0000%
752.0	Anomalies of ovaries	0.0000%	0.0000%	0.0021%	0.0000%
800.54	Fracture of vault of skull, open without mention of intracra	0.0005%	0.0000%	0.0000%	0.0000%
787.1	Heartburn	0.0000%	0.0048%	0.0000%	0.0000%
765.15	Other preterm immaturity, 1250-1499g	0.0005%	0.0000%	0.0000%	0.0000%
737.20	Lordosis (acquired) (postural)	0.0005%	0.0000%	0.0000%	0.0000%
744.02	Other anomalies of external ear with impairment of hearing	0.0005%	0.0000%	0.0000%	0.0000%
756.15	Fusion of spine (vertebra), congenital	0.0005%	0.0000%	0.0000%	0.0000%
800.75	Fracture of vault of skull, open with subarchnoid, subdural,	0.0005%	0.0000%	0.0000%	0.0000%
787.6	Incontinence of feces	0.0000%	0.0048%	0.0000%	0.0000%

ICD-9-CM	ICD-9-CM	PureO	ContiB	NewB	PastB
764.06	Light-for-dates without mention of fetal malnutrition, 1500	0.0005%	0.0000%	0.0000%	0.0000%
752.7	Indeterminate sex and pseudohermaphroditism	0.0005%	0.0000%	0.0000%	0.0000%
743.66	Specified congenital anomalies of orbit	0.0005%	0.0000%	0.0000%	0.0000%
730.38	Periostitis without mention of osteomyelitis, other specified	0.0005%	0.0000%	0.0000%	0.0000%
754.43	Congenital bowing of tibia and fibula	0.0005%	0.0000%	0.0000%	0.0000%
769	Respiratory distress syndrome	0.0005%	0.0000%	0.0000%	0.0000%
754.59	Other varus deformities of feet	0.0005%	0.0000%	0.0000%	0.0000%
770.0	Congenital pneumonia	0.0005%	0.0000%	0.0000%	0.0000%
745.8	Other bulbus cordis anomalies and anomalies of cardiac se	0.0005%	0.0000%	0.0000%	0.0000%
788.29	Other specified retention of urine	0.0005%	0.0000%	0.0000%	0.0000%
730.04	Acute osteomyelitis, hand	0.0005%	0.0000%	0.0000%	0.0000%
750.4	Other specified anomalies of esophagus	0.0005%	0.0000%	0.0000%	0.0000%
752.19	Other anomalies of fallopian tubes and broad ligaments	0.0005%	0.0000%	0.0000%	0.0000%
730.91	Unspecified infection of bone, shoulder region	0.0000%	0.0000%	0.0021%	0.0000%
786.2	Cough	0.0000%	0.0000%	0.0021%	0.0000%
732.8	Other specified forms of osteochondropathy	0.0005%	0.0000%	0.0000%	0.0000%
801.41	Fracture of base of skull, closed with intracranial injury of	0.0005%	0.0000%	0.0000%	0.0000%
788.35	Post-void dribbling	0.0005%	0.0000%	0.0000%	0.0000%
744.9	Unspecified anomalies of face and neck	0.0005%	0.0000%	0.0000%	0.0000%
774.39	Neonatal jaundice due to delayed conjugation from other ca	0.0005%	0.0000%	0.0000%	0.0000%
736.73	Cavus deformity of foot	0.0005%	0.0000%	0.0000%	0.0000%
788.69	Other abnormality of urination	0.0005%	0.0000%	0.0000%	0.0000%
750.15	Macroglossia	0.0005%	0.0000%	0.0000%	0.0000%
755.21	Transverse deficiency of upper limb	0.0005%	0.0000%	0.0000%	0.0000%
755.8	Other specified anomalies of unspecified limb	0.0005%	0.0000%	0.0000%	0.0000%
774.6	Unspecified fetal and neonatal jaundice	0.0005%	0.0000%	0.0000%	0.0000%
759.3	Situs inversus	0.0005%	0.0000%	0.0000%	0.0000%
732.9	Unspecified osteochondropathy	0.0005%	0.0000%	0.0000%	0.0000%
783.1	Abnormal weight gain	0.0000%	0.0000%	0.0000%	0.0032%
741.90	Spina bifida wihtout mention of hydrocephalus, unspecified	0.0005%	0.0000%	0.0000%	0.0000%
800.72	Fracture of vault of skull, open with subarachnoid, subdura	0.0005%	0.0000%	0.0000%	0.0000%
775.6	Neonatal hypoglycaemia	0.0005%	0.0000%	0.0000%	0.0000%
784.5	Other speech disturbance	0.0000%	0.0000%	0.0021%	0.0000%
776.6	Anemia of prematurity	0.0005%	0.0000%	0.0000%	0.0000%
801.03	Fracture of base of skull, closed without mention of intracr	0.0005%	0.0000%	0.0000%	0.0000%
756.52	Osteopetrosis	0.0005%	0.0000%	0.0000%	0.0000%
801.10	Fracture of base of skull, closed with cerebral laceration an	0.0005%	0.0000%	0.0000%	0.0000%
736.5	Genu recurvatum (acquired)	0.0005%	0.0000%	0.0000%	0.0000%
762.3	Fetus or newborn affected by placental transfusion syndron	0.0005%	0.0000%	0.0000%	0.0000%

ICD-9-CM	ICD-9-CM	PureO	ContiB	NewB	PastB
730.02	Acute osteomyelitis, upper arm	0.0005%	0.0000%	0.0000%	0.0000%
765.14	Other preterm immaturity, 1000-1249g	0.0005%	0.0000%	0.0000%	0.0000%
779.3	Feeding problems in newborn	0.0005%	0.0000%	0.0000%	0.0000%
737.19	Kyphosis (acquired),other	0.0000%	0.0000%	0.0021%	0.0000%
756.54	Polyostotic fibrous dysplasia of bone	0.0005%	0.0000%	0.0000%	0.0000%
746.01	Congenital atresia of pulmonary valve	0.0005%	0.0000%	0.0000%	0.0000%
789.31	Abdominal or pelvic swelling, mass, or lump, right upper q	0.0005%	0.0000%	0.0000%	0.0000%
801.72	Fracture of base of skull, open with subarachnoid, subdural	0.0005%	0.0000%	0.0000%	0.0000%
779.8	Other specified conditions originating in the perinatal perio	0.0005%	0.0000%	0.0000%	0.0000%
800.12	Fracture of vault of skull, closed with cerebral laceration ar	0.0005%	0.0000%	0.0000%	0.0000%
733.10	Pathologic fracture, unspecified site	0.0005%	0.0000%	0.0000%	0.0000%
800.15	Fracture of vault of skull, closed with cerebral laceration ar	0.0000%	0.0000%	0.0000%	0.0032%
730.93	Unspecified infection of bone, forearm	0.0005%	0.0000%	0.0000%	0.0000%
746.7	Hypoplastic left heart syndrome	0.0005%	0.0000%	0.0000%	0.0000%
753.6	Atresia and stenosis of urethra and bladder neck	0.0005%	0.0000%	0.0000%	0.0000%
758.9	Conditons due to anomaly of unspecified chromosome	0.0005%	0.0000%	0.0000%	0.0000%
789.9	Other symptoms involving abdomen and pelvis	0.0000%	0.0000%	0.0000%	0.0032%
754.50	Talipes varus	0.0005%	0.0000%	0.0000%	0.0000%
755.39	Longitudinal deficiency, phalanges, complete or partial	0.0005%	0.0000%	0.0000%	0.0000%
746.82	Cor triatriatum	0.0005%	0.0000%	0.0000%	0.0000%
790.4	Nonspecific elevation of levels of transaminase or lactic ac	0.0000%	0.0000%	0.0021%	0.0000%
744.03	Anomaly of middle ear, except ossicles	0.0005%	0.0000%	0.0000%	0.0000%
790.5	Other nonspecific abnormal serum enzyme levels	0.0005%	0.0000%	0.0000%	0.0000%
800.34	Fracture of vault of skull, closed with other and unspecified	0.0000%	0.0000%	0.0021%	0.0000%
755.34	Longitudinal deficiency, femoral, complete or parital(with	0.0005%	0.0000%	0.0000%	0.0000%
800.44	Fracture of vault of skull, closed with intracranial injury of	0.0000%	0.0000%	0.0021%	0.0000%
738.7	Cauliflower ear	0.0005%	0.0000%	0.0000%	0.0000%
800.53	Fracture of vault of skull, open without mention of intracra	0.0005%	0.0000%	0.0000%	0.0000%
790.93	Elevated prostate specific antigen (PSA)	0.0000%	0.0000%	0.0000%	0.0032%
751.8	Other specified anomalies of digestive system	0.0005%	0.0000%	0.0000%	0.0000%
750.6	Congenital hiatus hernia	0.0005%	0.0000%	0.0000%	0.0000%
754.69	Other valgus deformities of feet	0.0005%	0.0000%	0.0000%	0.0000%
733.92	Chondromalacia	0.0005%	0.0000%	0.0000%	0.0000%
742.0	Encephalocele	0.0005%	0.0000%	0.0000%	0.0000%
753.9	Unspecified anomalies of urinary system	0.0000%	0.0000%	0.0021%	0.0000%
800.74	Fracture of vault of skull, open with subarachnoid, subdura	0.0005%	0.0000%	0.0000%	0.0000%
793.5	Nonspecific abnormal findings on radiological and other ex	0.0000%	0.0048%	0.0000%	0.0000%
751.9	Unspecified anomalies of digestive system	0.0005%	0.0000%	0.0000%	0.0000%
794.09	Other nonspecific abnormal results of function studies, brai	0.0000%	0.0000%	0.0021%	0.0000%

ICD-9-CM	ICD-9-CM	PureO	ContiB	NewB	PastB
736.01	Cubitus valgus (acquired)	0.0005%	0.0000%	0.0000%	0.0000%
794.31	Abnormal electrocardiogram〔ECG〕〔EKG〕	0.0005%	0.0000%	0.0000%	0.0000%
730.98	Unspecified infection of bone, other specified sites	0.0005%	0.0000%	0.0000%	0.0000%
794.4	Nonspecific abnormal results of function studies, kidney	0.0005%	0.0000%	0.0000%	0.0000%
801.04	Fracture of base of skull, closed without mention of intracr	0.0005%	0.0000%	0.0000%	0.0000%
728.4	Laxity of ligament	0.0000%	0.0000%	0.0021%	0.0000%
730.10	Chronic osteomyelitis, unspecified site	0.0000%	0.0000%	0.0021%	0.0000%
750.8	Other specified anomalies of upper alimentary tract	0.0005%	0.0000%	0.0000%	0.0000%
754.79	Other deformities of feet	0.0005%	0.0000%	0.0000%	0.0000%
796.3	Nonspecific low blood pressure reading	0.0000%	0.0048%	0.0000%	0.0000%
736.05	Wrist drop (acquired)	0.0005%	0.0000%	0.0000%	0.0000%
743.10	Microphthalmos, unspecified	0.0005%	0.0000%	0.0000%	0.0000%
742.1	Microcephalus	0.0000%	0.0000%	0.0000%	0.0032%
727.9	Unspecified disorder of synovium, tendon, and bursa	0.0005%	0.0000%	0.0000%	0.0000%
765.03	Extreme immaturity, 750-999g	0.0005%	0.0000%	0.0000%	0.0000%
733.12	Pathologic fracture of distal radius and ulna	0.0005%	0.0000%	0.0000%	0.0000%
752.42	Imperforate hymen	0.0005%	0.0000%	0.0000%	0.0000%
754.1	Certain congenital musculoskeletal deformities of sternocle	0.0005%	0.0000%	0.0000%	0.0000%
737.12	Kyphosis, postlaminectomy	0.0000%	0.0000%	0.0021%	0.0000%
731.2	Hypertrophic pulmonary osteoarthropathy	0.0000%	0.0000%	0.0021%	0.0000%
746.9	Unspecified anomaly of heart	0.0005%	0.0000%	0.0000%	0.0000%
743.65	Specified congenital anomalies of lacrimal passages	0.0005%	0.0000%	0.0000%	0.0000%
765.16	Other preterm immaturity, 1500-1749g	0.0005%	0.0000%	0.0000%	0.0000%
735.4	Other hammer toe (acquired)	0.0000%	0.0000%	0.0000%	0.0032%
765.19	Other preterm immaturity, 2500g and over	0.0005%	0.0000%	0.0000%	0.0000%
755.55	Acrocephlosyndactyly	0.0005%	0.0000%	0.0000%	0.0000%
786.8	Hiccough	0.0005%	0.0000%	0.0000%	0.0000%
755.14	Syndactyly of toes with fusion of bone	0.0005%	0.0000%	0.0000%	0.0000%
739.1	Nonallopathic lesions, cervical region	0.0000%	0.0000%	0.0021%	0.0000%
730.29	Unspecified osteomyelitis, multiple sites	0.0005%	0.0000%	0.0000%	0.0000%
730.00	Acute osteomyelitis, unspecified site	0.0000%	0.0000%	0.0021%	0.0000%
736.30	Unspecified acquired deformity of hip	0.0000%	0.0000%	0.0000%	0.0032%
756.13	Absence of vertebra, congenital	0.0005%	0.0000%	0.0000%	0.0000%
695.0	Toxic erythema	0.0000%	0.0048%	0.0000%	0.0000%
664.24	Third-degree perineal laceration, postpartum condition or c	0.0005%	0.0000%	0.0000%	0.0000%
673.12	Amniotic fluid embolism, delivered, with mention of postp	0.0005%	0.0000%	0.0000%	0.0000%
665.22	Inversion of uterus, delivered, with mention of postpartum	0.0005%	0.0000%	0.0000%	0.0000%
719.48	Pain in joint, other specified sites	0.0005%	0.0000%	0.0000%	0.0000%
647.91	Unspecified infection or infestation in the mother classifiab	0.0005%	0.0000%	0.0000%	0.0000%

ICD-9-CM	ICD-9-CM	PureO	ContiB	NewB	PastB
652.01	Unsrable lie, delivered, with or without mention of antepar	0.0005%	0.0000%	0.0000%	0.0000%
719.58	Stiffness of joint, not elsewhere classified, other specified s	0.0005%	0.0000%	0.0000%	0.0000%
658.81	Other problems associated with amniotic cavity and membr	0.0005%	0.0000%	0.0000%	0.0000%
659.73	Abnormality in fetal heart rate or rhythm, antepartum cond	0.0005%	0.0000%	0.0000%	0.0000%
680.0	Carbuncle and furuncle, face	0.0000%	0.0000%	0.0021%	0.0000%
719.62	Other symptoms referable to joint, upper arm	0.0005%	0.0000%	0.0000%	0.0000%
648.73	Bone and joint disorders of back,pelvis and lower limbs in	0.0005%	0.0000%	0.0000%	0.0000%
719.63	Other symptoms referable to joint, forearm	0.0005%	0.0000%	0.0000%	0.0000%
724.09	Other spinal stenosis	0.0005%	0.0000%	0.0000%	0.0000%
659.81	Other specified indications for care or intervention related t	0.0005%	0.0000%	0.0000%	0.0000%
648.83	Abnormal glucose tolerance in the mother, classifiable else	0.0005%	0.0000%	0.0000%	0.0000%
665.51	Other injury to pelvic organs, delivered, with or without me	0.0005%	0.0000%	0.0000%	0.0000%
674.01	Cerebrovascular disorders in the puerperium, delivered, wit	0.0000%	0.0000%	0.0000%	0.0032%
646.23	Unspecified renal disease in pregnancy, without mention of	0.0000%	0.0000%	0.0000%	0.0032%
718.93	Unspecified derangement of joint, forearm	0.0000%	0.0000%	0.0000%	0.0032%
665.64	Damage to pelvic joints and ligaments, postpartum conditio	0.0005%	0.0000%	0.0000%	0.0000%
719.17	Hemarthrosis, ankle and foot	0.0000%	0.0048%	0.0000%	0.0000%
716.98	Arthropathy, unspecified, other specified sites	0.0005%	0.0000%	0.0000%	0.0000%
716.56	Unspecified polyarthropathy or polyarthritis, lower leg	0.0000%	0.0048%	0.0000%	0.0000%
648.63	Other cardiovascular diseases in the mother classifiable els	0.0005%	0.0000%	0.0000%	0.0000%
665.03	Rupture of uterus before onset of labor, antepartum conditi	0.0005%	0.0000%	0.0000%	0.0000%
654.44	Other abnormalities in shape or position of gravid uterus ar	0.0005%	0.0000%	0.0000%	0.0000%
669.82	Other complications of labor and delivery, not elsewhere cl	0.0005%	0.0000%	0.0000%	0.0000%
719.84	Other specified disorders of joint, hand	0.0000%	0.0000%	0.0000%	0.0032%
669.91	Unspecified complication of labor and delivery, delivered,	0.0005%	0.0000%	0.0000%	0.0000%
709.1	Vascular disorders of skin	0.0005%	0.0000%	0.0000%	0.0000%
715.28	Osteoarthrosis, licalized, secondary, other specified sites	0.0005%	0.0000%	0.0000%	0.0000%
665.84	Other specified obstetrical trauma, postpartum condition or	0.0005%	0.0000%	0.0000%	0.0000%
718.53	Ankylosis of joint, forearm	0.0005%	0.0000%	0.0000%	0.0000%
660.41	Shoulder (girdle) dystocia, delivered, with or without ment	0.0005%	0.0000%	0.0000%	0.0000%
718.57	Ankylosis of joint, ankle and foot	0.0005%	0.0000%	0.0000%	0.0000%
651.13	Triplet pregnancy, antepartum condition or complication	0.0005%	0.0000%	0.0000%	0.0000%
673.24	Obstetrical blood-clot embolism, postpartum condition or c	0.0005%	0.0000%	0.0000%	0.0000%
648.03	Diabetes mellitus conditions in the mother classifiable else	0.0005%	0.0000%	0.0000%	0.0000%
726.4	Enthesopathy of wrist and carpus	0.0000%	0.0000%	0.0021%	0.0000%
719.94	Unspecified disorder of joint, hand	0.0005%	0.0000%	0.0000%	0.0000%
726.64	Patellar tendinitis	0.0005%	0.0000%	0.0000%	0.0000%
719.95	Unspecified disorder of joint, pelvic region and thigh	0.0005%	0.0000%	0.0000%	0.0000%
703.9	Unspecified disease of nail	0.0000%	0.0000%	0.0021%	0.0000%

ICD-9-CM	ICD-9-CM	PureO	ContiB	NewB	PastB
709.9	Unspecified disorder of skin and subcutaneous tissue	0.0005%	0.0000%	0.0000%	0.0000%
716.13	Traumatic arthropathy, forearm	0.0005%	0.0000%	0.0000%	0.0000%
660.91	Unspecified obstructed labor, delivered, with or without me	0.0005%	0.0000%	0.0000%	0.0000%
659.41	Grand multiparity, delivered, with or without mention of an	0.0005%	0.0000%	0.0000%	0.0000%
653.21	Inlet contraction of pelvis, delivered, with or without menti	0.0005%	0.0000%	0.0000%	0.0000%
716.36	Climacteric arthritis, lower leg	0.0005%	0.0000%	0.0000%	0.0000%
720.1	Spinal enthesopathy	0.0005%	0.0000%	0.0000%	0.0000%
664.54	Vulval and perineal hematoma, postpartum condition or co	0.0005%	0.0000%	0.0000%	0.0000%
661.03	Primary uterine inertia, antepartum condition or complicati	0.0005%	0.0000%	0.0000%	0.0000%
664.81	Other specified trauma to perineum and vulva, delivered, w	0.0005%	0.0000%	0.0000%	0.0000%
654.71	Congenital or acquired abnormality of vagina, delivered, w	0.0005%	0.0000%	0.0000%	0.0000%
723.2	Cervicocranial syndrome	0.0005%	0.0000%	0.0000%	0.0000%
661.23	Other and unspecified uterine inertia, antepartum condition	0.0005%	0.0000%	0.0000%	0.0000%
642.54	Severe pre-eclampsia, postpartum condition or complicatio	0.0005%	0.0000%	0.0000%	0.0000%
657.03	Polyhydramnios, antepartum condition or complication	0.0005%	0.0000%	0.0000%	0.0000%
718.41	Contracture of joint, shoulder region	0.0005%	0.0000%	0.0000%	0.0000%
711.00	Pyogenic arthritis, site unspecified	0.0005%	0.0000%	0.0000%	0.0000%
669.84	Other complications of labor and delivery, not elsewhere cl	0.0005%	0.0000%	0.0000%	0.0000%
661.93	Unspecified abnormality of labor, antepartum condition or	0.0005%	0.0000%	0.0000%	0.0000%
695.4	Lupus erythematosus	0.0000%	0.0000%	0.0021%	0.0000%
654.83	Congenital or acquired abnormality of vulva, antepartum co	0.0005%	0.0000%	0.0000%	0.0000%
695.81	Ritter's disease	0.0005%	0.0000%	0.0000%	0.0000%
668.04	Pulmonary complications of the administration of anestheti	0.0005%	0.0000%	0.0000%	0.0000%
651.83	Other specified multiple gestation,antepartum condition or	0.0005%	0.0000%	0.0000%	0.0000%
668.82	Other complications of anesthesia or other sedation in labo	0.0005%	0.0000%	0.0000%	0.0000%
663.51	Vasa previa, delivered, with or without mention of antepart	0.0005%	0.0000%	0.0000%	0.0000%
669.01	Maternal distress, delivered, with or without mention of an	0.0005%	0.0000%	0.0000%	0.0000%
718.54	Ankylosis of joint, hand	0.0005%	0.0000%	0.0000%	0.0000%
690.10	Seborrheic dermatitis, unspecified	0.0000%	0.0000%	0.0021%	0.0000%
671.83	Other venous complications in pregnancy and the puerperiu	0.0005%	0.0000%	0.0000%	0.0000%
718.02	Articular cartilage disorder, upper arm	0.0005%	0.0000%	0.0000%	0.0000%
718.65	Unspecified intrapelvic protrusion of acetabulum, pelvic re	0.0005%	0.0000%	0.0000%	0.0000%
691.0	Diaper or napkin rash	0.0000%	0.0048%	0.0000%	0.0000%
673.14	Amniotic fluid embolism, postpartum condition or complic	0.0005%	0.0000%	0.0000%	0.0000%
711.08	Pyogenic arthritis, other specified sites	0.0005%	0.0000%	0.0000%	0.0000%
673.84	Other pulmonary embolism, postpartum condition or comp	0.0005%	0.0000%	0.0000%	0.0000%
669.04	Maternal distress, postpartum condition or complication	0.0000%	0.0000%	0.0000%	0.0032%
674.04	Cerebrovascular disorders in the puerperium, postpartum co	0.0000%	0.0000%	0.0021%	0.0000%
692.4	Contact dermatitis and other eczema, due to other chemical	0.0000%	0.0000%	0.0021%	0.0000%

ICD-9-CM	ICD-9-CM	PureO	ContiB	NewB	PastB
655.53	Suspected damage to fetus frpm drugs, affecting manageme	0.0000%	0.0000%	0.0021%	0.0000%
722.32	Schmorl's nodes, lumbar region	0.0005%	0.0000%	0.0000%	0.0000%
726.61	Pes anserinus tendinitis or bursitis	0.0005%	0.0000%	0.0000%	0.0000%
718.11	Loose body in joint, shoulder region	0.0005%	0.0000%	0.0000%	0.0000%
648.13	Thyroid dysfunction conditions in the mother classifiable e	0.0005%	0.0000%	0.0000%	0.0000%
669.11	Shock during or following labor and delivery, delivered, w	0.0005%	0.0000%	0.0000%	0.0000%
642.64	Eclampsia, postpartum condition or complication	0.0005%	0.0000%	0.0000%	0.0000%
718.15	Loose body in joint, pelvic region and thigh	0.0000%	0.0000%	0.0021%	0.0000%
704.00	Alopecia, unspecified	0.0000%	0.0048%	0.0000%	0.0000%
722.70	Intervertebral disc disorder with myelopathy, unspecified re	0.0000%	0.0000%	0.0021%	0.0000%
726.8	Other peripheral enthesopathies	0.0005%	0.0000%	0.0000%	0.0000%
711.97	Unspecified infective arthritis, ankle and foot	0.0005%	0.0000%	0.0000%	0.0000%
645.13	Prolonged pregnancy, antepartum condition or complication	0.0005%	0.0000%	0.0000%	0.0000%
718.22	Pathological dislocation, upper arm	0.0005%	0.0000%	0.0000%	0.0000%
719.21	Villonodular synovitis, shoulder region	0.0000%	0.0000%	0.0021%	0.0000%
711.98	Unspecified infective arthritis, other specified sites	0.0005%	0.0000%	0.0000%	0.0000%
705.89	Other specified disorders of sweat glands	0.0005%	0.0000%	0.0000%	0.0000%
711.99	Unspecified infective arthritis, multiple sites	0.0000%	0.0000%	0.0021%	0.0000%
716.18	Traumatic arthropathy, other specified sites	0.0005%	0.0000%	0.0000%	0.0000%
692.89	Dermatitis due to other specified agents	0.0005%	0.0000%	0.0000%	0.0000%
719.30	Palindromic rheumatism, unspecified	0.0005%	0.0000%	0.0000%	0.0000%
714.2	Other rheumatoid arthritis with visceral or systemic involve	0.0005%	0.0000%	0.0000%	0.0000%
656.11	Rhesus isoimmunization, affecting management of mother	0.0005%	0.0000%	0.0000%	0.0000%
653.31	Outlet contraction of pelvis, delivered, with or without men	0.0005%	0.0000%	0.0000%	0.0000%
716.80	Other specified arthropathy, unspecified site	0.0005%	0.0000%	0.0000%	0.0000%
658.10	Premature rupture of membranes, unspecified as to episode	0.0005%	0.0000%	0.0000%	0.0000%
716.81	Other specified arthropathy, shoulder region	0.0000%	0.0000%	0.0000%	0.0032%
718.33	Recurrent dislocation of joint, forearm	0.0000%	0.0000%	0.0021%	0.0000%
645.21	Prolonged pregnancy, delivered, with or without mention o	0.0005%	0.0000%	0.0000%	0.0000%
715.12	Osteoarthrosis, localized, primary, upper arm	0.0000%	0.0000%	0.0000%	0.0032%
693.1	Dermatitis due to food	0.0005%	0.0000%	0.0000%	0.0000%
525.1	Loss of teeth due to accident, extraction, or local periodont	0.0000%	0.0000%	0.0000%	0.0032%
636.02	Illegally induced abortion, complicated by genital tract and	0.0005%	0.0000%	0.0000%	0.0000%
573.0	Chronic passive congestion of liver	0.0005%	0.0000%	0.0000%	0.0000%
576.9	Unspecified disorder of biliary tract	0.0005%	0.0000%	0.0000%	0.0000%
623.4	Old vaginal laceration	0.0005%	0.0000%	0.0000%	0.0000%
533.20	Peptic ulcer, site unspecified, acute with hemorrhage and p	0.0005%	0.0000%	0.0000%	0.0000%
583.0	Nephritis and nephropathy, not specified as acute or chroni	0.0005%	0.0000%	0.0000%	0.0000%
567.1	Pneumococcal peritonitis	0.0000%	0.0000%	0.0021%	0.0000%

ICD-9-CM	ICD-9-CM	PureO	ContiB	NewB	PastB
576.4	Fistula of bile duct	0.0005%	0.0000%	0.0000%	0.0000%
528.2	Oral aphthae	0.0005%	0.0000%	0.0000%	0.0000%
586	Renal failure, unspecified	0.0005%	0.0000%	0.0000%	0.0000%
626.0	Absence of menstruation	0.0005%	0.0000%	0.0000%	0.0000%
624.5	Hematoma of vulva	0.0005%	0.0000%	0.0000%	0.0000%
579.0	Celiac disease	0.0000%	0.0048%	0.0000%	0.0000%
556.4	Pseudopolyposis of colon	0.0005%	0.0000%	0.0000%	0.0000%
626.4	Irregular menstrual cycle	0.0005%	0.0000%	0.0000%	0.0000%
583.2	Nephritis and nephropathy, not specified as acute or chroni	0.0000%	0.0048%	0.0000%	0.0000%
602.0	Calculus of prostate	0.0005%	0.0000%	0.0000%	0.0000%
552.01	Femoral hernia with obstruction, unilateral or unspecfied, r	0.0005%	0.0000%	0.0000%	0.0000%
551.20	Ventral hernia unspecified, with gangrene	0.0005%	0.0000%	0.0000%	0.0000%
597.80	Urethritis, unspecified	0.0000%	0.0000%	0.0000%	0.0032%
532.61	Duodenal ulcer, chronic or unspecified with hemorrhage ar	0.0005%	0.0000%	0.0000%	0.0000%
597.89	Other urethritis	0.0005%	0.0000%	0.0000%	0.0000%
535.20	Gastric mucosal hypertrophy, without mention of hemorrha	0.0000%	0.0048%	0.0000%	0.0000%
590.3	Pyeloureteritis cystica	0.0005%	0.0000%	0.0000%	0.0000%
524.5	Dentofacial functional abnormalities	0.0005%	0.0000%	0.0000%	0.0000%
601.3	Prostatocystitis	0.0000%	0.0000%	0.0021%	0.0000%
627.2	Menopausal or female climacteric states	0.0000%	0.0048%	0.0000%	0.0000%
589.0	Unilateral small kidney of unknown cause	0.0005%	0.0000%	0.0000%	0.0000%
628.0	Infertility, female, associated with anovulation	0.0005%	0.0000%	0.0000%	0.0000%
641.83	Other antepartum hemorrhage, antepartum condition or cor	0.0005%	0.0000%	0.0000%	0.0000%
608.86	Edema of male genital organs	0.0005%	0.0000%	0.0000%	0.0000%
529.6	Glossodynia	0.0000%	0.0000%	0.0000%	0.0032%
533.60	Peptic ulcer, site unspecified, chronic or unspecified with h	0.0005%	0.0000%	0.0000%	0.0000%
583.1	Nephritis and nephropathy, not specified as acute or chroni	0.0000%	0.0048%	0.0000%	0.0000%
562.03	Diverticulitis of small intestine with hemorrhage	0.0005%	0.0000%	0.0000%	0.0000%
596.55	Detrusor sphincter dyssynergia	0.0000%	0.0000%	0.0000%	0.0032%
581.0	Nephrotic syndrome, with lesion of proliferative glomerulo	0.0000%	0.0000%	0.0000%	0.0032%
564.4	Other postoperative functional disorders	0.0000%	0.0000%	0.0000%	0.0032%
571.3	Alcoholic liver damage, unspecified	0.0000%	0.0000%	0.0021%	0.0000%
533.91	Peptic ulcer, site unspecified, unspecified as acute or chron	0.0005%	0.0000%	0.0000%	0.0000%
629.9	Unspecified disorder of female genital organs	0.0005%	0.0000%	0.0000%	0.0000%
558.3	Allergic gastroenteritis and colitis	0.0005%	0.0000%	0.0000%	0.0000%
533.71	Peptic ulcer, site unspecified, chronic without mention of h	0.0000%	0.0000%	0.0021%	0.0000%
556.3	Ulcerative colitis, ulcerative (chronic) proctosigmoiditis	0.0000%	0.0000%	0.0000%	0.0032%
621.2	Hypertrophy of uterus	0.0000%	0.0000%	0.0000%	0.0032%
637.02	Unspecified abortion, complicated by genital tract and pelv	0.0005%	0.0000%	0.0000%	0.0000%

ICD-9-CM	ICD-9-CM	PureO	ContiB	NewB	PastB
534.60	Gastrojejunal ulcer, chronic or unspecified with hemorrhag	0.0000%	0.0000%	0.0021%	0.0000%
606.0	Azoospermia	0.0000%	0.0048%	0.0000%	0.0000%
603.1	Infected hydrocele	0.0005%	0.0000%	0.0000%	0.0000%
576.5	Spasm of sphincter of Oddi	0.0005%	0.0000%	0.0000%	0.0000%
569.84	Angiodysplasia of intestine (without mention of hemorrhag	0.0005%	0.0000%	0.0000%	0.0000%
616.0	Cervicitis and endocervicitis	0.0005%	0.0000%	0.0000%	0.0000%
524.62	Arthralgia of temporomandibular joint	0.0000%	0.0000%	0.0000%	0.0032%
524.71	Alveolar maxillary hyperplasia	0.0000%	0.0000%	0.0021%	0.0000%
534.91	Gastrojejunal ulcer, unspecified as acute or chronic, withou	0.0005%	0.0000%	0.0000%	0.0000%
641.00	Placenta previa without hemorrhage, unspecified as to episo	0.0005%	0.0000%	0.0000%	0.0000%
531.71	Gastric ulcer, chronic without mention of hemorrhage or pe	0.0005%	0.0000%	0.0000%	0.0000%
523.9	Unspecified gingival and periodontal disease	0.0005%	0.0000%	0.0000%	0.0000%
603.8	Other specified types of hydrocele	0.0005%	0.0000%	0.0000%	0.0000%
536.42	Mechanical complication of gastrostomy	0.0005%	0.0000%	0.0000%	0.0000%
569.61	Infection of colostomy or enterostomy	0.0005%	0.0000%	0.0000%	0.0000%
531.20	Gastric ulcer, acute with hemorrhage and perforation, witho	0.0005%	0.0000%	0.0000%	0.0000%
600.3	Cyst of prostate	0.0005%	0.0000%	0.0000%	0.0000%
625.2	Mittelschmerz	0.0005%	0.0000%	0.0000%	0.0000%
621.9	Unspecified disorders of uterus	0.0005%	0.0000%	0.0000%	0.0000%
634.52	Abortion, complicated by shock, complete	0.0005%	0.0000%	0.0000%	0.0000%
642.21	Other pre-existing hypertension complicating pregnancy, de	0.0005%	0.0000%	0.0000%	0.0000%
626.9	Unspecified disorder of menstruation and other abnormal b	0.0000%	0.0000%	0.0000%	0.0032%
642.33	Transient hypertension of pregnancy, antepartum condition	0.0005%	0.0000%	0.0000%	0.0000%
594.9	Calculus of lower urinary tract, unspecified	0.0000%	0.0000%	0.0000%	0.0032%
579.8	Other specified intestinal malabsorption	0.0005%	0.0000%	0.0000%	0.0000%
599.9	Unspecified disorder of urethra and urinary tract	0.0000%	0.0000%	0.0000%	0.0032%
576.3	Perforation of bile duct	0.0005%	0.0000%	0.0000%	0.0000%
396.9	Mitral and aortic valve diseases, unspecified	0.0000%	0.0000%	0.0021%	0.0000%
368.16	Psychophysical visual disturbances	0.0005%	0.0000%	0.0000%	0.0000%
506.0	Bronchitis and pneumonitis due to fumes and vapors	0.0000%	0.0000%	0.0021%	0.0000%
365.23	Chronic angle-closure glaucoma	0.0005%	0.0000%	0.0000%	0.0000%
429.9	Heart disease, unspecified	0.0000%	0.0000%	0.0000%	0.0032%
414.03	Coronary atherosclerosis of nonautolgous biological bypass	0.0005%	0.0000%	0.0000%	0.0000%
402.90	Unspecified hypertensive heart disease without congestive	0.0005%	0.0000%	0.0000%	0.0000%
414.10	Aneurysm of heart (wall)	0.0000%	0.0000%	0.0021%	0.0000%
375.32	Acute dacryocystitis	0.0005%	0.0000%	0.0000%	0.0000%
376.40	Deformity of orbit, unspecified	0.0000%	0.0000%	0.0021%	0.0000%
374.04	Cicatricial entropion	0.0005%	0.0000%	0.0000%	0.0000%
385.83	Retained foreign body of middle ear	0.0005%	0.0000%	0.0000%	0.0000%

ICD-9-CM	ICD-9-CM	PureO	ContiB	NewB	PastB
376.10	Chronic inflammation of orbit, unspecified	0.0000%	0.0000%	0.0021%	0.0000%
366.22	Total traumatic cataract	0.0005%	0.0000%	0.0000%	0.0000%
493.02	Extrinsic asthma with acute exacerbation	0.0000%	0.0000%	0.0000%	0.0032%
440.1	Atherosclerosis of renal artery	0.0005%	0.0000%	0.0000%	0.0000%
376.00	Acute inflammation of orbit, unspecified	0.0005%	0.0000%	0.0000%	0.0000%
405.09	Other malignant secondary hypertension	0.0000%	0.0000%	0.0000%	0.0032%
451.11	Phlebitis and thrombophlebitis of femoral vein (deep) (supe	0.0005%	0.0000%	0.0000%	0.0000%
414.19	Other aneurysm of heart	0.0005%	0.0000%	0.0000%	0.0000%
371.48	Peripheral degenerations of cornea	0.0000%	0.0000%	0.0021%	0.0000%
378.24	Intermittent exotropia, alternating	0.0005%	0.0000%	0.0000%	0.0000%
379.91	Pain in or around eye	0.0005%	0.0000%	0.0000%	0.0000%
381.60	Obstruction of Eustachian tube, unspecified	0.0005%	0.0000%	0.0000%	0.0000%
429.71	Acquired cardiac septal defect	0.0000%	0.0048%	0.0000%	0.0000%
415.11	Iatrogenic pulmonary embolism and infarction	0.0005%	0.0000%	0.0000%	0.0000%
365.22	Acute angle-closure glaucoma	0.0005%	0.0000%	0.0000%	0.0000%
370.01	Marginal corneal ulcer	0.0000%	0.0000%	0.0000%	0.0032%
376.89	Other orbital disorders	0.0000%	0.0000%	0.0021%	0.0000%
371.82	Corneal disorder due to contact lens	0.0005%	0.0000%	0.0000%	0.0000%
443.89	Other specified peripheral vascular diseases	0.0005%	0.0000%	0.0000%	0.0000%
433.80	Occlusion and stenosis of other specified precerebral artery	0.0005%	0.0000%	0.0000%	0.0000%
410.42	Acute myocardial infarction of other inferior wall, subsequ	0.0005%	0.0000%	0.0000%	0.0000%
476.0	Chronic laryngitis	0.0005%	0.0000%	0.0000%	0.0000%
375.30	Dacryocystitis, unspecified	0.0000%	0.0000%	0.0000%	0.0032%
477.8	Allergic rhinitis due to other allergen	0.0005%	0.0000%	0.0000%	0.0000%
460	Acute nasopharyngitis [common cold]	0.0000%	0.0048%	0.0000%	0.0000%
416.9	Chronic pulmonary heart disease, unspecified	0.0005%	0.0000%	0.0000%	0.0000%
427.2	Paroxysmal tachycardia, unspecified	0.0005%	0.0000%	0.0000%	0.0000%
417.1	Aneurysm of pulmonary artery	0.0000%	0.0000%	0.0021%	0.0000%
378.52	Third or oculomotor nerve palsy, total	0.0005%	0.0000%	0.0000%	0.0000%
374.34	Blepharochalasis	0.0000%	0.0000%	0.0021%	0.0000%
461.3	Acute sinusitis of sphenoidal	0.0005%	0.0000%	0.0000%	0.0000%
451.82	Phlebitis and thrombophlebitis of superficial veins of upper	0.0005%	0.0000%	0.0000%	0.0000%
403.01	Malignant hypertensive renal disease with renal failure	0.0000%	0.0048%	0.0000%	0.0000%
451.89	Phlebitis and thrombophlebitis of other sites	0.0005%	0.0000%	0.0000%	0.0000%
516.3	Idiopathic fibrosing alveolitis	0.0005%	0.0000%	0.0000%	0.0000%
379.29	Other disorders of vitreous	0.0000%	0.0048%	0.0000%	0.0000%
518.2	Compensatory emphysema	0.0000%	0.0000%	0.0021%	0.0000%
370.03	Central corneal ulcer	0.0005%	0.0000%	0.0000%	0.0000%
371.55	Macular corneal dystrophy	0.0005%	0.0000%	0.0000%	0.0000%

ICD-9-CM	ICD-9-CM	PureO	ContiB	NewB	PastB
434.00	Cerebral thrombosis without mention of cerebral infarction	0.0005%	0.0000%	0.0000%	0.0000%
446.1	Acute febrile mucocutaneous lymph node syndrome (MCL	0.0005%	0.0000%	0.0000%	0.0000%
421.9	Acute endocarditis, unspecified	0.0005%	0.0000%	0.0000%	0.0000%
464.31	Acute epiglottitis with obstruction	0.0000%	0.0000%	0.0021%	0.0000%
376.9	Unspecified disorder of orbit	0.0000%	0.0048%	0.0000%	0.0000%
376.51	Enophthalmos due to atrophy of orbital tissue	0.0005%	0.0000%	0.0000%	0.0000%
374.05	Trichiasis without entropion	0.0005%	0.0000%	0.0000%	0.0000%
413.0	Angina decubitus	0.0000%	0.0000%	0.0021%	0.0000%
382.4	Unspecified suppurative otitis media	0.0000%	0.0000%	0.0000%	0.0032%
522.4	Acute apical periodontitis of pulpal origin	0.0000%	0.0000%	0.0000%	0.0032%
478.34	Paralysis of vocal cords or larynx, bilateral, complete	0.0000%	0.0000%	0.0000%	0.0032%
404.02	Malignant hypertensive heart and renal disease with renal f	0.0000%	0.0000%	0.0021%	0.0000%
376.11	Orbital granuloma	0.0000%	0.0048%	0.0000%	0.0000%
426.50	Bundle branch block, unspecified	0.0000%	0.0048%	0.0000%	0.0000%
422.99	Other and unspecified acute myocarditis	0.0005%	0.0000%	0.0000%	0.0000%
493.12	Intrinsic asthma with acute exacerbation	0.0000%	0.0048%	0.0000%	0.0000%
423.0	Hemopericardium	0.0005%	0.0000%	0.0000%	0.0000%
493.21	Chronic obstructive asthma (with obstructive pulmonary di	0.0000%	0.0000%	0.0000%	0.0032%
378.16	Alternating exotropia with A pattern	0.0005%	0.0000%	0.0000%	0.0000%
387.0	Otosclerosis involving oval window, nonobliterative	0.0005%	0.0000%	0.0000%	0.0000%
478.71	Cellulitis and perichondritis disease of larynx	0.0005%	0.0000%	0.0000%	0.0000%
410.70	Acute subendocardial infarction, episode of care unspecifie	0.0000%	0.0000%	0.0000%	0.0032%
362.83	Retinal edema	0.0005%	0.0000%	0.0000%	0.0000%
395.1	Rheumatic aortic insufficiency	0.0005%	0.0000%	0.0000%	0.0000%
410.10	Acute myocardial infarction of other anterior wall, episode	0.0000%	0.0048%	0.0000%	0.0000%
379.04	Scleromalacia perforans	0.0005%	0.0000%	0.0000%	0.0000%
437.8	Other and Ill-defined cerebrovascular disease	0.0000%	0.0000%	0.0000%	0.0032%
373.2	Chalazion	0.0005%	0.0000%	0.0000%	0.0000%
480.1	Pneumonia due to respiratory syncytial virus	0.0000%	0.0048%	0.0000%	0.0000%
508.8	Respiratory conditions due to other specified external agent	0.0000%	0.0048%	0.0000%	0.0000%
364.23	Lens-induced iridocyclitis	0.0005%	0.0000%	0.0000%	0.0000%
384.00	Acute myringitis, unspecified	0.0005%	0.0000%	0.0000%	0.0000%
410.12	Acute myocardial infarction of other anterior wall, subsequ	0.0000%	0.0000%	0.0021%	0.0000%
434.90	Unspecified cerebral artery occlusion without mention of c	0.0005%	0.0000%	0.0000%	0.0000%
365.59	Glaucoma associated with other lens disorders	0.0005%	0.0000%	0.0000%	0.0000%
461.2	Acute sinusitis of ethmoidal	0.0005%	0.0000%	0.0000%	0.0000%
375.00	Dacryoadenitis, unspecified	0.0005%	0.0000%	0.0000%	0.0000%
368.00	Amblyopia, unspecified	0.0005%	0.0000%	0.0000%	0.0000%
389.8	Other specified forms of hearing loss	0.0005%	0.0000%	0.0000%	0.0000%

ICD-9-CM	ICD-9-CM	PureO	ContiB	NewB	PastB
371.53	Granular corneal dystrophy	0.0005%	0.0000%	0.0000%	0.0000%
372.39	Other and unspecified conjunctivitis	0.0005%	0.0000%	0.0000%	0.0000%
385.02	Tympanosclerosis involving tympanic membrane and ear o	0.0005%	0.0000%	0.0000%	0.0000%
458.2	Iatrogenic hypotension	0.0000%	0.0000%	0.0000%	0.0032%
374.23	Cicatricial lagophthalmos	0.0000%	0.0000%	0.0021%	0.0000%
390	Rheumatic fever without mention of heart involvement	0.0005%	0.0000%	0.0000%	0.0000%
385.03	ympanosclerosis involving tympanic membrane, ear ossicle	0.0005%	0.0000%	0.0000%	0.0000%
424.3	Pulmonary valve disorders	0.0000%	0.0000%	0.0000%	0.0032%
380.31	Hematoma of auricle or pinna	0.0005%	0.0000%	0.0000%	0.0000%
482.81	Pneumonia due to Anaerobes	0.0005%	0.0000%	0.0000%	0.0000%
378.53	Fourth or trochlear nerve palsy	0.0005%	0.0000%	0.0000%	0.0000%
448.1	Nevus, non-neoplastic	0.0005%	0.0000%	0.0000%	0.0000%
385.22	Impaired mobility of other ear ossicles	0.0005%	0.0000%	0.0000%	0.0000%
375.01	Acute dacryoadenitis	0.0005%	0.0000%	0.0000%	0.0000%
464.20	Acute laryngotracheitis without mention of obstruction	0.0000%	0.0000%	0.0000%	0.0032%
392.9	Rheumatic chorea without mention of heart involvement	0.0005%	0.0000%	0.0000%	0.0000%
376.6	Retained (old) foreign body following penetrating wound o	0.0005%	0.0000%	0.0000%	0.0000%
386.19	Other and unspecified peripheral vertigo	0.0000%	0.0048%	0.0000%	0.0000%
519.01	Infection of tracheostomy	0.0000%	0.0048%	0.0000%	0.0000%
377.30	Optic neuritis, unspecified	0.0000%	0.0000%	0.0021%	0.0000%
363.8	Other disorders of choroid	0.0000%	0.0000%	0.0021%	0.0000%
379.33	Anterior dislocation of lens	0.0005%	0.0000%	0.0000%	0.0000%
377.49	Other disorders of optic nerve	0.0000%	0.0000%	0.0021%	0.0000%
375.02	Chronic dacryoadenitis	0.0000%	0.0000%	0.0000%	0.0032%
410.92	Acute myocardial infarction of unspecified site, subsequent	0.0005%	0.0000%	0.0000%	0.0000%
376.82	Myopathy of extraocular muscles	0.0000%	0.0048%	0.0000%	0.0000%
370.04	Hypopyon ulcer	0.0005%	0.0000%	0.0000%	0.0000%
378.35	Accommodative component in esotropia	0.0005%	0.0000%	0.0000%	0.0000%
368.40	Visual field defect, unspecified	0.0005%	0.0000%	0.0000%	0.0000%
383.33	Granulations of postmastoidectomy cavity	0.0005%	0.0000%	0.0000%	0.0000%
521.5	Hypercementosis	0.0000%	0.0048%	0.0000%	0.0000%
487.8	Influenza with other manifestations	0.0000%	0.0000%	0.0021%	0.0000%
522.0	Pulpitis	0.0005%	0.0000%	0.0000%	0.0000%
426.11	First degree atrioventricular block	0.0005%	0.0000%	0.0000%	0.0000%
378.71	Duane's syndrome	0.0005%	0.0000%	0.0000%	0.0000%
448.9	Other and unspecified capillary diseases	0.0000%	0.0000%	0.0021%	0.0000%
372.45	Recurrent pterygium	0.0005%	0.0000%	0.0000%	0.0000%
491.8	Other chronic bronchitis	0.0000%	0.0000%	0.0021%	0.0000%
491.9	Unspecified chronic bronchitis	0.0000%	0.0000%	0.0021%	0.0000%

ICD-9-CM	ICD-9-CM	PureO	ContiB	NewB	PastB
291.0	Alcohol withdrawal delirium	0.0000%	0.0000%	0.0000%	0.0032%
360.03	Chronic endophthalmitis	0.0000%	0.0000%	0.0021%	0.0000%
234.8	Carcinoma in situ of other specified sites	0.0000%	0.0000%	0.0000%	0.0032%
244.3	Other iatrogenic hypothyroidism	0.0000%	0.0000%	0.0000%	0.0032%
361.07	Old retinal detachment, total or subtotal	0.0000%	0.0000%	0.0000%	0.0032%
337.0	Idiopathic peripheral autonomic neuropathy	0.0005%	0.0000%	0.0000%	0.0000%
230.5	Carcinoma in situ of anal canal	0.0005%	0.0000%	0.0000%	0.0000%
283.19	Other non-autoimmune hemolytic anemias, unspecified	0.0000%	0.0048%	0.0000%	0.0000%
306.4	Psychogenic gastrointestinal malfunction	0.0005%	0.0000%	0.0000%	0.0000%
231.8	Carcinoma in situ of other specified parts of respiratory sys	0.0005%	0.0000%	0.0000%	0.0000%
360.89	Other disorders of globe	0.0005%	0.0000%	0.0000%	0.0000%
341.8	Other demyelinating diseases of central nervous system	0.0000%	0.0000%	0.0000%	0.0032%
252.1	Hypoparathyroidism	0.0000%	0.0048%	0.0000%	0.0000%
212.2	Benign neoplasm of trachea	0.0005%	0.0000%	0.0000%	0.0000%
232.2	Carcinoma in situ of ear and external auditory canal	0.0000%	0.0000%	0.0000%	0.0032%
235.1	Neoplasm of uncertain behavior of lip, oral cavity and phar	0.0005%	0.0000%	0.0000%	0.0000%
355.79	Other mononeuritis of lower limb	0.0005%	0.0000%	0.0000%	0.0000%
225.8	Benign neoplasm of other specified sites of nervous system	0.0005%	0.0000%	0.0000%	0.0000%
357.6	Polyneuropathy due to drugs	0.0000%	0.0048%	0.0000%	0.0000%
317	Mild mental retardation	0.0000%	0.0000%	0.0000%	0.0032%
307.20	Tic disorder, unspecified	0.0005%	0.0000%	0.0000%	0.0000%
318.0	Moderate mental retardation	0.0005%	0.0000%	0.0000%	0.0000%
295.80	Other specified types of schizophrenia, unspecified	0.0000%	0.0048%	0.0000%	0.0000%
343.3	Monoplegic infantile cerebral palsy	0.0005%	0.0000%	0.0000%	0.0000%
335.22	Progressive bulbar palsy	0.0005%	0.0000%	0.0000%	0.0000%
285.22	Anemia in neoplastic disease	0.0005%	0.0000%	0.0000%	0.0000%
275.40	Unspecified disorders of calcium metabolism	0.0000%	0.0048%	0.0000%	0.0000%
319	Unspecified mental retardation	0.0005%	0.0000%	0.0000%	0.0000%
296.7	Bipolar affective disorder, unspecified	0.0000%	0.0048%	0.0000%	0.0000%
344.09	Other guadriplegia and quadriparesis	0.0000%	0.0000%	0.0000%	0.0032%
272.1	Pure hyperglyceridemia	0.0000%	0.0000%	0.0000%	0.0032%
285.29	Anemia of other chronic illness	0.0000%	0.0048%	0.0000%	0.0000%
292.2	Pathological drug intoxication	0.0000%	0.0048%	0.0000%	0.0000%
296.42	Bipolar affective disorder, manic, moderate	0.0000%	0.0000%	0.0000%	0.0032%
333.4	Huntington's chorea	0.0005%	0.0000%	0.0000%	0.0000%
240.0	Goiter, specified as simple	0.0000%	0.0000%	0.0000%	0.0032%
294.0	Amnestic syndrome	0.0000%	0.0048%	0.0000%	0.0000%
300.11	Conversion disorder	0.0000%	0.0000%	0.0000%	0.0032%
295.24	Schizophrenic disorders, catatonic type, chronic with acute	0.0000%	0.0000%	0.0000%	0.0032%

ICD-9-CM	ICD-9-CM	PureO	ContiB	NewB	PastB
344.89	Other specified paralytic syndromes	0.0005%	0.0000%	0.0000%	0.0000%
357.9	Unspecified inflammatory and toxic neuropathy	0.0000%	0.0000%	0.0021%	0.0000%
320.89	Meningitis due to other specified bacteria	0.0000%	0.0000%	0.0021%	0.0000%
359.3	Familial periodic paralysis	0.0000%	0.0048%	0.0000%	0.0000%
255.3	Other corticoadrenal overactivity	0.0000%	0.0048%	0.0000%	0.0000%
333.99	Other extrapyramidal diseases and abnormal movement dis	0.0000%	0.0048%	0.0000%	0.0000%
205.90	Unspecified myeloid leukemia, without mention of remissi	0.0000%	0.0048%	0.0000%	0.0000%
334.2	Primary cerebellar degeneration	0.0005%	0.0000%	0.0000%	0.0000%
362.29	Other nondiabetic proliferative retinopathy	0.0000%	0.0048%	0.0000%	0.0000%
360.43	Hemophthalmos, except current injury	0.0000%	0.0000%	0.0000%	0.0032%
276.3	Alkalosis	0.0005%	0.0000%	0.0000%	0.0000%
295.94	Unspecified schizophrenia, chronic with acute exacerbation	0.0000%	0.0000%	0.0000%	0.0032%
322.1	Eosinophilic meningitis	0.0000%	0.0000%	0.0000%	0.0032%
362.65	Secondary pigmentary degeneration	0.0005%	0.0000%	0.0000%	0.0000%
216.0	Benign neoplasm of skin of lip	0.0005%	0.0000%	0.0000%	0.0000%
361.30	Retinal defect, unspecified	0.0005%	0.0000%	0.0000%	0.0000%
345.70	Epilepsia partialis continua without mention of intractable	0.0005%	0.0000%	0.0000%	0.0000%
221.9	Benign neoplasm of female genital organ, site unspecified	0.0000%	0.0000%	0.0000%	0.0032%
322.2	Chronic meningitis	0.0000%	0.0000%	0.0000%	0.0032%
205.11	Chronic myeloid leukemia, in remission	0.0000%	0.0000%	0.0000%	0.0032%
345.81	Other forms of epilepsy with intractable epilepsy	0.0000%	0.0000%	0.0021%	0.0000%
290.42	Arteriosclerotic dementia with delusional features	0.0000%	0.0000%	0.0000%	0.0032%
296.52	Bipolar affective disorder, depressed, moderate	0.0000%	0.0000%	0.0000%	0.0032%
279.8	Other specified disorders involving the immune mechanism	0.0000%	0.0000%	0.0021%	0.0000%
233.5	Carcinoma in situ of penis	0.0000%	0.0000%	0.0000%	0.0032%
213.9	Benign neoplasm of bone and articular cartilage, site unspe	0.0000%	0.0048%	0.0000%	0.0000%
245.8	Other and unspecified chronic thyroiditis	0.0005%	0.0000%	0.0000%	0.0000%
272.7	Lipidoses	0.0005%	0.0000%	0.0000%	0.0000%
232.8	Carcinoma in situ of other specified sites of skin	0.0000%	0.0000%	0.0021%	0.0000%
296.89	Other manic-depressive psychosis	0.0000%	0.0048%	0.0000%	0.0000%
296.53	Bipolar affective disorder, depressed, severe without menti	0.0000%	0.0048%	0.0000%	0.0000%
355.4	Lesion of medial popliteal nerve	0.0005%	0.0000%	0.0000%	0.0000%
257.8	Other testicular dysfunction	0.0005%	0.0000%	0.0000%	0.0000%
305.00	Alcohol abuse, unspecified	0.0000%	0.0000%	0.0000%	0.0032%
346.91	Migraine, unspecified，with intractable migraine，so state	0.0000%	0.0000%	0.0021%	0.0000%
293.9	Unspecified transient organic mental disorder	0.0005%	0.0000%	0.0000%	0.0000%
279.03	Other selective immunoglobulin deficiency	0.0005%	0.0000%	0.0000%	0.0000%
333.6	Idiopathic torsion dystonia	0.0000%	0.0000%	0.0021%	0.0000%
288.2	Genetic anomalies of leukocytes	0.0000%	0.0000%	0.0021%	0.0000%

ICD-9-CM	ICD-9-CM	PureO	ContiB	NewB	PastB
295.20	Schizophrenic disorders, catatonic type, unspecified	0.0000%	0.0000%	0.0000%	0.0032%
259.1	Precocious sexual development and puberty, not elsewhere	0.0005%	0.0000%	0.0000%	0.0000%
357.5	Alcoholic polyneuropathy	0.0000%	0.0048%	0.0000%	0.0000%
279.06	Common variable immunodeficiency	0.0000%	0.0000%	0.0021%	0.0000%
250.51	Diabetes with ophthalmic manifestations, Type I [insulin de	0.0005%	0.0000%	0.0000%	0.0000%
288.9	Unspecified disease of white blood cells	0.0000%	0.0048%	0.0000%	0.0000%
250.73	Diabetes with peripheral circulatory disorders, Type I [insu	0.0005%	0.0000%	0.0000%	0.0000%
261	Nutritional marasmus	0.0005%	0.0000%	0.0000%	0.0000%
359.1	Hereditary progressive muscular dystrophy	0.0000%	0.0048%	0.0000%	0.0000%
250.32	Diabetes with other coma, Type II [non-insulin dependent t	0.0000%	0.0000%	0.0000%	0.0032%
296.90	Unspecified affective psychoses	0.0000%	0.0048%	0.0000%	0.0000%
265.1	Other and unspecified manifestations of thiamine deficienc	0.0005%	0.0000%	0.0000%	0.0000%
333.92	Neuroloptic malignant syndrome	0.0000%	0.0048%	0.0000%	0.0000%
279.12	Wiskott-Aldrich syndrome	0.0005%	0.0000%	0.0000%	0.0000%
334.1	Hereditary spastic paraplegia	0.0005%	0.0000%	0.0000%	0.0000%
289.50	Diseases of spleen, unspecified	0.0005%	0.0000%	0.0000%	0.0000%
360.12	Panuveitis	0.0005%	0.0000%	0.0000%	0.0000%
330.8	Other specified cerebral degenerations in childhood	0.0000%	0.0000%	0.0021%	0.0000%
360.21	Progressive high (degenerative) myopia	0.0005%	0.0000%	0.0000%	0.0000%
349.82	Toxic encephalopathy	0.0000%	0.0048%	0.0000%	0.0000%
360.42	Blind hypertensive eye	0.0000%	0.0000%	0.0021%	0.0000%
268.0	Rickets, active	0.0005%	0.0000%	0.0000%	0.0000%
334.3	Other cerebellar ataxia	0.0005%	0.0000%	0.0000%	0.0000%
270.0	Disturbances of amino-acid transport	0.0005%	0.0000%	0.0000%	0.0000%
235.6	Neoplasm of uncertain behavior of larynx	0.0005%	0.0000%	0.0000%	0.0000%
270.6	Disorders of urea cycle metabolism	0.0005%	0.0000%	0.0000%	0.0000%
250.61	Diabetes with neurological manifestations, Type I [insulin d	0.0005%	0.0000%	0.0000%	0.0000%
227.5	Benign neoplasm of carotid body	0.0000%	0.0000%	0.0021%	0.0000%
361.04	Recent retinal detachment, partial, with retinal dialysis	0.0005%	0.0000%	0.0000%	0.0000%
271.0	Glycogenosis	0.0005%	0.0000%	0.0000%	0.0000%
361.06	Old retinal detachment, partial	0.0005%	0.0000%	0.0000%	0.0000%
279.2	Combined immunity deficiency	0.0005%	0.0000%	0.0000%	0.0000%
361.10	Retinoschisis, unspecified	0.0005%	0.0000%	0.0000%	0.0000%
352.1	Glossopharyngeal neuralgia	0.0000%	0.0000%	0.0021%	0.0000%
242.31	Toxic nodular goiter, unspecified, with mention of thyrotox	0.0005%	0.0000%	0.0000%	0.0000%
352.3	Disorders of pneumogastric〔10th〕 nerve	0.0005%	0.0000%	0.0000%	0.0000%
250.00	Diabetes mellitus without mention of complication, Type II	0.0005%	0.0000%	0.0000%	0.0000%
352.5	Disorders of hypoglossal〔12th〕 nerve	0.0000%	0.0000%	0.0000%	0.0032%
290.3	Senile dementia with delirium	0.0000%	0.0000%	0.0021%	0.0000%

ICD-9-CM	ICD-9-CM	PureO	ContiB	NewB	PastB
362.35	Central retinal vein occlusion	0.0000%	0.0000%	0.0021%	0.0000%
345.2	Petit mal status	0.0005%	0.0000%	0.0000%	0.0000%
208.20	Subacute leukemia of unspecified cell type, without mentic	0.0005%	0.0000%	0.0000%	0.0000%
233.4	Carcinoma in situ of prostate	0.0005%	0.0000%	0.0000%	0.0000%
250.71	Diabetes with peripheral circulatory disorders, Type I [insu	0.0000%	0.0000%	0.0000%	0.0032%
255.5	Other adrenal hypofunction	0.0000%	0.0048%	0.0000%	0.0000%
276.0	Hyperosmolality and/or hypernatremia	0.0000%	0.0000%	0.0000%	0.0032%
015.84	Other specified joint tuberculosis, tubercle bacilli not founc	0.0000%	0.0000%	0.0000%	0.0032%
003.9	Salmonella infection , unspecified	0.0005%	0.0000%	0.0000%	0.0000%
010.15	Tuberculous pleurisy in primary progressive tuberculosis, t	0.0005%	0.0000%	0.0000%	0.0000%
101	Vincent's angina	0.0000%	0.0048%	0.0000%	0.0000%
017.04	Tuberculosis of skin and subcutaneous cellular tissue, tuber	0.0005%	0.0000%	0.0000%	0.0000%
011.91	Pulmonary tuberculosis, unspecified, bacteriological or hist	0.0005%	0.0000%	0.0000%	0.0000%
091.9	Unspecified secondary syphilis	0.0005%	0.0000%	0.0000%	0.0000%
189.9	Malignant neoplasm of urinary organ, site unspecified	0.0000%	0.0048%	0.0000%	0.0000%
011.24	Tuberculosis of lung with cavitation, tubercle bacilli not fo	0.0000%	0.0048%	0.0000%	0.0000%
137.0	Late effects of respiratory or unspecified tuberculosis	0.0005%	0.0000%	0.0000%	0.0000%
170.8	Malignant neoplasm of short bones of lower limb	0.0005%	0.0000%	0.0000%	0.0000%
172.1	Malignant melanoma of skin of eyelid, including canthus	0.0005%	0.0000%	0.0000%	0.0000%
040.81	Tropical pyomyositis	0.0005%	0.0000%	0.0000%	0.0000%
015.93	Tuberculosis of unspecified bones and joints, tubercle bacil	0.0000%	0.0000%	0.0021%	0.0000%
013.01	Tuberculous meningitis, bacteriological or histological exan	0.0005%	0.0000%	0.0000%	0.0000%
146.6	Malignant neoplasm of lateral wall of oropharynx	0.0000%	0.0000%	0.0021%	0.0000%
081.0	Murine〔endemic〕typhus	0.0005%	0.0000%	0.0000%	0.0000%
138	Late effects of acute poliomyelitis	0.0000%	0.0000%	0.0021%	0.0000%
184.2	Malignant neoplasm of labia minora	0.0005%	0.0000%	0.0000%	0.0000%
190.6	Malignant neoplasm of choroid	0.0005%	0.0000%	0.0000%	0.0000%
017.93	Tuberculosis of other specified organ, tubercle bacilli founc	0.0000%	0.0000%	0.0021%	0.0000%
015.85	Other specified joint tuberculosis, tubercle bacilli not founc	0.0005%	0.0000%	0.0000%	0.0000%
016.46	Tuberculosis of epididymis, tubercle bacilli not found by ba	0.0005%	0.0000%	0.0000%	0.0000%
017.06	Tuberculosis of skin and subcutaneous cellular tissue, tuber	0.0005%	0.0000%	0.0000%	0.0000%
170.9	Malignant neoplasm of bone and articular cartilage, site un	0.0000%	0.0000%	0.0021%	0.0000%
147.1	Malignant neoplasm of posterior wall of nasopharynx	0.0005%	0.0000%	0.0000%	0.0000%
081.2	Scrub typhus	0.0005%	0.0000%	0.0000%	0.0000%
008.63	Enteritis due to Norwalk virus	0.0005%	0.0000%	0.0000%	0.0000%
141.5	Malignant neoplasm of tongue of junctional zone	0.0005%	0.0000%	0.0000%	0.0000%
040.89	Other specified bacterial diseases	0.0005%	0.0000%	0.0000%	0.0000%
200.04	Reticulosarcoma, lymph nodes of axilla and upper limb	0.0005%	0.0000%	0.0000%	0.0000%
110.0	Dermatophytosis of scalp and beard	0.0005%	0.0000%	0.0000%	0.0000%

ICD-9-CM	ICD-9-CM	PureO	ContiB	NewB	PastB
200.12	Lymphosarcoma, intrathoracic lymph nodes	0.0005%	0.0000%	0.0000%	0.0000%
015.96	Tuberculosis of unspecified bones and joints, tubercle bacil	0.0000%	0.0000%	0.0000%	0.0032%
182.8	Malignant neoplasm of other specified sites of body of uter	0.0005%	0.0000%	0.0000%	0.0000%
012.01	Tuberculous pleurisy, bacteriological or histological exami	0.0000%	0.0000%	0.0021%	0.0000%
183.8	Malignant neoplasm of other specified sites of uterine adne	0.0000%	0.0048%	0.0000%	0.0000%
005.9	Food poisoning, unspecified	0.0005%	0.0000%	0.0000%	0.0000%
112.85	Candidal enteritis	0.0000%	0.0048%	0.0000%	0.0000%
142.8	Malignant neoplasm of other major salivary glands	0.0000%	0.0048%	0.0000%	0.0000%
016.33	Tuberculosis of other urinary organs, tubercle bacilli found	0.0000%	0.0000%	0.0000%	0.0032%
041.02	Group B streptococcus infections of unspecified site	0.0000%	0.0000%	0.0021%	0.0000%
201.93	Hodgkin’ s disease, unspecified, intra-abdominal lymph n	0.0005%	0.0000%	0.0000%	0.0000%
041.4	Escherichia coli〔E.coli〕infections of unspeciied site	0.0000%	0.0000%	0.0021%	0.0000%
008.46	Intestinal infections due to other anaerobes	0.0000%	0.0000%	0.0000%	0.0032%
041.85	Other Gram-negative organisms infections of unspecified s	0.0005%	0.0000%	0.0000%	0.0000%
016.35	Tuberculosis of other urinary organs, tubercle bacilli not fo	0.0000%	0.0000%	0.0021%	0.0000%
192.9	Malignant neoplasm of nervous system, part unspecified	0.0005%	0.0000%	0.0000%	0.0000%
187.8	Malignant neoplasm of other specified sites of male genital	0.0005%	0.0000%	0.0000%	0.0000%
041.9	Unspecified bacterial infection of unspecified site	0.0000%	0.0000%	0.0021%	0.0000%
202.50	Letterer-Siwe disease, unspecified site, extranodal solid org	0.0005%	0.0000%	0.0000%	0.0000%
015.52	Limb bones tuberculosis, bacteriological or histological exa	0.0000%	0.0000%	0.0021%	0.0000%
159.8	Malignant neoplasm of other sites of digestive system and	0.0005%	0.0000%	0.0000%	0.0000%
046.1	Jakob-Creutzfeldt disease	0.0000%	0.0000%	0.0021%	0.0000%
202.86	Other lymphomas, intrapelvic lymph nodes	0.0000%	0.0048%	0.0000%	0.0000%
015.22	Knee tuberculosis, bacteriological or histological examinati	0.0005%	0.0000%	0.0000%	0.0000%
203.01	Multiple myeloma, in remission	0.0000%	0.0000%	0.0021%	0.0000%
194.5	Malignant neoplasm of carotid body	0.0005%	0.0000%	0.0000%	0.0000%
013.33	Tuberculous abscess of brain, tubercle bacilli found (in spu	0.0000%	0.0000%	0.0000%	0.0032%
110.3	Dermatophytosis of groin and perianal area	0.0005%	0.0000%	0.0000%	0.0000%
017.85	Tuberculosis of esophagus, tubercle bacilli not found by ba	0.0000%	0.0000%	0.0000%	0.0032%
195.1	Malignant neoplasm of other and ill-defined sites of thorax	0.0005%	0.0000%	0.0000%	0.0000%
200.02	Reticulosarcoma, intrathoracic lymph nodes	0.0005%	0.0000%	0.0000%	0.0000%
148.8	Malignant neoplasm of other specified sites of hypopharyn	0.0005%	0.0000%	0.0000%	0.0000%
017.90	Tuberculosis of other specified organ, unspecified	0.0005%	0.0000%	0.0000%	0.0000%
173.9	Malignant neoplasm of skin, site unspecified	0.0005%	0.0000%	0.0000%	0.0000%
143.8	Malignant neoplasm of other sites of gum	0.0000%	0.0000%	0.0021%	0.0000%
046.3	Progressive multifocal leukoencephalopathy	0.0000%	0.0000%	0.0021%	0.0000%
200.13	Lymphosarcoma, intra-abdominal lymph nodes	0.0005%	0.0000%	0.0000%	0.0000%
015.53	Limb bones tuberculosis, tubercle bacilli found (in sputum)	0.0005%	0.0000%	0.0000%	0.0000%
182.1	Malignant neoplasm of isthmus uteri	0.0005%	0.0000%	0.0000%	0.0000%

ICD-9-CM	ICD-9-CM	PureO	ContiB	NewB	PastB
110.4	Dermatophytosis of foot	0.0005%	0.0000%	0.0000%	0.0000%
013.03	Tuberculous meningitis, tubercle bacilli found (in sputum)	0.0005%	0.0000%	0.0000%	0.0000%
131.00	Urogenital trichomoniasis, unspecified	0.0005%	0.0000%	0.0000%	0.0000%
200.80	Other named variants lymphoma, unspecified site, extranod	0.0005%	0.0000%	0.0000%	0.0000%
054.42	Dendritic keratitis	0.0005%	0.0000%	0.0000%	0.0000%
201.48	Lymphocytic-histiocytic predominance, lymph nodes of mu	0.0000%	0.0000%	0.0021%	0.0000%
049.8	Other specified non-arthropod-borne viral diseases of centr	0.0005%	0.0000%	0.0000%	0.0000%
144.1	Malignant neoplasm of floor of mouth, lateral portion	0.0000%	0.0000%	0.0021%	0.0000%
078.19	Other specified viral warts	0.0005%	0.0000%	0.0000%	0.0000%
159.0	Malignant neoplasm of intestinal tract, part unspecified	0.0005%	0.0000%	0.0000%	0.0000%
015.24	Knee tuberculosis, tubercle bacilli not found (in sputum) by	0.0005%	0.0000%	0.0000%	0.0000%
201.63	Mixed cellularity, intra-abdominal lymph nodes	0.0000%	0.0000%	0.0021%	0.0000%
038.44	Septicemia due to Serratia	0.0000%	0.0000%	0.0021%	0.0000%
011.22	Tuberculosis of lung with cavitation, bacteriological or hist	0.0000%	0.0000%	0.0000%	0.0032%
110.5	Dermatophytosis of the body	0.0000%	0.0048%	0.0000%	0.0000%
170.5	Malignant neoplasm of short bones of upper limb	0.0005%	0.0000%	0.0000%	0.0000%
014.83	Other tuberculosis of intestines, peritoneum, and mesenteri	0.0000%	0.0000%	0.0000%	0.0032%
015.26	Knee tuberculosis, tubercle bacilli not found by bacteriolog	0.0005%	0.0000%	0.0000%	0.0000%
012.85	Other specified respiratory tuberculosis, tubercle bacilli not	0.0005%	0.0000%	0.0000%	0.0000%
017.94	Tuberculosis of other specified organ, tubercle bacilli not fo	0.0000%	0.0048%	0.0000%	0.0000%
015.12	Hip tuberculosis, bacteriological or histological examinatio	0.0000%	0.0000%	0.0021%	0.0000%
202.04	Nodular lymphoma, lymph nodes of axilla and upper limb	0.0000%	0.0000%	0.0021%	0.0000%
054.5	Herpetic septicemia	0.0000%	0.0048%	0.0000%	0.0000%
202.06	Nodular lymphoma, intrapelvic lymph nodes	0.0000%	0.0000%	0.0021%	0.0000%
014.84	Other tuberculosis of intestines, peritoneum, and mesenteri	0.0000%	0.0048%	0.0000%	0.0000%
187.6	Malignant neoplasm of spermatic cord	0.0005%	0.0000%	0.0000%	0.0000%
160.4	Malignant neoplasm of frontal sinus	0.0000%	0.0000%	0.0021%	0.0000%
202.12	Mycosis fungoides, intrathoracic lymph nodes	0.0005%	0.0000%	0.0000%	0.0000%
054.72	Herpes simplex meningitis	0.0005%	0.0000%	0.0000%	0.0000%
117.4	Mycotic mycetomas	0.0005%	0.0000%	0.0000%	0.0000%
016.05	Tuberculosis of kidney, tubercle bacilli not found by bacter	0.0005%	0.0000%	0.0000%	0.0000%
202.40	Leukemic reticuloendotheliosis, unspecified site, extranoda	0.0005%	0.0000%	0.0000%	0.0000%
015.13	Hip tuberculosis, tubercle bacilli found (in sputum) by micr	0.0005%	0.0000%	0.0000%	0.0000%
202.51	Letterer-Siwe disease, lymph nodes of head, face and neck	0.0005%	0.0000%	0.0000%	0.0000%
176.5	Kaposi's sarcoma of lymph nodes	0.0005%	0.0000%	0.0000%	0.0000%
004.9	Shigellosis, unspecified	0.0005%	0.0000%	0.0000%	0.0000%
015.01	Vertebral column tuberculosis, bacteriological or histologic	0.0000%	0.0048%	0.0000%	0.0000%
039.3	Cervicofacial diseases due to actinomycotic infections	0.0000%	0.0000%	0.0000%	0.0032%
015.56	Limb bones tuberculosis, tubercle bacilli not found by bact	0.0000%	0.0000%	0.0000%	0.0032%

ICD-9-CM	ICD-9-CM	PureO	ContiB	NewB	PastB
011.45	Tuberculous fibrosis of lung, tubercle bacilli not found by b	0.0005%	0.0000%	0.0000%	0.0000%
164.8	Malignant neoplasm of thymus, heart, and mediastinum, ot	0.0005%	0.0000%	0.0000%	0.0000%
011.62	Tuberculous pneumonia〔any form〕, bacteriological or h	0.0000%	0.0000%	0.0021%	0.0000%
062.0	Japanese encephalitis	0.0005%	0.0000%	0.0000%	0.0000%
099.1	Lymphogranuloma venereum	0.0005%	0.0000%	0.0000%	0.0000%
112.3	Candidiasis of skin and nails	0.0005%	0.0000%	0.0000%	0.0000%
040.0	Gas gangrene	0.0005%	0.0000%	0.0000%	0.0000%
123.1	Cysticercosis	0.0000%	0.0048%	0.0000%	0.0000%
015.75	Other specified bone tuberculosis, tubercle bacilli not found	0.0005%	0.0000%	0.0000%	0.0000%
140.6	Malignant neoplasm of commissure of lip	0.0000%	0.0000%	0.0021%	0.0000%
011.73	Tuberculous pneumothorax, tubercle bacilli found (in sputu	0.0005%	0.0000%	0.0000%	0.0000%
009.1	Colitis, enteritis, and gastroenteritis of presumed infectious	0.0000%	0.0000%	0.0000%	0.0032%
013.64	Tuberculous encephalitis or myelitis, tubercle bacilli not fo	0.0000%	0.0048%	0.0000%	0.0000%
136.8	Other specified infectious and parasitic diseases	0.0000%	0.0000%	0.0021%	0.0000%
015.54	Limb bones tuberculosis, tubercle bacilli not found (in sput	0.0000%	0.0048%	0.0000%	0.0000%
		100.0000%	100.0000%	100.0000%	100.0000%
